# Preparation of *N*_In_-Methyl-6-[^18^F]fluoro- and 5-Hydroxy-7-[^18^F]fluorotryptophans as Candidate PET-Tracers for Pathway-Specific Visualization of Tryptophan Metabolism

**DOI:** 10.3390/ijms242015251

**Published:** 2023-10-17

**Authors:** Niklas Kolks, Felix Neumaier, Bernd Neumaier, Boris D. Zlatopolskiy

**Affiliations:** 1 Institute of Neuroscience and Medicine, Nuclear Chemistry (INM-5), Forschungszentrum Jülich GmbH, Wilhelm-Johnen-Str., 52428 Jülich, Germany; niklas.kolks@uk-koeln.de (N.K.); f.neumaier@fz-juelich.de (F.N.); boris.zlatopolskiy@uk-koeln.de (B.D.Z.); 2 Institute of Radiochemistry and Experimental Molecular Imaging, Faculty of Medicine and University Hospital Cologne, University of Cologne, Kerpener Str. 62, 50937 Cologne, Germany

**Keywords:** positron emission tomography (PET) imaging, radiotracer, radiofluorination, tryptophan metabolism, serotonin synthesis, kynurenine pathway, indoleamine-2,3-dioxygenase (IDO), tryptophan hydroxylase (TPH)

## Abstract

Tryptophan (Trp) is an essential proteinogenic amino acid and metabolic precursor for several signaling molecules that has been implicated in many physiological and pathological processes. Since the two main branches of Trp metabolism—serotonin biosynthesis and kynurenine pathway—are differently affected by a variety of neurological and neoplastic diseases, selective visualization of these pathways is of high clinical relevance. However, while positron emission tomography (PET) with existing probes can be used for non-invasive assessment of total Trp metabolism, optimal imaging agents for pathway-specific PET imaging are still lacking. In this work, we describe the preparation of two ^18^F-labeled Trp derivatives, *N*_In_-methyl-6-[^18^F]fluorotryptophan (*N*_In_-Me-6-[^18^F]FTrp) and 5-hydroxy-7-[^18^F]fluorotryptophan (5-HO-7-[^18^F]FTrp). We also report feasible synthetic routes for the preparation of the hitherto unknown boronate radiolabeling precursors and non-radioactive reference compounds. Under optimized conditions, alcohol-enhanced Cu-mediated radiofluorination of the respective precursors afforded *N*_In_-Me-6-[^18^F]FTrp and 5-HO-7-[^18^F]FTrp as application-ready solutions in radiochemical yields of 45 ± 7% and 29 ± 4%, respectively. As such, our work provides access to two promising candidate probes for pathway-specific visualization of Trp metabolism in amounts sufficient for their preclinical evaluation.

## 1. Introduction

**Figure 1:**
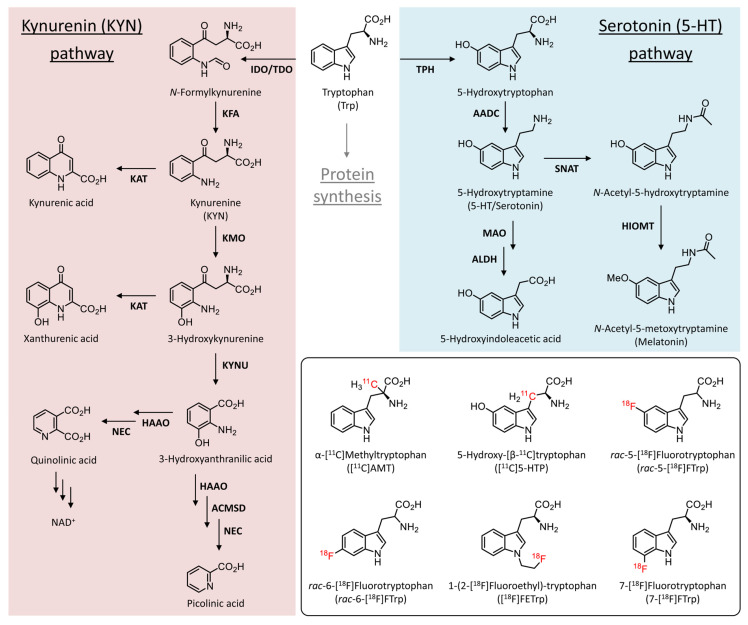
Pathways of tryptophan (Trp) metabolism and selected radiotracers evaluated as PET probes for their visualization. Shown are the key metabolites formed and the metabolizing enzymes involved in processing of Trp via the kynurenine (**left**, indicated in red) or serotonin (**right**, indicated in blue) pathways, respectively. In the inset (**bottom-right**), selected examples of ^11^C- and ^18^F-labeled Trp derivatives previously evaluated as PET-tracers are illustrated. Abbreviations: AADC, aromatic amino acid decarboxylase; ACMSD, aminocarboxymuconate semialdehyde decarboxylase; HAAO, 3-hydroxyanthanilic acid oxygenase; HIOMT, hydroxyindole *o*-methyltransferase; IDO, indoleamine-2,3-dioxygenase; KAT, kynurenine aminotransferases; KFA, kynurenine formamidase; KMO, kynurenine 3-monooxygenase; KYNU, kynureninase; NAD^+^, nicotinamide adenine dinucleotide; NEC, non-enzymatic cyclization; SNAT, serotonin *N*-acetyl transferase; TDO, tryptophan-2,3-dioxygenase; TPH, tryptophan hydroxylase.

Tryptophan (Trp) is an essential proteinogenic amino acid with an indole ring in the side chain. It is the least abundant amino acid in animal proteins (approx. 1.4%) and serves mainly as a metabolic precursor for a plethora of bioactive molecules (Figure 1) [1,2].

Under physiological conditions, over 95% of dietary Trp enters the kynurenine (KYN) pathway and is metabolized through cleavage of the indole ring by the rate-limiting enzymes tryptophan-2,3-dioxygenase (TDO) in the liver or indoleamine-2,3-dioxygenase (IDO) in other tissues [1,2]. Depending on the exact metabolic route, subsequent steps can produce an array of bioactive intermediates that may exert either neuroprotective or neurotoxic effects (Figure 1). While the concentration of toxic Trp metabolites in the normal brain is low, local upregulation of IDO or changes in the activity of downstream enzymes can result in significant built up of KYN and other intermediates in neuroinflammatory, neurodegenerative, and neuropsychiatric disorders [1,2,3,4]. In addition, accelerated Trp metabolism due to upregulation of IDO has been linked to tumor immune escape and has been shown to correlate with a poor prognosis in several types of cancer [5,6].

A minor fraction (1–2%) of dietary Trp enters the serotonin pathway and is converted into the neurotransmitter serotonin (5-hydroxytryptamine or 5-HT), which is mainly (≈90%) formed in the gastrointestinal tract but also present in the brain (≈5%) [7]. Serotonin is biosynthesized through 5-hydroxylation of the indole ring in Trp by the rate-limiting enzyme tryptophan hydroxylase (TPH) and subsequent decarboxylation by aromatic amino acid decarboxylase (AADC). In the pineal gland, serotonin may be further transformed into melatonin (Figure 1). Serotonergic signaling is well known for its multifaceted role in emotional and cognitive processing under physiological conditions. Furthermore, a number of neuropsychiatric and neurodegenerative disorders have been shown to be associated with dysfunction or loss of serotonergic neurons in the brain [8,9,10,11]. In addition, neuroinflammation-driven changes in KYN pathway activity in patients with neuropsychiatric disorders can reduce serotonin synthesis by lowering the Trp availability and suppressing the activity of TPH [12].

Given that several pathological conditions are associated with simultaneous and often opposite changes in KYN pathway activity and serotonin synthesis, selective visualization of the different branches of Trp metabolism could help in the understanding, prognosis and therapy of many disorders. However, optimal PET-tracers for pathway-specific imaging of Trp metabolism are still lacking. Thus, while ^11^C-labeled tracers like α-[^11^C]methyltryptophan ([^11^C]AMT) and 5-hydroxy-[β-^11^C]tryptophan ([^11^C]5-HTP) (Figure 1) have proven useful for imaging Trp metabolism and/or serotonin synthesis [13,14,15,16], their application is limited by several inherent shortcomings, such as the short half-life of carbon-11 (t_1/2_ = 20 min) and cumbersome production routes. Fluorine-18 possesses more favorable characteristics, like a longer half-life (t_1/2_ = 109 min) and lower kinetic energy of emitted positrons. Although the first ^18^F-labeled racemic fluorotryptophans (*rac*-5- and *rac*-6-[^18^F]fluorotryptophan, Figure 1) were already described in 1972, their preparation suffered from low molar activities and radiochemical yields (RCYs), long reaction times and complex synthetic procedures with dangerous diazonium salts [17]. A more recent example of an ^18^F-labeled tracer that is thought to preferentially target the KYN pathway is 1-(2-[^18^F]fluoroethyl)-tryptophan ([^18^F]FETrp, Figure 1), which accumulates in IDO1-expressing cells and IDO1-positive tumor xenografts [18,19]. However, the radiosynthesis of [^18^F]FETrp is elaborate and requires separation of enantiomers by chiral preparative HPLC [19].

Being interested in easily accessible PET-tracers for imaging of Trp metabolism, we previously developed an efficient route for the preparation of 4–7-[^18^F]fluorotryptophans (4–7-[^18^F]FTrps) via alcohol-enhanced Cu-mediated radiofluorination and subjected them to a preclinical evaluation [20,21]. Whereas 4–6-[^18^F]FTrps suffered from rapid defluorination in vivo, 7-[^18^F]FTrp (Figure 1) was stable and showed preferential uptake in serotonergic brain regions and the melatonin-producing pineal gland of healthy rats. However, there is still an unmet need for PET-tracers that could be used to selectively address the different branches of Trp metabolism.

**Figure 2:**
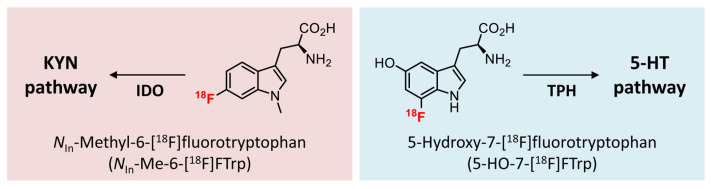
Candidate PET-tracers for pathway-specific imaging of tryptophan metabolism prepared in the present study.

The aim of the present work was to prepare *N*_In_-methyl-6-[^18^F]fluorotryptophan (*N*_In_-Me-6-[^18^F]FTrp) and 5-hydroxy-7-[^18^F]fluorotryptophan (5-HO-7-[^18^F]FTrp) as candidate PET-tracers for selective visualization of either the KYN pathway or serotonin synthesis, respectively (Figure 2). Based on the exceptionally broad substrate specificity of amino acid transporters like LAT1, which are highly expressed in the blood–brain-barrier and have been shown to accept a range of structurally diverse unnatural amino acids [22,23,24], both probes should be efficiently transported into the brain. Additionally, *N*_In_-Me-6-[^18^F]FTrp was chosen based on previous findings that both *N*_In_-methyltryptophan and 6-fluorotryptophan are substrates of IDO [18,25,26], suggesting that probes derived from these compounds could be used for visualization of the KYN pathway in the brain. Furthermore, *N*_In_-methyltryptophan has been shown to not be a substrate of AADC [27] or TDO [18,28], and the electron-withdrawing fluorine substituent in 6-position of the indole ring should destabilize the cationic transition state formed during 5-hydroxylation of tryptophans by TPH [29,30], suggesting that *N*_In_-Me-6-[^18^F]FTrp will neither enter the serotonin pathway nor be subject to significant peripheral metabolism via the KYN pathway in the liver. Conversely, 5-HO-7-[^18^F]FTrp was chosen based on the low affinity of 5-hydroxytryptophanes for IDO [18] and the fact that 7-fluorotryptophan is neither a good substrate of IDO nor of TDO [26,31]. Furthermore, 5-hydroxytryptophan is a known endogenous AADC substrate and aromatic fluorine substituents have been shown to be well tolerated by this enzyme [32,33,34,35,36], which suggests that this candidate could be used to target the second step in serotonin biosynthesis while minimizing metabolism via the KYN pathway. In addition to the preparation of the two radiolabeled amino acids, we describe the synthesis of the non-radioactive reference compounds and the corresponding radiolabeling precursors.

## 2. Results and Discussion

### 2.1. Chemistry

**Scheme 1:**
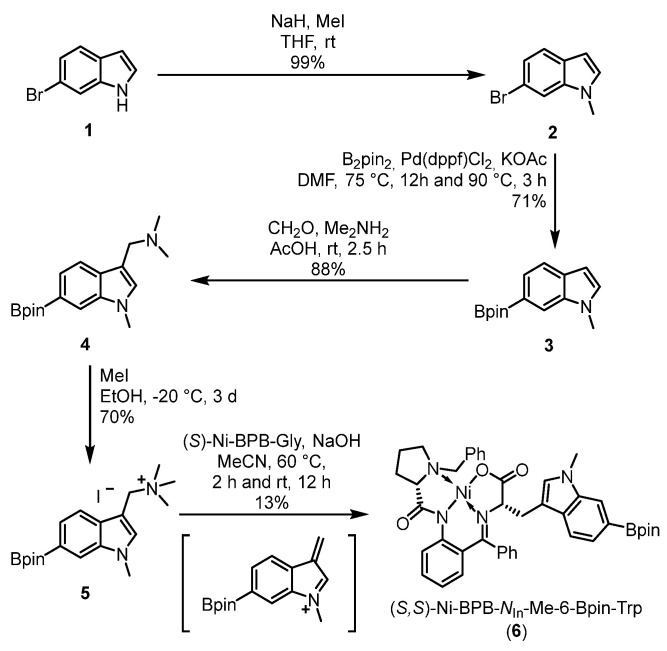
Synthesis of radiolabeling precursor **6**.

The synthesis of the radiolabeling precursor for *N*_In_-Me-6-[^18^F]FTrp was started from commercially available 6-bromoindole (**1**). First, **1** was methylated with iodomethane (MeI) using sodium hydride as a base, which afforded intermediate **2** [37] in a quantitative yield (Scheme 1). Next, **2** was borylated to give 6-Bpin-*N*-methylindole (**3**) [38]. The latter was introduced into a Mannich-type reaction [39], furnishing 6-Bpin-*N_In_*-methylgramine (**4**) in a yield of 88%. Subsequent addition of MeI at low temperature resulted in the quaternized gramine **5** as hydroiodide salt, which was used for stereoselective alkylation of an (*S*)-Ni-BPB-Gly complex [40]. In the latter reaction, the *N*-benzylproline moiety directed the diastereoselective Michael addition of deprotonated (*S*)-Ni-BPB-Gly to an 1-methyl-3-methylidene-3*H*-indolium species (generated in situ from **5** via elimination of trimethylamine followed by isomerization), resulting in selective formation of (*S*,*S*)-Ni-BPB-*N*_In_-Me-6-Bpin-Trp (**6**) [41]. The yield for this reaction step was fair (13%), and the product had to be purified via successive normal- and reversed-phase chromatography, so that precursor **6** was obtained in a total yield of 6% over 5 steps (Scheme 1). However, given that presence of the *N*_In_-methyl group hindered formation of the reactive intermediate, this was considered to be acceptable.

**Scheme 2:**
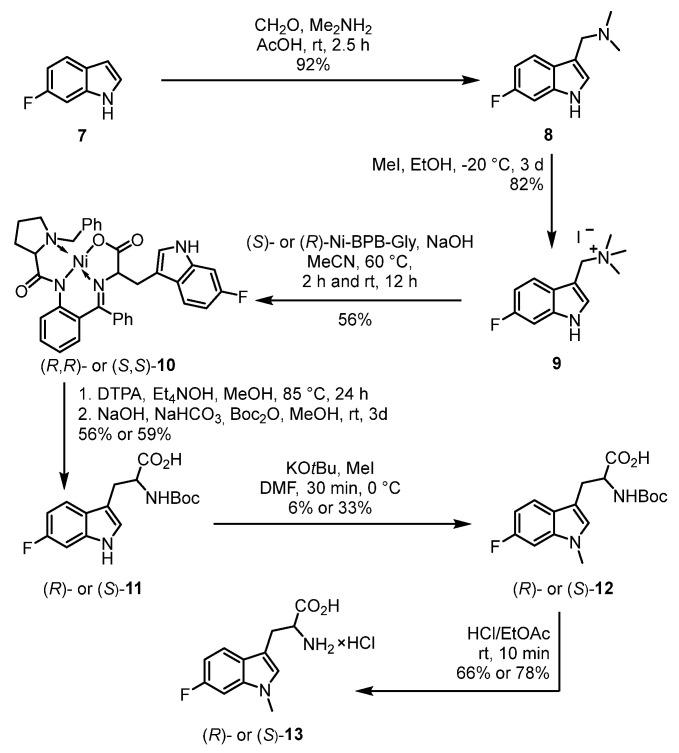
Synthesis of (*R*)- and (*S*)-*N*_In_-Me-6-FTrps [(*R*)- and (*S*)-**13**].

The initial steps for preparation of the reference compound *N*_In_-Me-6-FTrp were similar to those for preparation of **6** described above (Scheme 2). In particular, 6-fluoroindole (**7**) was subjected to a Mannich-type reaction (92% yield) followed by quaternization of the resulting intermediate (**8**) with MeI to furnish 1-(6-fluoro-1*H*-indol-3-yl)-*N*,*N*,*N*-trimethylmethanaminium iodide (**9**) in 82% yield. Initial attempts to utilize this quaternized gramine for Michael addition with an achiral Ni-BPA-Gly complex [42] were unsuccessful owing to an extremely low solubility of the complex in MeCN used as reaction solvent. Since direct production of racemic intermediates through this method was not feasible, we instead opted to synthesize (*R*)- and (*S*)-*N*_In_-Me-6-FTrp separately. To this end, either (*R*)- or (*S*)-Ni-BPB-Gly were alkylated with **9**, furnishing (*R*,*R*)- or (*S*,*S*)-**10** in a yield of 56%. These complexes were then decomposed with (Et_4_N)_2_DTPA [43], followed by in situ *N*_α_-Boc protection to give the respective protected tryptophans, (*R*)- and (*S*)-**11**, in 56% and 59% yield, respectively. Subsequent *N*_In_-methylation and deprotection of the resulting intermediates (*R*)- and (*S*)-**12** yielded the desired reference compounds (*R*)- and (*S*)-*N*_In_-Me-6-FTrp [(*R*)- and (*S*)-**13**] as hydrochloride salts in total yields of 1% and 6% over six steps, respectively (Scheme 2).

**Scheme 3:**
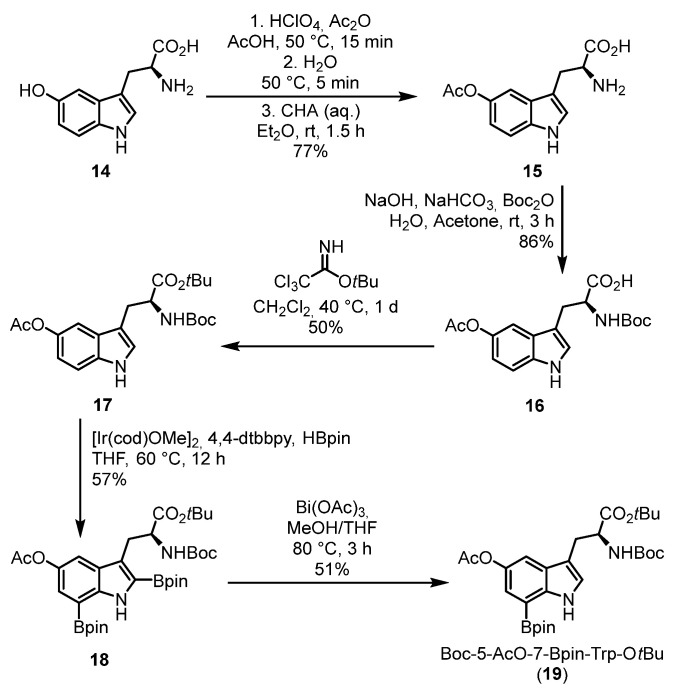
Synthesis of radiolabeling precursor **19**.

To access the radiolabeling precursor for 5-HO-7-[^18^F]FTrp, the hydroxy-, amino-, and carboxyl-groups in 5-hydroxytryptophan (**14**) were protected by successive introduction of an acetoxy-, a Boc-, and a *tert*-butyl-group, which afforded the corresponding intermediates in yields of 77% (**15**), 86% (**16**), and 50% (**17**), respectively (Scheme 3) [44,45,46]. The resulting Boc-5-AcO-Trp-O*t*Bu (**17**) was then 2,7-diborylated under Ir-catalysis [47] to obtain the diborylated tryptophan **18**. According to previous reports, diborylated tryptophans can usually be selectively 2-deborylated in situ using Pd(OAc)_2_/AcOH [21], but **18** proved to be completely stable towards Pd-mediated deborylation. Gratifyingly, if Bi(OAc)_3_ was used as deborylating agent [48], the desired radiolabeling precursor Boc-5-AcO-7-Bpin-Trp-O*t*Bu (**19**) was obtained in a yield of 51%, corresponding to a total yield of 9% over 5 steps (Scheme 3).

**Scheme 4:**
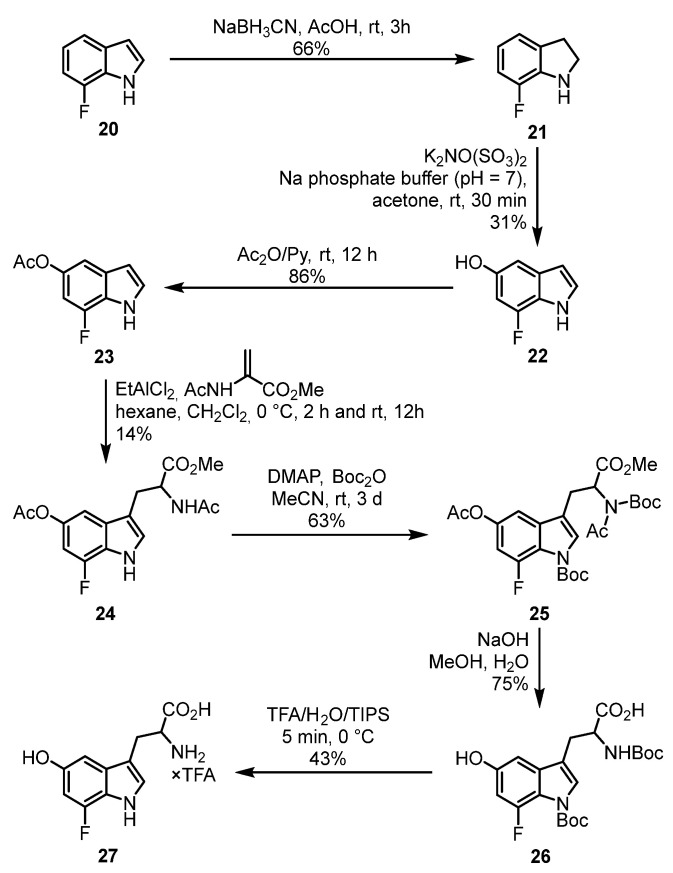
Synthesis of racemic 5-HO-7-FTrp (**27**).

Due to the difficulty of introducing a hydroxy group into the indole ring together with the low stability of substituted 5-hydroxyindoles and -tryptophans, synthesis of the reference compound 5-HO-7-FTrp proved to be particularly challenging (for details on various synthetic routes evaluated, see supporting information). However, we eventually obtained the desired product using a combination of oxidation via potassium nitrosodisulfate (Fremy’s Salt) [49] and Friedel–Crafts alkylation [50] (Scheme 4). To this end, 7-fluoroindole (**20**) was initially reduced to 7-fluoroindoline (**21**) using sodium cyanoborohydride in acetic acid (66% yield) as described by Lohray et al. [51]. Indoline **21** was then utilized as substrate for regioselective introduction of a hydroxy group in the 5-position of the indole ring using Fremy’s salt [49]. This step was performed in phosphate buffer at pH = 7 for 30 min, which provided the best conversions with minimal formation of side-products. The yield of isolated 5-hydroxy-7-fluoroindole (**22**) thus obtained amounted to 31%, and subsequent *O*-acylation of **22** [52] afforded intermediate **23** in a yield of 86%. Transformation of indole **23** to a tryptophan derivative was accomplished according to a publication by Angelini et al. [50], via the EtAlCl_2_-catalyzed Friedel–Crafts reaction with methyl 2-acetamidoacrylate. The latter furnished methyl 2-acetamido-3-(5-acetoxy-7-fluoro-1*H*-indol-3-yl)propanoate (**24**) in a yield of 14%, which was then Boc-protected at both nitrogen atoms to obtain **25** (63% yield). Subsequent two-step deprotection via the intermediate **26** afforded the reference compound racemic 5-HO-7-FTrp as TFA salt (**27** × TFA) in a total yield of 1% over 7 steps.

### 2.2. Radiosyntheses

According to a modified protocol for alcohol-enhanced Cu-mediated radiofluorination, [^18^F]fluoride ([^18^F]F^−^) was loaded onto a QMA carbonate anion exchange cartridge and eluted with Et_4_NHCO_3_ (5.2 µmol) in MeOH (0.5 mL), followed by MeOH evaporation [20,53,54]. Application of *n*BuOH instead of MeOH for [^18^F]F^−^ elution, as described in the original procedure, reduced the elution efficiency and radiochemical conversions (RCCs).

**Scheme 5:**
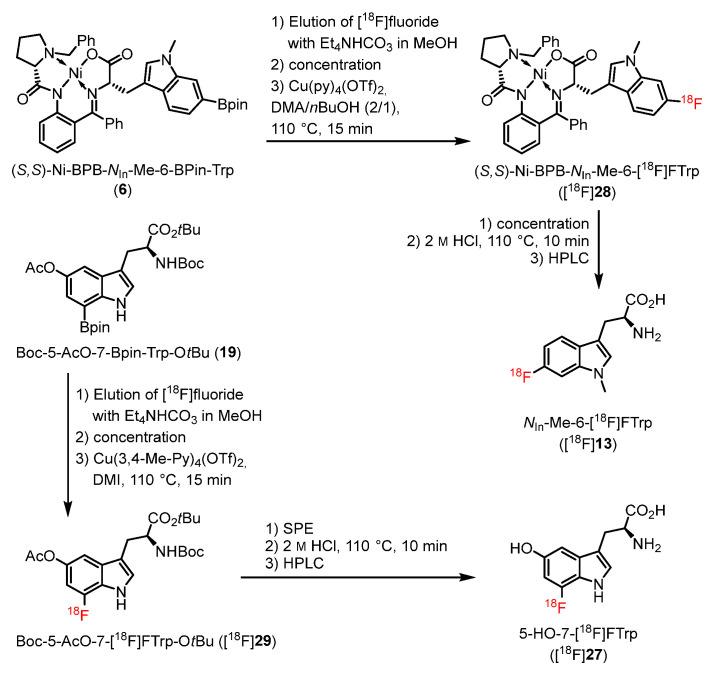
Radiosynthesis of *N*_In_-Me-6-[^18^F]FTrp ([^18^F]**13**) and 5-HO-7-[^18^F]FTrp ([^18^F]**27**) via Cu-mediated radiofluorination.

For radiosynthesis of *N*_In_-Me-6-[^18^F]FTrp ([^18^F]**13**), the approach laid out by Zlatopolskiy et al. was followed using the conventional Cu-mediator, Cu(Py)_4_(OTf)_2_, as described previously [21]. Briefly, after elution of [^18^F]F^−^ and removal of MeOH, a solution of boronate precursor **6** (10 µmol) and Cu(Py)_4_(OTf)_2_ (10 µmol) in a mixture of DMA/*n*BuOH (2/1, 0.75 mL) was added, and the resulting mixture was heated at 110 °C for 15 min under air. This furnished the radiolabeled intermediate [^18^F]**28** in RCCs of 75 ± 9%. After evaporation of the solvents, [^18^F]**28** was decomposed with 2 m HCl (0.3 mL) at 110 °C for 10 min [55], and the resulting tracer was isolated by HPLC to obtain a ready-to-use solution in 30% EtOH in sodium acetate buffer (for details see Section 3.2.2). This afforded *N*_In_-Me-6-[^18^F]FTrp ([^18^F]**13**) in RCYs of 45 ± 7% over two steps within 83 ± 8 min with a radiochemical purity (RCP) and enantiomeric excess (ee) of >99%, and molar activities (A_m_) of 66 ± 33 GBq/µmol (1–1.5 GBq tracer) (Scheme 5).

In contrast to **6**, alcohol-enhanced Cu-mediated radiofluorination of **19** with Cu(Py)_4_(OTf)_2_ as the mediator afforded radiolabeled intermediate [^18^F]**29** in a RCC of only 11%. In an attempt to improve the ^18^F-incorporation, we tested alternative Cu-mediators and reaction solvents for the labeling of **19**. Unexpectedly, neither application of Cu(4-Ph-Py)_4_(ClO_4_)_2_ nor of Cu(3,4-Me_2_-Py)_4_(OTf)_2_, which were identified as the most efficient radiofluorination mediators in our recent study [56], resulted in the formation of [^18^F]**29** if the reactions were performed in DMA/*n*BuOH. Replacement of DMA by 1,3-dimethyl-2-imidazolidinone (DMI) led to substantial improvements of the RCCs to 57% and 55% for Cu(4-Ph-Py)_4_(ClO_4_)_2_ and Cu(3,4-Me_2_-Py)_4_(OTf)_2_, respectively. However, under these reaction conditions, significant formation of unidentified radiolabeled side-products (up to 18%) was observed. Gratifyingly, no side-products were formed if the radiofluorinations were instead performed in pure DMI, while the RCCs were not significantly affected (50 ± 10% and 55 ± 7%, respectively). After further evaluation of alternative Cu-mediators and reaction solvents, we ultimately settled on the use of Cu(3,4-Me_2_-Py)_4_(OTf)_2_ and pure DMI. Thus, boronate precursor **19** and Cu(3,4-Me_2_-Py)_4_(OTf)_2_ (10 µmol of each) in DMI (0.75 mL) were added to Et_4_N[^18^F]F/Et_4_NHCO_3_, and the resulting solution was heated under air at 110 °C for 15 min. In this case, due to the significantly higher boiling point of DMI compared to DMA, the solvent had to be separated from intermediate [^18^F]**29** using solid-phase extraction (SPE). Following elution of [^18^F]**29** with MeOH (1 mL) and removal of MeOH under reduced pressure, 2 m HCl (0.3 mL) was added, and the intermediate was deprotected at 110 °C for 10 min. After subsequent HPLC purification, the final tracer was obtained as a ready-to-use solution in 8% EtOH in sodium acetate buffer (for details see Section 3.2.2). Using this protocol, 5-HO-7-[^18^F]FTrp ([^18^F]**27**) was obtained in RCYs of 29 ± 4% within 81 ± 3 min, with an RCP and ee of >99%, and an A_m_ of 46 ± 17 GBq/µmol (1.05–1.35 GBq tracer) (Scheme 5).

## 3. Materials and Methods

### 3.1. Chemistry

#### 3.1.1. General

Unless noted otherwise, all chemicals and solvents were purchased from VWR International (Langenfeld, Germany), Sigma-Aldrich (Steinheim, Germany), ChemPUR (Karlsruhe, Germany), ABCR GmbH (Karlsruhe, Germany), or Fluka AG (Buchs, Switzerland) and used without further purification. NMR spectra were measured at ambient temperature in deuterium oxide (D_2_O), deuterochloroform (CDCl_3_), deuteromethanol (CD_3_OD) or deuterodimethylsulfoxide [(CD_3_)_2_SO] as indicated. ^1^H-NMR spectra were acquired using a Bruker Avance II 300 (300 MHz; Bruker Daltonik GmbH, Bremen, Germany), a Bruker Avance 200 (200 MHz; Bruker Daltonik GmbH, Bremen, Germany), or a Varian INOVA 400 (400 MHz; Varian, Darmstadt, Germany) spectrometer. ^13^C-NMR spectra [additional APT (Attached Proton Test)] were acquired using a Bruker DPX Avance 200 (50 MHz; Bruker Daltonik GmbH, Bremen, Germany), a Bruker Avance II 300 (75 MHz) or a Varian INOVA 400 (101 MHz) spectrometer. ^19^F-NMR spectra were acquired using a Varian INOVA 400 (376 MHz) spectrometer. The measured chemical shifts (*δ*) are reported in parts per million (ppm) relative to residual peaks of non-deuterated solvents. The observed signal multiplicities are characterized as follows: s = singlet, d = doublet, t = triplet, q = quartet, p = pentet, m = multiplet, dd = doublet of doublets, dt = doublet of triplets, dq = doublet of quartets, ddd = doublet of doublets of doublets, ddq = doublet of doublets of quartets, dtd = doublet of triplets of doublets, dtq = doublet of triplets of quartets, td = triplet of doublets and qd = quartet of doublets. Coupling constants *J* are reported in Hertz (Hz). High resolution mass spectra were measured with a FTICR “LTQ FT Ultra” (Thermo Fisher Scientific Inc., Bremen, Germany). Manual column chromatography was performed with silica gel, 60 Å, 230–400 mesh particle size from Merck KGaA (Darmstadt, Germany) or silica gel (w/Ca, 0.1%), 60 Å, 230–400 mesh particle size from Sigma-Aldrich GmbH (Steinheim, Germany). Automated column chromatography was either performed on a Grace Reveleris X1 (Grace Reveleris, Columbia, MD, USA) or on a Büchi Pure C-815 Flash system (Büchi Labortechnik, Essen, Germany), using Si60 FlashPure cartridges or Reveleris™ C_18_ reversed phase cartridges, respectively. Thin-layer chromatography (TLC) was performed using aluminum sheets coated with silica gel 0.25 mm SIL G/UV 254 (Merck KGaA, Darmstadt, Germany). Chromatograms were inspected under UV light (λ = 254 nm) and/or stained with phosphomolybdic acid (4% in EtOH). If not stated otherwise, all reactions were carried out with magnetic stirring. Organic extracts were dried over anhydrous MgSO_4_. Air- or moisture-sensitive reagents were handled under argon (>99.999%, Air Liquide GmbH, Düsseldorf, Germany). Solutions were concentrated under reduced pressure (1–900 mbar) at 40–50 °C using a rotary evaporator (Büchi Labortechnik, Essen, Germany). Solvent proportions are indicated in a volume/volume ratio.

#### 3.1.2. 6-Bromo-1-methyl-1*H*-indole (**2**) [57]

NaH in mineral oil (0.6 g, 15.3 mmol, 1.2 eq, 60 wt% in oil) was added in portions over 15 min to an ice-cold solution of 6-bromoindole (2.5 g, 12.8 mmol, 1 eq) in anhydrous THF (26 mL), and the reaction mixture was stirred for 20 min. Iodomethane (2.27 g, 1.0 mL, 16.0 mmol, 1.25 eq) was then added and the mixture was stirred for an additional 20 min. The reaction mixture was concentrated under reduced pressure and the residue was taken up in saturated NaHCO_3_ (25 mL). The resulting emulsion was extracted with CH_2_Cl_2_ (3 × 20 mL) and the combined organic phases were dried, filtered through a pad of silica gel and concentrated under reduced pressure to obtain **2** as a brown oil (2.68 g, 12.8 mmol, 99%). ^1^H-NMR (300 MHz, CDCl_3_) δ ppm: 7.64–7.45 (m, 2H), 7.33–7.14 (m, 1H), 7.05 (d, *J* = 3.1 Hz, 1H), 6.49 (dd, *J* = 3.1, 0.9 Hz, 1H), 3.78 (s, 3H); ^13^C-NMR (75 MHz, CDCl_3_) δ ppm: 137.52 (C), 129.45 (CH), 127.29 (C), 122.52 (CH), 122.06 (CH), 115.13 (C-Br), 112.28 (CH), 101.21 (CH), 32.91 (CH_3_); MS (ESI) *m*/*z* [M+H]^+^ calcd. for C_9_H_8_BrN^+^ 210.07, found 210.20; Elemental analysis: Calcd. for C_9_H_7_BrN: C, 51.46; H, 3.84; N, 6.67. Found: C, 51.7 ± 0.3; H, 3.83 ± 0.06; N, 6.64 ± 0.04.

#### 3.1.3. 1-Methyl-6-(4,4,5,5-tetramethyl-1,3,2-dioxaborolan-2-yl)-1*H*-indole (**3**)

A solution of **2** (4 g, 19 mmol, 1 eq), bis(pinacolato)diboron (5.79 g, 22.8 mol, 1.2 eq), Pd(dppf)Cl_2_ (1.39 g, 1.9 mmol, 0.1 eq) and KOAc (3.9 g, 40 mmol, 2.1 eq) in anhydrous DMF (100 mL) was stirred at 75 °C overnight and at 90 °C for 3 h [37]. The solvent was removed under reduced pressure and the residue was taken up in Et_2_O (200 mL). The solution was washed with H_2_O (3 × 200 mL) and brine (3 × 200 mL), dried and concentrated under reduced pressure. The residue was purified via column chromatography [silica gel (0.1% Ca), hexane:EtOAc/4:1] to obtain **3** as a colorless oil (3.5 g, 13.6 mmol, 71%). R_f_ = 0.35 (silica gel, CHCl_3_:acetone/10:1); ^1^H-NMR (400 MHz, CDCl_3_) δ ppm: 7.88 (q, *J* = 0.9 Hz, 1H), 7.67 (dd, *J* = 8.0, 0.8 Hz, 1H), 7.62–7.58 (m, 1H), 7.13 (d, *J* = 3.0 Hz, 1H), 6.51 (dd, *J* = 3.0, 0.9 Hz, 1H), 3.86 (s, 3H), 1.42 (s, 12H), 1.30 (s, 3H); ^13^C-NMR (101 MHz, CDCl_3_) δ ppm: 136.45 (C), 131.07 (C), 130.20 (CH), 125.18 (CH), 120.17 (CH), 116.20 (CH), 100.99 (CH), 83.55 (C × 2), 32.98 (CH_3_), 24.93 (CH_3_ × 4), C-B was not observed; HRMS (ESI) *m*/*z* [M+H]^+^ calcd. for C_15_H_21_BNO_2_^+^ 258.16599, found 258.16632.

#### 3.1.4. *N*,*N*-Dimethyl-1-[1-methyl-6-(4,4,5,5-tetramethyl-1,3,2-dioxaborolan-2-yl)-1*H*-indol-3-yl]methanamine (**4**)

37% Formaldehyde (1.27 mL, 1.38 g, 17 mmol, 1.25 eq) and 40% Me_2_NH (2.99 mL, 3.36 g, 23.8 mmol, 1.75 eq) were added to an ice-cold solution of **3** (3.5 g, 13.6 mmol, 1 eq) in AcOH (15 mL) [39]. The reaction mixture was stirred at ambient temperature for 2.5 h, cooled to 0 °C and basified to pH = 10 with 3 m NaOH. The mixture was extracted with EtOAc (4 × 75 mL) and the combined organic phases were dried and concentrated under reduced pressure to obtain **4** (3.77 g, 12 mmol, 88%) as a yellow solid. ^1^H-NMR (200 MHz, CDCl_3_) δ ppm: 7.84 (q, *J* = 0.8 Hz, 1H), 7.72–7.55 (m, 2H), 7.13 (s, 1H), 3.83 (d, *J* = 0.7 Hz, 3H), 3.70 (s, 2H), 2.32 (d, *J* = 0.7 Hz, 6H), 1.39 (d, *J* = 0.7 Hz, 12H), 1.25 (d, *J* = 0.6 Hz, 2H); ^13^C-NMR (50 MHz, CDCl_3_) δ ppm: δ 136.65 (C), 130.89 (C), 130.38 (CH), 125.11 (CH), 118.48 (CH), 116.32 (CH), 110.75 (C), 83.59 (C), 53.96 (CH_2_), 44.88 (CH_3_ × 2), 32.94 (CH_3_), 24.93 (CH_3_ × 4), C-B was not observed; HRMS (ESI) *m*/*z* [M+H]^+^ calcd. for C_18_H_28_BN_2_O_2_^+^ 315.22384, found 315.22430.

#### 3.1.5. *N*,*N*,*N*-Trimethyl-1-[1-methyl-6-(4,4,5,5-tetramethyl-1,3,2-dioxaborolan-2-yl)-1*H*-indol-3-yl]methanaminium Iodide (**5**)

Iodomethane (8.5 g, 3.72 mL, 60 mmol, 5 eq) was added to an ice-cold solution of **4** (3.77 g, 12 mmol, 1 eq) in anhydrous EtOH (30 mL), and the reaction mixture was left in the fridge at −20 °C for 3 d. The precipitate was filtered off and washed with cold Et_2_O to obtain **5** (3.78 g, 8.3 mmol, 70%) as a colorless solid. ^1^H-NMR [400 MHz, (CD_3_)_2_SO] δ ppm: 7.96–7.74 (m, 3H), 7.49 (dd, *J* = 8.0, 0.9 Hz, 1H), 4.69 (s, 2H), 3.91 (s, 3H), 3.34 (s, 3H), 3.04 (s, 9H), 2.51 (p, *J* = 1.8 Hz, 3H), 1.32 (s, 12H); ^13^C-NMR [75 MHz, (CD_3_)_2_SO] δ ppm: 141.45 (C), 140.80 (CH), 135.91 (C), 131.06 (CH), 123.44 (CH), 122.20 (CH), 106.17 (C), 88.71 (C × 2), 65.36 (CH_2_), 56.36 (CH_3_ × 3), 38.18 (CH_3_), 29.94 (CH_3_ × 4), C-B was not observed; HRMS (ESI) *m*/*z* [M−I]^+^ calcd. for C_19_H_30_BN_2_O_2_^+^ 329.23949, found 329.23984.

#### 3.1.6. (*S*,*S*)-Ni-BPB-*N*_In_-methyl-6-Bpin-tryptophan (**6**)

Powdered NaOH (0.5 g, 12.5 mmol, 2.5 eq) was added to a solution of **5** (2.3 g, 5 mmol, 1 eq) and (*S*)-[Ni(II)-BPB-Gly] (2.56 g, 5 mmol, 1 eq) in anhydrous MeCN (125 mL), and the resulting suspension was stirred at 60 °C for 2 h and at ambient temperature overnight. Saturated NH_4_Cl (300 mL) was added, and the resulting emulsion was extracted with EtOAc (3 × 300 mL). The combined organic fractions were dried and concentrated under reduced pressure. The residue was purified via NP column chromatography (silica gel, CH_2_Cl_2_:acetone/2:1) followed by RP chromatography (Chromabond C_18_, H_2_O:MeCN, gradient) to obtain **6** as a red solid (0.5 g, 0.65 mmol, 13%). R_f_ = 0.35 (silica gel, CH_2_Cl_2_:aceton/10:1); ^1^H-NMR (400 MHz, CDCl_3_) δ ppm: 8.24 (d, *J* = 8.7 Hz, 1H), 7.97 (d, *J* = 7.5 Hz, 2H), 7.84 (d, *J* = 17.5 Hz, 1H), 7.69–7.06 (m, 8H), 7.01–6.86 (m, 1H), 6.69 (d, *J* = 4.3 Hz, 1H), 4.43–4.14 (m, 1H), 3.80 (d, *J* = 24.7 Hz, 3H), 3.49–3.02 (m, 3H), 2.91–2.66 (m, 1H), 2.25–2.04 (m, 1H), 1.96 (dq, *J* = 15.9, 7.6, 6.7 Hz, 1H), 1.76 (dtd, *J* = 32.1, 11.6, 10.9, 6.3 Hz, 1H), 1.38 (s, 12H), (contains trace amounts of boronic acid derivative after RP chromatography); ^13^C-NMR (101 MHz, CDCl_3_) δ ppm: 180.14 (C), 179.41 (C), 170.84 (C), 142.84 (C), 136.94 (C), 134.78 (CH), 134.14 (C), 133.46 (CH), 133.16 (C), 132.29 (CH), 131.55 (CH), 131.22 (C), 130.29 (CH), 129.65 (CH), 129.04 (CH), 128.79 (CH), 128.72 (CH), 128.21 (CH), 128.03 (CH), 127.69 (CH), 127.33 (CH), 126.23 (C), 125.54 (CH), 123.50 (CH), 120.52 (CH), 118.93 (CH), 117.69 (CH), 116.14 (CH), 108.62 (C), 83.57 (C × 2), 70.26 (CH), 63.07 (CH), 56.90 (CH_2_), 32.94 (CH_3_), 30.62 (CH_2_), 30.27 (CH_2_), 24.87 (CH_3_ × 4), 22.66 (CH_2_), C-B was not observed (contains trace amounts of boronic acid derivative after RP chromatography); HRMS (ESI) *m*/*z* [M+H]^+^ calcd. for C_43_H_46_BN_4_NiO_5_^+^ 767.29093, found 767.29184.

#### 3.1.7. 1-(6-Fluoro-1*H*-indol-3-yl)-*N*,*N*-dimethylmethanamine (**8**)

37% Formaldehyde (1.38 mL, 1.50 g, 18.48 mmol, 1.25 eq) and 40% Me_2_NH (2.89 g, 2.48 mL, 24.87 mmol, 1.75 eq) were added to an ice-cold solution of 6-fluoroindole (2.0 g, 14.78 mmol, 1 eq) in AcOH (15 mL). The reaction mixture was stirred at ambient temperature for 2.5 h, cooled in an ice-water bath, and basified to pH = 10 with 3 m NaOH. The mixture was then extracted with EtOAc (4 × 40 mL), and the combined organic phases were dried and concentrated under reduced pressure to obtain **8** (2.61 g, 13.5 mmol, 92%) as an off-white solid. ^1^H-NMR [400 MHz, (CD_3_)_2_SO] δ ppm: 7.58 (dd, *J* = 8.7, 5.6 Hz, 1H), 7.21 (d, *J* = 2.3 Hz, 1H), 7.12 (dd, *J* = 10.2, 2.4 Hz, 1H), 6.83 (ddd, *J* = 9.9, 8.6, 2.4 Hz, 1H), 3.50 (d, *J* = 0.8 Hz, 2H), 2.14 (s, 6H); ^13^C-NMR [101 MHz, (CD_3_)_2_SO] δ ppm: 159.27 (d, *J* = 233.6 Hz, C-F), 136.62 (d, *J* = 12.8 Hz, C), 125.31 (d, *J* = 3.2 Hz, CH), 124.79 (C), 120.55 (d, *J* = 10.2 Hz, CH), 112.48 (C), 107.22 (d, *J* = 24.4 Hz, CH), 97.69 (d, *J* = 25.4 Hz, CH), 54.88 (CH_2_), 45.38 (CH_3_ × 2); ^19^F-NMR [376 MHz, (CD_3_)_2_SO] δ ppm: −122.46; HRMS (ESI) *m*/*z* [M+H]^+^ calcd. for C_11_H_14_FN_2_^+^ 193.11410, found 193.11367.

#### 3.1.8. 1-(6-Fluoro-1*H*-indol-3-yl)-*N*,*N*,*N*-trimethylmethanaminium Iodide (**9**)

Iodomethane (9.6 g, 4.2 mL, 67.5 mmol, 5 eq) was added to an ice-cold solution of **8** (2.61 g, 13.5 mmol, 1 eq) in anhydrous EtOH (30 mL) and the reaction mixture was left in the fridge at −20 °C for 12 h. The mixture was then concentrated under reduced pressure and the residue was sonicated and triturated with EtOAc until a precipitate formed. The precipitate was filtered off, rinsed with Et_2_O and dried to obtain **9** (3.7 g, 11.1 mmol, 82%) as a colorless solid. ^1^H-NMR [400 MHz, (CD_3_)_2_SO] δ ppm: 7.98–7.60 (m, 2H), 7.36–6.90 (m, 2H), 4.76 (d, *J* = 47.7 Hz, 2H), 3.09 (d, *J* = 32.5 Hz, 9H); ^13^C-NMR [101 MHz, (CD_3_)_2_SO] δ ppm: 159.51 (d, *J* = 235.9 Hz, C-F), 136.47 (d, *J* = 11.8 Hz, C), 131.40 (CH), 124.99 (C), 121.54–117.99 (m, CH), 109.17 (d, *J* = 24.4 Hz, CH), 102.55 (C), 98.56 (d, *J* = 25.7 Hz, CH), 60.81 (CH_2_), 51.72 (CH_3_ × 3); ^19^F-NMR [376 MHz, (CD_3_)_2_SO] δ ppm: −121.12; HRMS (ESI) *m*/*z* [M−I]^+^ calcd. for C_12_H_16_FN_2_^+^ 207.12920, found 207.12930.

#### 3.1.9. (*R*,*R*)- or (*S*,*S*)-Ni(II)-BPB-6-fluoro-tryptophan [(*R*,*R*)- or (*S*,*S*)-**10**] [40]

(*R*,*R*)-**10**: Powdered NaOH (0.3 g, 7.5 mmol, 2.5 eq) was added to a solution of **9** (1.0 g, 3 mmol, 1 eq) and (*R*)-[Ni(II)-BPB-Gly] (1.5 g, 3 mmol, 1 eq) in anhydrous MeCN (80 mL), and the resulting suspension was stirred under argon at 60 °C for 3 h and at ambient temperature overnight. Saturated NH_4_Cl (200 mL) was then added, and the resulting emulsion was extracted with EtOAc (3 × 100 mL). The combined organic phases were dried and concentrated under reduced pressure. The residue was purified via column chromatography (silica gel, CHCl_3_:acetone/30:1) to obtain (*R*,*R*)-**10** as a red solid (1.12 g, 1.7 mmol, 56%);

(*S*,*S*)-**10**: This compound (red solid; 1.12 g, 1.7 mmol, 56%) was prepared according to the procedure described for (*R*,*R*)-**10** using **9** (1.0 g, 3 mmol, 1 eq), (*S*)-[Ni(II)-BPB-Gly] (1.5 g, 3 mmol, 1 eq), powdered NaOH (0.3 g, 7.5 mmol, 2.5 eq), and anhydrous MeCN (80 mL).

Spectroscopic/analytical data were identical for both isomers. Only data for (*R*,*R*)-**10** are reported. ^1^H-NMR (400 MHz, CDCl_3_) δ ppm: 8.24 (d, *J* = 8.7 Hz, 1H), 8.12–7.90 (m, 2H), 7.65–7.48 (m, 2H), 7.40–7.27 (m, 4H), 7.26–7.11 (m, 3H), 7.04 (dd, *J* = 9.6, 2.3 Hz, 1H), 6.94–6.81 (m, 2H), 6.81–6.61 (m, 3H), 4.35–4.12 (m, 2H), 3.56–3.40 (m, 2H), 3.29 (ddd, *J* = 27.7, 12.4, 5.7 Hz, 2H), 3.09 (dd, *J* = 14.7, 5.8 Hz, 1H), 2.87 (ddd, *J* = 10.4, 6.3, 3.4 Hz, 1H), 2.28–1.71 (m, 5H), 1.50 (dtq, *J* = 12.1, 8.4, 4.9, 4.2 Hz, 1H), 1.33–1.12 (m, 1H); ^13^C-NMR (101 MHz, CDCl_3_) δ ppm: 180.20 (C), 179.52 (C), 170.85 (C), 160.06 (d, *J* = 237.6 Hz, C-F), 142.68 (C), 136.45 (d, *J* = 12.5 Hz, C), 134.03 (C), 133.55 (CH), 133.25 (C), 132.36 (CH), 131.50 (CH), 129.76 (CH), 129.11 (CH), 128.78 (CH), 127.93 (CH), 127.30 (CH), 126.22 (C), 124.81 (C) 123.47 (CH), 120.69 (CH), 109.63 (CH), 108.51 (d, *J* = 24.6 Hz, CH), 97.48 (d, *J* = 26.0 Hz, CH), 71.32 (CH), 70.41 (CH), 63.31 (CH_2_), 57.02 (CH_2_), 30.76 (CH_2_), 30.51 (CH_2_), 22.81 (CH_2_); ^19^F-NMR (376 MHz, CDCl_3_) δ ppm: −121.32; HRMS (ESI) *m*/*z* [M+H]^+^ calcd. for C_36_H_32_O_3_N_4_FNi^+^ 645.18119, found 645.18112.

#### 3.1.10. (*R*)- or (*S*)-2-[(*tert*-Butoxycarbonyl)amino]-3-(6-fluoro-1*H*-indol-3-yl)propanoic Acid [(*R*)- or (*S*)-**11**]

(*R*)-**11**: A solution of DTPA (4.13 g, 10.5 mmol) in 25% Et_4_NOH (12.1 mL) was added to a solution of (*R*,*R*)-**10** (0.62 g, 1 mmol, 1 eq) in MeOH (30 mL), and the deeply red-colored mixture was stirred at 85 °C for 24 h. The resulting faintly green colored solution was cooled to ambient temperature and MeOH was removed under reduced pressure. The remaining aqueous solution was cooled to 0 °C, and the precipitated (*R*)-BPB was filtered off and washed with cold H_2_O. The filtrate was basified to pH = 9.0–9.5 with 1 m NaOH and washed with Et_2_O (3 × 20 mL). Saturated NaHCO_3_ (4.5 mL), followed by Boc_2_O (0.66 g, 3 mmol, 3 eq) and MeOH, was added to the aqueous solution until the mixture was homogeneous. The mixture was stirred for 3 d at ambient temperature, and MeOH was removed under reduced pressure. The remaining aqueous phase was washed with Et_2_O (3 × 20 mL), acidified to pH = 2 with solid NaHSO_4_, and extracted with Et_2_O (2 × 30 mL). The organic phase was washed with 1 m NaHSO_4_ (3×), H_2_O (3×), and brine (2×); dried; and concentrated under reduced pressure to obtain (*R*)-**11** as a colorless solid (0.18 g, 0.56 mmol, 56%).

(*S*)-**11**: This compound (colorless solid; 0.17 g, 0.53 mmol, 59%) was prepared from (*S*,*S*)-**10** using the procedure described for (*R*)-**11**.

Spectroscopic/analytical data were identical for both isomers. Only data for (*R*)-**11** are reported. NMR spectra showed the presence of two rotamers. Only data for the major rotamer are reported. ^1^H-NMR [400 MHz, (CD_3_)_2_SO] δ ppm: 7.51 (dd, *J* = 8.7, 5.4 Hz, 1H), 7.22–7.06 (m, 2H), 7.00 (d, *J* = 8.1 Hz, 1H), 6.90–6.78 (m, 1H), 4.23–4.06 (m, 1H), 3.18–3.07 (m, 1H), 2.97 (dd, *J* = 14.6, 9.3 Hz, 1H), 1.33 (s, 9H); ^13^C-NMR [101 MHz, (CD_3_)_2_SO] δ ppm: 174.33 (C), 159.29 (d, *J* = 233.6 Hz, C-F), 155.86 (C), 136.42 (C), 124.70 (CH), 124.56 (C), 119.59 (CH), 110.99 (C), 107.26 (d, *J* = 24.3 Hz, CH), 97.78 (d, *J* = 25.4 Hz, CH), 78.49 (C), 54.96 (CH), 28.63 (CH_3_ × 3), 27.79 (CH_2_); ^19^F-NMR [376 MHz, (CD_3_)_2_SO] δ ppm: −122.52; Elemental analysis: Calcd. for C_16_H_19_FN_2_O_4_: C, 59.62; H, 5.94; N, 8.69. Found: C, 59.4 ± 0.1; H, 6.02 ± 0.05; N, 8.35 ± 0.02.

#### 3.1.11. (*R*)- or (*S*)-2-[(*tert*-Butoxycarbonyl)amino]-3-(6-fluoro-1-methyl-1*H*-indol-3-yl)propanoic Acid [(*R*)- or (*S*)-**12**]

(*R*)-**12**: KO*t*Bu (1 m in *t*BuOH, 1.6 g, 1.8 mL, 2 eq) was added to an ice-cold solution of (*R*)-**11** (0.29 g, 0.9 mmol, 1 eq) in anhydrous DMF (10 mL) and the reaction mixture was stirred for 15 min. Iodomethane (0.19 g, 0.084 mL, 1.35 mmol, 1.5 eq) was then added, and the mixture was stirred for another 15 min, quenched with 1 m NaHSO_4_, and extracted with EtOAc. The organic phase was washed with 1 m NaHSO_4_, dried, and concentrated under reduced pressure. The residue was purified via column chromatography (silica gel, hexane:EtOAc/2:1, 3% AcOH) to obtain (*R*)-**12** (17 mg, 0.05 mmol, 6%) as a colorless foam.

(*S*)-**12**: This compound (colorless foam; 96 mg, 0.3 mmol, 33%) was prepared from (*S*)-**11** using the procedure described for (*R*)-**12**.

Spectroscopic/analytical data was identical for both isomers. Only data for (*S*)-**12** are reported. R_f_ = 0.34 [silica gel, hexane:EtOAc/2:1 (3% AcOH)]; ^1^H-NMR (400 MHz, CDCl_3_) δ ppm: 7.51 (dd, *J* = 8.7, 5.2 Hz, 1H), 7.28 (s, 0H), 6.96 (dd, *J* = 9.8, 2.3 Hz, 1H), 6.91–6.81 (m, 2H), 5.09 (d, *J* = 8.1 Hz, 1H), 4.66 (q, *J* = 6.2 Hz, 1H), 3.66 (d, *J* = 15.9 Hz, 3H), 3.30 (qd, *J* = 14.7, 5.5 Hz, 2H), 1.45 (s, 6H), 1.25 (s, 3H); ^13^C-NMR (101 MHz, CDCl_3_) δ ppm: 176.49 (C), 159.99 (d, *J* = 237.8 Hz, C-F), 155.54 (C), 136.98 (d, *J* = 11.9 Hz, C), 128.39 (CH), 127.93 (CH), 124.76 (C), 119.74 (d, *J* = 10.0 Hz, CH), 108.78 (C), 107.89 (d, *J* = 24.9 Hz, CH), 95.66 (d, *J* = 26.1 Hz, CH), 80.26 (C), 54.14 (CH), 32.78 (CH_3_), 28.32 (CH_3_ × 3), 27.89 (CH_2_); ^19^F-NMR (376 MHz, CDCl_3_) δ ppm: −120.83; Elemental analysis: Calcd. for C_17_H_21_FN_2_O_4_: 60.70; H, 6.29; N, 8.33. Found: C, 60.3 ± 0.3; H, 6.3 ± 0.02; N, 8.12 ± 0.04.

#### 3.1.12. (*R*)- or (*S*)-2-Amino-3-(6-fluoro-1-methyl-1*H*-indol-3-yl)propanoic Acid Hydrochloride [(*R*)- or (*S*)-**13** × HCl]

4.8 m HCl in EtOAc [58] was prepared by adding acetyl chloride (2.84 mL, 3.12 g, 40 mmol, 0.97 eq) to an ice-cold solution of MeOH (1.66 mL, 1.31g, 41 mmol, 1 eq) in EtOAc (8.34 mL). The resulting mixture was incubated for 10 min at 0 °C and for 20 min at ambient temperature.

(*R*)-**13** × HCl: A solution of (*R*)-**12** (20 mg, 0.06 mmol) in the freshly prepared 4.8 m HCl in EtOAc (0.6 mL) was incubated for 10 min at ambient temperature and then centrifuged for 10 min at 9000 rpm. The supernatant was discarded, and the precipitate was successively washed with EtOAc (2 × 1 mL), Et_2_O (1 mL), and pentane (1 mL). The residue was dried at 40 °C and 4 mbar for 4 h to obtain (*R*)-**13** × HCl (11 mg, 0.04 mmol, 66%) as a colorless solid.

(*S*)-**13** × HCl: This compound (colorless solid, 13 mg, 0.047 mmol, 78%) was prepared from (*S*)-**12** (20 mg, 0.06 mmol) using the procedure described for (*R*)-**13** × HCl.

Spectroscopic/analytical data were identical for both isomers. Only data for (*S*)-**13** × HCl are reported. ^1^H-NMR [400 MHz, (CD_3_)_2_SO] δ ppm: 8.46 (s, 2H), 7.61 (dd, *J* = 8.7, 5.4 Hz, 1H), 7.30 (dd, *J* = 10.4, 2.3 Hz, 1H), 7.24 (s, 1H), 6.90 (ddd, *J* = 9.8, 8.7, 2.4 Hz, 1H), 4.06 (d, *J* = 28.0 Hz, 1H), 3.72 (s, 3H), 3.29 (d, *J* = 6.0 Hz, 2H), 2.51 (p, *J* = 1.8 Hz, 2H); ^13^C-NMR [101 MHz, (CD_3_)_2_SO] δ ppm: 171.12 (C), 159.56 (d, *J* = 234.6 Hz, C-F), 137.20 (d, *J* = 12.4 Hz, C), 130.43 (d, *J* = 3.4 Hz, C), 124.62 (CH), 120.22 (d, *J* = 10.3 Hz, CH), 107.56 (d, *J* = 24.6 Hz, CH), 106.96 (C), 96.59 (d, *J* = 26.1 Hz, CH), 52.97 (CH), 33.08 (CH_3_), 26.18 (CH_2_); ^19^F-NMR [376 MHz, (CD_3_)_2_SO] δ ppm: −121.37; HRMS (ESI) *m*/*z* [M+H]^+^ calcd. for C_12_H_14_FN_2_O_2_^+^ 237.10338, found 237.10363.

#### 3.1.13. (*S*)-3-(5-Acetoxy-1*H*-indol-3-yl)-2-aminopropanoic Acid (**15**) [44]

A solution of 20% Ac_2_O in glacial AcOH (*w*/*v*, 121.5 mL; prepared from 22.5 mL, 24.3 g, 44 mmol Ac_2_O) was slowly added to a solution of 5-hydroxytryptophan (5.0 g, 22.5 mmol, 1 eq) in 0.1 m HClO_4_ in glacial AcOH (225 mL), and the reaction mixture was stirred at 50 °C for 15 min. H_2_O (4.6 mL) was added, and the mixture was stirred at 50 °C for another 5 min. After cooling to ambient temperature, the reaction mixture was placed in a water–ice bath, and 40% aqueous cyclohexylamine (*w*/*v*, 9 mL; prepared from 4.1 mL, 3.6 g, 36.3 mmol cyclohexylamine) in Et_2_O (225 mL) was added slowly. The cooling bath was removed, and the mixture was stirred at ambient temperature for 1.5 h. The precipitate was filtered off and rinsed with Et_2_O:AcOH (1:1, 35 mL) and Et_2_O (10 mL). The crude product was then suspended in EtOH:H_2_O (1:1, 25 mL), and the suspension was refluxed for 5 min, stirred at ambient temperature for 1 h, and stored at 5 °C overnight. The solid was filtered off, rinsed with 50% EtOH (3 × 10 mL), and dried under reduced pressure to obtain **15** × AcOH × H_2_O (5.9 g, 17.34 mmol, 77%) as a colorless solid. ^1^H-NMR [400 MHz, (CD_3_)_2_SO] δ ppm: 7.49–7.21 (m, 3H), 6.82 (dd, *J* = 8.6, 2.3 Hz, 1H), 3.64–3.43 (m, 1H), 3.28 (dd, *J* = 15.1, 4.3 Hz, 1H), 3.12–2.95 (m, 1H), 2.25 (s, 3H), 1.92 (s, 3H); ^13^C-NMR [101 MHz, (CD_3_)_2_SO] δ ppm: 172.70 (C), 171.32 (C), 170.43 (C), 143.83 (C), 134.60 (C), 127.83 (C), 126.37 (CH), 115.79 (CH), 112.11 (CH), 111.02 (CH), 109.90 (C), 54.97 (CH), 27.40 (CH_2_), 21.35 (CH_3_); HRMS (ESI) *m*/*z* [M+H]^+^ calcd. for C_13_H_15_N_2_O_4_^+^ 263.10263, observed 263.10283.

#### 3.1.14. (*S*)-3-(5-Acetoxy-1*H*-indol-3-yl)-2-[(*tert*-butoxycarbonyl)amino]propanoic Acid (**16**) [45]

Boc_2_O (5.7 g, 26.3 mmol, 1.5 eq) was added to a suspension of **15** × AcOH × H_2_O (5.93 g, 17.34 mmol, 1 eq) in a solution of NaHCO_3_ (1.5 g, 17.4 mmol, 1 eq) in 1 m NaOH (35 mL, 2 eq). The mixture was diluted with H_2_O (130 mL) and acetone (200 mL) until all components were dissolved and the homogeneous reaction mixture was stirred for 3 h. Acetone was removed under reduced pressure, and the remaining aqueous phase was washed with Et_2_O (3 × 50 mL), acidified to pH = 2 with 1 m NaHSO_4_, and extracted with Et_2_O (3 × 60 mL). The organic phase was washed with 1 m NaHSO_4_ (50 mL), H_2_O (3 × 50 mL) and brine (2 × 50 mL), dried, and concentrated under reduced pressure to obtain **16** (5.42 g, 14.9 mmol, 86%) as a colorless foam. NMR spectra showed the presence of two rotamers. Only data for the major rotamer are reported. ^1^H-NMR [400 MHz, (CD_3_)_2_SO] δ ppm: 7.35 (d, *J* = 8.7 Hz, 1H), 7.23 (dd, *J* = 4.1, 2.2 Hz, 2H), 7.14 (d, *J* = 8.6 Hz, 1H), 7.06 (d, *J* = 2.4 Hz, 1H), 6.95 (dd, *J* = 10.3, 8.0 Hz, 2H), 6.88–6.79 (m, 2H), 6.61 (dd, *J* = 8.6, 2.3 Hz, 1H), 4.30–4.04 (m, 2H), 3.19–2.81 (m, 3H), 2.27 (s, 3H), 1.35 (d, *J* = 3.3 Hz, 14H), 1.17–1.03 (m, 1H); ^13^C-NMR [101 MHz, (CD_3_)_2_SO] δ ppm: 174.60 (C), 174.34 (C), 170.38 (C), 155.93 (C), 155.85 (C), 150.78 (C), 143.90 (C), 134.32 (C), 131.13 (C), 128.28 (C), 127.79 (C), 125.72 (CH), 124.64 (CH), 115.85 (CH), 112.19 (CH), 111.73 (CH), 111.00 (C), 110.77 (C), 109.63 (CH), 102.54 (CH), 78.51 (C), 65.39 (C), 55.06 (CH), 54.82 (CH), 28.66 (CH_3_), 28.62 (CH_3_), 28.25 (CH_3_), 27.44 (CH_2_), 27.21 (CH_2_), 21.36 (CH_3_), 15.63 (CH_3_); HRMS (ESI) *m*/*z* [M+H]^+^ calcd. for C_18_H_22_N_2_NaO_6_^+^ 385.13701, found 385.13715.

#### 3.1.15. *tert*-Butyl (*S*)-3-(5-acetoxy-1*H*-indol-3-yl)-2-[(*tert*-butoxycarbonyl)amino]propanoate (**17**)

A solution of **16** (5.42 g, 14.9 mmol, 1 eq) and *tert*-butyl 2,2,2-trichloroacetimidate (6.51 g, 29.8 mmol, 2 eq) in anhydrous CH_2_Cl_2_ (75 mL) was stirred at 40 °C for 24 h [46]. The mixture was concentrated under reduced pressure to approximately half of its original volume and stored at −20 °C for 12 h. The precipitate was filtered off and washed with cold CH_2_Cl_2_ (30 mL). The filtrate was successively washed with 10% NaHCO_3_ (30 mL), 1 m NaHSO_4_ (30 mL), H_2_O (30 mL) and brine (30 mL), dried, and concentrated under reduced pressure. The residue was purified via column chromatography (hexane:EtOAc/3:1) to obtain **17** (3.13 g, 7.5 mmol, 50%) as a yellow foam. R_f_ = 0.23 (hexane:EtOAc/1:1); NMR spectra showed the presence of two rotamers. Only data for the major rotamer are reported. ^1^H-NMR (400 MHz, CDCl_3_) δ ppm: 8.45 (s, 1H), 7.35–7.15 (m, 2H), 7.01 (d, *J* = 2.3 Hz, 1H), 6.89 (dd, *J* = 8.7, 2.2 Hz, 1H), 5.11 (d, *J* = 8.1 Hz, 1H), 3.20 (qd, *J* = 14.9, 5.8 Hz, 2H), 2.33 (s, 3H), 1.42 (d, *J* = 21.9 Hz, 18H); ^13^C-NMR (101 MHz, CDCl_3_) δ ppm: 171.35 (C), 170.56 (C), 155.26 (C), 144.20 (C), 133.98 (C), 128.29 (C), 124.22 (CH), 116.18 (CH), 111.59 (CH), 111.10 (CH), 110.83 (C), 81.95 (C), 79.66 (C), 54.67 (CH), 28.33 (CH_3_ × 3), 28.01 (CH_2_), 27.94 (CH_3_ × 3), 21.15 (CH_3_); HRMS (ESI) *m*/*z* [M+H]^+^ calcd. for C_22_H_31_N_2_O_6_^+^ 419.21766, found 419.21789.

#### 3.1.16. *tert*-Butyl (*S*)-3-[5-acetoxy-2,7-bis(4,4,5,5-tetramethyl-1,3,2-dioxaborolan-2-yl)-1*H*-indol-3-yl]-2-[(*tert*-butoxycarbonyl)amino]propanoate (**18**)

Pinacolborane (4.3 g, 5.03 mL, 34.5 mmol, 5 eq) was added to a solution of **17** (2.9 g, 6.9 mmol, 1 eq), [Ir(cod)OMe]_2_ (112.3 mg, 172.6 μmol, 2.5 mol%) and 4,4′-di-*tert*-butyl-2,2′-bipyridine (92.0 mg, 345.1 μmol, 5 mol%) in anhydrous THF (70 mL), and the resulting mixture was heated at 60 °C for 16 h. The reaction mixture was concentrated under reduced pressure and the residue was taken up in Et_2_O (170 mL). The resulting solution was washed with 10% NaHCO_3_ (3 × 150 mL) and brine (3 × 150 mL), dried and concentrated under reduced pressure. The residue was purified via column chromatography [silica gel (0.1% Ca), hexane:EtOAc/4:1] to obtain **18** (2.61 g, 3.9 mmol, 57%) as a colorless foam. R_f_ = 0.38 (silica gel, hexane:EtOAc/2:1); NMR spectra showed the presence of two rotamers. Only data for the major rotamer are reported: ^1^H-NMR (400 MHz, CDCl_3_) δ ppm: 7.48 (d, *J* = 2.3 Hz, 1H), 7.42 (d, *J* = 2.3 Hz, 1H), 7.28 (s, 1H), 4.26–4.00 (m, 1H), 3.46–3.17 (m, 2H), 2.44–2.21 (m, 4H), 1.54–1.38 (m, 42H), 1.38–1.16 (m, 14H); ^13^C-NMR (101 MHz, CDCl_3_) δ ppm: 172.29 (C), 170.39 (C), 155.59 (C), 143.96 (C), 140.84 (C), 127.54 (C), 125.32 (CH), 123.50 (C), 115.04 (CH), 84.39 (C × 2), 84.09 (C × 2), 81.04 (C), 78.91 (C), 56.11 (CH), 28.30 (CH_3_ × 3), 28.00 (CH_3_ × 3), 27.46 (CH_2_), 25.07 (CH_3_ × 3), 24.59 (CH_3_ × 4), 21.09 (CH_3_ × 4), 14.21 (CH_3_), C-B was not observed; HRMS (ESI) *m*/*z* [M+H]^+^ calcd. for C_34_H_53_B_2_N_2_O_10_^+^ 671.38808, found 671.38871.

#### 3.1.17. *tert*-Butyl (*S*)-3-[5-acetoxy-7-(4,4,5,5-tetramethyl-1,3,2-dioxaborolan-2-yl)-1*H*-indol-3-yl]-2-[(*tert*-butoxycarbonyl)amino]propanoate (**19**)

A solution of **18** (0.5 g, 0.75 mmol, 1 eq) and Bi(OAc)_3_ (58 mg, 0.15 mmol, 20 mol%) in MeOH/THF (5:4, 5 mL) was stirred at 80 °C for 3 h [48]. The mixture was concentrated under reduced pressure and the residue was purified via NP column chromatography [silica gel (0.1% Ca), hexane:EtOAc/2:1] followed by RP-chromatography (Chromabond C_18_, 60% MeCN) to obtain **19** (0.21 g, 0.38 mmol, 51%) as a colorless solid. R_f_ = 0.27 (hexane:EtOAc/1:1); NMR spectra showed the presence of two rotamers. Only data for the major rotamer are reported: ^1^H-NMR (400 MHz, CDCl_3_) δ ppm: 9.10 (s, 1H), 7.57–6.95 (m, 3H), 5.06 (d, *J* = 8.1 Hz, 1H), 4.59–4.45 (m, 1H), 3.25 (qd, *J* = 14.9, 5.4 Hz, 2H), 2.31 (s, 3H), 1.53–1.30 (m, 30H); ^13^C-NMR (101 MHz, CDCl_3_) δ ppm: 171.26 (C), 170.43 (C), 155.20 (C), 143.96 (C), 139.12 (C), 127.67 (C), 124.02 (CH), 122.87 (CH), 114.85 (CH), 110.31 (C), 84.07 (C), 81.88 (C), 79.50 (C), 54.66 (CH), 28.32 (CH_3_ × 3), 28.00 (CH_3_ × 3, 27.81 (CH_2_), 25.00 (CH_3_ × 4), 21.05 (CH_3_), C-B was not observed; HRMS (ESI) *m*/*z* [M+H]^+^ calcd. for C_28_H_42_BN_2_O_8_^+^ 545.30287, found 545.30306.

#### 3.1.18. 7-Fluoroindoline (**21**) [59]

NaBH_3_CN (4.7 g, 74 mmol, 2 eq) was added in portions to a solution of 7-fluoroindole (5 g, 37 mmol, 1 eq) in AcOH (20 mL), the reaction mixture was stirred for 3 h and poured into 2 m NaOH (375 mL). The resulting emulsion was extracted with CH_2_Cl_2_ (3 × 100 mL) and the combined organic phases were washed with brine (100 mL), dried and concentrated under reduced pressure. The residue was purified via column chromatography (hexane:EtOAc/10:1) to obtain **21** (3.36 g, 24.5 mmol, 66%) as a colorless liquid. R_f_ = 0.45 (hexane:EtOAc/10:1); ^1^H-NMR (500 MHz, CDCl_3_) δ ppm: 6.94 (dq, *J* = 7.3, 1.1 Hz, 1H), 6.84 (ddq, *J* = 10.0, 8.2, 0.8 Hz, 1H), 6.67 (ddd, *J* = 8.2, 7.3, 4.6 Hz, 1H), 3.83 (s, 1H), 3.65 (t, *J* = 8.4 Hz, 2H), 3.11 (t, *J* = 8.4 Hz, 2H); ^13^C-NMR (101 MHz, CDCl_3_) δ ppm: 149.41 (d, *J* = 239.8 Hz, C-F), 138.41 (d, *J* = 12.6 Hz, C), 133.14 (d, *J* = 4.7 Hz, C), 120.11 (d, *J* = 2.9 Hz, CH), 119.21 (d, *J* = 5.7 Hz, CH), 113.86 (d, *J* = 17.4 Hz, CH), 47.99 (C), 30.21 (d, *J* = 2.2 Hz, C); ^19^F-NMR (376 MHz, CDCl_3_) δ ppm: −135.86; HRMS (ESI) *m*/*z* [M+H]^+^ calcd. for C_8_H_9_FN^+^ 138.07135, found 138.07120.

#### 3.1.19. 5-Hydroxy-7-fluoroindole (**22**)

A solution of potassium nitrosodisulfonate (7.75 g, 28.9 mmol, 2.2 eq; Fremy’s salt, prepared according to [60]) in 0.1 m sodium phosphate buffer (350 mL, pH = 7) was added slowly over 20 min to a solution of **21** (1.8 g, 13.1 mmol, 1 eq) in acetone (180 mL). TLC/HPLC-control was performed every 10 min to monitor consumption of the starting material (30 min total reaction time). After complete conversion, the mixture was diluted with EtOAc (140 mL) and the organic layer was separated. The aqueous layer was extracted with EtOAc (6 × 140 mL) and the combined organic phases were washed with H_2_O (2 × 100 mL) and brine (100 mL), dried and concentrated under reduced pressure. The crude product was purified via column chromatography (hexane:EtOAc/4:1) to obtain **22** (0.62 g, 4.1 mmol, 31%) as a purple liquid, which was immediately used for the next step. R_f_ = 0.32 (hexane:EtOAc/4:1); ^1^H-NMR (400 MHz, CDCl_3_) δ ppm: 8.24 (s, 1H), 7.21 (t, *J* = 2.8 Hz, 1H), 6.86 (dd, *J* = 2.1, 0.7 Hz, 1H), 6.61 (dd, *J* = 11.8, 2.1 Hz, 1H), 6.48 (td, *J* = 3.3, 2.1 Hz, 1H), 5.06 (s, 1H); ^13^C-NMR (101 MHz, CDCl_3_) δ ppm: 150.45 (C), 148.75 (d, *J* = 146.8 Hz, C-F), 131.22 (d, *J* = 6.4 Hz, C), 125.79 (CH), 119.61 (d, *J* = 13.2 Hz, C), 102.79 (d, *J* = 2.7 Hz, CH), 100.75 (d, *J* = 3.6 Hz, CH), 98.15 (d, *J* = 19.6 Hz, CH), 29.73 (C); ^19^F-NMR [376 MHz, (CD_3_)_2_SO] δ ppm: −133.07; elemental analysis: Calcd. for C_8_H_6_FNO: C, 63.57; H, 4.00; N, 9.27. Found: C, 61.9 ± 0.5; H, 4.28 ± 0.08; N, 9.02 ± 0.1; deviation of the found from the calculated value of the carbon content was larger than 0.4%, presumably due to the high instability of the compound.

#### 3.1.20. 5-Acetoxy-7-fluoroindole (**23**) [61]

Ac_2_O (0.44 mL, 0.47 g, 4.62 mmol, 2.2 eq) was added to a solution of **22** (0.33 g, 2.1 mmol, 1 eq) in pyridine (4.2 mL), and the resulting mixture was stirred at ambient temperature for 16 h. The mixture was diluted with H_2_O (42 mL), titrated with 3 m HCl to pH = 2, and extracted with EtOAc (4 × 20 mL). The combined organic phases were washed with 10% NaHCO_3_ (40 mL), dried and concentrated under reduced pressure. The residue was purified via column chromatography (hexane:EtOAc/4:1) to obtain **23** (0.25 g, 1.8 mmol, 86%) as a purple oil. R_f_ = 0.43 (hexane:EtOAc/3:1); ^1^H-NMR (400 MHz, CDCl_3_) δ ppm: 8.45 (s, 1H), 7.23 (t, *J* = 2.8 Hz, 1H), 7.16 (dd, *J* = 1.9, 0.7 Hz, 1H), 6.74 (dd, *J* = 11.1, 1.9 Hz, 1H), 6.57 (td, *J* = 3.3, 2.1 Hz, 1H), 2.35 (s, 3H); ^13^C-NMR (101 MHz, CDCl_3_) δ ppm: 170.30 (C), 148.67 (d, *J* = 245.6 Hz, C-F), 143.68 (C), 130.56 (d, *J* = 6.1 Hz, C), 126.01 (CH), 122.26 (d, *J* = 13.1 Hz, C), 108.61 (d, *J* = 3.9 Hz, CH), 103.68 (d, *J* = 2.6 Hz, CH), 102.31 (d, *J* = 19.5 Hz, CH), 21.12 (C); ^19^F-NMR (376 MHz, CDCl_3_) δ ppm: −123.81; elemental analysis: Calcd. for C_10_H_8_FNO_2_: C, 62.18; H, 4.17; N, 7.25. Found: C, 62.3 ± 0.1; H, 4.21 ± 0.04; N, 7.36 ± 0.02.

#### 3.1.21. Methyl 2-Acetamido-3-(5-acetoxy-7-fluoro-1*H*-indol-3-yl)propanoate (**24**) [50]

1 m EtAlCl_2_ in hexane (20 g, 26 mL, 26 mmol, 5 eq) was added dropwise to an ice-cold solution of methyl 2-acetamidoacrylate (0.74 g, 5.2 mmol, 1 eq) and **23** (1 g, 5.2 mmol, 1 eq) in anhydrous CH_2_Cl_2_ (20 mL). The reaction mixture was stirred at 0 °C for 2 h and at ambient temperature for 16 h and then poured into cold 10% NaHCO_3_ (50 mL). The resulting suspension was filtered through a layer of celite, and the filtrate was extracted with CH_2_Cl_2_ (50 mL). The organic phase was dried and concentrated under reduced pressure. The residue was purified via column chromatography (hexane:EtOAc/gradient; 1:2 to 100% EtOAc) to obtain **24** (0.26 g, 0.77 mmol, 14%) as a colorless foam. R_f_ = 0.38 (hexane:EtOAc/1:2); ^1^H-NMR (400 MHz, CDCl_3_) δ ppm: 8.56 (s, 1H), 7.28 (s, 0H), 7.07–6.99 (m, 2H), 6.71 (dd, *J* = 11.0, 1.9 Hz, 1H), 6.09 (d, *J* = 7.8 Hz, 1H), 4.94 (dt, *J* = 7.8, 5.2 Hz, 1H), 3.72 (s, 3H), 3.37–3.15 (m, 2H), 2.33 (s, 3H), 2.00 (s, 3H); ^13^C-NMR (101 MHz, CDCl_3_) δ ppm: 172.18 (C), 170.18 (C), 169.99 (C), 148.69 (d, *J* = 246.5 Hz, C), 143.84 (d, *J* = 8.8 Hz, C), 130.58 (C), 124.60 (CH), 122.40 (C), 111.42 (d, *J* = 2.3 Hz, C), 106.89 (d, *J* = 3.7 Hz, CH), 102.70 (d, *J* = 19.4 Hz, CH), 53.07 (CH), 52.50 (CH_3_), 27.56 (CH_2_), 23.19 (CH_3_), 21.06 (CH_3_); ^19^F-NMR (376 MHz, CDCl_3_) δ ppm: −132.62; HRMS (ESI) *m*/*z* [M+H]^+^ calcd. for C_16_H_18_FN_2_O_5_^+^ 337.11943, found 337.11935.

#### 3.1.22. *tert*-Butyl 5-Acetoxy-3-{2-[*N*-(*tert*-butoxycarbonyl)acetamido]-3-methoxy-3-oxopropyl}-7-fluoro-1*H*-indole-1-carboxylate (**25**)

A solution of **24** (0.26 g, 0.77 mmol, 1 eq), DMAP (9.4 mg, 0.077 mmol, 0.1 eq) and Boc_2_O (1.01 g, 4.62 mmol, 6 eq) in anhydrous MeCN (2.5 mL) was left to stand at ambient temperature for 3 d. The mixture was concentrated under reduced pressure and the residue taken up in Et_2_O (25 mL). The ethereal phase was washed with 1 m NaHSO_4_ (25 mL), saturated NaHCO_3_ (25 mL), H_2_O (25 mL) and brine (25 mL), dried, and concentrated under reduced pressure. The residue was purified via column chromatography (hexane:EtOAc/3:1) to obtain **25** (0.263 g, 0.49 mmol, 63%) as a yellow oil. R_f_ = 0.37 (hexane:EtOAc/3:1); ^1^H-NMR (400 MHz, CDCl_3_) δ ppm: 7.47 (s, 1H), 7.28 (s, 1H), 7.06 (d, *J* = 2.1 Hz, 1H), 6.84 (dd, *J* = 12.4, 2.1 Hz, 1H), 5.42 (dd, *J* = 9.6, 5.1 Hz, 1H), 3.77 (s, 3H), 3.50 (ddd, *J* = 15.1, 5.1, 1.0 Hz, 1H), 3.29–3.18 (m, 1H), 2.40 (s, 3H), 2.33 (s, 3H), 1.64 (s, 9H), 1.39 (s, 9H); ^13^C-NMR (101 MHz, CDCl_3_) δ ppm: 172.75 (C), 170.40 (C), 169.42 (C), 151.36 (d, *J* = 126.5 Hz, C), 148.48 (C), 148.20 (C), 146.39 (C), 134.52 (C), 127.72 (CH), 120.05 (C), 116.66 (C), 107.25 (d, *J* = 4.1 Hz, CH), 106.58 (d, *J* = 25.5 Hz, CH), 84.29 (d, *J* = 8.6 Hz, C), 56.11 (CH), 52.40 (CH_3_), 27.96 (CH_3_ × 3), 27.69 (CH_3_ × 3), 26.56 (CH_3_), 24.97 (CH_2_), 21.05 (CH_3_); ^19^F-NMR [376 MHz, (CD_3_)_2_SO] δ ppm: −113.08; HRMS (ESI) *m*/*z* [M+Na]^+^ calcd. for C_26_H_33_FN_2_NaO_9_^+^ 559.20623, found 559.20601.

#### 3.1.23. 3-[1-(*tert*-Butoxycarbonyl)-7-fluoro-5-hydroxy-1*H*-indol-3-yl]-2-[(*tert*-butoxycarbonyl)amino]propanoic Acid (**26**)

NaOH (0.1 g, 2.45 mmol, 5 eq) was added to a solution of **25** (0.263 g, 0.49 mmol, 1 eq) in 67% MeOH (2.46 mL). The reaction mixture was stirred at 50 °C for 16 h and concentrated under reduced pressure to approx. 1.2 mL. The remaining aqueous solution was washed with Et_2_O (10 mL), and the pH was adjusted to 6 with 1 m NaHSO_4_. The resulting emulsion was extracted with EtOAc (3 × 15 mL), and the combined organic phases were dried and concentrated under reduced pressure to obtain **26** (0.162 g, 0.37 mmol, 75%) as a colorless oil. ^1^H-NMR [400 MHz, (CD_3_)_2_SO] δ ppm: 9.66 (s, 1H), 7.48 (s, 1H), 7.16 (d, *J* = 8.4 Hz, 1H), 6.74 (d, *J* = 2.1 Hz, 1H), 6.59 (dd, *J* = 13.8, 2.1 Hz, 1H), 4.15 (ddd, *J* = 10.3, 8.3, 4.2 Hz, 1H), 3.00 (dd, *J* = 14.8, 4.3 Hz, 1H), 2.85 (dd, *J* = 14.8, 10.2 Hz, 1H), 2.60–2.42 (m, 3H), 1.57 (s, 9H), 1.33 (s, 9H); ^13^C-NMR [101 MHz, (CD_3_)_2_SO] δ ppm: 173.95 (C), 155.29 (d, *J* = 123.7 Hz, C), 150.90 (C), 148.61 (C), 148.40 (C), 135.26 (C), 127.30 (CH), 116.88 (C), 115.41 (C), 101.40 (d, *J* = 24.1 Hz, CH), 100.24 (CH), 83.81 (C), 78.58 (C), 53.52 (CH), 28.58 (CH_3_ × 3), 28.00 (CH_3_ × 3), 26.82 (CH_2_); ^19^F-NMR [376 MHz, (CD_3_)_2_SO] δ ppm: −114.47; HRMS (ESI) *m*/*z* [M+Na]^+^ calcd. for C_21_H_27_FN_2_NaO_7_^+^ 461.16945, found 461.16956.

#### 3.1.24. 2-Amino-3-(7-fluoro-5-hydroxy-1*H*-indol-3-yl)propanoic Acid Trifluoroacetate (**27** × TFA)

H_2_O (7.5 µL, 2.5 vol% of total solution), triisopropylsilane (7.5 µL, 2.5 vol% of total solution) and ice-cold trifluoroacetic acid (TFA) (285 µL, 95 vol% of total solution) were sequentially added to **26** (100 mg, 0.23 mmol). The mixture was manually stirred for 5 min at 4 °C and concentrated under reduced pressure at ambient temperature. The residue was taken up in Et_2_O and sonicated in an ultrasound bath. The resulting precipitate was centrifuged off, and the crude product was purified via preparative HPLC and freeze dried to obtain **27** × TFA (33.4 mg, 0.1 mmol, 43%) as a colorless solid. ^1^H-NMR (400 MHz, CD_3_OD) δ ppm: 7.19 (s, 1H), 6.80 (d, *J* = 2.0 Hz, 1H), 6.50 (dd, *J* = 12.4, 2.0 Hz, 1H), 4.23 (dd, *J* = 8.2, 4.7 Hz, 1H), 3.44 (ddd, *J* = 15.3, 4.8, 0.8 Hz, 1H), 3.26 (dd, *J* = 15.3, 8.2 Hz, 1H); ^13^C-NMR (101 MHz, CD_3_OD) δ ppm: 170.21 (C), 150.82 (d, *J* = 8.8 Hz, C), 149.41 (d, *J* = 243.5 Hz, C), 130.57 (d, *J* = 6.9 Hz, C), 125.64 (CH), 119.65 (d, *J* = 13.8 Hz, C), 106.73 (d, *J* = 2.5 Hz, C), 97.63 (d, *J* = 3.5 Hz, CH), 97.44 (d, *J* = 19.1 Hz, CH), 52.98 (CH), 26.25 (CH_2_); ^19^F-NMR (376 MHz, CD_3_OD) δ ppm: −77.09 (TFA), −135.25; HRMS (ESI) *m*/*z* [M+H-TFA]^+^ calcd. for C_11_H_12_FN_2_O_3_^+^ 239.08264, found 293.08298.

### 3.2. Radiochemistry

#### 3.2.1. General

No-carrier-added aqueous [^18^F]fluoride was produced via the ^18^O(p,n)^18^F nuclear reaction through the bombardment of enriched [^18^O]H_2_O with 17 MeV protons in a BC1710 cyclotron (The Japan Steel Works, Tokyo, Japan) or a GE PETtrace (GE Healthcare, Chicago, IL, USA), both at the INM-5 (Forschungszentrum Jülich). Radioactivity was measured using a CRC-55tR Dose Calibrator from Capintec, Inc. (Florham Park, The Netherlands) and/or a Curiementor 2 (PTW, Freiburg, Germany). QMA carbonate light plus cartridges (130 mg, Waters GmbH, Eschborn, Germany) were preconditioned with 2 mL H_2_O. SepPak C18 light cartridges (130 mg, Waters GmbH, Eschborn, Germany) were preconditioned with 5 mL EtOH followed by 5 mL H_2_O.

#### 3.2.2. Semi-Preparative HPLC

Semi-preparative HPLC was performed on a dedicated semi-preparative HPLC system consisting of a Knauer K-100 pump (Knauer Wissenschaftliche Geräte GmbH, Berlin, Germany), a Knauer K-2501 UV Detector (Knauer Wissenschaftliche Geräte GmbH, Berlin, Germany), a Rheodyne 6 port injection valve equipped with a 2 ml injection loop, and a custom-made Geiger counter. HPLC columns were purchased from Phenomenex (Aschaffenburg, Germany) and Merck KGaA (Darmstadt, Germany). The identity of ^18^F-labeled tracers and their enantiomeric excess (ee) were determined via co-injection of an authentic sample of the respective reference compound. The UV and radioactivity detectors were connected in series, giving a time delay of 0.2–0.9 min between the corresponding responses depending on the flow rate.

Acetate buffer: AcOH (11 mL, 192.34 mmol) was added to a solution of NaOAc (10 g, 122 mmol) in H_2_O (60 mL), and the resulting mixture was diluted with H_2_O to 100 mL. An aliquot of this solution (10 mL) was further diluted with H_2_O to 1 L and used as HPLC eluent (pH ≈ 5).

Method A: Column: Luna C18(2), 5 μm, 100 Å, 250 × 10 mm equipped with the appropriate SecurityGuard™ cartridge (2 × 3 mm) (Phenomenex, Aschaffenburg, Germany); eluent: 30% EtOH in acetate buffer; flow rate: 3.5 mL/min.

Method B: Column: Luna C18(2), 5 μm, 100 Å, 250 × 10 mm equipped with the appropriate SecurityGuard™ cartridge (2 × 3 mm) (Phenomenex, Aschaffenburg, Germany); eluent: 8% EtOH in acetate buffer; flow rate: 3.5 mL/min.

#### 3.2.3. Analytical HPLC

HPLC analyses were carried out on a Dionex Ultimate^®^ 3000 System (Thermo Fisher Scientific Inc., Bremen, Germany) with Ultimate^®^ 3000 variable wavelength detector coupled in series with a Berthold LB500 NaI detector (Berthold Technologies, Bad Wildbad, Germany). Two Rheodyne 6 port injection valves equipped with equal sample loops were installed before and behind the chromatographic column. Radiochemical conversions (RCCs) were determined using post-column injection as described previously [62]. Isolated yields of radiochemically and chemically pure ^18^F-labeled compounds are reported as decay-corrected radiochemical yields (RCYs). The UV and radioactivity detectors were connected in series, giving a time delay of 0.1–0.3 min between the corresponding responses depending on the flow rate.

Method C: Column: Synergi Hydro-RP, 4 µm, 80 Å, 250 × 4.6 mm equipped with the appropriate SecurityGuard™ cartridge (2 × 3 mm) (Phenomenex, Aschaffenburg, Germany); eluent: 20% MeCN (0.1% TFA); flow rate: 1 mL/min.

Method D: Column: Synergi Hydro-RP, 4 µm, 80 Å, 250 × 4.6 mm equipped with the appropriate SecurityGuard™ cartridge (2 × 3 mm) (Phenomenex, Aschaffenburg, Germany); eluent: 10% MeCN (0.1% TFA); flow rate: 1.5 mL/min.

Method E: Column: Astec Chitobiotic T, 5 µm, 250 × 10 mm (Sigma-Aldrich Chemie GmbH, Taufkirchen, Germany) equipped with the appropriate SecurityGuard™ cartridge (2 × 3 mm); eluent: 70% MeOH (0.02% HCO_2_H); flow rate: 3 mL/min.

#### 3.2.4. (*S*)-2-Amino-3-(6-[^18^F]fluoro-1-methyl-1*H*-indol-3-yl)propanoic Acid (*N*_In_-Me-6-[^18^F]FTrp, [^18^F]**13**)

[^18^F]Fluoride (0.5–10 GBq) in [^18^O]H_2_O was loaded (from the male side) onto a QMA carbonate light plus cartridge. The cartridge was washed with MeOH (2 mL), dried with air (10 mL), and eluted (from the female side) with a solution of Et_4_NHCO_3_ (1 mg, 5.23 µmol) in MeOH (0.5 mL). If the QMA cartridge was loaded, flushed, and eluted from the female side only, a significant amount of [^18^F]fluoride sometimes remained on the resin (probably because QMA-light cartridges have a single frit on the male side but four frits on the female side). MeOH was removed within 5 min at 80 °C under reduced pressure (300–400 mbar) in a stream of argon. A solution of precursor **6** (7.7 mg, 10 µmol) and Cu(OTf)_2_(Py)_4_ (6.8 mg, 10 µmol) in DMA/*n*BuOH (2:1, 0.75 mL) was added, the reaction mixture was stirred under air at 110 °C for 15 min, and then concentrated at the same temperature under reduced pressure (300–400 mbar) in a stream of argon for 10 min. The residue was taken up in 2 m HCl (0.3 mL) and stirred at 110 °C for 10 min to decompose the intermediate (*S*,*S*)-Ni-BPB-*N*_In_-Me-6-[^18^F]FTrp ([^18^F]**28**) into [^18^F]**13**. The HCl was removed under reduced pressure (300–400 mbar) in a stream of argon for 10 min, the residue was diluted with HPLC mobile phase (30% EtOH/acetate buffer, 1.5 mL), and the radiolabeled product was isolated by semi-preparative HPLC (method A, product fraction at 11.5–12.5 min). The purity of the isolated product was analyzed according to method C {R_t_([^18^F]**13**): 13.5 min}. Enantiomeric excess was determined according to method E {R_t_([^18^F]**13**): 9.7 min}. The product [^18^F]**13** was obtained as a ready-to-use solution in RCYs of 45 ± 7% within 83 ± 8 min with a radiochemical purity (RCP) of >99% and an ee of >99%. Molar activities (A_m_) amounted to 66 ± 33 GBq/µmol (1–1.5 GBq tracer).

#### 3.2.5. 2-Amino-3-(7-[^18^F]fluoro-5-hydroxy-1*H*-indol-3-yl)propanoic Acid (5-HO-7-[^18^F]FTrp, [^18^F]**27**)

[^18^F]Fluoride (0.5–10 GBq) in [^18^O]H_2_O was loaded (from the male side) onto a QMA carbonate light plus cartridge. The cartridge was washed with MeOH (2 mL), dried with air (10 mL), and eluted (from the female side) with a solution of Et_4_NHCO_3_ (1 mg, 5.23 µmol) in MeOH (0.5 mL). If the QMA cartridge was loaded, flushed, and eluted from the female side only, a significant amount of [^18^F]fluoride sometimes remained on the resin (probably because QMA-light cartridges have a single frit on the male side but four frits on the female side). MeOH was removed within 5 min at 80 °C under reduced pressure (300–400 mbar) in a stream of argon. A solution of precursor **19** (5.4 mg, 10 µmol) and Cu(OTf)_2_(3,4-Me_2_-Py)_4_ (6.8 mg, 10 µmol) in DMI (0.75 mL) was added, and the reaction mixture was stirred under air at 110 °C for 15 min. The reaction mixture was then diluted with H_2_O (7 mL), and the resulting suspension was loaded (from the female side) onto a SepPak C18 light cartridge. The cartridge was washed with H_2_O (5 mL) and dried with air (20 mL), and the radiolabeled intermediate (*S*)-Boc-5-AcO-7-[^18^F]FTrp-O*t*Bu ([^18^F]**29**) was eluted (from the female side) with MeOH (1 mL). MeOH was removed within 5 min at 80 °C under reduced pressure (300–400 mbar) in a stream of argon. The residue was taken up in 2 m HCl (0.3 mL) and stirred at 110 °C for 10 min to deprotect [^18^F]**29** into [^18^F]**27**. 2 m NaOH (0.25 mL) was added, the mixture was diluted with HPLC mobile phase (8% EtOH in acetate buffer, 1 mL), and the radiolabeled product was isolated using semi-preparative HPLC (method B, product fraction at 13–14 min). The purity of the isolated product was analyzed according to method D {R_t_([^18^F]**27**): 7.9 min}. Enantiomeric excess was determined according to method E {R_t_([^18^F]**27**): 7.7 min}. The product [^18^F]**27** was obtained as a ready-to-use solution in RCYs of 29 ± 4% within 81 ± 3 min, with an RCP of >99% and ee of >99%. A_m_ amounted to 46 ± 17 GBq/µmol (1.05–1.35 GBq tracer).

## 4. Conclusions

In summary, *N*_In_-Me-6-[^18^F]FTrp ([^18^F]**13**) and 5-HO-7-[^18^F]FTrp ([^18^F]**27**) were efficiently prepared in RCYs of 45 ± 7% and 29 ± 4% via Cu-mediated radiofluorination of the respective pinacol boronate precursors and subsequent decomposition/deprotection of the resulting radiolabeled intermediates. In addition, feasible synthetic routes for preparation of the hitherto unknown radiolabeling precursors and non-radioactive reference compounds were developed. The latter proved to be especially challenging in the case of 5-HO-7-FTrp, synthesis of which ultimately succeeded starting from 7-fluoroindole using a route that involves oxidation with Fremy’s salt and Friedel–Crafts alkylation as key steps. As such, our work provides access to two promising new ^18^F-labeled Trp derivatives (and the corresponding non-labeled reference compounds) in amounts sufficient for their preclinical evaluation as candidate PET-tracers for pathway-specific visualization of Trp metabolism.

## Data Availability

The data presented in this study are contained within the article and Supplementary Materials or available from the authors on reasonable request.

## Supplementary Materials

The supporting information can be downloaded at: https://www.mdpi.com/article/10.3390/ijms242015251/s1. Description of unsuccessful syntheses of 5-hydroxy-7-fluorotryptophan (**27**); HPLC chromatograms and ^1^H-, ^13^C- and ^19^F-NMR spectra of all compounds. Reference [63] is cited in the supplementary materials.
