# Supplementary material for: Preparation of NIn-Methyl-6-[18F]fluoro- and 5-Hydroxy-7-[18F]fluorotryptophans as Candidate PET-Tracers for Pathway-Specific Visualization of Tryptophan Metabolism
Source: Int J Mol Sci. 2023 Oct 17;24(20):15251. doi: 10.3390/ijms242015251 (PMC10607147; doi:10.3390/ijms242015251)
Supplement: Supplementary file 1 [file ijms-24-15251-s001.zip › ijms-2640407-supplementary.pdf]

Supporting Information

Preparation of N_{In}-methyl-6-[^{18}F]fluoro- and 5-hydroxy-7-[^{18}F]fluoro-tryptophans as candidate PET-tracers for pathway-specific visualization of tryptophan metabolism

Niklas Kolks, Felix Neumaier, Bernd Neumaier and Boris D. Zlatopolskiy

Table of contents

1 Materials and Methods 2
1.1 General 2
1.2 Nuclear magnetic resonance (NMR) spectroscopy 2
1.3 Mass spectrometry (MS) 2
1.4 Column chromatography 2
1.5 Thin layer chromatography (TLC) 3
1.6 High-performance liquid chromatography (HPLC) 3
1.7 Chemistry 3
2 Organic syntheses 4
2.1 Unsuccessful synthetic pathways for 5-hydroxy-7-fluorotryptophan 4
3 Characterization 37
3.1 ^{1}H-, ^{13}C- and ^{19}F-NMR-spectra 37
3.2 HPLC-Data 100
4 References 108

1 Materials and Methods

1.1 General

Unless noted otherwise, all chemicals and solvents were purchased from VWR International (Radnor, PA, USA), Sigma-Aldrich (Steinheim, Germany), ChemPUR (Karlsruhe, Germany), ABCR GmbH (Karlsruhe, Germany) or Fluka AG (Buchs, Switzerland) and used without further purification.

1.2 Nuclear magnetic resonance (NMR) spectroscopy

NMR spectra were measured at ambient temperature in deuterium oxide (D_2O), deuterochloroform ($CDCl_3$), deuteromethanol (CD_3OD) or deuterodimethylsulfoxide [$(CD_3)_2SO$] as indicated. ^{1}H-NMR spectra were measured using a Bruker Avance II 300 (300 MHz), a Bruker Avance 200 (200 MHz) or a Varian Inova (400 MHz) spectrometer. ^{13}C-NMR spectra [additional APT (Attached Proton Test)] were measured using a Bruker DPX Avance 200 (50 MHz), a Bruker Avance II 300 (75 MHz) or a Varian INOVA 400 (101 MHz) spectrometer. ^{19}F-NMR spectra were measured using a Varian INOVA 400 (376 MHz) spectrometer. The measured chemical shifts (δ) are reported in parts per million (ppm) relative to residual peaks of non-deuterated solvents. The observed signal multiplicities are characterized as follows: s = singlet, d = doublet, t = triplet, q = quartet, m = multiplet, dd = doublet of doublets, dt = doublet of triplets, dq = doublet of quartets, ddd = doublet of doublets of doublets, ddt = doublet of doublets of triplets, dddd = doublet of doublets of doublets of doublets, td = triplet of doublets and qd = quartet of doublets. Coupling constants *J* are reported in Hertz (Hz).

1.3 Mass spectrometry (MS)

Low resolution mass spectra (LRMS) were measured with an MSQ PlusTM mass spectrometer (Thermo Electron Corporation, San Jose, USA). High resolution mass spectra were measured with a FTICR "LTQ FT Ultra" (Thermo Fisher Scientific Inc., Bremen, Germany).

1.4 Column chromatography

Manual column chromatography was performed with silica gel, 60 Å, 230–400 mesh particle size from Fluka AG (Buchs, Switzerland) or silica gel (w/Ca, 0.1%), 60 Å, 230–400 mesh particle size from Sigma-Aldrich GmbH (Steinheim, Germany).

Automated column chromatography was either performed on a Grace Reverelis X1 (Columbia, Maryland, USA) or on a Büchi Pure C-815 Flash system using Si60 FlashPure cartridges or Reveleris™ C_{18} reversed phase cartridges.

1.5 Thin layer chromatography (TLC)

TLC was performed using aluminum sheets coated with silica gel 0.25 mm SIL G/UV 254 (Merck KGaA, Darmstadt, Germany). Chromatograms were inspected under UV light (λ = 254 nm) and/or stained with phosphomolybdic acid (20% in EtOH).

1.6 High-performance liquid chromatography (HPLC)

HPLC analyses were carried out on a Dionex Ultimate® 3000 System with Ultimate® 3000 variable wavelength detector coupled in series with a Berthold LB500 NaI detector. Two Rheodyne 6 port injection valves equipped with equal sample loops were installed before and behind the chromatographic column. The UV and radioactivity detectors were connected in series, giving a time delay of 0.1–0.2 min between the corresponding responses, depending on the flow rate. Semi-preparative HPLC was performed on a dedicated semi-preparative system consisting of a Knauer K-100 pump, a Knauer K-2501 UV Detector, a Rheodyne 6 port injection valve equipped with a 2 mL injection loop and a custom-made Geiger counter. HPLC columns were purchased from Phenomenex (Aschaffenburg, Germany) and Merck KGaA (Darmstadt, Germany).

1.7 Chemistry

All reactions were carried out with magnetic stirring. Organic extracts were dried over anhydrous $MgSO_4$. Air or moisture sensitive reagents were handled under argon (>99.999%, Air Liquide GmbH). Solutions were concentrated under reduced pressure (1–900 mbar) at 40–50 °C using a rotary evaporator (Büchi Labortechnik, Essen, Germany). Solvent proportions are indicated in a volume/volume ratio.

2 Organic syntheses

2.1 Unsuccessful synthetic pathways for 5-hydroxy-7-fluorotryptophan

Several alternative synthetic pathways for the reference structure 5-HO-7-FTrp were attempted but proved to be unfeasible, as documented in the following subsections.

2.1.1 Via indole synthesis using Bartoli-reaction

2.1.1.1 Proposed synthetic route:

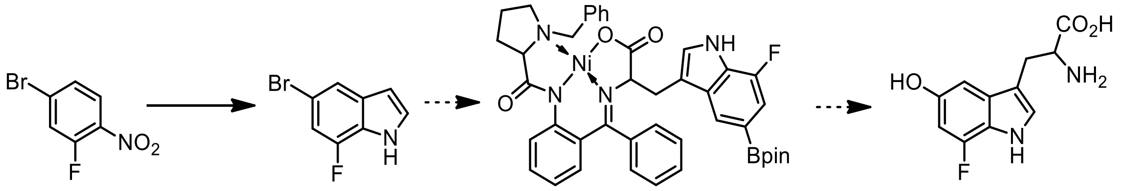

2.1.1.2 Preparation of 5-bromo-7-fluoroindole (S1) [1]

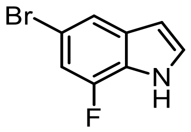

S1

Under argon, dry magnesium (1 eq, 16.36 mmol, 0.39 g) was placed in a pre-dried three necked flask equipped with a thermometer, a reflux-condenser and a dropping-funnel. The Mg was suspended in dry THF (8 mL), the funnel was filled with 1 M vinylbromide in THF (1.1 eq, 18 mmol, 18 mL) and the reaction was started by slow addition of the vinylbromide and heating of the flask. When the reaction started (temperature increase and color change from clear to yellow), the flask was placed in a cooling bath to keep the reaction temperature at 50-60 °C. After complete addition of vinylbromide, the reaction mixture was cooled to ambient temperature. Based on titration with I_2 in THF, the concentration of the vinylmagnesium bromide solution thus obtained amounted to 0.46 M.

4-Bromo-2-fluoronitrobenzene (1 g, 4.5 mmol, 1 eq) was dissolved in THF (17 mL) and the freshly prepared vinylmagnesium bromide solution (29.3 mL, 13.5 mmol, 3 eq) was slowly added over the course of 30 min at –40 °C. The mixture was stirred for 1 h at –40 °C, poured into saturated NH_4Cl (10 mL) and extracted with EtOAc. The organic phase was dried and concentrated under reduced pressure and the residue was purified by column chromatography (silica gel, hexane:EtOAc / gradient, 20:1 to 10:1) and RP chromatography (MeCN/water, gradient) to obtain the desired product as a yellow oil (0.115 g, 0.54 mmol, 12%).

Yield 12%.

^{1}H-NMR (400 MHz, $CDCl_3$) δ ppm: 8.39 (s, 1H), 7.59 (dd, J = 1.6, 0.7 Hz, 1H), 7.32 – 7.20 (m, 1H), 7.09 (dd, J = 10.3, 1.6 Hz, 1H), 6.56 (td, J = 3.3, 2.1 Hz, 1H).

^{13}C-NMR (101 MHz, $CDCl_3$) δ ppm: 149.02 (d, J = 248.3 Hz, C-F), 132.41 (d, J = 5.7 Hz, C), 125.88 (CH), 123.11 (CH), 119.22 (d, J = 3.6 Hz, CH), 111.65 (d, J = 8.0 Hz, C), 110.59 (d, J = 19.7 Hz, CH), 103.07 (d, J = 2.2 Hz, CH).

^{19}F-NMR (376 MHz, $CDCl_3$) δ ppm: –132.66.

LRMS (ESI) *m/z* $[M+H]^+$ calcd. for $C_8H_6BrFN^+$ 213.97, found 214.05.

It was not possible to reproduce the yield of S1 given in the literature [1] with either commercial or freshly prepared vinylmagnesium bromide. Production of the necessary amount of indole would have required more starting material than available.

2.1.2 Via indole synthesis using iodinated aniline for Cassar-Sonogashira-reaction

2.1.2.1 Proposed synthetic route:

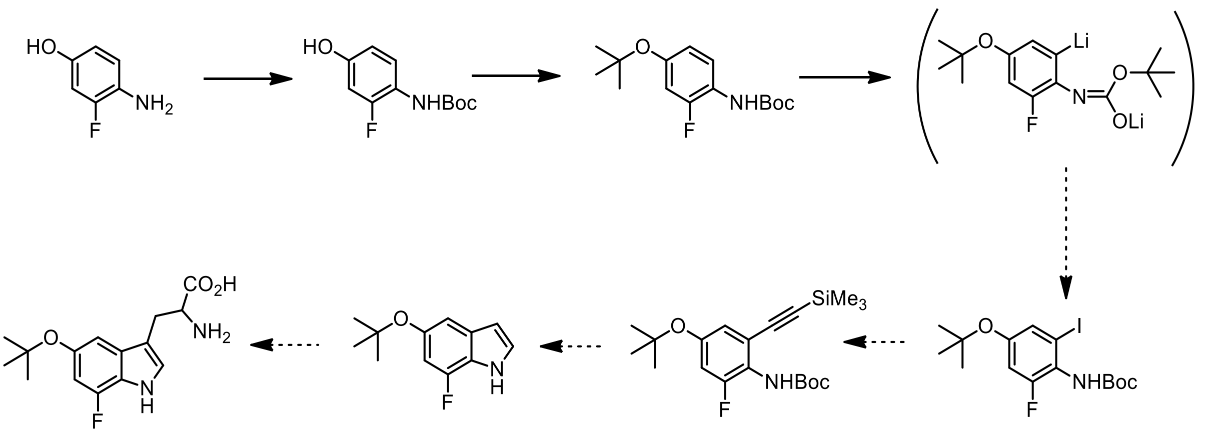

2.1.2.2 Preparation of *tert*-butyl (2-fluoro-4-hydroxyphenyl)carbamate (S2) [1]

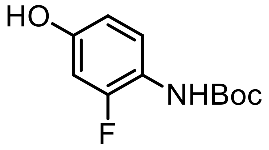

S2

4-Amino-3-fluorophenol (2.5 g, 19.6 mmol, 1 eq) was added to a mixture of Boc_2O (4.3 g, 19.6 mmol, 1 eq) and $InCl_3$ (43 mg, 0.196 mmol, 1 mol%) at 35 °C and the reaction mixture was stirred for 1 h (until TLC/HPLC indicated complete conversion of the starting material). The mixture was diluted with EtOAc (200 mL), washed with water (80 mL), dried, and concentrated under reduced pressure. The residue was purified by column chromatography (silica gel, hexane:EtOAc / 3:1) to obtain the desired product as a clear, light-brown oil (3.951 g, 17.4 mmol, 89%).

Yield 89%.

^{1}H-NMR (400 MHz, $CDCl_3$) δ ppm: 7.56 (d, J = 9.8 Hz, 1H), 6.62 – 6.49 (m, 2H), 6.42 (s, 1H), 2.21 (s, 1H), 1.53 (d, J = 1.2 Hz, 9H).

^{13}C-NMR (101 MHz, $CDCl_3$) δ ppm: 154.62 (d, J = 164.8 Hz, C-F), 153.30 (d, J = 11.0 Hz, C), 153.02 (C), 123.65 (CH), 118.37 (d, J = 11.5 Hz, C), 111.30 (d, J = 3.2 Hz, CH), 103.35 (d, J = 22.5 Hz, CH), 81.14 (C), 28.32 (CH_3 × 3).

^{19}F-NMR (376 MHz, $CDCl_3$) δ ppm: –127.39.

LRMS (ESI) *m/z* $[M+H]^+$ calcd. for $C_{11}H_{15}FNO_3^+$ 228.24, found 228.28.

2.1.2.3 Preparation of *tert*-butyl (4-(*tert*-butoxy)-2-fluorophenyl)carbamate (S3) [1]

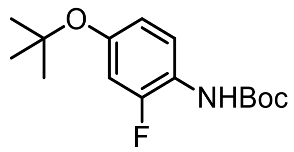

S3

S2 (3.45 g, 15 mmol, 1 eq) was dissolved in CH_2Cl_2 (24 mL), $Mg(ClO_4)_2$ (0.345 g, 1.5 mmol, 0.1 eq, dried at 150 °C for 2 h) and Boc_2O (7.85 g, 34.5 mmol, 2.3 eq) were added and the mixture was stirred at 40 °C overnight (the reaction progress was monitored via TLC and HPLC). Additional $Mg(ClO_4)_2$ (0.345 g, 1.5 mmol, 0.1 eq, dried at 150 °C for 2 h) and Boc_2O (7.85 g, 34.5 mmol, 2.3 eq) were added and the mixture was stirred at 40 °C for 3 d. The mixture was then diluted with H_2O (80 mL), extracted with CH_2Cl_2 (2 × 150 mL), dried, and concentrated under reduced pressure. The residue was purified via column chromatography (silica gel, hexane:EtOAc / 4:1) to obtain the desired product as a yellowish solid (2.05 g, 7.2 mmol, 48%).

Yield 48%.

^{1}H-NMR (400 MHz, $CDCl_3$) δ ppm: 7.90 (d, J = 9.8 Hz, 1H), 6.87 – 6.70 (m, 2H), 6.58 (s, 1H), 1.54 (s, 9H), 1.34 (s, 9H).

^{13}C-NMR (101 MHz, $CDCl_3$) δ ppm: 152.65 (C), 151.98 (d, J = 232.7 Hz, C-F), 150.72 (C), 122.46 (d, J = 10.5 Hz, C), 120.16 (d, J = 3.0 Hz, CH), 111.30 (d, J = 19.5 Hz, CH), 80.83 (C), 79.10 (C), 28.69 (CH_3 × 3), 28.29 (CH_3 × 3).

^{19}F-NMR (376 MHz, $CDCl_3$) δ ppm: –133.52.

LRMS (ESI) *m/z* $[M+H]^+$ calcd. for $C_{15}H_{23}FNO_3^+$ 284.35, found 284.37.

2.1.2.4 Attempt to prepare *tert*-butyl [4-(*tert*-butoxy)-2-fluoro-6-iodophenyl]carbamate (S4) [1]

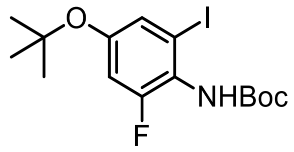

S4

tert-Butyllithium (42.5 mmol, 2 eq) in pentane (25 mL) was added to a solution of **S3** (6 g, 21.25 mmol, 1 eq) in anhydrous THF (40 mL) cooled in an acetone-dry ice bath. After 3 h at –50 °C, the mixture was treated with iodine (2.7 g, 10.63 mmol, 0.5 eq) and allowed to warm to ambient temperature. Saturated $Na_2S_2O_3$ (50 mL) was added and the reaction mixture was extracted with Et_2O (3 × 50 mL). Evaporation and crystallization from hexanes did not afford the desired product.

It was not possible to obtain the product according to the procedure described in the literature [1].

2.1.3 Via cyclization to form tryptophan

2.1.3.1 Proposed synthetic route:

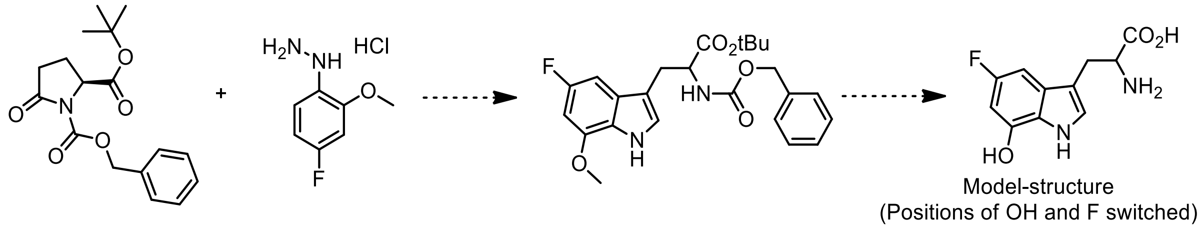

2.1.3.2 Preparation of (*S*)-1-benzyl 2-*tert*-butyl 5-oxopyrrolidine-1,2-dicarboxylate (S5)

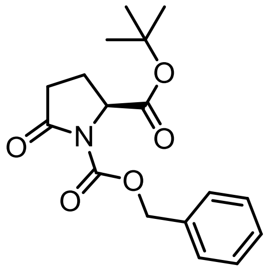

S5

NaH (60% in oil, 1.18 g, 29.6 mmol, 1.1 eq) was added in small portions to a solution of *tert*-butyl pyroglutamate (5 g, 26.9 mmol, 1 eq) in anhydrous THF (80 mL) and the reaction mixture was stirred for 30 min. The mixture was cooled to –50 °C and benzylchloroformate (5 g, 4.2 mL, 29.6 mmol, 1.1 eq) was added. The mixture was stirred for 20 h at ambient temperature and concentrated under reduced pressure. Saturated NH_4Cl (200 mL) was added and the mixture was extracted with EtOAc (2 × 200 mL). The combined organic fractions were dried and concentrated under reduced pressure. The residue was purified via column chromatography (silica gel, hexane:EtOAc / 2:1) to obtain the desired product as a colorless oil (7.05 g, 22.1 mmol, 82%).

Yield 82%.

^{1}H-NMR (400 MHz, $CDCl_3$) δ ppm: 7.46 – 7.27 (m, 5H), 5.39 – 5.18 (m, 2H), 4.56 (dd, J = 9.4, 2.6 Hz, 1H), 2.65 (ddd, J = 17.6, 10.6, 9.5 Hz, 1H), 2.50 (ddd, J = 17.5, 9.3, 3.2 Hz, 1H), 2.33 (ddt, J = 13.4, 10.6, 9.4 Hz, 1H), 2.11 – 2.00 (m, 1H), 1.40 (s, 9H).

^{13}C-NMR (101 MHz, $CDCl_3$) δ ppm: 173.12 (C), 170.08 (C), 150.94 (C), 135.09 (C), 128.56 (CH × 2), 128.41 (CH), 128.22 (CH × 2), 82.57 (C), 68.23 (CH_2), 59.40 (CH), 31.03 (CH_2), 27.80 (CH_3 × 3), 21.91 (CH_2).

LRMS (ESI) *m/z* $[M+H]^+$ calcd. for $C_{11}H_{12}FN_2O_3^+$ 320.15, found 320.25.

2.1.3.3 Preparation of (4-fluoro-2-methoxyphenyl)hydrazine hydrochloride (S6)

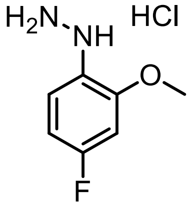

S6

2 N $NaNO_2$ (24.5 mL, 49 mmol) was added dropwise to an ice-cold solution of 4-fluoro-2-methoxy-aniline (4.66 g, 33 mmol, 1 eq) in 38% HCl (20 mL) and the reaction mixture was stirred at 0 °C for 30 min. 1 M Tin(II)Cl_2 × 2 H_2O in 38% HCl (99 mL, 99 mmol) was then added and the mixture was stirred at ambient temperature for 5 h. The precipitate was filtered off and dried to obtain the desired product as a brown solid (6.06 g, 31.04 mmol, 95%).

Yield 95%.

^{1}H-NMR (400 MHz, D_2O) δ ppm: 6.99 (dd, J = 8.7, 5.8 Hz, 1H), 6.77 (dd, J = 10.6, 2.7 Hz, 1H), 6.60 (td, J = 8.6, 2.7 Hz, 1H), 3.75 (s, 3H).

^{13}C-NMR (101 MHz, D_2O) δ ppm: 161.04 (d, J = 242.3 Hz, C-F), 152.24 (d, J = 10.5 Hz, C), 121.04 (d, J = 10.5 Hz, CH), 106.86 (d, J = 23.0 Hz, CH), 100.36 (d, J = 27.6 Hz, CH), 56.02 (CH_3).

^{19}F-NMR (376 MHz, D_2O) δ ppm: −114.24.

LRMS (ESI) *m/z* $[M+H-HCl]^+$ calcd. For $C_7H_8FNO^+$ 142.15, found 142.22.

2.1.3.4 Attempt to prepare *tert*-butyl 2-{[(benzyloxy)carbonyl]amino}-3-(5-fluoro-7-methoxy-1*H*-indol-3-yl)propanoate (S7)

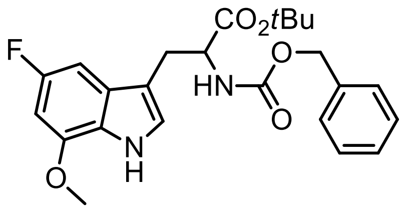

S7

A solution of **S6** (0.6 g, 3.1 mmol, 1 eq) and **S5** (1.61 g, 5 mmol, 1.65 eq) in acetic acid/water/EtOH (25/40/35, 16 mL) was refluxed for 2 days. H_2O (50 mL) was added and the mixture was extracted with CH_2Cl_2. The combined organic layers were washed with saturated $NaHCO_3$, dried and concentrated under reduced pressure. The residue was subjected to column chromatography (silica gel, hexane:EtOAc / 6:1) but none of the fractions contained the desired product.

2.1.4 Via borylation of protected 7-fluorotryptophan

2.1.4.1 Proposed synthetic route:

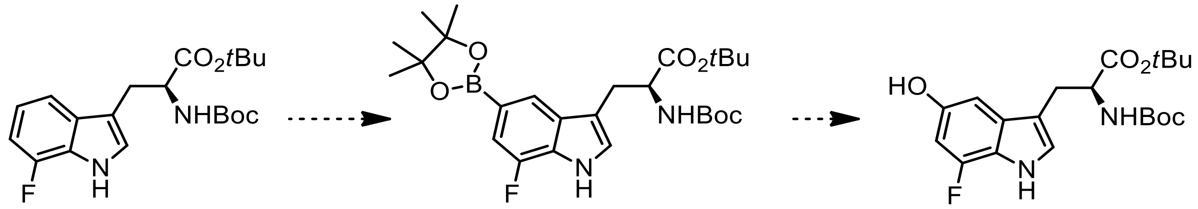

2.1.4.2 Attempt to prepare (*S*)-Boc-5-acetoxy-7-bis(4,4,5,5-tetramethyl-1,3,2-dioxaborolan-2-yl)-tryptophan-*t*Bu (S8)

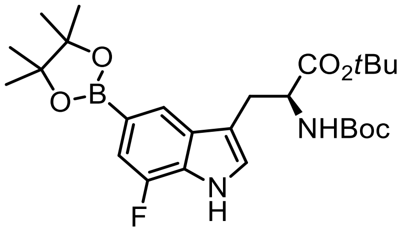

S8

Pinacolborane (0.98 mL, 0.59 g, 4.63 mmol, 5 eq) was added to a solution of Boc-7-FTrp-CO_2*t*Bu (0.35 g, 0.93 mmol, 1 eq), [Ir(cod)OMe]$_2$ (0.016 g, 0.023 mmol, 2.5 mol%) and 4,4'-di-*tert*-butyl-2,2'-bipyridine (0.013 g, 0.047 mmol, 5 mol%) in anhydrous THF (8 ml) and the reaction mixture was heated at 60 °C for 3 days. Additional [Ir(cod)OMe]$_2$ (0.016 g, 0.023 mmol, 2.5 mol%), 4,4'-di-*tert*-butyl-2,2'-bipyridine (0.013 g, 0.047 mmol, 5 mol%), pinacolborane (0.59 mL, 2.78 mmol, 3 eq) and B_2Pin_2 (0.7 g, 2.78 mmol, 3 eq) were added under argon and the mixture was stirred at 60 °C for another 7 days. Et_2O (15 mL) was added and the resulting solution was washed with 10% $NaHCO_3$ (3 × 10 mL) and brine (3 × 10 mL), dried and concentrated under reduced pressure. The residue was purified via column chromatography (silica gel, 0.1% Ca, Hexane:EtOAc / 3:1) and the purified intermediate was dissolved in acetic acid (3.5 mL). Pd(OAc)$_2$ (33 mg, 0.14 mmol, 5 mol%) was added and the mixture was heated at 30 °C for 20 h. Et_2O (35 mL) was added and the resulting solution was washed with 10% $NaHCO_3$ (3 × 20 mL) and brine (3 × 20 mL), dried, and concentrated under reduced pressure. Sequential column chromatography of the residue (silica gel, 0.1% Ca, hexane:EtOAc / 4:1) did not provide the desired product.

2.1.5 Via synthesis of *rac*-5-iodo-7-fluorotryptophan

2.1.5.1 Proposed synthetic route:

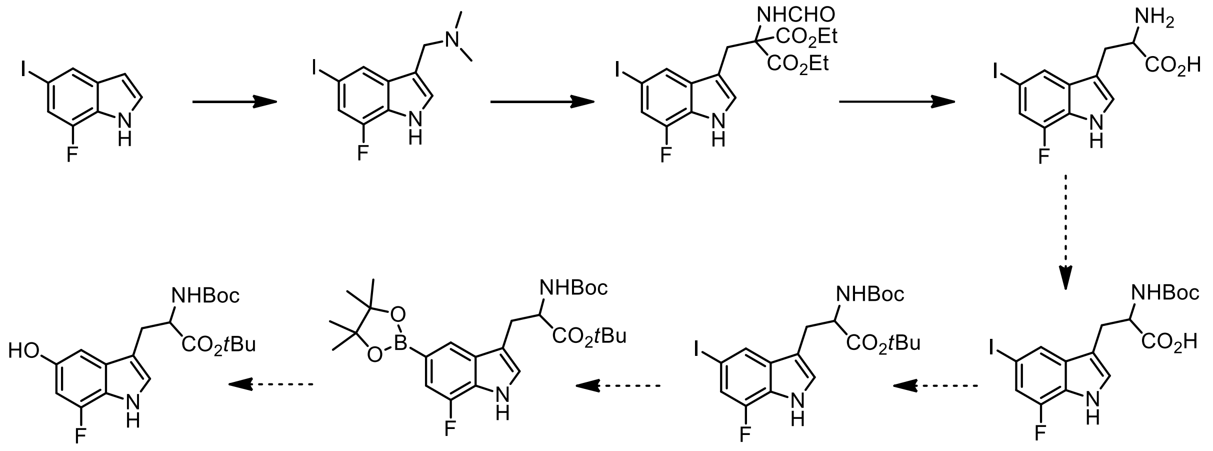

2.1.5.2 Preparation of 1-(7-fluoro-5-iodo-1*H*-indol-3-yl)-*N,N*-dimethylmethanamine (S9) [2]

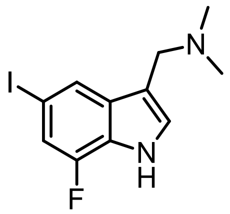

S9

37% Formaldehyde (0.35 mL, 4.75 mmol, 1.25 eq) and 40% Me_2NH (0.84 mL, 6.65 mmol, 1.75 eq) were added to an ice-cold solution of 5-iodo-7-fluoroindole (1.0 g, 3.8 mmol, 1 eq) in AcOH (4 mL). The mixture was stirred at ambient temperature overnight, cooled to 4 °C and basified to pH = 10 with 3 N NaOH. The mixture was extracted with EtOAc (4 × 15 mL), dried and concentrated under reduced pressure to obtain the desired product as a colorless solid (1.2 g, 3.7 mmol, 97%).

Yield 97%.

^{1}H-NMR [400 MHz, $(CD_3)_2SO$] δ ppm: 11.64 (s, 1H), 7.79 (d, J = 1.4 Hz, 1H), 7.30 (s, 1H), 7.23 (dd, J = 10.5, 1.4 Hz, 1H), 3.48 (s, 2H), 2.12 (s, 6H).

^{13}C-NMR [101 MHz, $(CD_3)_2SO$] δ ppm: 149.37 (d, J = 248.7 Hz, C-F), 133.89 (C), 126.96 (CH), 124.68 (d, J = 3.1 Hz, CH_2), 124.13 (d, J = 12.7 Hz, C), 114.79 – 114.29 (m, CH), 113.01 (C), 80.47 (d, J = 6.6 Hz, C-I), 54.60 (CH_2), 45.36 (CH_3 × 2).

^{19}F-NMR [376 MHz, $(CD_3)_2SO$] δ ppm: –130.88.

LRMS (ESI) *m/z* $[M+H]^+$ calcd. for $C_{11}H_{13}FIN_2^+$ 319.01, found 319.13.

2.1.5.3 Preparation of diethyl 2-((7-fluoro-5-iodo-1*H*-indol-3-yl)methyl)-2-formamidomalonate (S10)

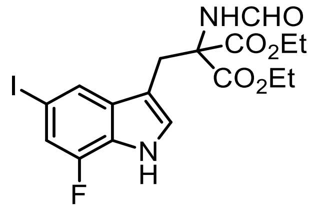

S10

A suspension of pulverized NaOH (50 mg, 1.25 mmol, 0.25 eq) in a solution of **S9** (1.6 g, 5 mmol, 1 eq) and diethyl formamidomalonate (1.12 g, 5.5 mmol, 1.1 eq) in anhydrous toluene (40 mL) was stirred under reflux for 3 d. EtOAc (50 mL) was added and the mixture was successively washed with 1 M $NaHSO_4$ (2 × 50 mL), 10% $NaHCO_3$ (2 × 50 mL) and brine (2 × 50 mL). The organic phase was dried and concentrated under reduced pressure. The residue was treated with Et_2O and the resulting colorless precipitate was recovered by filtration and purified via column chromatography (silica gel, Hexane:EtOAc / 1:1, v/v) to obtain the desired product as a colorless solid (1.5 g, 3.2 mmol, 64%).

Yield 64%.

^{1}H-NMR [400 MHz, $(CD_3)_2SO$] δ ppm: 8.63 (d, J = 1.6 200Hz, 1H), 8.02 (d, J = 1.5 Hz, 1H), 7.49 (d, J = 1.3 Hz, 1H), 7.23 (dd, J = 10.4, 1.3 Hz, 1H), 7.12 (d, J = 2.5 Hz, 1H), 4.21 – 4.02 (m, 4H), 3.58 (s, 2H), 1.18 (t, J = 7.1 Hz, 6H).

^{13}C-NMR [101 MHz, $(CD_3)_2SO$] δ ppm: 167.38 (C × 2), 161.45 (CH), 149.41 (d, J = 249.0 Hz, C-F), 134.09 (C), 127.23 (CH), 123.73 (d, J = 3.0 Hz, CH), 123.59 (d, J = 13.2 Hz, C), 114.50 (d, J = 18.9 Hz, CH), 108.18 (C), 80.71 (C-I), 66.54 (C), 62.46 (CH_2), 28.36 (CH_2), 14.23 (CH_3 × 2).

^{19}F-NMR [376 MHz, $(CD_3)_2SO$] δ ppm: –130.97.

LRMS (ESI) *m/z* $[M+H]^+$ calcd. For $C_{17}H_{19}FIN_2O_5^+$ 477.03, found 477.10.

2.1.5.4 Preparation of 2-amino-3-(7-fluoro-5-iodo-1*H*-indol-3-yl)propanoic acid (S11)

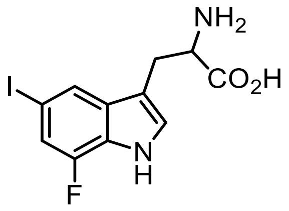

S11

A suspension of **S10** (1.4 g, 3 mmol, 1 eq) in 2.5 N NaOH (12.5 mL, 31.25 mmol) was heated at 125°C for 6 h. AcOH (1.78 mL, 1.87 g, 31.25 mmol) was added and the mixture was stirred at 125 °C for 2 h and at ambient temperature for 12 h. The precipitate was filtered off to afford the desired product as a colorless solid (0.73 g, 2.1 mmol, 70%)

Yield 70%.

^{1}H-NMR (400 MHz, D_2O) δ ppm: 8.01 (s, 0H), 7.44 (d, J = 5.4 Hz, 1H), 7.02 (dd, J = 11.4, 2.7 Hz, 2H), 3.37 (d, J = 3.8 Hz, 2H), 1.86 (s, 0H).

^{13}C-NMR (101 MHz, D_2O) δ ppm: 167.73 (C), 148.19 (d, J = 124.4 Hz, C-F), 127.48 (d, J = 4.5 Hz, CH), 123.20 (CH), 115.32 (d, J = 20.3 Hz, CH), 104.60 (C), 80.14 (d, J = 6.6 Hz, C-I), 66.53 (C), 29.41 (CH_2).

^{19}F-NMR (376 MHz, D_2O) δ ppm: –131.48.

LRMS (ESI) *m/z* $[M+H]^+$ calcd. For $C_{11}H_{11}FIN_2O2^+$ 348.98, found 349.05.

2.1.5.5 Preparation of Boc-5-iodo-7-fluorotryptophan (S12)

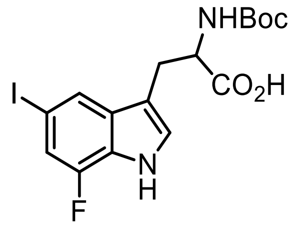

S12

$NaHCO_3$ (16.8 mg, 0.2 mmol, 1 eq) and a solution Boc_2O (66 mg, 0.3 mmol, 1.5 eq) in MeOH (0.5 mL) were added to a suspension of **S11** (75 mg, 0.2 mmol, 1 eq) in 1 N NaOH (0.3 mL, 1.5 eq). The mixture was diluted with H_2O (~1 ml) and MeOH (~1 mL) until all components were dissolved and stirred for 3 d. MeOH was removed under reduced pressure, the aqueous phase was washed with Et_2O (3 × 2 mL), acidified to pH = 2 with 1 N $NaHSO_4$ and extracted with Et_2O (3 × 10 mL). The organic phases were successively washed with 1 N $NaHSO_4$ (10 mL), H_2O (3 × 10 mL) and brine (2 × 10 mL), dried and concentrated under reduced pressure. The crude product was obtained as a slightly yellow residue (30 mg, 0.06 mmol, 30%).

This path proved to be inefficient for the planned follow-up steps, requiring large amounts of expensive 5-iodo-7-fluoroindole to produce sufficient amounts of Boc-5-I-7-FTrp-O*t*Bu.

2.1.6 Synthesis via reduction of 7-fluoroindole followed by hydroxylation with Fremy's salt

2.1.6.1 Proposed synthetic route:

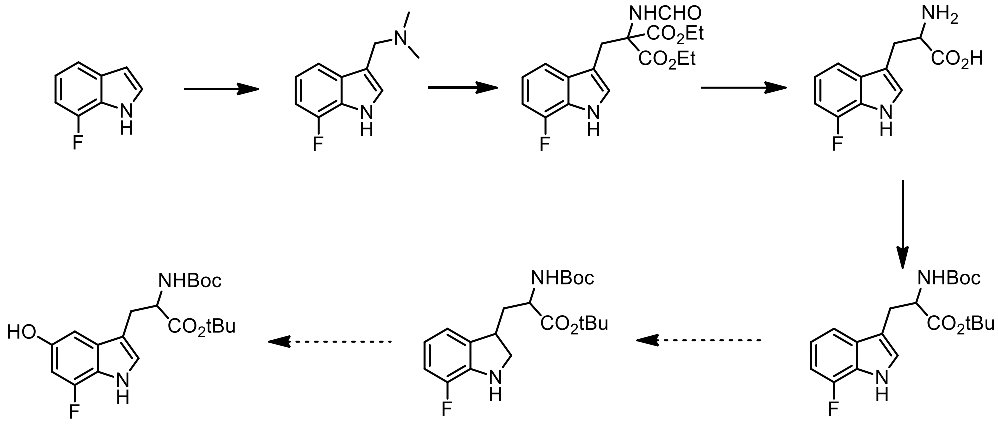

2.1.6.2 Preparation of 1-(7-fluoro-1*H*-indol-3-yl)-*N,N*-dimethylmethanamine (S13) [2]

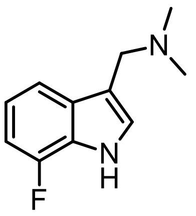

S13

37% Formaldehyde (1.37 mL, 18.5 mmol, 1.25 eq) and 40% Me_2NH (40%, 3.25 mL, 25.9 mmol, 1.75 eq) were added to an ice-cold solution of 7-fluoroindole (2 g, 14.8 mmol, 1 eq) in AcOH (15 mL). The mixture was allowed to reach ambient temperature and stirred for 16 h. Thereafter, it was cooled to 4 °C and basified to pH = 10–12 with 3 N NaOH. The resulting mixture was extracted with EtOAc (4 × 30 mL). The combined organic phases were washed with brine (15 mL), dried and concentrated under reduced pressure. The crude product was obtained as a sticky brown solid (2.2 g, 11.4 mmol, 77%).

Yield 77%.

^{1}H-NMR [400 MHz, $(CD_3)_2SO$] δ ppm: 7.51 – 7.45 (m, 1H), 7.39 (d, J = 2.4 Hz, 1H), 7.02 – 6.86 (m, 2H), 3.75 (s, 2H), 2.28 (s, 6H).

^{13}C-NMR [101 MHz, $(CD_3)_2SO$] δ ppm: 149.69 (d, J = 242.7 Hz, C-F), 131.96 (C), 127.12 (CH), 124.48 (d, J = 13.1 Hz, C), 119.44 (d, J = 6.1 Hz, CH), 115.71 (d, J = 3.2 Hz, CH), 111.11 (C), 106.39 (d, J = 16.0 Hz, CH), 53.76 (CH_2), 44.33 (CH_3 × 2).

^{19}F-NMR [376 MHz, $(CD_3)_2SO$] δ ppm: –133.71.

LRMS (ESI) *m/z* $[M+H]^+$ calcd. For $C_{11}H_{14}FN_2^+$ 193.11, found 193.32.

2.1.6.3 Preparation of diethyl 2-[(7-fluoro-1*H*-indol-3-yl)methyl]-2-formamidomalonate (S14)

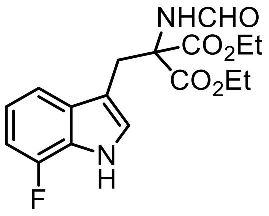

S14

A suspension of pulverized NaOH (62 mg, 1.55 mmol, 0.25 eq) in a solution of **S13** (1.2 g, 6.2 mmol, 1 eq) and diethyl formamidomalonate (1.4 g, 6.82 mmol, 1.1 eq) in anhydrous toluene (50 mL) was stirred under reflux for 3 d. EtOAc (55 mL) was added and the mixture was successively washed with 1 M $NaHSO_4$ (2 × 55 mL), 10% $NaHCO_3$ (2 × 55 mL) and brine (2 × 55 mL), dried and concentrated under reduced pressure. The residue was purified by column chromatography (silica gel, hexane:EtOAc / 1:1, v/v) to obtain the desired product as a colorless solid (0.95 g, 2.7 mmol, 44%).

Yield 44%.

^{1}H-NMR (400 MHz, $CDCl_3$) δ ppm: 8.55 (s, 1H), 8.20 (d, J = 1.4 Hz, 1H), 7.32 – 7.25 (m, 1H), 7.05 – 6.96 (m, 2H), 6.92 – 6.84 (m, 2H), 4.36 – 4.12 (m, 4H), 3.89 (s, 2H), 1.29 (t, J = 7.1 Hz, 6H).

^{13}C-NMR (101 MHz, $CDCl_3$) δ ppm: 167.36 (C), 160.18 (CH), 149.60 (d, J = 243.7 Hz, C-F), 131.68 (C), 124.27 (d, J = 13.3 Hz, C), 124.13 (CH), 119.80 (d, J = 6.2 Hz, C), 114.60 (d, J = 3.3 Hz, CH), 109.75 (C), 106.87 (d, J = 16.0 Hz, CH), 66.67 (C), 62.86 (CH_2), 28.26 (CH_2), 13.93 (CH_3 × 2).

^{19}F-NMR (376 MHz, $CDCl_3$) δ ppm: −135.45.

HRMS (ESI) *m/z* $[M+H]^+$ calcd. for $C_{17}H_{20}FN_2O_5^+$ 351.13563, found 351.13508.

m/z $[M+Na]^+$ calcd. for $C_{17}H_{19}FN_2NaO_5^+$ 373.11757, found 373.11702.

m/z $[M+K]^+$ calcd. for $C_{17}H_{19}FKN_2O_5^+$ 389.09151, found 389.09094.

2.1.6.4 Preparation of 7-fluorotryptophan (S15)

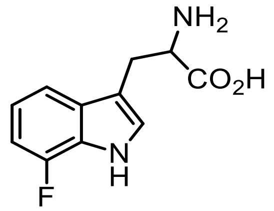

S15

0.75 N NaOH (5.2 mL) was added to a solution of **S14** (0.4 g, 1.14 mmol, 1 eq) in THF (10.4 mL). The mixture was stirred at ambient temperature for 24 h, acidified with acetic acid (2.6 mL) and refluxed for 24 h. The reaction mixture was concentrated under reduced pressure, the residue was taken up in 3 N HCl (5.2 mL) and the resulting solution was refluxed for another 24 h. The mixture was cooled to ambient temperature, basified to pH = 6.0 with 2 M KOH and concentrated under reduced pressure. The residue was taken up in H_2O and the precipitate was filtered and dried under reduced pressure to obtain the desired product as a colorless solid (0.21 g, 0.94 mmol, 82%).

Yield 82%.

^{1}H-NMR (400 MHz, CD_3OD) δ ppm: 7.43 (dt, J = 8.0, 0.8 Hz, 1H), 7.29 (s, 1H), 7.03 (td, J = 7.9, 4.7 Hz, 1H), 6.90 (ddd, J = 11.4, 7.8, 0.8 Hz, 1H), 4.28 (dd, J = 7.8, 5.0 Hz, 1H), 3.55 – 3.47 (m, 1H), 3.44 – 3.34 (m, 1H).

^{13}C-NMR (101 MHz, CD_3OD) δ ppm: 170.17 (C), 149.87 (d, J = 243.3 Hz, C-F), 130.90 (d, J = 5.6 Hz, C), 125.23 (CH), 124.96 (d, J = 13.6 Hz, C), 119.24 (d, J = 6.3 Hz, CH), 113.69 (d, J = 3.4 Hz, CH), 107.58 (C), 106.09 (d, J = 16.5 Hz, CH), 53.05 (C), 26.13 (CH_2).

^{19}F-NMR (376 MHz, CD_3OD) δ ppm: –136.97.

LRMS (ESI) *m/z* $[M+H]^+$ calcd. for $C_{11}H_{12}FN_2O_2^+$ 223.22, found 223.25.

2.1.6.5 Preparation of 2-[(*tert*-butoxycarbonyl)amino]-3-(7-fluoro-1*H*-indol-3-yl)propanoic acid (S16) [3]

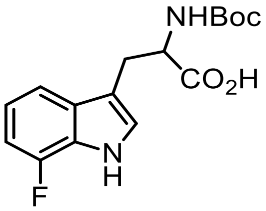

S16

$NaHCO_3$ (0.37 g, 4.5 mmol, 1 eq) and a solution of Boc_2O (1.47 g, 6.75 mmol, 1.5 eq) in MeOH (10 mL) were added to a suspension of **S15** (1.0 g, 4.5 mmol, 1 eq) in 1 N NaOH (6.75 mL, 1.5 eq). The mixture was diluted with H_2O (~20 mL) and MeOH (~20 mL) until all components were dissolved and stirred for 3 d. MeOH was removed under reduced pressure, the aqueous phase was washed with Et_2O (3 × 20 mL), acidified to pH = 2 with 1 N $NaHSO_4$ and extracted with Et_2O (3 × 30 mL). The combined organic phases were successively washed with 1 N $NaHSO_4$ (20 mL), H_2O (3 × 20 mL) and brine (2 × 20 mL), and concentrated under reduced pressure to obtain the desired product as a colorless foam (1.13 g, 3.5 mmol, 77%).

Yield 77%.

^{1}H-NMR (400 MHz, $CDCl_3$) δ ppm: 7.37 (d, J = 7.9 Hz, 1H), 7.01 (ddd, J = 16.0, 9.9, 6.0 Hz, 2H), 6.91 (dd, J = 11.1, 7.6 Hz, 1H), 5.15 (d, J = 8.0 Hz, 1H), 4.70 (q, J = 6.1 Hz, 1H), 3.33 (qd, J = 14.9, 5.6 Hz, 2H), 1.44 (s, 6H), 1.31 (s, 3H).

^{13}C-NMR (101 MHz, $CDCl_3$) δ ppm: 176.49 (C), 155.64 (C), 149.62 (d, J = 243.6 Hz, C-F), 131.51 (d, J = 3.8 Hz, C), 124.51 (d, J = 13.4 Hz, C), 123.79 (CH), 119.81 (CH), 114.56 (CH), 110.80 (C), 106.94 (d, J = 16.0 Hz, CH), 80.42 (C), 54.18 (CH), 28.30 (CH_3 × 3), 27.68 (CH_2).

^{19}F-NMR (376 MHz, $CDCl_3$) δ ppm: −135.25.

LRMS (ESI) *m/z* $[M+Na]^+$ calcd. for $C_{16}H_{19}FN_2NaO_4^+$ 345.12, found 345.07.

m/z $[2M+H]^+$ calcd. for $C_{32}H_{98}F_2N_4O_8^+$ 645.27, found 645.21.

m/z $[2M+Na]^+$ calcd. for $C_{32}H_{38}F_2N_4NaO_8^+$ 667.26, found 667.17.

2.1.6.6 Preparation of *tert*-butyl 2-((*tert*-butoxycarbonyl)amino)-3-(7-fluoro-1*H*-indol-3-yl)propanoate (S17) [4]

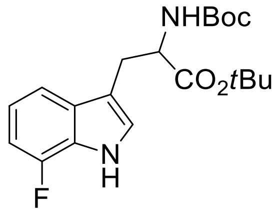

S17

A solution of **S16** (1.7 g, 5.3 mmol, 1 eq) and *tert*-butyl 2,2,2-trichloroacetimidate (2.31 g, 10.6 mmol, 2 eq) in anhydrous CH_2Cl_2 (26.5 mL) was stirred at 40 °C for 3 d. Approximately half of the solvent was removed under reduced pressure and the mixture was stored at -20 °C for 12 h. The precipitate was filtered off and washed with cold CH_2Cl_2 (15 mL). The filtrate was successively washed with 10% $NaHCO_3$ (15 mL), 1 N $NaHSO_4$ (15 mL), H_2O (15 mL) and brine (15 mL), dried and concentrated under reduced pressure. The residue was purified by normal-phase column chromatography (silica gel, hexane:EtOAc / gradient, 4:1 to 2:1, v/v) and RP flash chromatography (CHROMABOND Sorbent C_{18}, H_2O:MeCN, gradient, 20% to 100% MeCN). The residue was treated with Et_2O and the resulting precipitate filtered off and dried to obtain the desired product as a colorless solid (1.1 g, 2.9 mmol, 55%).

Yield 55%.

^{1}H-NMR [400 MHz, $CDCl_3$ + 10% $(CD_3)_2SO$] δ ppm: 7.22 – 7.12 (m, 1H), 6.89 (d, J = 9.7 Hz, 1H), 6.79 (dq, J = 8.2, 5.3, 3.6 Hz, 1H), 6.67 (ddd, J = 10.8, 7.9, 2.4 Hz, 1H), 5.09 (d, J = 8.6 Hz, 1H), 4.29 (q, J = 7.0 Hz, 1H), 3.03 (q, J = 9.4, 8.7 Hz, 2H), 1.32 – 1.07 (m, 18H).

^{13}C-NMR [101 MHz, $CDCl_3$ + 10% $(CD_3)_2SO$] δ ppm: 171.16 (C), 155.11 (C), 149.60 (d, J = 243.3 Hz, C-F), 131.58 (C), 124.34 (d, J = 13.4 Hz, C), 124.11 (CH), 120.15 – 117.43 (m, CH), 114.59 (CH), 112.21 – 109.15 (m, C), 106.07 (d, J = 16.3 Hz, CH), 81.48 (C), 79.24 (C), 54.62 (CH), 28.23 (CH_3 × 3), 27.85 (CH_2), 27.80 (CH_3 × 3).

^{19}F-NMR [376 MHz, $CDCl_3$ + 10% $(CD_3)_2SO$] δ ppm: –134.53.

LRMS (ESI) *m/z* $[M+H]^+$ calcd. for $C_{20}H_{28}FN_2O_4^+$ 379.20, found 379.20.

2.1.6.7 Attempt to prepare *tert*-butyl 2-[(*tert*-butoxycarbonyl)amino]-3-(7-fluoroindolin-3-yl)propanoate (S18) [5]

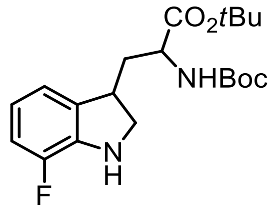

S18

$NaBH_3CN$ (33 mg, 0.52 mmol, 2 eq) was added in portions to a solution of **S17** (0.1 g, 0.26 mmol, 1 eq) in THF (0.75 mL) and AcOH (0.1 mL) and the mixture was stirred for 1 h. More $NaBH_3CN$ (132 mg, 2.08 mmol, 8 eq) and AcOH (0.1 mL) were then added and the mixture was stirred for another 1 h. The solvents were partially removed under reduced pressure and 2 N NaOH (5 mL) was added. The mixture was extracted with CH_2Cl_2 (3 × 5 mL), the organic fraction was washed with brine (5 mL), dried and concentrated under reduced pressure to afford a gummy residue consisting of unchanged **S17**.

2.1.7 Synthesis via Ni-BPB-Trp-complex with Bpin-leaving group

2.1.7.1 Proposed synthetic route:

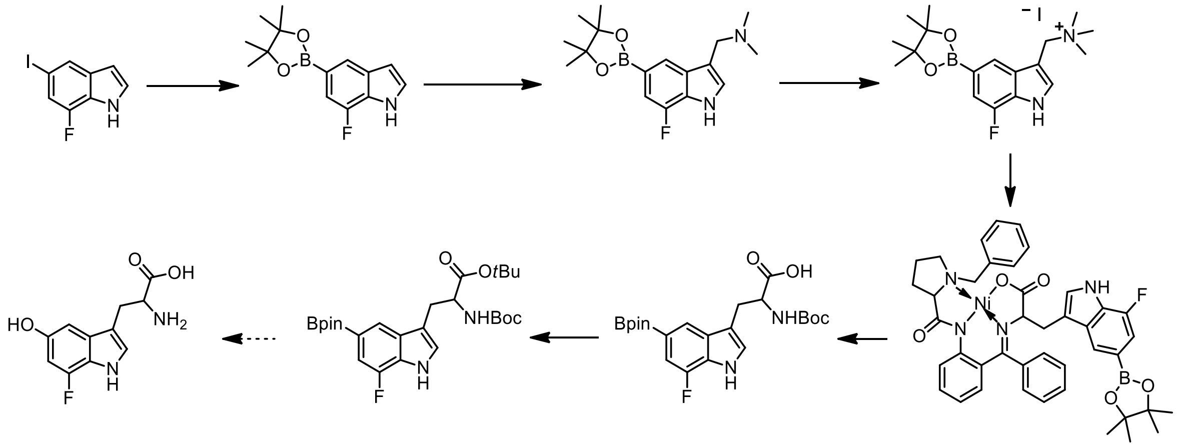

2.1.7.2 Preparation of 7-fluoro-5-(4,4,5,5-tetramethyl-1,3,2-dioxaborolan-2-yl)-1*H*-indole (S19) [6]

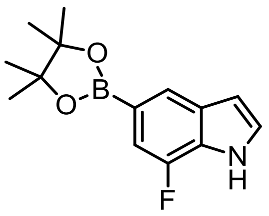

S19

A suspension of KOAc (2 g, 20 mmol, 2.1 eq) in a solution of 7-fluoro-5-iodoindole (2.5 g, 9.6 mmol, 1 eq), bis(pinacolato)diboron (3 g, 11.5 mmol, 1.2 eq) and Pd(dppf)Cl_2 (0.7 g, 0.96 mmol, 0.1 eq) in anhydrous DMF (30 mL) was stirred at 75 °C overnight and 90 °C for 3 h. The solvent was removed under reduced pressure and the residue was taken up in Et_2O (50 mL). The resulting suspension was washed with H_2O (3 × 50 mL) and brine (3 × 50 mL), dried and concentrated under reduced pressure. The residue was purified by column chromatography (silica gel, 0.1% Ca, hexane:EtOAc / 4:1) to obtain the desired product as a grey solid (2 g, 7.6 mmol, 80%).

Yield 80%.

^{1}H-NMR (400 MHz, $CDCl_3$) δ ppm: 8.50 (s, 1H), 7.96 (q, J = 0.9 Hz, 1H), 7.38 – 7.21 (m, 2H), 6.61 (td, J = 3.4, 2.1 Hz, 1H), 1.28 (s, 12H).

^{13}C-NMR (101 MHz, $CDCl_3$) δ ppm: 149.34 (d, J = 244.2 Hz, C-F), 131.51 (d, J = 5.1 Hz, C), 126.33 (d, J = 13.6 Hz, C), 124.60 (d, J = 52.5 Hz, CH), 111.79 (d, J = 14.3 Hz, CH), 106.31 – 101.69 (m, CH), 83.62 (d, J = 17.8 Hz, C × 2), 25.03 (CH_3 × 4), C-B was not observed.

^{19}F-NMR (376 MHz, $CDCl_3$) δ ppm: −136.54.

LRMS (ESI) *m/z* [M+H]$^+$ calcd. For $C_{14}H_{18}BFNO_2^+$ 262.14, found 262.26.

m/z [M+K]$^+$ calcd. For $C_{14}H_{17}BFNNaO_2^+$ 300.10, found 300.37.

2.1.7.3 Preparation of 1-[7-fluoro-5-(4,4,5,5-tetramethyl-1,3,2-dioxaborolan-2-yl)-1*H*-indol-3-yl]-*N,N*-dimethylmethanamine (S20) [2]

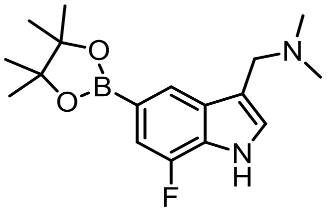

S20

37% Formaldehyde (1 mL, 13.13 mmol, 1.25 eq) and 40% Me_2NH (2.33 mL, 18.4 mmol, 1.75 eq) were added to an ice-cold solution of **S19** (2.73 g, 10.5 mmol, 1 eq) in AcOH (25 mL). The mixture was allowed to warm to ambient temperature, stirred for 4 h, cooled to 4 °C and basified to pH = 10 with 3 N NaOH. The mixture was extracted with EtOAc (5 × 20 mL) and the combined organic phases were dried and concentrated under reduced pressure. The crude product was obtained as an oily colorless solid (2.9 g, 9.1 mmol, 86%) that was used without purification.

Yield 86%.

^{1}H-NMR (400 MHz, CD_3OD) δ ppm: 7.93 (s, 1H), 7.30 (s, 1H), 7.19 (d, J = 11.8 Hz, 1H), 4.88 (s, 4H), 3.74 (s, 2H), 2.32 (s, 7H), 1.37 (s, 12H), 1.22 (s, 3H).

^{13}C-NMR (101 MHz, CD_3OD) δ ppm: 149.37 (d, J = 244.0 Hz, C-F), 131.62 (d, J = 5.5 Hz, C), 126.00 (CH, 122.14 (CH), 111.68 (C), 110.56 (d, J = 14.6 Hz, CH), 83.52 (C × 2), 53.06 (CH_2), 43.54 (CH_3 × 2), 23.83 (CH_3 × 4), C-B was not observed.

^{19}F-NMR (376 MHz, CD_3OD) δ ppm: –138.30.

LRMS (ESI) *m/z* $[M+H]^+$ calcd. for $C_{17}H_{25}BFN_2O_2^+$ 319.20, found 319.18.

2.1.7.4 Preparation of 1-[7-fluoro-5-(4,4,5,5-tetramethyl-1,3,2-dioxaborolan-2-yl)-1*H*-indol-3-yl]-*N,N,N*-trimethylmethanaminium iodide (S21)

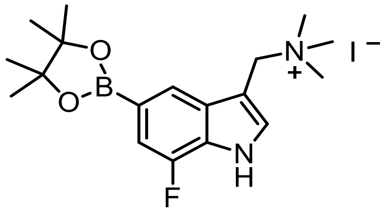

S21

Iodomethane (0.78 mL, 12.5 mmol, 5 eq) was added to an ice-cold solution of **S20** (0.785 g, 2.5 mmol, 1 eq) in anhydrous EtOH (70 mL), the reaction mixture was allowed to warm to ambient temperature and stirred for 3 d. The mixture was concentrated under reduced pressure and the residue was triturated with pentane until a solid precipitate formed. The precipitate was filtered off, rinsed with pentane and dried under reduced pressure. The crude product was obtained as a grey solid (0.98 g, 2.1 mmol, 84%).

Yield 84%.

^{1}H-NMR [400 MHz, $(CD_3)_2SO$] δ ppm: 8.15 (s, 1H), 8.05 (s, 1H), 7.82 (dd, J = 13.4, 2.7 Hz, 1H), 7.21 (dd, J = 11.6, 5.1 Hz, 1H), 4.90 (s, 1H), 4.75 (s, 1H), 3.35 (s, 4H), 3.13 (s, 3H), 3.06 (s, 5H), 1.32 (d, J = 4.2 Hz, 12H).

^{13}C-NMR [101 MHz, $(CD_3)_2SO$] δ ppm: 149.49 (d, J = 241.4 Hz, C-F), 132.30 (d, J = 27.2 Hz, C), 126.67 (d, J = 6.7 Hz, C), 122.56 (d, J = 2.2 Hz, CH), 111.77 (d, J = 13.8 Hz, CH), 104.01 (d, J = 22.2 Hz, C), 84.18 (d, J = 3.6 Hz, C × 2), 60.27 (CH_2), 58.71 (CH_2), 54.94 (CH_3 × 3), 51.71 (CH_3 × 3), 25.21 (CH_3 × 4), C-B was not observed.

^{19}F-NMR [376 MHz, $(CD_3)_2SO$] δ ppm: –133.96.

LRMS (ESI) *m/z* $[M\text{-}I]^+$ calcd. For $C_{18}H_{27}BFN_2O_2^+$ 333.21, found 333.20.

2.1.7.5 Preparation of (*S,S*)-Ni(II)-BPB-5-Bpin-7-FTrp (S22) [7]

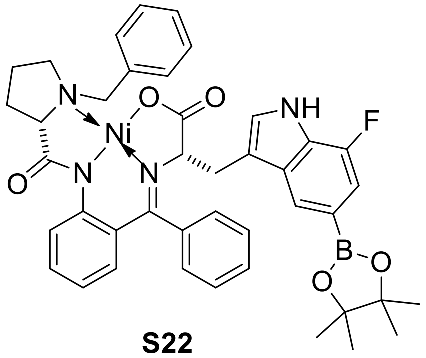

S22

Powdered NaOH (0.084 g, 2.09 mmol, 2.5 eq) was added to a solution of **S21** (0.385 g, 0.837 mmol, 1 eq) and (*S*)-Ni-BPB-Gly (0.392 g, 0.837 mmol, 1 eq) in anhydrous MeCN (40 mL) and the reaction mixture was stirred at 60°C overnight. Additional NaOH powder (0.084 g, 2.09 mmol, 2.5 eq) was added and the mixture was stirred at 60°C for another 5 h and at ambient temperature for 3 d. Additional NaOH powder (0.084 g, 2.09 mmol, 2.5 eq) was then added and the mixture was stirred at 60°C overnight. Saturated NH_4Cl (150 mL) was added, the mixture was extracted with EtOAc (3 × 40 mL), and the combined organic fractions were dried and concentrated under reduced pressure. The residue was purified via normal-phase column chromatography (silica gel, $CHCl_3$:acetone / 3:1) and RP flash chromatography (CHROMABOND Sorbent C_{18}, water:MeCN, gradient) to obtain the desired product as a red solid (200 mg, 0.26 mmol, 31%).

Yield 31%.

^{1}H-NMR (400 MHz, $CDCl_3$) δ ppm: 9.19 (s, 1H), 8.21 (t, J = 7.2 Hz, 1H), 7.99 (d, J = 7.1 Hz, 2H), 7.61 (d, J = 5.1 Hz, 1H), 7.47 (dq, J = 31.7, 7.6, 6.9 Hz, 2H), 7.35 – 7.23 (m, 5H), 7.20 – 7.08 (m, 2H), 7.06 (d, J = 4.7 Hz, 1H), 6.84 (d, J = 7.5 Hz, 1H), 6.67 (d, J = 4.4 Hz, 2H), 4.27 (dd, J = 13.8, 4.9 Hz, 1H), 3.50 – 3.35 (m, 2H), 3.24 (td, J = 11.9, 9.8, 5.8 Hz, 2H), 3.01 (ddd, J = 10.2, 6.6, 3.0 Hz, 1H), 2.40 (q, J = 9.7 Hz, 1H), 2.27 – 2.11 (m, 1H), 1.94 – 1.82 (m, 2H), 1.76 – 1.58 (m, 1H), 1.28 (dd, J = 6.5, 3.5 Hz, 8H), 1.19 (d, J = 5.9 Hz, 4H).

^{13}C-NMR (101 MHz, $CDCl_3$) δ ppm: 180.00 (C), 179.28 (C), 171.04 (C), 149.42 (d, J = 244.5 Hz, C-F), 142.95 (C), 133.89 (C), 133.42 (CH), 133.18 (C), 132.09 (CH), 131.92 (C), 131.53 (CH), 129.63 (CH), 128.79 (d, J = 5.8 Hz, CH), 128.01 (CH), 127.31 (CH), 126.50 (C), 125.11 (CH), 123.72 (CH), 122.86 (CH), 120.41 (CH), 111.88 (d, J = 14.0 Hz, CH), 111.27 (C), 83.48 (C × 2), 70.31 (CH), 63.06 (CH_2), 56.98

(CH_2), 30.82 (CH_2), 30.34 (CH_2), 24.80 (d, J = 16.3 Hz, CH_3 × 4), 22.99 (CH_2), C-B was not observed.

^{19}F-NMR (376 MHz, $CDCl_3$) δ ppm: −136.78.

LRMS (ESI) *m/z* [M+H]$^+$ calcd. for $C_{42}H_{43}BFN_4NiO_5^+$ 771.27, found 771.35.

2.1.7.6 Preparation of (*S*)-2-[(*tert*-butoxycarbonyl)amino]-3-[7-fluoro-5-(4,4,5,5-tetramethyl-1,3,2-dioxaborolan-2-yl)-1*H*-indol-3-yl]propanoic acid (S23) [8]

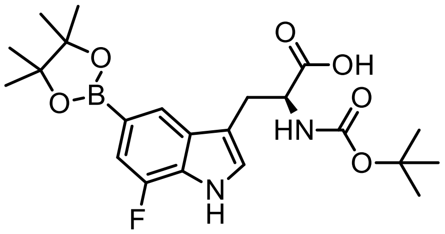

S23

A solution of DTPA (0.43 g, 1.1 mmol, 5 eq) in 25% Et_4NOH (25%, 1.27 mL, 2.2 mmol, 10 eq) was added to a solution of **S22** (0.17 g, 0.22 mmol, 1 eq) in MeOH (10 mL) and the mixture was stirred at 85 °C for 24 h. MeOH was removed under reduced pressure (oily precipitate in solution) and the residual suspension was basified to pH = 9 with 1 M NaOH. The precipitate was filtered off, washed with cold H_2O, and the combined aqueous phases were added to 10% $NaHCO_3$ (2 mL). Boc_2O (0.16 g, 0.733 mmol, 3.33 eq) followed by MeOH were added until the mixture was homogeneous. The mixture was stirred for 3 d at ambient temperature and MeOH was removed under reduced pressure. *N,N*-dimethyltrimethylendiamine (0.063 g, 0.077 mL, 0.62 mmol, 2.8 eq) was added, the mixture was stirred for 30 min, acidified to pH = 2 with solid $NaHSO_4$ and extracted with Et_2O (2 × 10 mL). The combined organic phases were washed with 1 M $NaHSO_4$ (×3), H_2O (×3) and brine (×2), dried and concentrated under reduced pressure to afford the desired product as a colorless solid (0.044 g, 0.1 mmol, 45%).

Yield 45%.

^{1}H-NMR [400 MHz, $(CD_3)_2SO$] δ ppm: 7.91 (d, J = 5.7 Hz, 2H), 7.34 – 7.15 (m, 2H), 7.07 (dd, J = 11.9, 7.3 Hz, 1H), 4.15 (dddd, J = 20.2, 10.0, 8.2, 4.6 Hz, 1H), 3.16 (dd, J = 14.5, 4.7 Hz, 1H), 2.97 (dd, J = 14.6, 9.8 Hz, 1H), 1.39 (s, 1H), 1.32 (s, 12H), 1.19 (s, 2H).

^{13}C-NMR [101 MHz, $(CD_3)_2SO$] δ ppm: 54.73 (CH), 28.62 (CH_3 × 4), 25.15 (CH_3 × 4).

^{19}F-NMR [376 MHz, $(CD_3)_2SO$] δ ppm: –135.61.

LRMS (ESI) *m/z* [M *t*Bu+2H]$^+$ calcd. for $C_{18}H_{23}BFN_2O_6^+$ 393.16, found 393.25.

m/z [M+H]$^+$ calcd. for $C_{22}H_{31}BFN_2O_6^+$ 449.23, found 449.33.

m/z [M+Na]$^+$ calcd. for $C_{22}H_{30}BFN_2NaO_6^+$ 471.21, found 471.27.

HRMS (ESI) *m/z* [M+Na]$^+$ calcd. for $C_{22}H_{30}BFN_2NaO_6^+$ 471.20732, found 471.20753.

2.1.7.7 Preparation of *tert*-butyl 2-[(*tert*-butoxycarbonyl)amino]-3-[7-fluoro-5-(4,4,5,5-tetramethyl-1,3,2-dioxaborolan-2-yl)-1*H*-indol-3-yl]propanoate (S24)

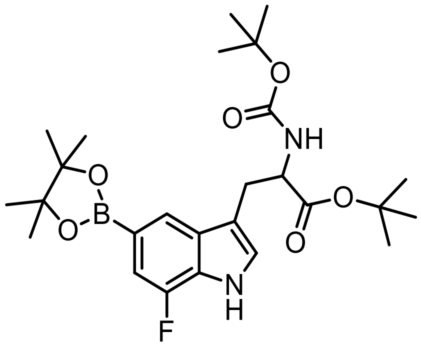

S24

A solution of **S23** (40 mg, 0.09 mmol, 1 eq) and *tert*-butyl 2,2,2-trichloroacetimidate (38 mg, 31 µL, 0.18 mmol, 2 eq) in anhydrous CH_2Cl_2 (3 mL) was stirred at 40 °C for 1 d. Approximately half of the solvent was removed under reduced pressure and the mixture was stored at –20 °C for 3 d. The precipitate was filtered off and washed with cold CH_2Cl_2 (8 mL). The filtrate was washed with 10% $NaHCO_3$ (8 mL), 1 N $NaHSO_4$ (8 mL), H_2O (8 mL) and brine (8 mL), dried and concentrated under reduced pressure. The residue was purified by column chromatography (silica gel, hexane:EtOAc / 5:1, v/v) to obtain the desired product as a yellowish solid (24 mg, 0.05 mmol, 80%).

This path proved to be too inefficient for the planned follow-up steps, requiring large amounts of expensive starting material to produce sufficient amounts of Boc-5-Bpin-7-FTrp-O*t*Bu.

2.1.8 Synthesis via Ni-BPB-Trp-complex with Boc-leaving group

2.1.8.1 Proposed synthetic route:

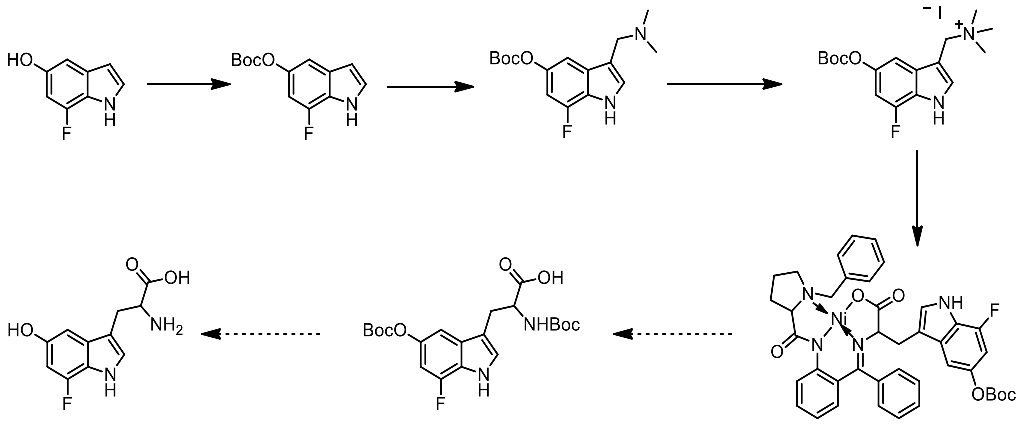

2.1.8.2 Preparation of *tert*-butyl (7-fluoro-1*H*-indol-5-yl) carbonate (S25)

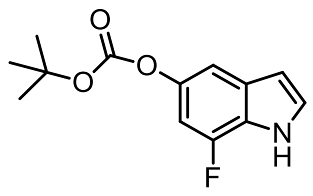

S25

A solution of 5-hydroxy-7-fluoroindole (63 mg, 0.41 mmol, 1 eq), Boc_2O (0.2 g, 0.9 mmol, 2.2 eq) and $Zn(OAc)_2 \cdot 2\ H_2O$ (20 mg, 0.09 mmol, 0.22 eq) in CH_2Cl_2 (0.4 mL) was stirred at 50 °C for 1 d. The mixture was diluted with H_2O and extracted with CH_2Cl_2 (3 × 10 mL). The combined organic layers were dried and concentrated under reduced pressure. The residue was purified by column chromatography (hexane/EtOAc: 4/1) to obtain the desired product as a grey solid (82 mg, 0.33 mmol, 80%).

Yield 80%.

^{1}H-NMR (400 MHz, $CDCl_3$) δ ppm: 8.41 (s, 1H), 7.24 (dd, J = 2.0, 0.7 Hz, 1H), 7.23 – 7.21 (m, 1H), 6.82 (dd, J = 11.2, 2.0 Hz, 1H), 6.57 (td, J = 3.3, 2.1 Hz, 1H), 1.60 (s, 9H).

^{13}C-NMR (101 MHz, $CDCl_3$) δ ppm: 152.62 (C), 149.77 (C), 145.75 (d, J = 316.1 Hz, C-F), 130.43 (d, J = 6.1 Hz, C), 125.98 (CH), 122.21 (d, J = 13.1 Hz, C), 108.44 (d, J = 3.9 Hz, CH), 103.75 (d, J = 2.6 Hz, CH), 102.21 (d, J = 19.6 Hz, C), 83.50 (C), 27.74 (CH_3 × 3).

^{19}F-NMR (376 MHz, $CDCl_3$) δ ppm: –132.83.

LRMS (ESI) *m/z* $[M\text{-}tBu+H]^+$ calcd. for $C_9H_7FNO_3^+$ 196.04, found 196.24.

m/z [M+H]$^+$ calcd. for $C_{13}H_{15}FNO_3^+$ 252.10, found 252.22.

m/z [M+MeOH]$^+$ calcd. for $C_{14}H_{18}FNO_4^+$ 283.12, found 283.19.

2.1.8.3 Preparation of *tert*-butyl {3-[(dimethylamino)methyl]-7-fluoro-1*H*-indol-5-yl} carbonate (S26) [2]

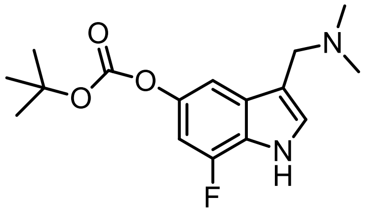

S26

37% Formaldehyde (0.17 mL, 2.26 mmol, 1.25 eq) and 40% Me_2NH (0.4 mL, 3.17 mmol, 1.75 eq) were added to an ice-cold solution of **S25** (0.454 g, 1.81 mmol, 1 eq) in AcOH (2 mL). The mixture was stirred at ambient temperature overnight, cooled to 4 °C and basified to pH = 10 with 3 N NaOH. The mixture was extracted with EtOAc (4 × 27 mL) and the combined organic phases were dried and concentrated under reduced pressure to obtain the crude product as a purple semisolid (0.537 g, 1.74 mmol, 96%).

Yield 96%.

^{1}H-NMR (400 MHz, CD_3OD) δ ppm: 7.33 (s, 1H), 7.24 (d, J = 2.0 Hz, 1H), 6.75 (dd, J = 11.4, 2.0 Hz, 1H), 3.66 (s, 2H), 2.28 (s, 6H), 1.56 (s, 9H).

^{13}C-NMR (101 MHz, CD_3OD) δ ppm: 152.79 (C), 148.64 (d, J = 245.3 Hz, C-F), 143.94 (d, J = 9.0 Hz, C), 130.53 (d, J = 6.6 Hz, C), 127.14 (CH), 122.38 (d, J = 13.2 Hz, C), 111.74 (C), 106.35 (d, J = 3.9 Hz, CH), 101.14 (d, J = 19.9 Hz, CH), 82.72 (C), 53.04 (CH_2), 43.54 (CH_3 × 2), 26.55 (CH_3 × 3).

^{19}F-NMR (376 MHz, CD_3OD) δ ppm: −134.87.

LRMS (ESI) *m/z* $[M\text{-}tBu+H]^+$ calcd. for $C_{12}H_{14}FN_2O_3^+$ 253.25, found 253.29.

m/z $[M+H]^+$ calcd. for $C_{16}H_{22}FN_2O_3^+$ 309.16, found 309.20.

2.1.8.4 Preparation of 1-{5-[(*tert*-butoxycarbonyl)oxy]-7-fluoro-1*H*-indol-3-yl}-*N,N,N*-trimethylmethanaminium iodide (S27)

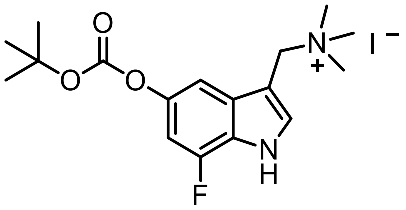

S27

Iodomethane (0.54 mL, 8.7 mmol, 5 eq) was added to an ice-cold solution of **S26** (0.537 g, 1.74 mmol, 1 eq) in anhydrous EtOH (10 mL) and the reaction mixture was stored at –20 °C overnight. The mixture was concentrated under reduced pressure and the residue was triturated with Et_2O until a solid precipitate formed. The precipitate was filtered off, washed with Et_2O and dried under reduced pressure to obtain the desired product as a colorless solid (0.724 g, 1.61 mmol, 93%).

Yield 93%.

^{1}H-NMR [400 MHz, $(CD_3)_2SO$] δ ppm: 12.32 (d, J = 2.6 Hz, 1H), 7.86 (dd, J = 15.1, 2.7 Hz, 1H), 7.61 (dd, J = 23.6, 2.0 Hz, 1H), 7.04 (dt, J = 11.6, 2.3 Hz, 1H), 4.72 (d, J = 37.3 Hz, 2H), 3.12 (d, J = 0.8 Hz, 2H), 3.04 (s, 6H), 1.50 (d, J = 1.5 Hz, 9H).

^{13}C-NMR [101 MHz, $(CD_3)_2SO$] δ ppm: 152.17 (C), 150.00 (C), 146.16 (d, J = 280.1 Hz, C-F), 132.97 (CH), 131.11 (C), 122.38 (C), 107.63 (CH), 103.98 (C), 103.10 (CH), 83.60 (C), 65.38 (CH_2), 60.49 (CH_2), 54.86 (CH_3), 51.69 (CH_3 × 3), 27.78 (CH_3 × 3).

^{19}F-NMR [376 MHz, $(CD_3)_2SO$] δ ppm: –130.81.

LRMS (ESI) *m/z* [M-I]$^+$ calcd. for $C_{17}H_{24}FN_2O_3^+$ 323.38, found 323.35.

2.1.8.5 Preparation of (*S*,*S*)-Ni(II)-BPB-5-OBoc-7-FTrp (S28)

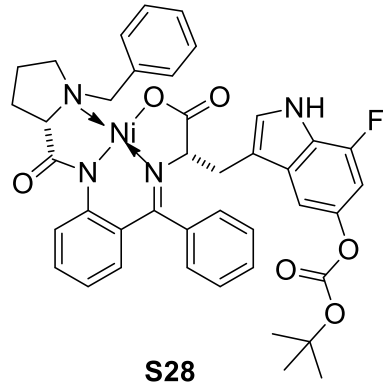

S28

Powdered NaOH (22 mg, 0.55 mmol, 5 eq) was added to a solution of **S27** (50 mg, 0.11 mmol, 1 eq) and (*S*)-Ni-BPB-Gly (55 mg, 0.11 mmol, 1 eq) in anhydrous MeCN (5 mL) and the reaction mixture was stirred at 60°C for 3 h and at ambient temperature overnight. Additional powdered NaOH (13 mg, 0.33 mmol, 3 eq) was added and the mixture was stirred at 60°C overnight. Saturated NH_4Cl (40 mL) was added, the resulting emulsion was extracted with EtOAc (4 × 10 mL), and the combined organic phases were dried and concentrated under reduced pressure. The residue was purified by column chromatography (silica gel, $CHCl_3$:acetone / 3:1) to afford the desired product as a red solid (14 mg, 0.018 mmol, 16%).

Yield 16%.

^{1}H-NMR (400 MHz, $CDCl_3$) δ ppm: 8.32 (dd, J = 8.7, 1.2 Hz, 1H), 8.15 – 8.04 (m, 2H), 7.65 – 7.48 (m, 3H), 7.46 – 7.39 (m, 2H), 7.39 – 7.27 (m, 2H), 7.25 – 7.18 (m, 2H), 7.18 – 7.06 (m, 2H), 7.05 – 6.95 (m, 1H), 6.82 (dd, J = 8.2, 1.7 Hz, 1H), 6.72 (ddd, J = 8.1, 6.9, 1.1 Hz, 1H), 6.65 (ddd, J = 8.2, 7.0, 1.1 Hz, 0H), 6.56 – 6.45 (m, 1H), 4.49 (dd, J = 12.6, 5.1 Hz, 1H), 3.85 – 3.62 (m, 3H), 3.52 – 3.15 (m, 3H), 2.20 – 1.99 (m, 3H), 1.61 (s, 3H), 1.27 (d, J = 7.4 Hz, 2H).

^{13}C-NMR (101 MHz, $CDCl_3$) δ ppm: 171.52 (d, J = 48.2 Hz, C), 159.46 (C), 153.55 (C), 151.24 (C), 148.53 (d, J = 38.8 Hz, C-F), 142.56 (C), 141.25 (C), 134.66 (C), 133.58 (CH), 133.40 (d, J = 6.6 Hz, C), 133.22 (CH), 133.08 (C), 132.67 (CH), 132.45 (CH), 132.26 (CH), 131.78 (CH), 131.33 (CH), 130.77 (CH), 130.63 (CH), 130.38 (CH), 130.05 (CH), 129.77 (CH), 129.63 (CH), 129.37 (CH), 129.15 (CH), 128.96 (CH), 128.85 (CH), 128.72 (CH), 128.53 (CH), 128.36 (CH), 128.07 (CH), 127.65 (d, J = 9.1 Hz, CH), 126.82 (C), 126.31 (CH), 125.71 (CH), 125.24 (C), 124.32 (CH), 123.59 (CH), 121.20 (CH), 120.90 (CH), 102.20 (d, J = 23.9 Hz, CH), 100.08 (CH),

83.91 (C), 70.84 (CH), 69.93 (CH), 63.18 (CH_2), 57.55 (CH_2), 30.74 (CH_2), 30.27 (CH_2), 27.98 (CH_3 × 3), 23.74 (CH_2).

^{19}F-NMR (376 MHz, $CDCl_3$) δ ppm: −114.32.

LRMS (ESI) *m/z* [M-Boc+H]$^+$ calcd. for $C_{36}H_{32}FN_4NiO_4^+$ 661.18, found 661.29.

This path proved to be too inefficient for the planned follow-up steps, requiring large amounts of expensive starting materials to produce sufficient amounts of Boc-5-Bpin-7-FTrp-O*t*Bu.

3 Characterization

3.1 ^{1}H-, ^{13}C- and ^{19}F-NMR-spectra

3.1.1 ^{1}H-NMR of 6-bromo-1-methyl-1*H*-indole (2)

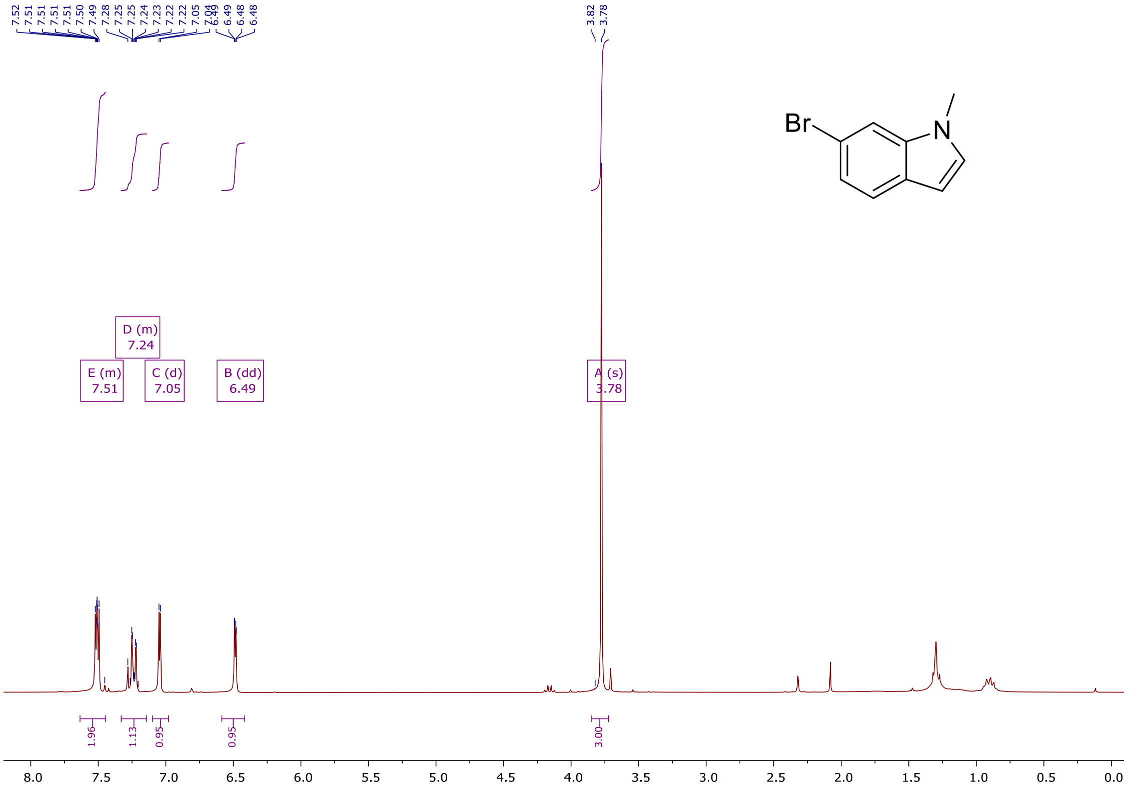

3.1.2 ^{13}C-NMR of 6-bromo-1-methyl-1*H*-indole (2)

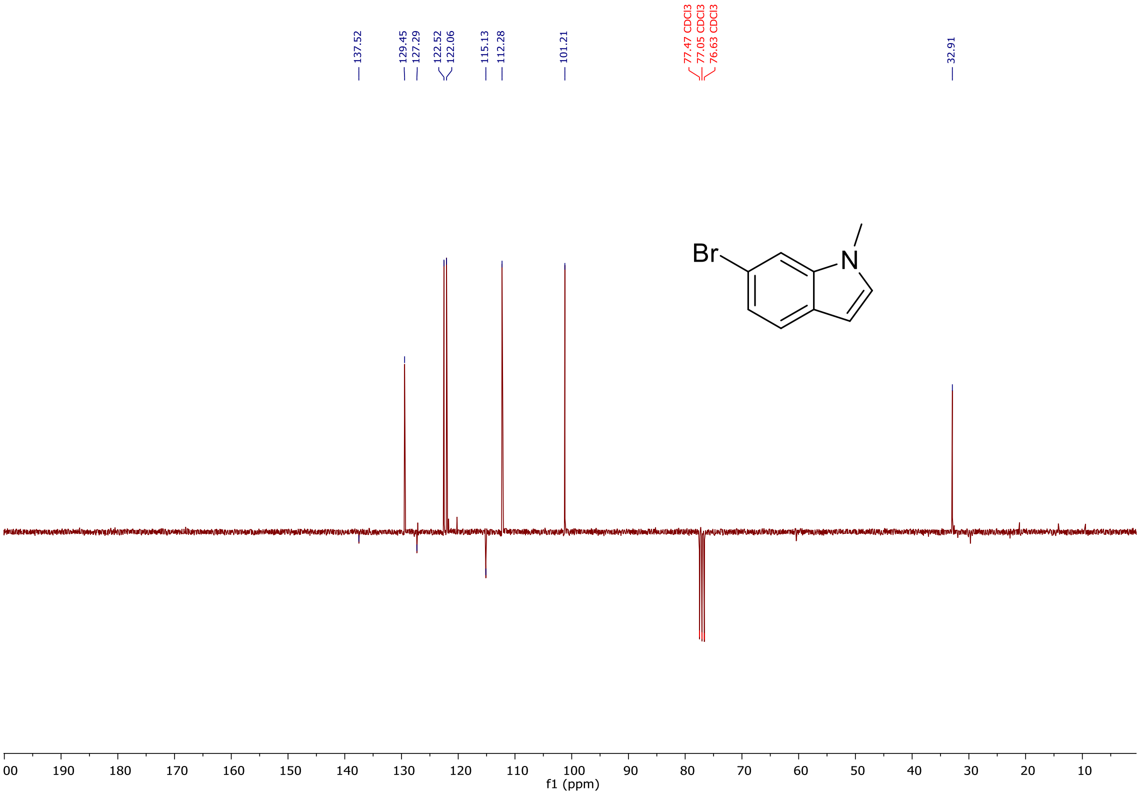

3.1.3 ^{1}H-NMR of 1-methyl-6-(4,4,5,5-tetramethyl-1,3,2-dioxaborolan-2-yl)-1*H*-indole (3)

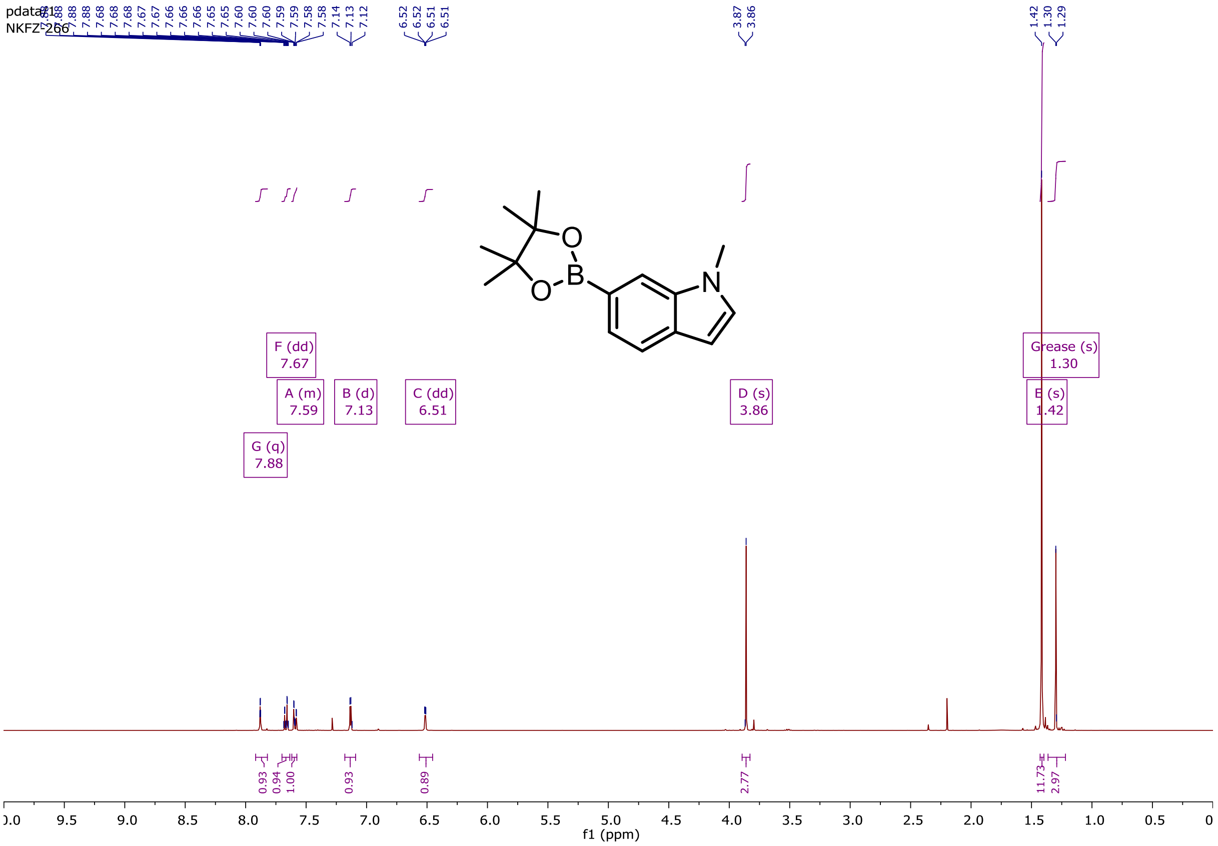

3.1.4 ^{13}C-NMR of 1-methyl-6-(4,4,5,5-tetramethyl-1,3,2-dioxaborolan-2-yl)-1*H*-indole (3)

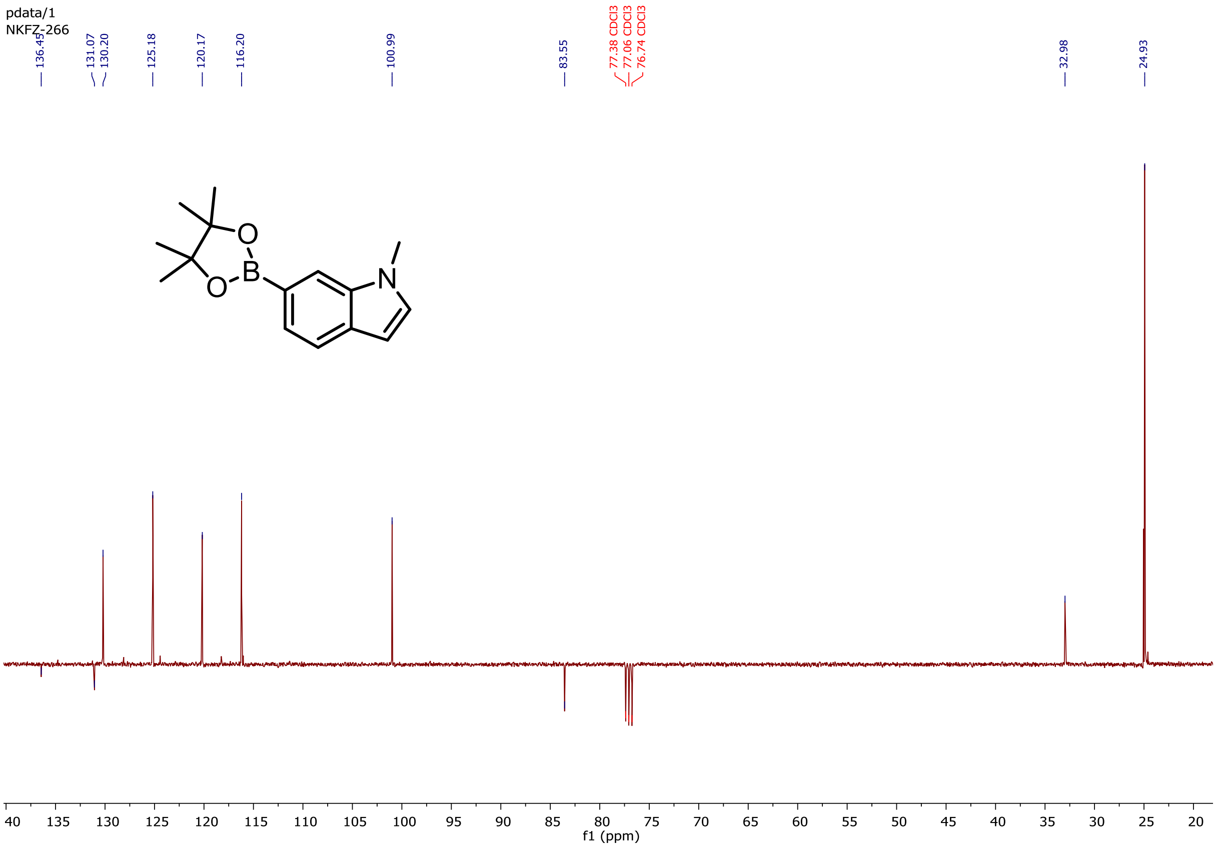

3.1.5 ^{1}H-NMR of *N,N*-dimethyl-1-[1-methyl-6-(4,4,5,5-tetramethyl-1,3,2-dioxaborolan-2-yl)-1*H*-indol-3-yl]methanamine (4)

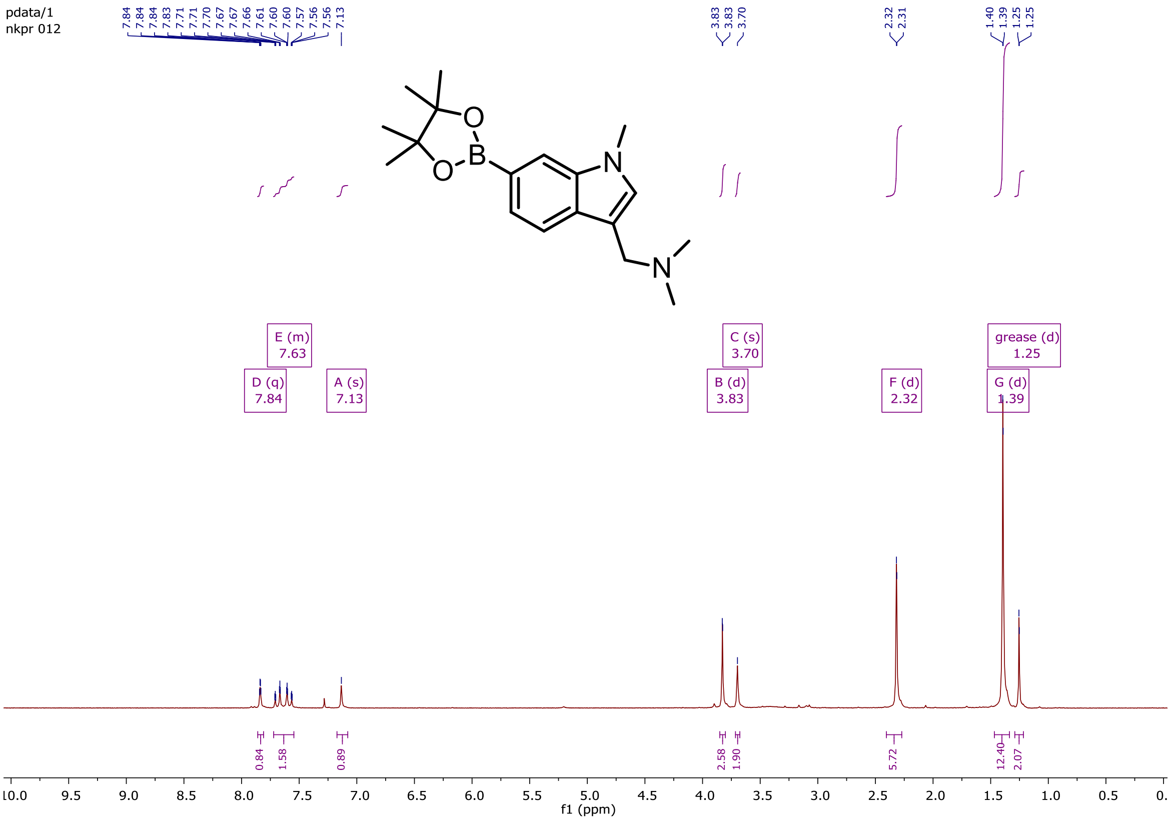

3.1.6 ^{13}C-NMR of *N,N*-dimethyl-1-[1-methyl-6-(4,4,5,5-tetramethyl-1,3,2-dioxaborolan-2-yl)-1*H*-indol-3-yl]methanamine (4)

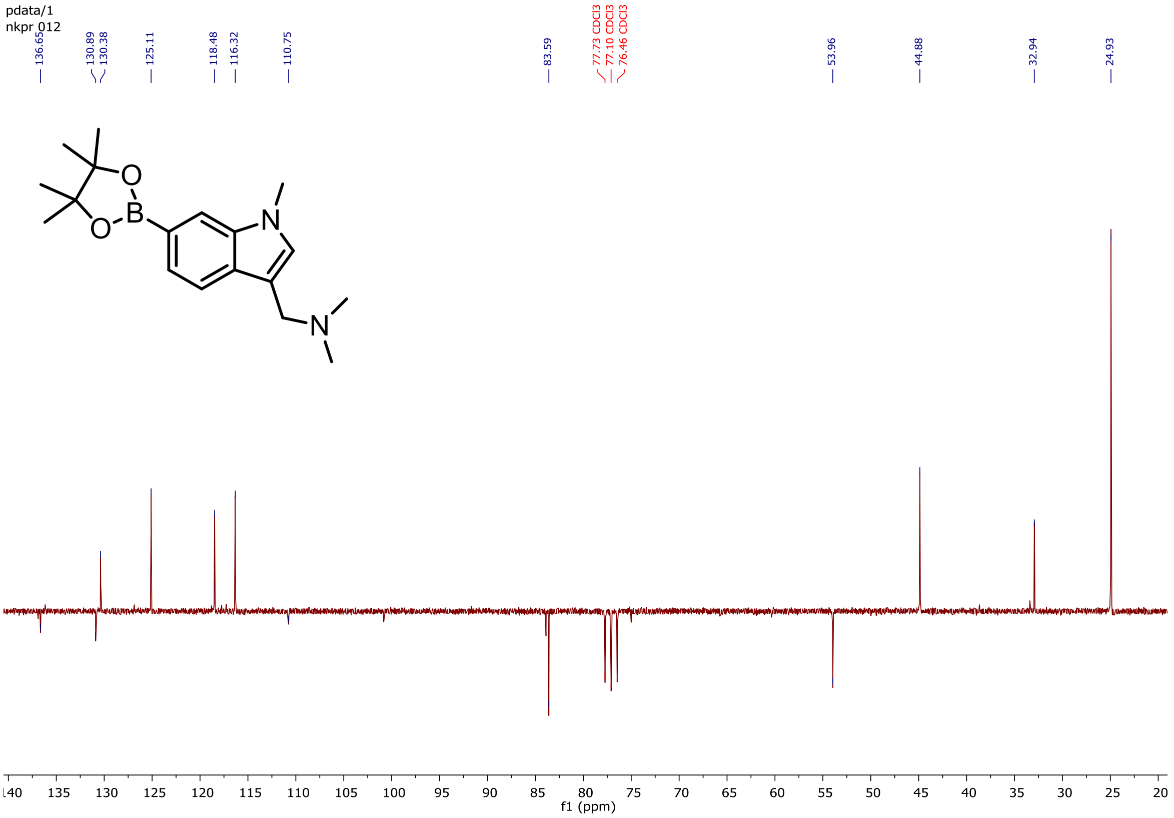

3.1.7 ^{1}H-NMR of *N,N,N*-trimethyl-1-[1-methyl-6-(4,4,5,5-tetramethyl-1,3,2-dioxaborolan-2-yl)-1*H*-indol-3-yl]methanaminium iodide (5)

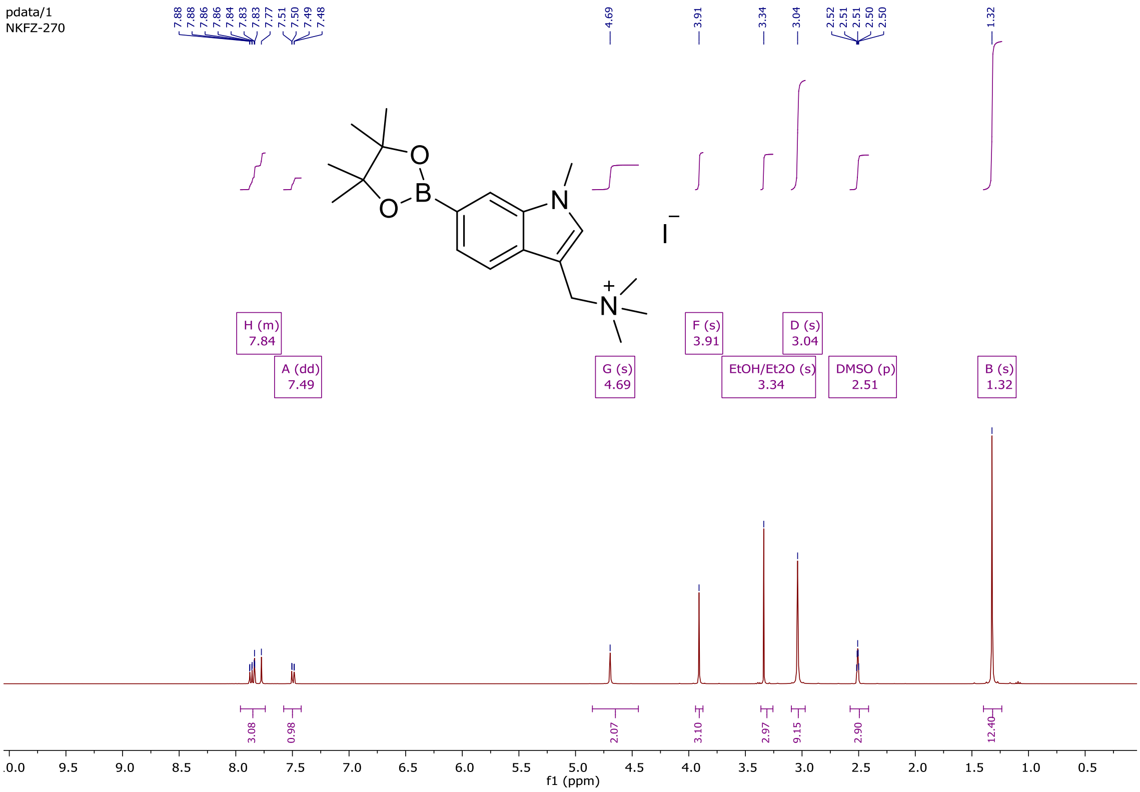

3.1.8 ^{13}C-NMR of *N,N,N*-trimethyl-1-[1-methyl-6-(4,4,5,5-tetramethyl-1,3,2-dioxaborolan-2-yl)-1*H*-indol-3-yl]methanaminium iodide (5)

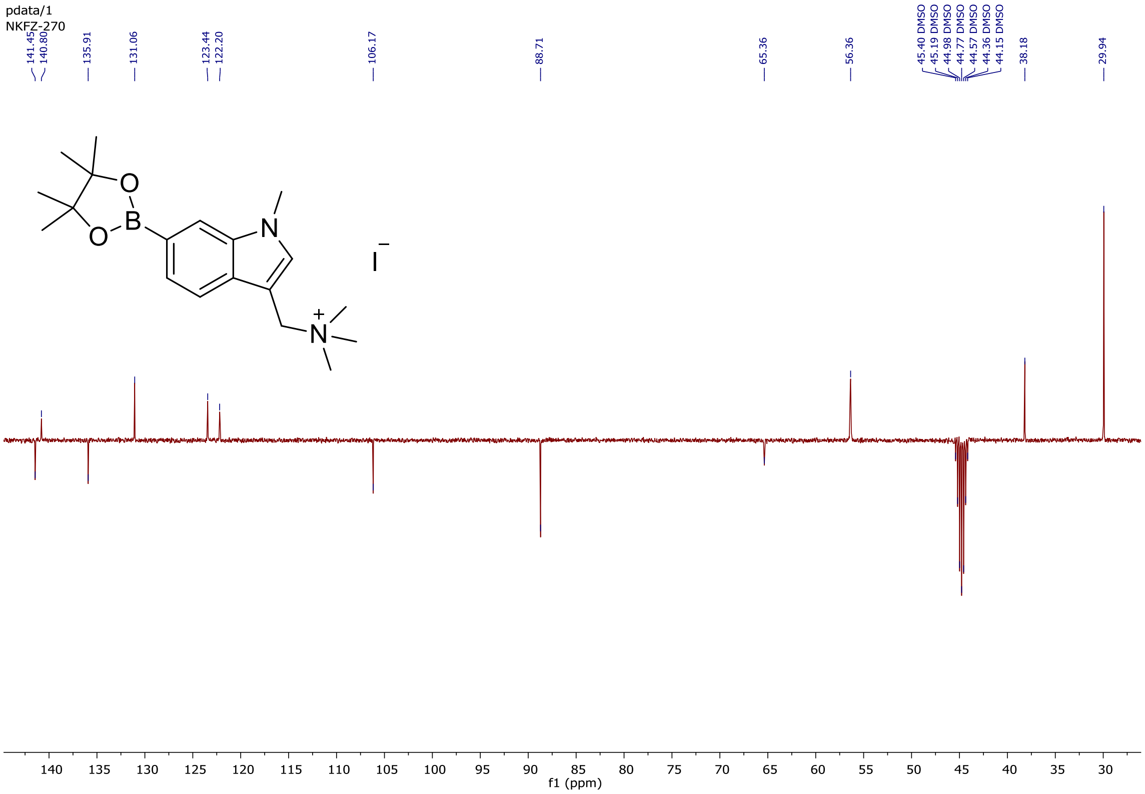

3.1.9 ^{1}H-NMR of (*S*,*S*)-Ni-BPB-*N*$_{In}$-methyl-6-Bpin-tryptophan (6)

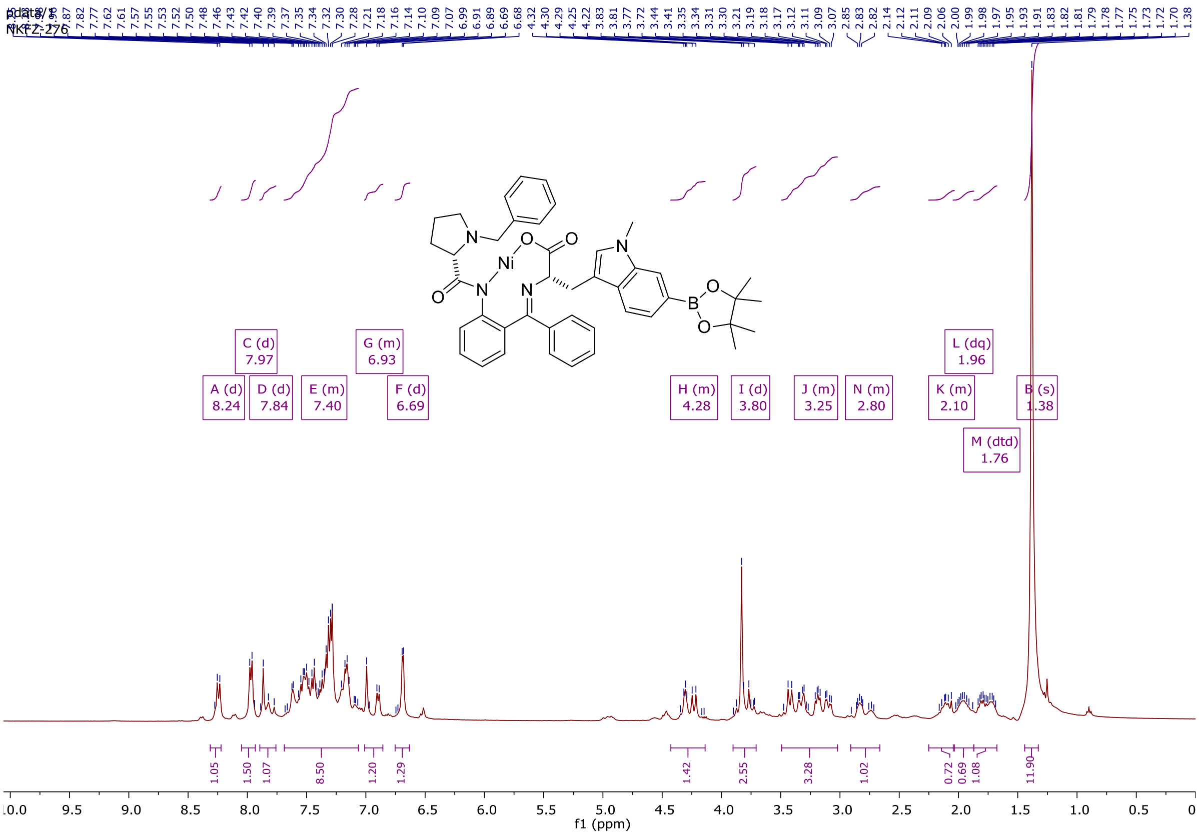

3.1.10 ^{13}C-NMR of (*S*,*S*)-Ni-BPB-*N*$_{In}$-methyl-6-Bpin-tryptophan (6)

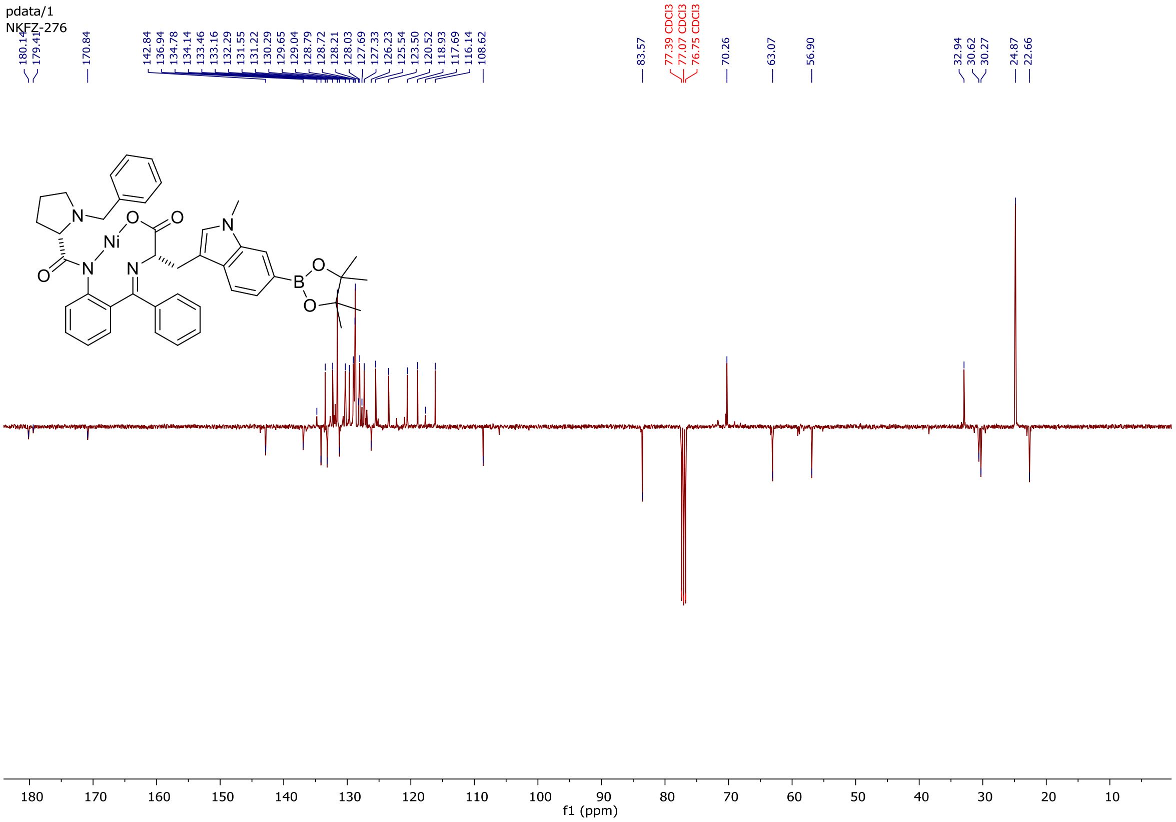

3.1.11 ^{1}H-NMR of 1-(6-fluoro-1*H*-indol-3-yl)-*N,N*-dimethylmethanamine (8)

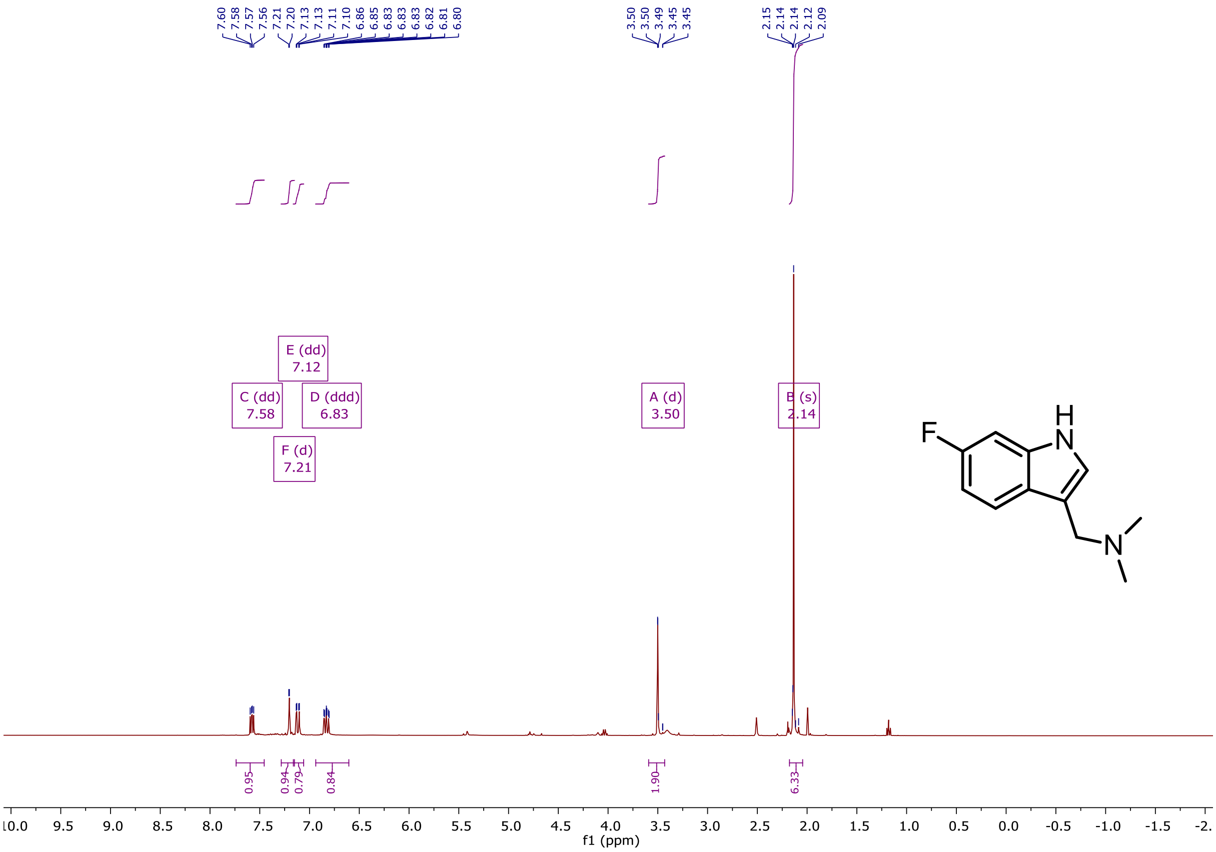

3.1.12 ^{13}C-NMR of 1-(6-fluoro-1*H*-indol-3-yl)-*N,N*-dimethylmethanamine (8)

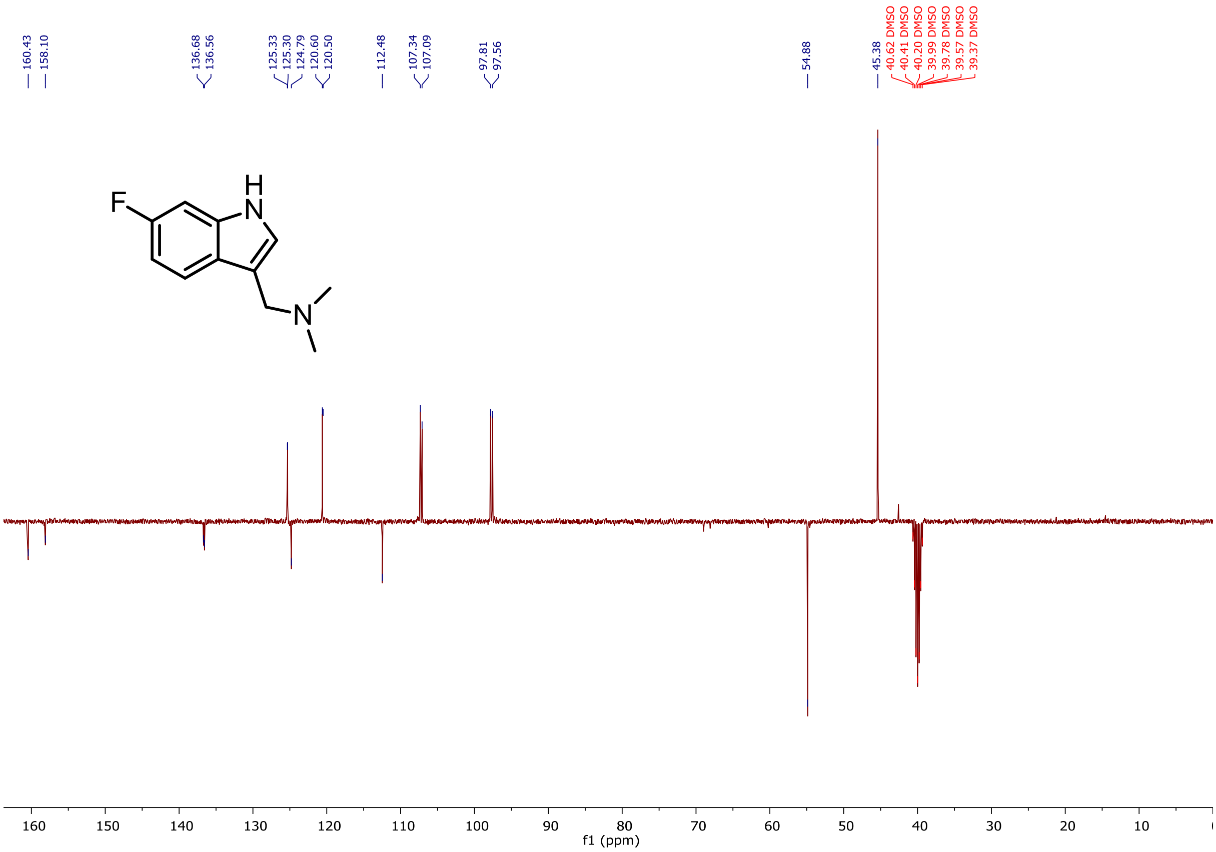

3.1.13 ^{19}F-NMR of 1-(6-fluoro-1*H*-indol-3-yl)-*N,N*-dimethylmethanamine (8)

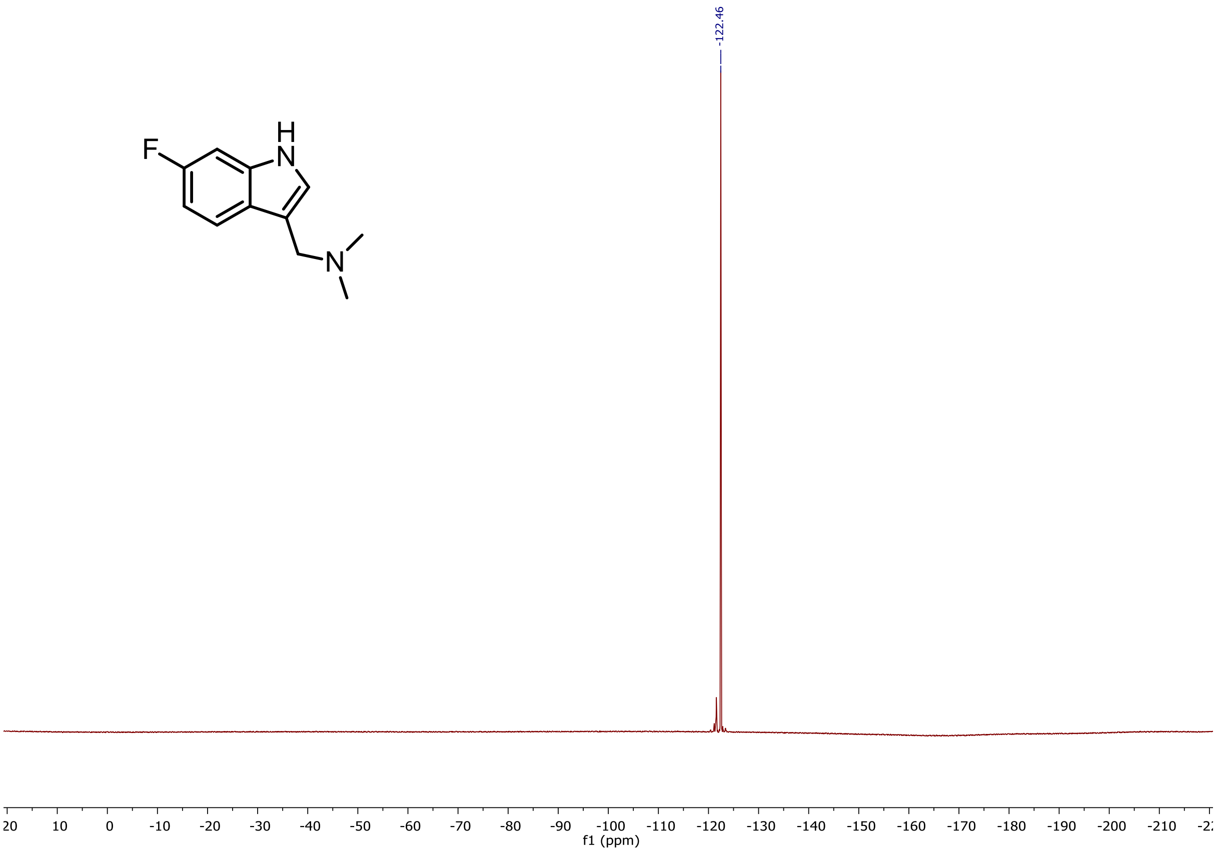

3.1.14 ^{1}H-NMR of 1-(6-fluoro-1*H*-indol-3-yl)-*N,N,N*-trimethylmethanaminium iodide (9)

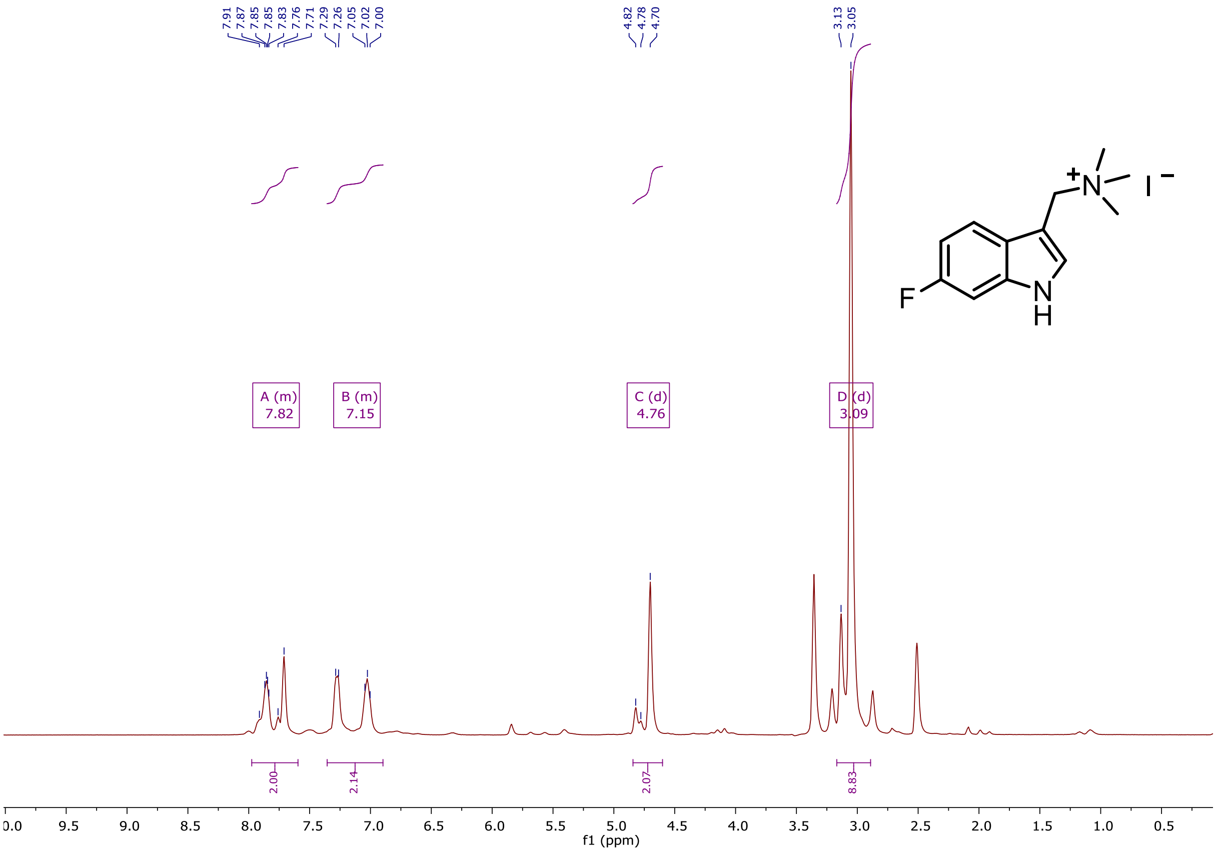

3.1.15 ^{13}C-NMR of 1-(6-fluoro-1*H*-indol-3-yl)-*N,N,N*-trimethylmethanaminium iodide (9)

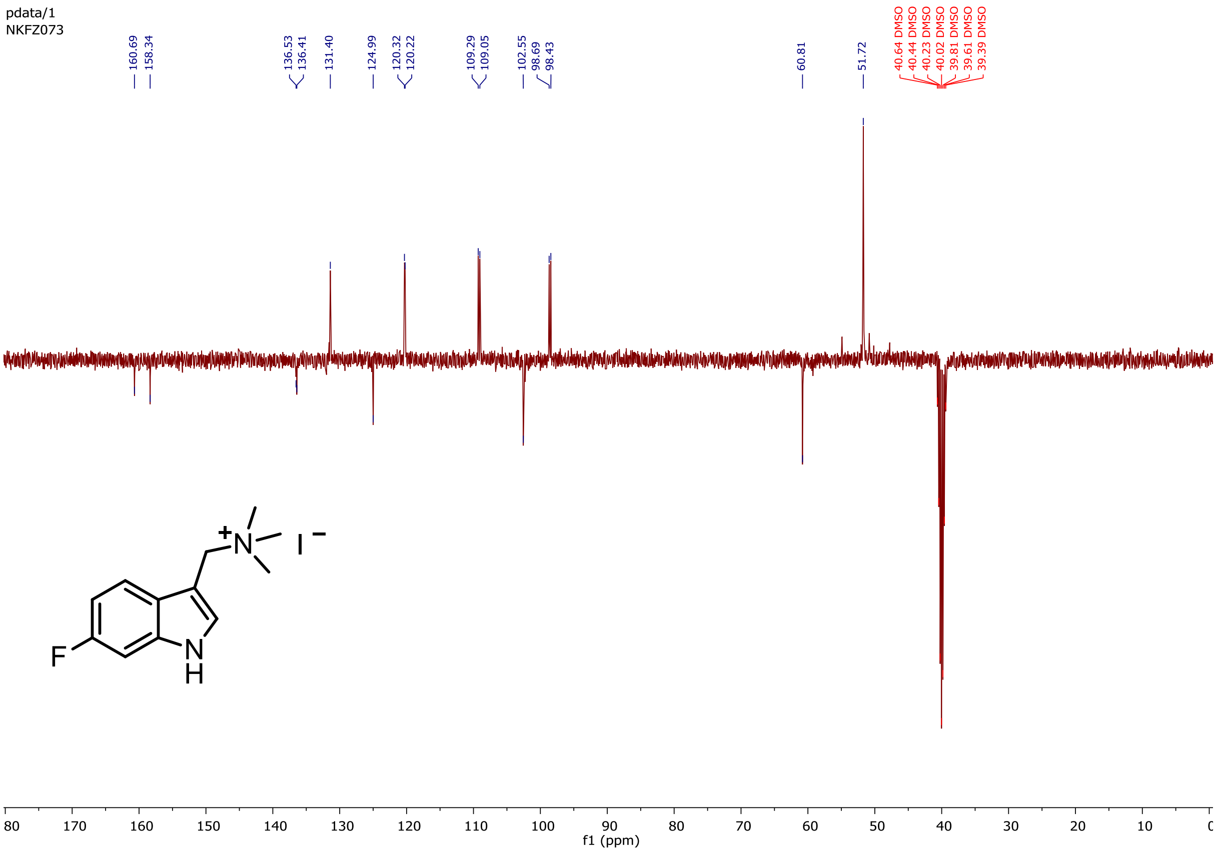

3.1.16 ^{19}F-NMR of 1-(6-fluoro-1*H*-indol-3-yl)-*N,N,N*-trimethylmethanaminium iodide (9)

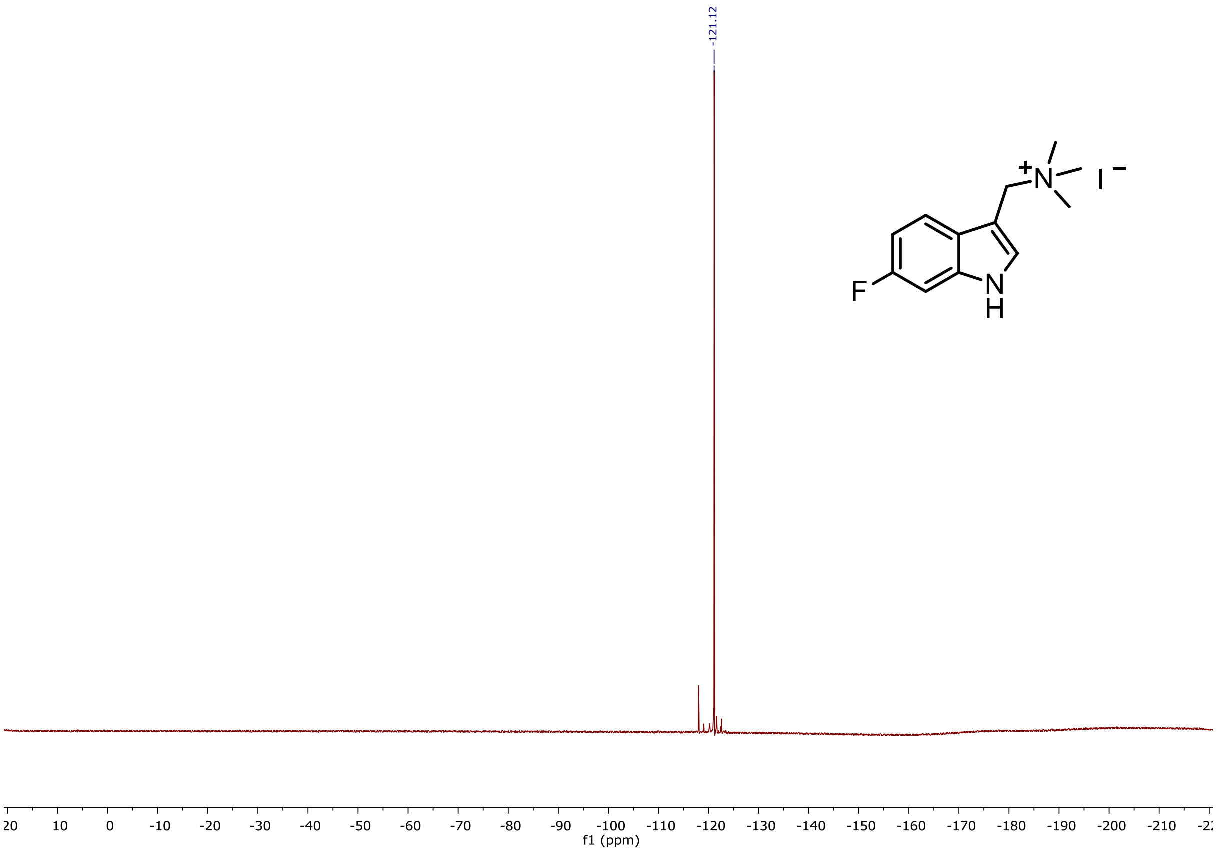

3.1.17 ^{1}H-NMR of (*R,R*)-Ni(II)-BPB-6-fluoro-tryptophan (*R,R*-10)

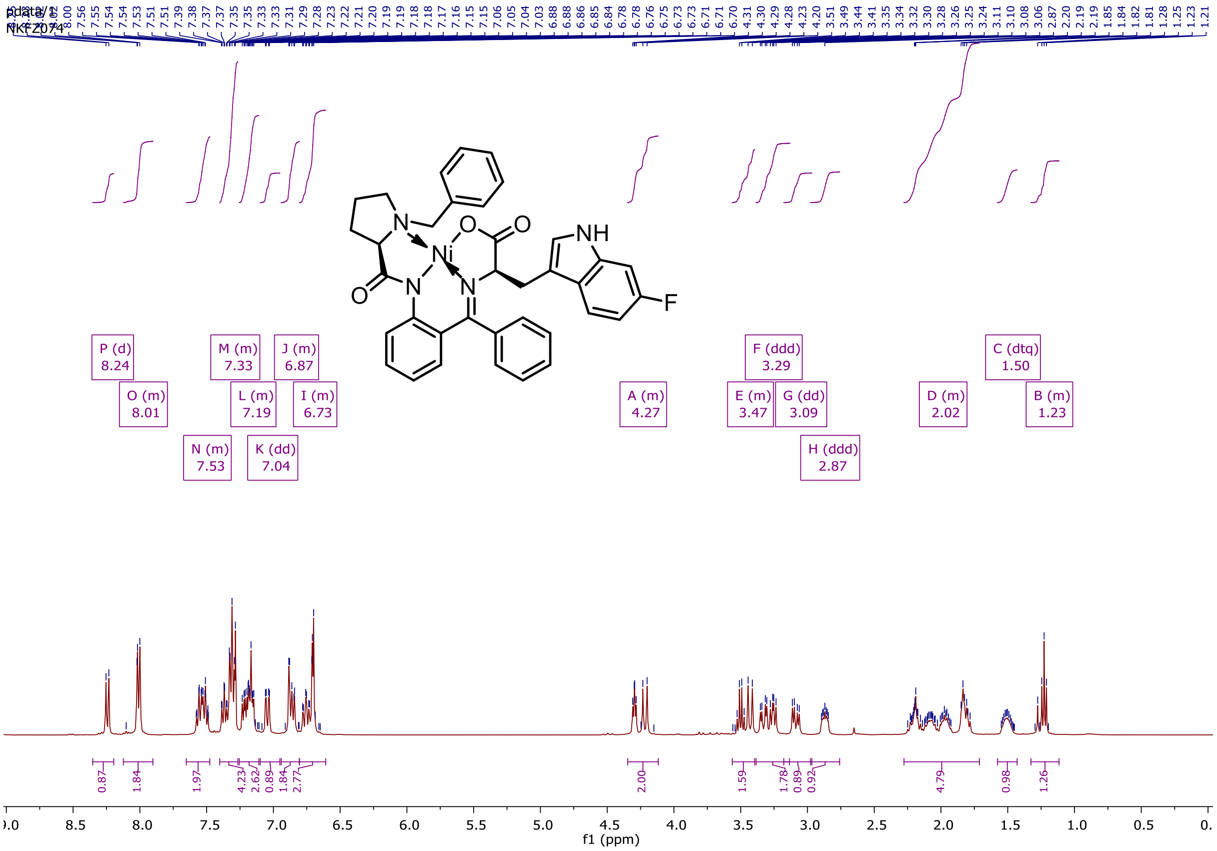

3.1.18 ^{13}C-NMR of (*R,R*)-Ni(II)-BPB-6-fluoro-tryptophan (*R,R*-10)

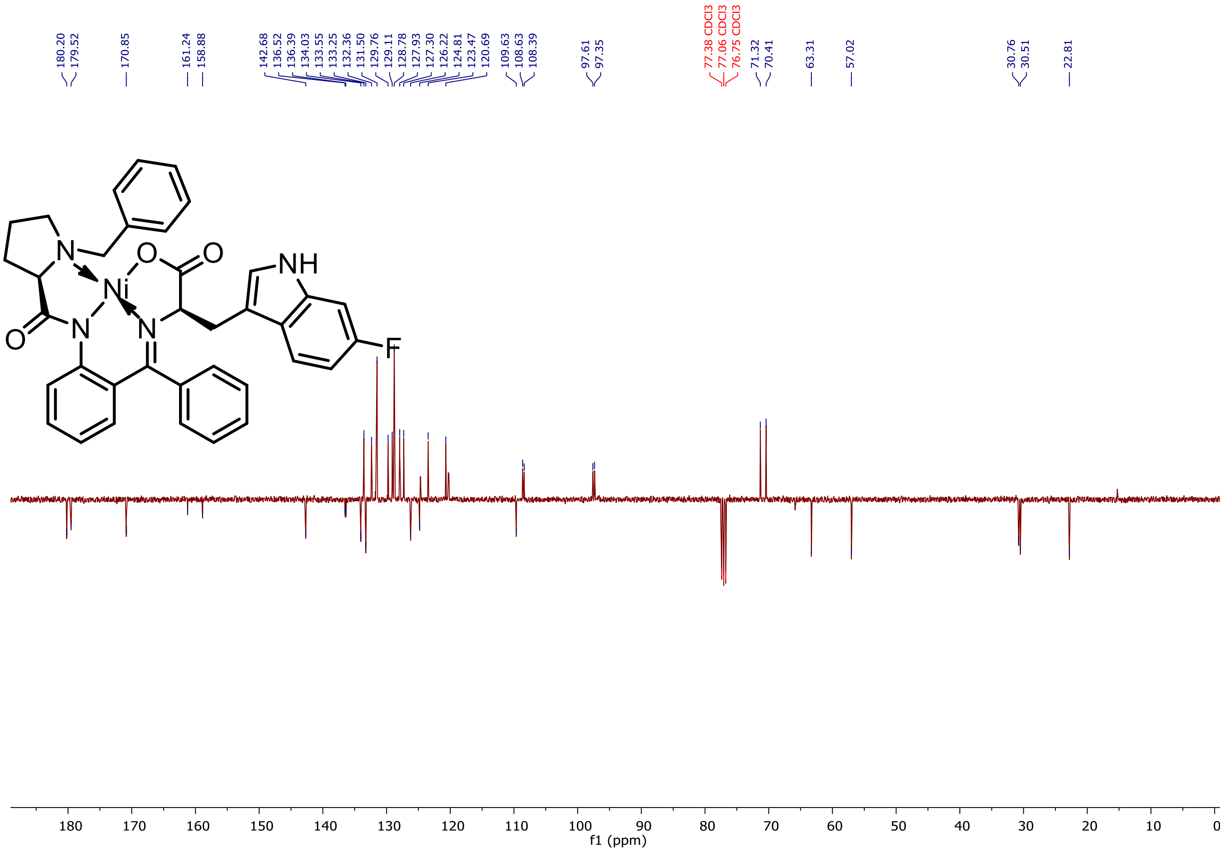

3.1.19 ^{19}F-NMR of (*R,R*)-Ni(II)-BPB-6-fluoro-tryptophan (*R,R*-10)

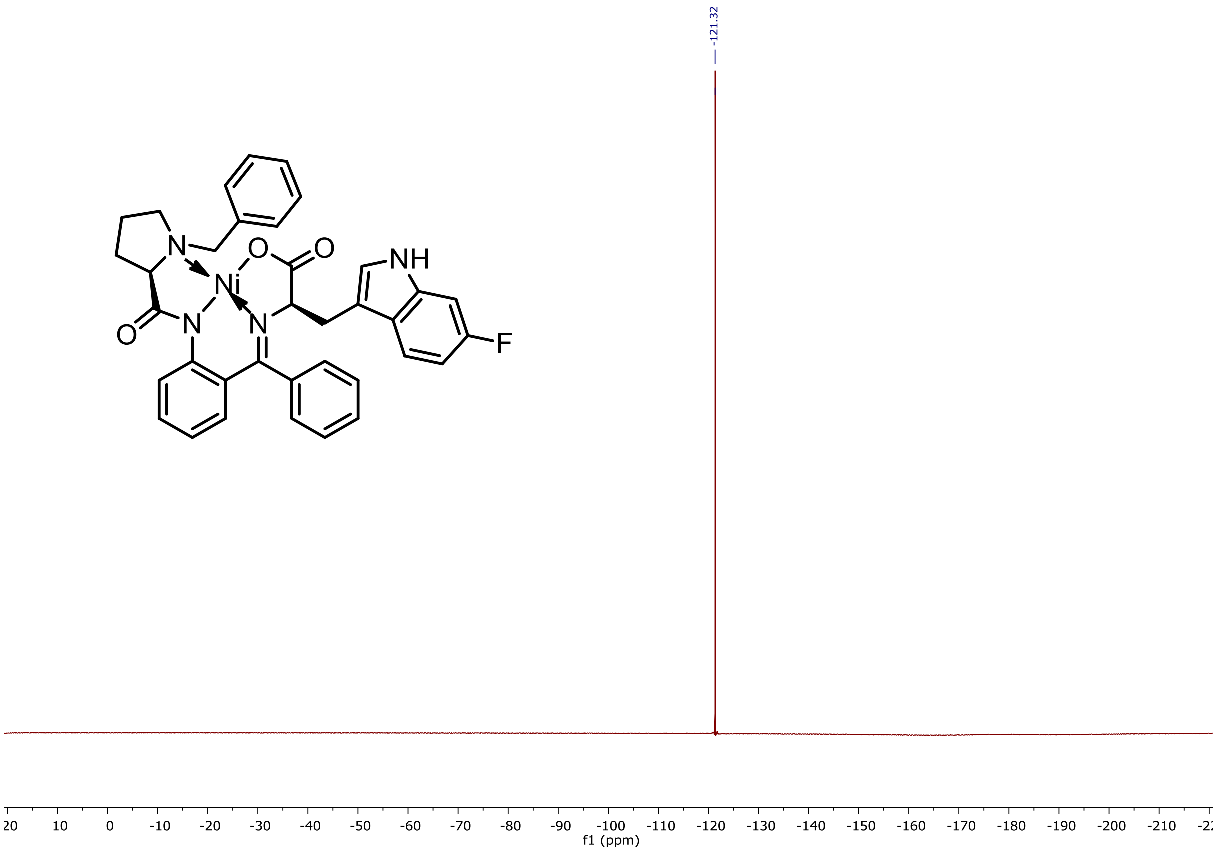

3.1.20 ^{1}H-NMR of (*R*)-2-[(*tert*-butoxycarbonyl)amino]-3-(6-fluoro-1*H*-indol-3-yl)propanoic acid (*R*-11)

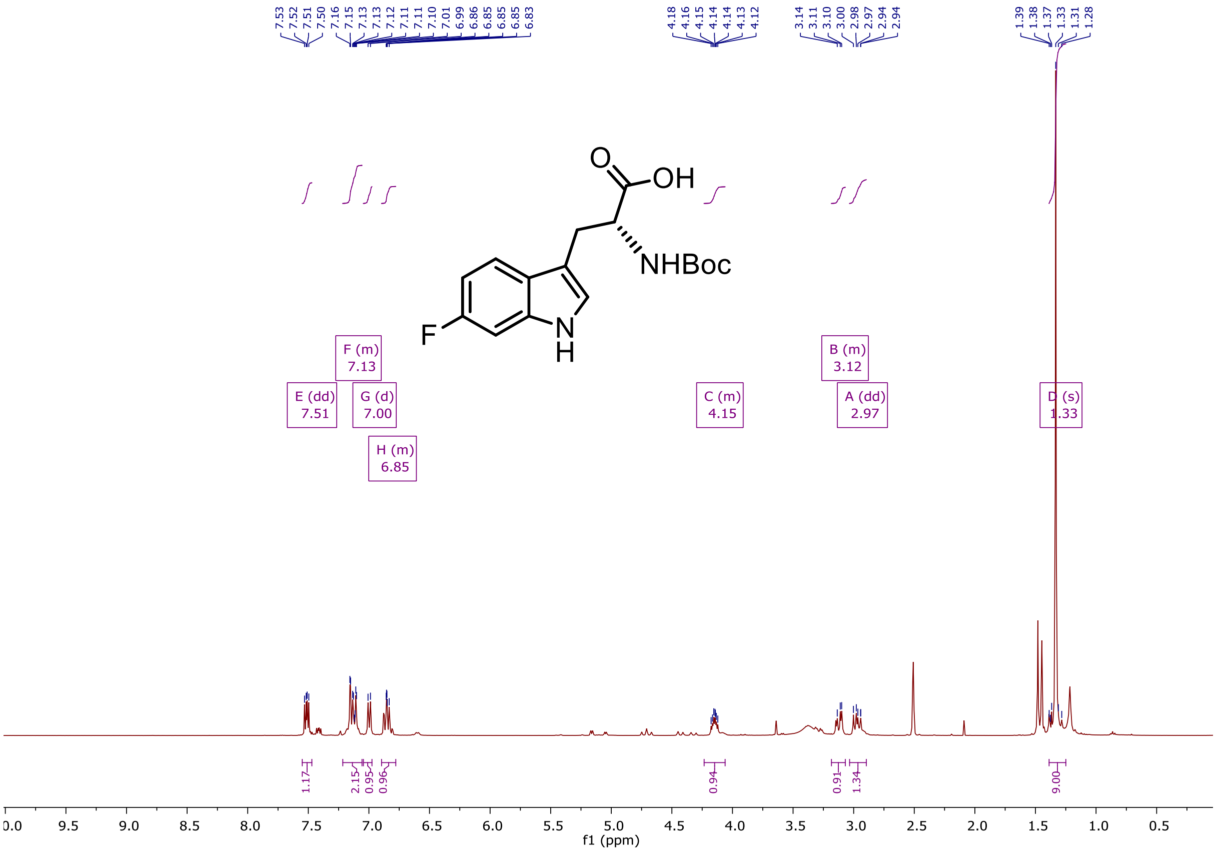

3.1.21 ¹³C-NMR of (*R*)-2-[(*tert*-butoxycarbonyl)amino]-3-(6-fluoro-1*H*-indol-3-yl)propanoic acid (*R*-11)

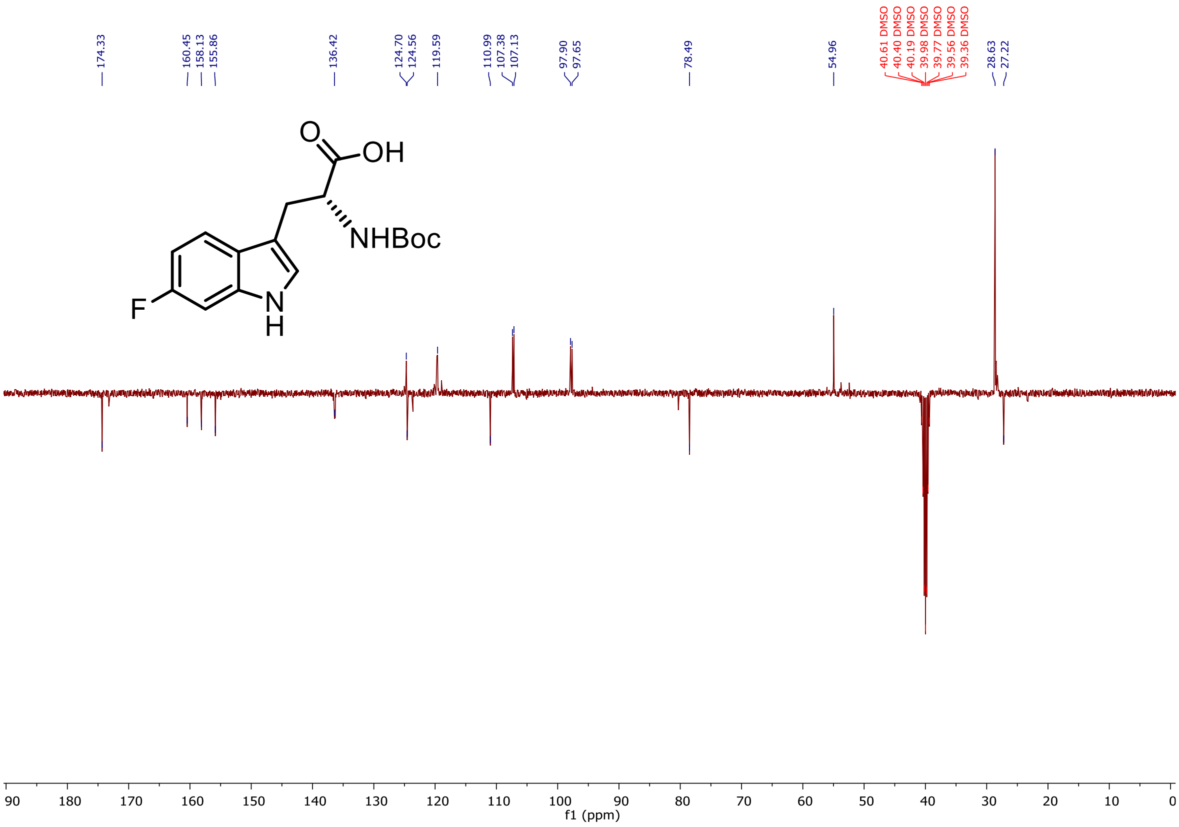

3.1.22 ¹⁹F-NMR of (*R*)-2-[(*tert*-butoxycarbonyl)amino]-3-(6-fluoro-1*H*-indol-3-yl)propanoic acid (*R*-11)

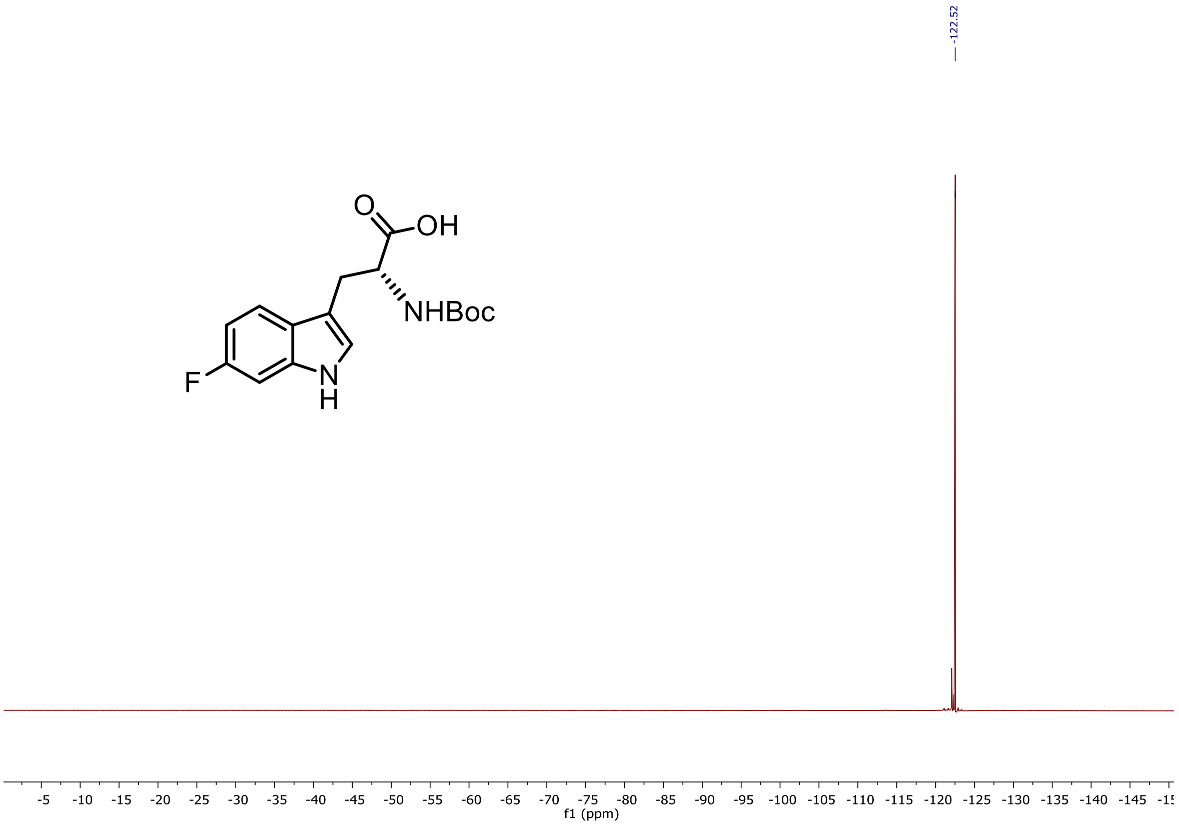

3.1.23 ^{1}H-NMR of (*S*)-2-[(*tert*-butoxycarbonyl)amino]-3-(6-fluoro-1-methyl-1*H*-indol-3-yl)propanoic acid (*S*-12)

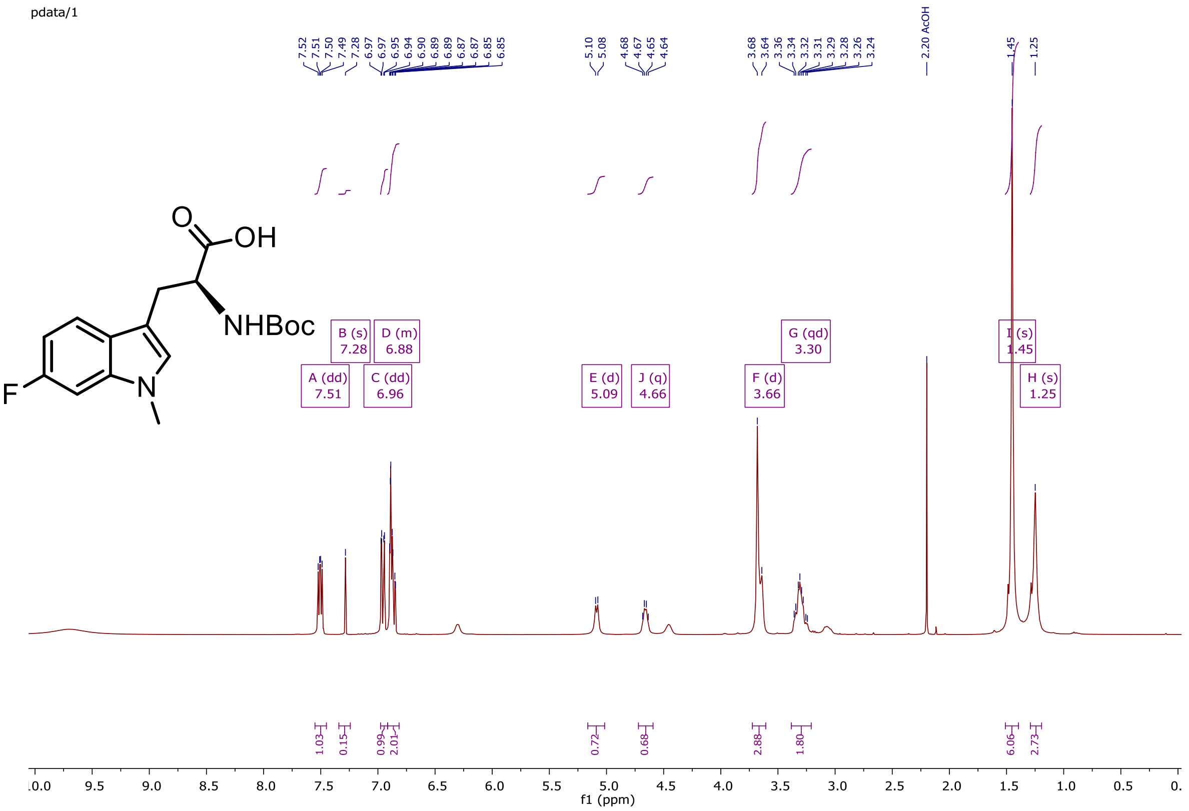

3.1.24 ^{13}C-NMR of (*S*)-2-[(*tert*-butoxycarbonyl)amino]-3-(6-fluoro-1-methyl-1*H*-indol-3-yl)propanoic acid (*S*-12)

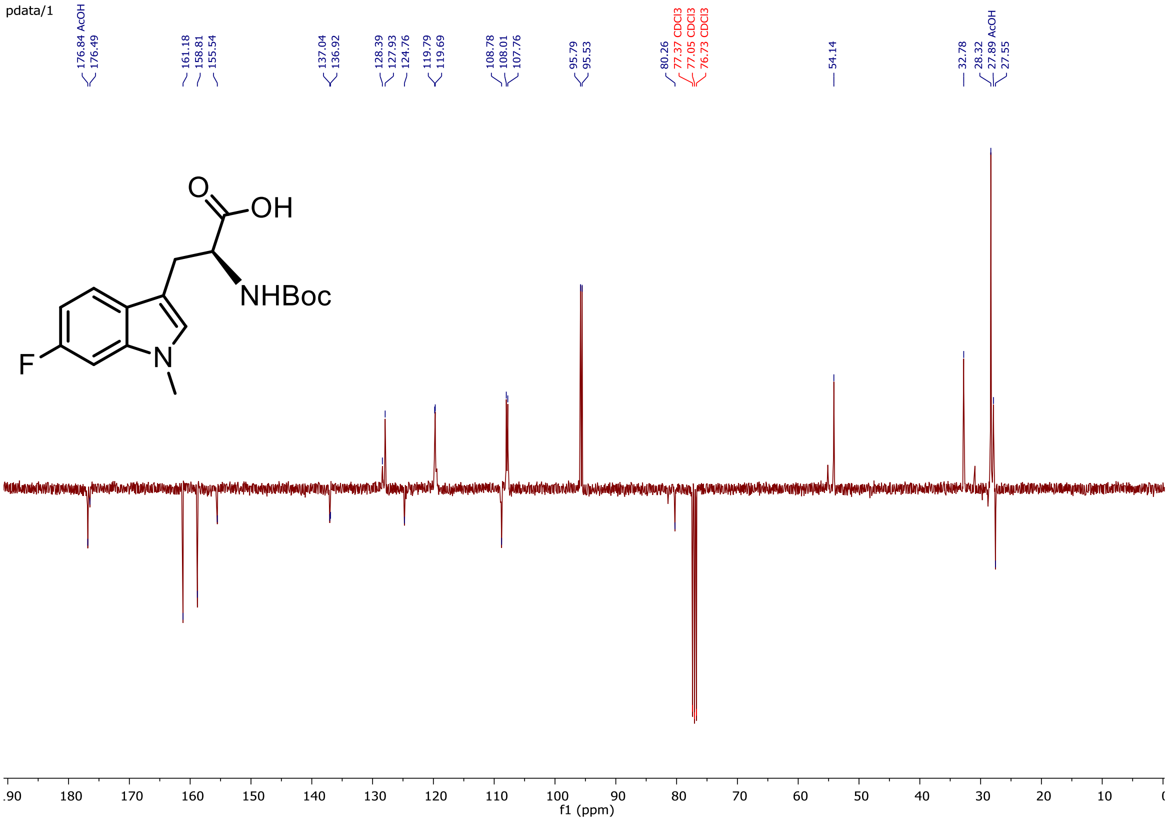

3.1.25 ^{19}F-NMR of (*S*)-2-[(*tert*-butoxycarbonyl)amino]-3-(6-fluoro-1-methyl-1*H*-indol-3-yl)propanoic acid (*S*-12)

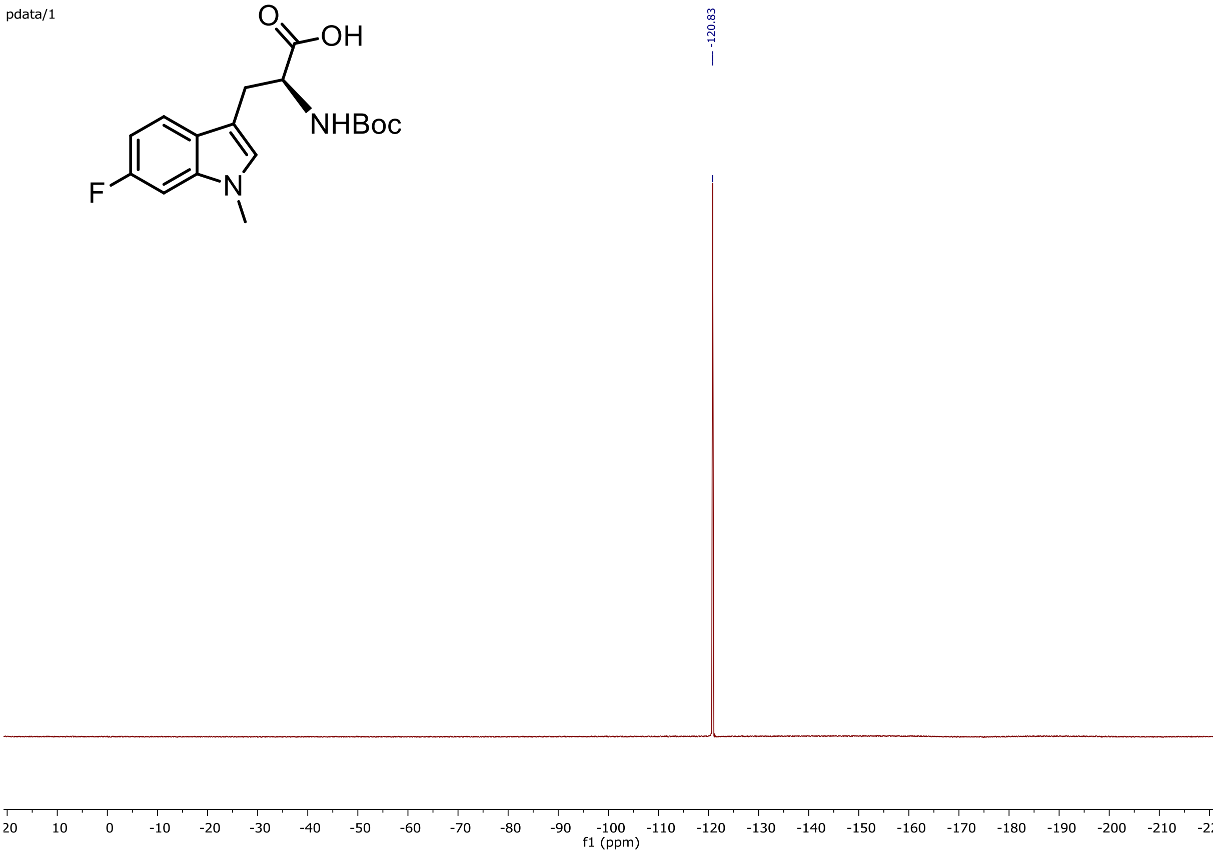

3.1.26 ^{1}H-NMR of (*S*)-2-amino-3-(6-fluoro-1-methyl-1*H*-indol-3-yl)propanoic acid hydrochloride (*S*-13 × HCl)

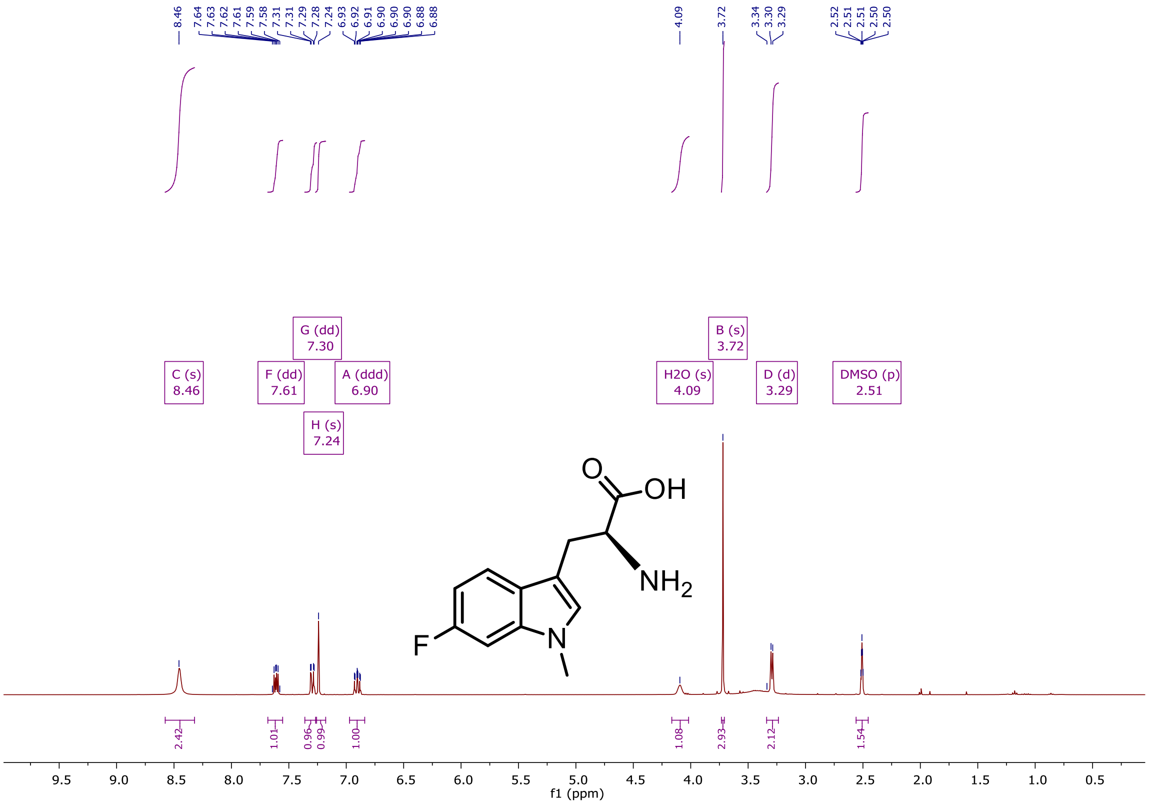

3.1.27 ^{13}C-NMR of (*S*)-2-amino-3-(6-fluoro-1-methyl-1*H*-indol-3-yl)propanoic acid hydrochloride (*S*-13 × HCl)

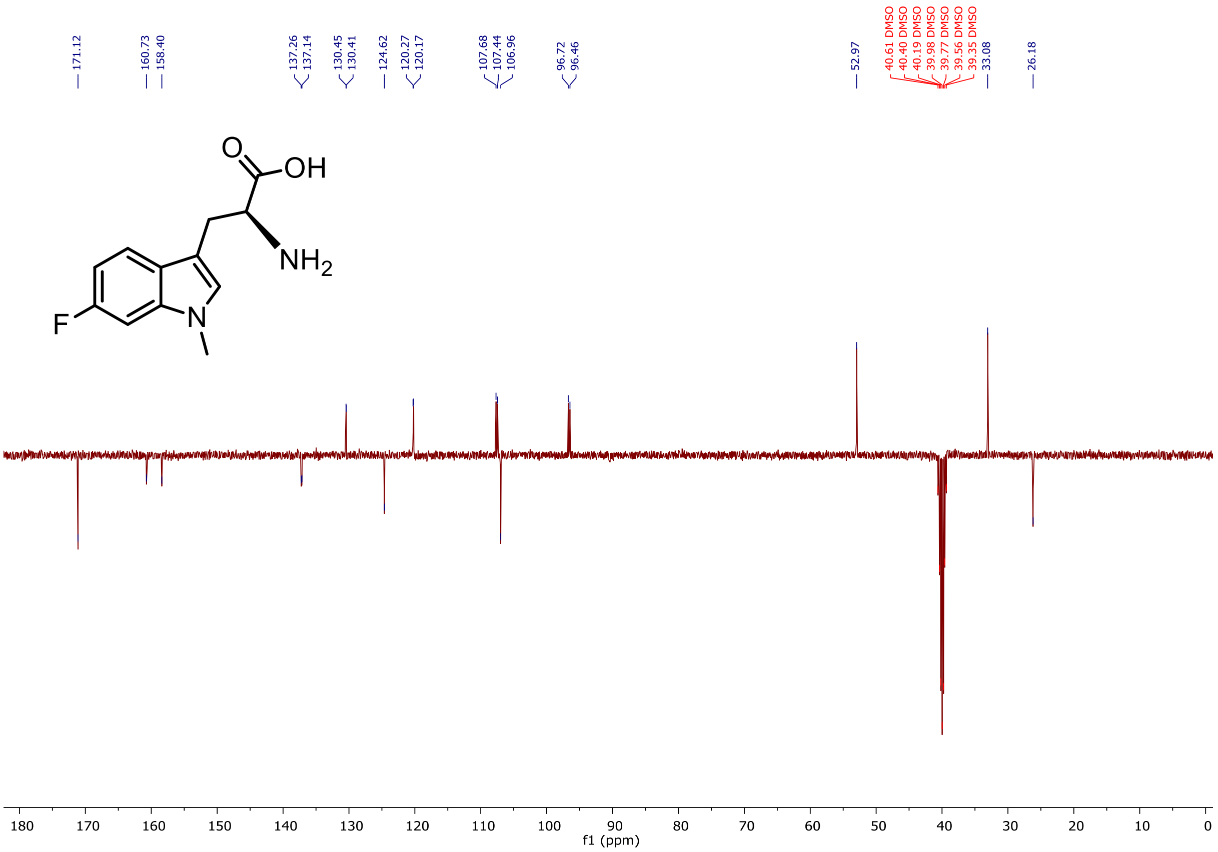

3.1.28 ^{19}F-NMR of (*S*)-2-amino-3-(6-fluoro-1-methyl-1*H*-indol-3-yl)propanoic acid hydrochloride (*S*-13 × HCl)

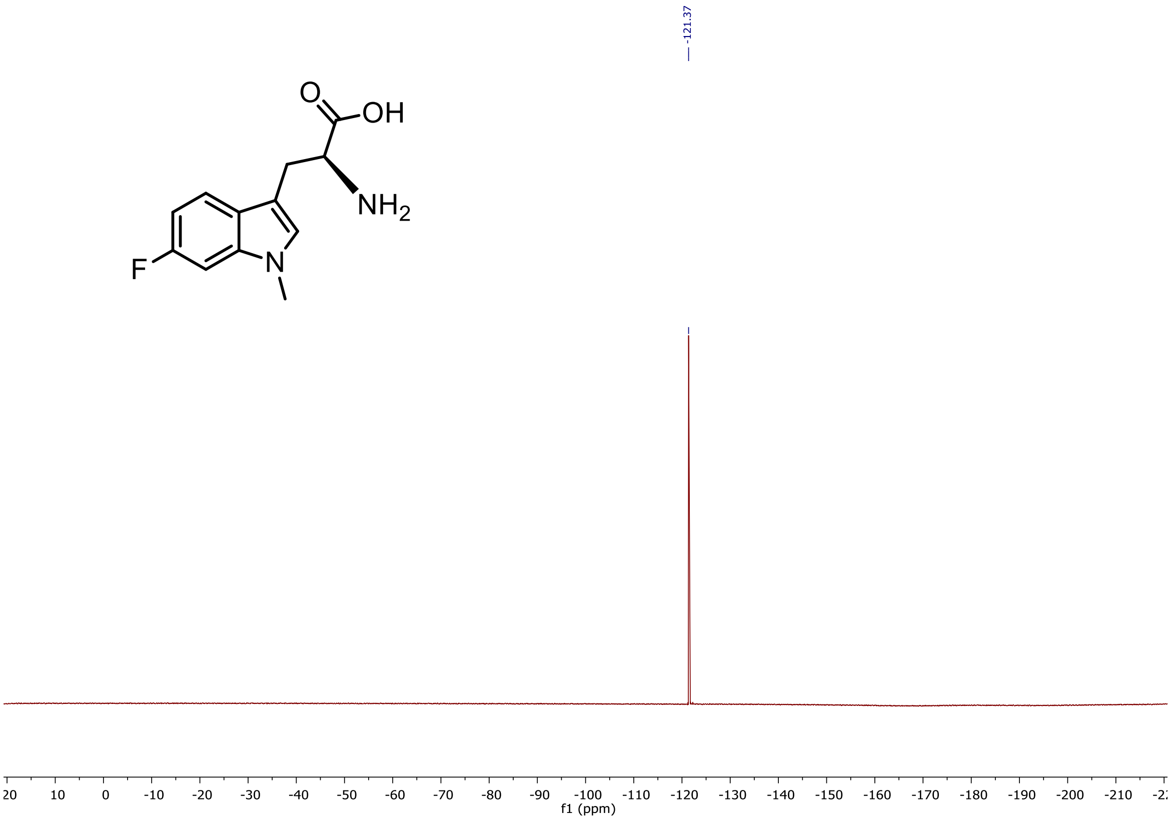

3.1.29 ^{1}H-NMR of (*S*)-3-(5-acetoxy-1*H*-indol-3-yl)-2-aminopropanoic acid (15)

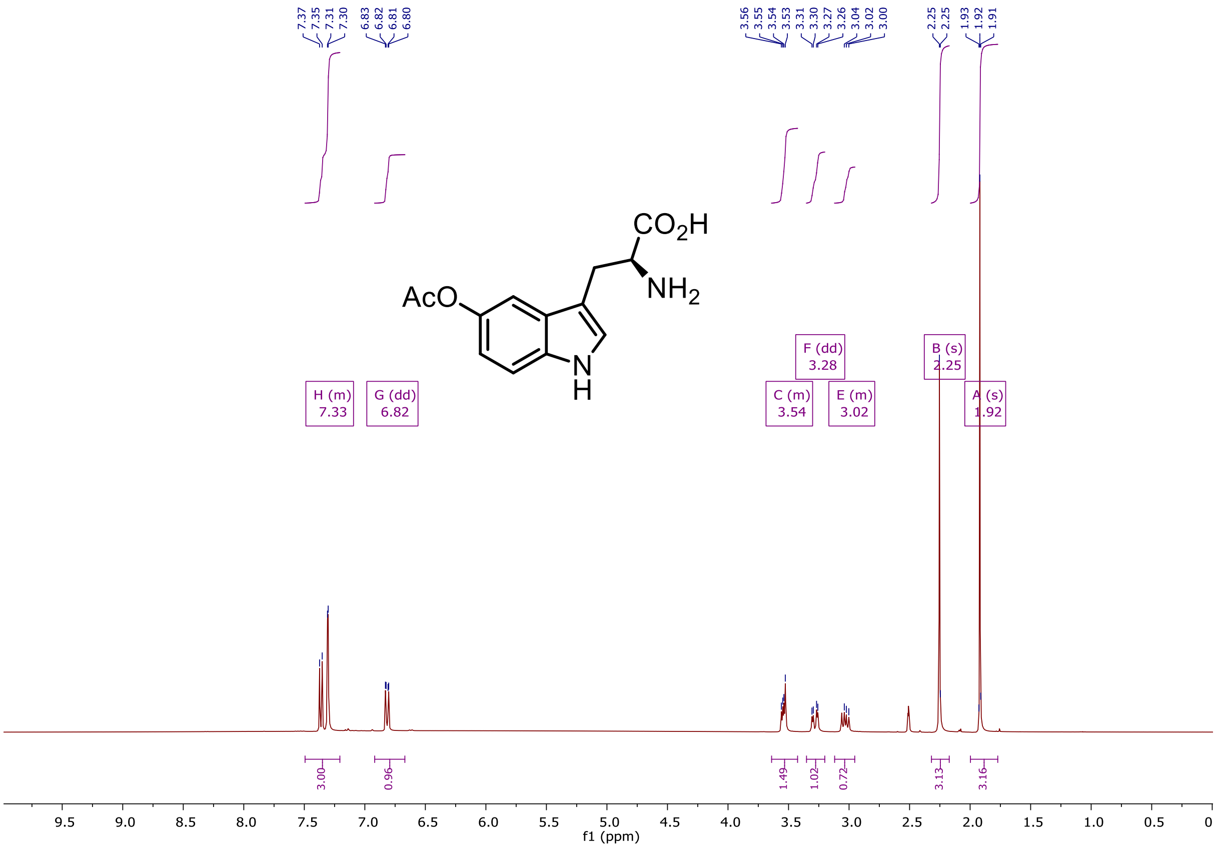

3.1.30 ^{13}C-NMR of (*S*)-3-(5-acetoxy-1*H*-indol-3-yl)-2-aminopropanoic acid (15)

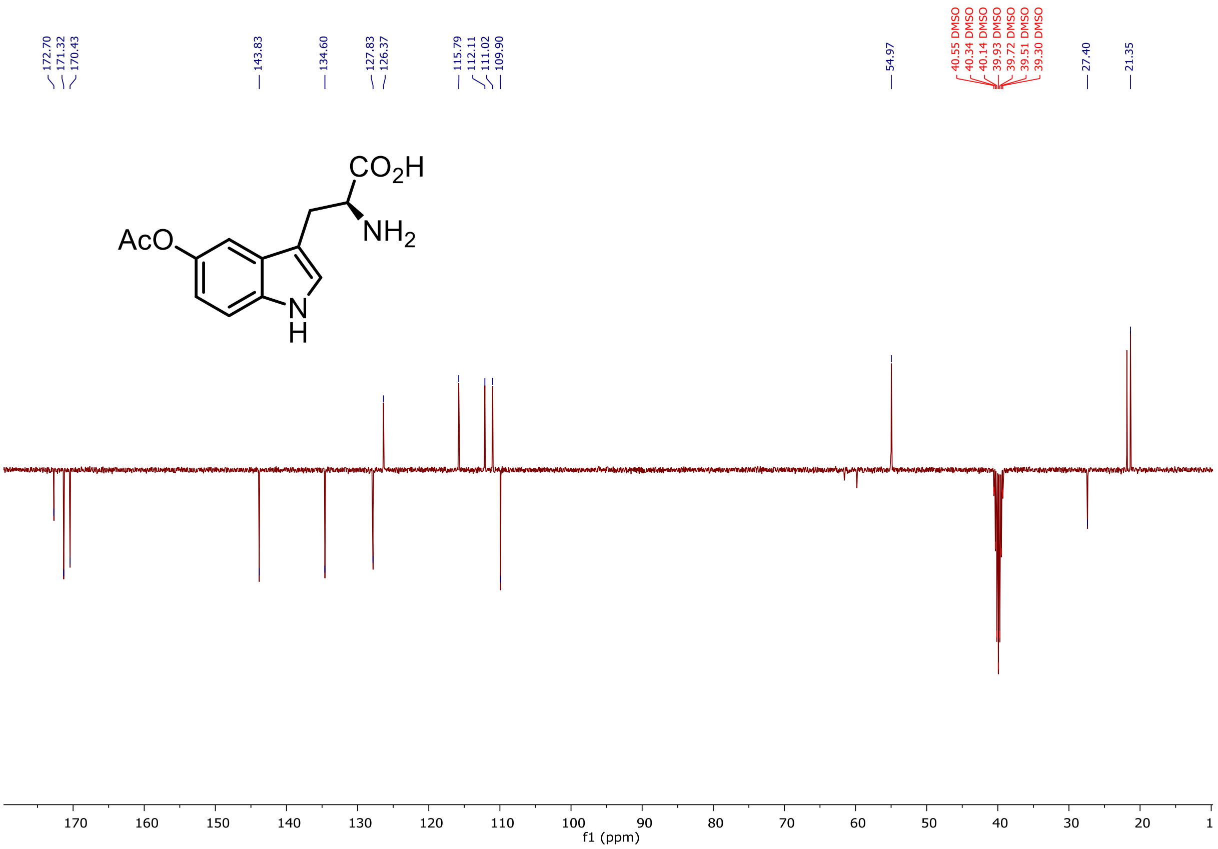

3.1.31 ^{1}H-NMR of (*S*)-3-(5-acetoxy-1*H*-indol-3-yl)-2-[(*tert*-butoxycarbonyl)amino]propanoic acid (16)

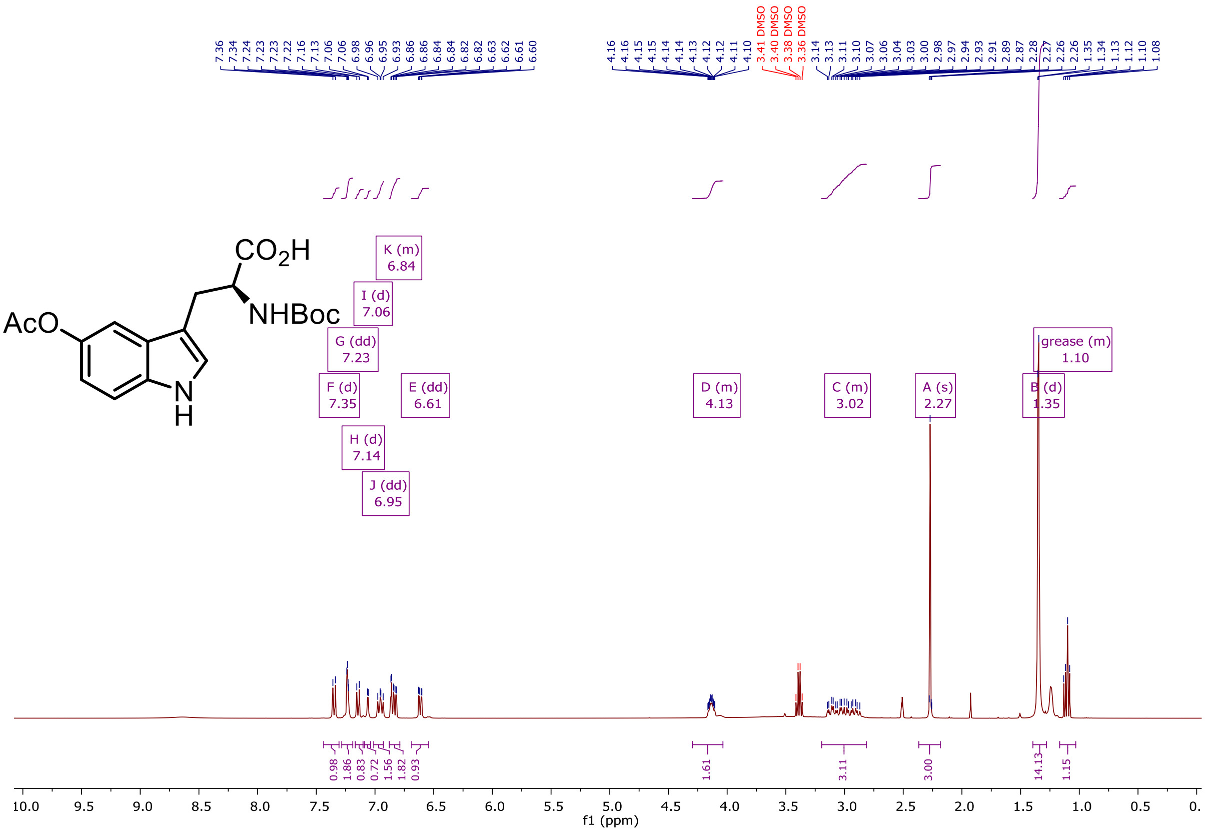

3.1.32 ^{13}C-NMR of (*S*)-3-(5-acetoxy-1*H*-indol-3-yl)-2-[(*tert*-butoxycarbonyl)amino]propanoic acid (16)

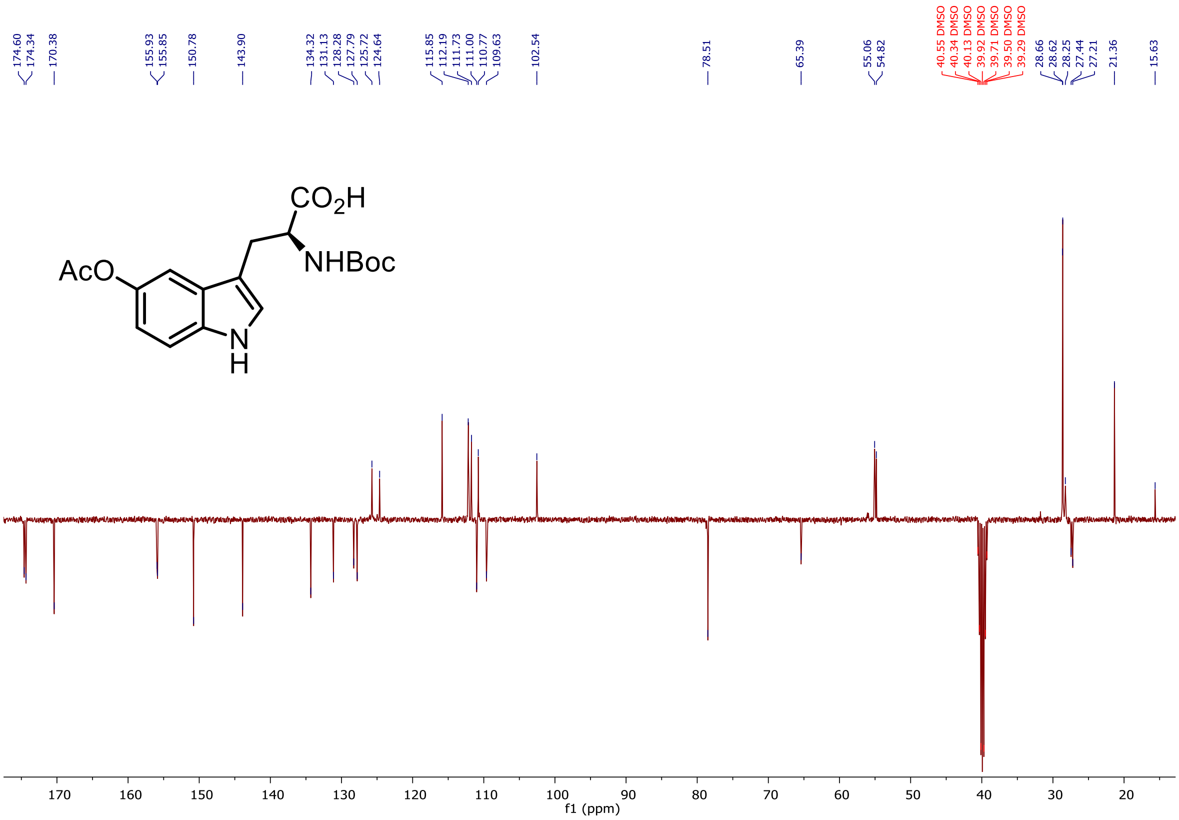

3.1.33 ^{1}H-NMR of *tert*-butyl (*S*)-3-(5-acetoxy-1*H*-indol-3-yl)-2-[(*tert*-butoxycarbonyl)amino]propanoate (17)

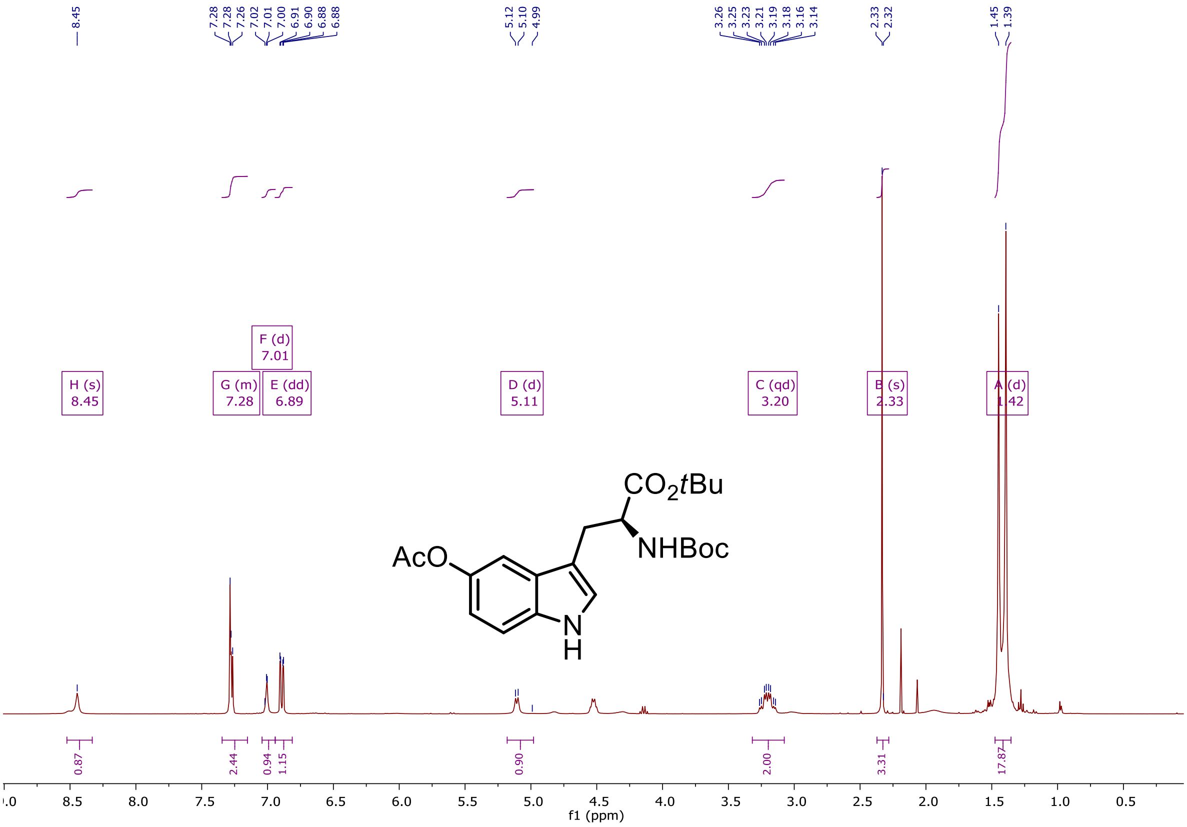

3.1.34 ^{13}C-NMR of *tert*-butyl (*S*)-3-(5-acetoxy-1*H*-indol-3-yl)-2-[(*tert*-butoxycarbonyl)amino]propanoate (17)

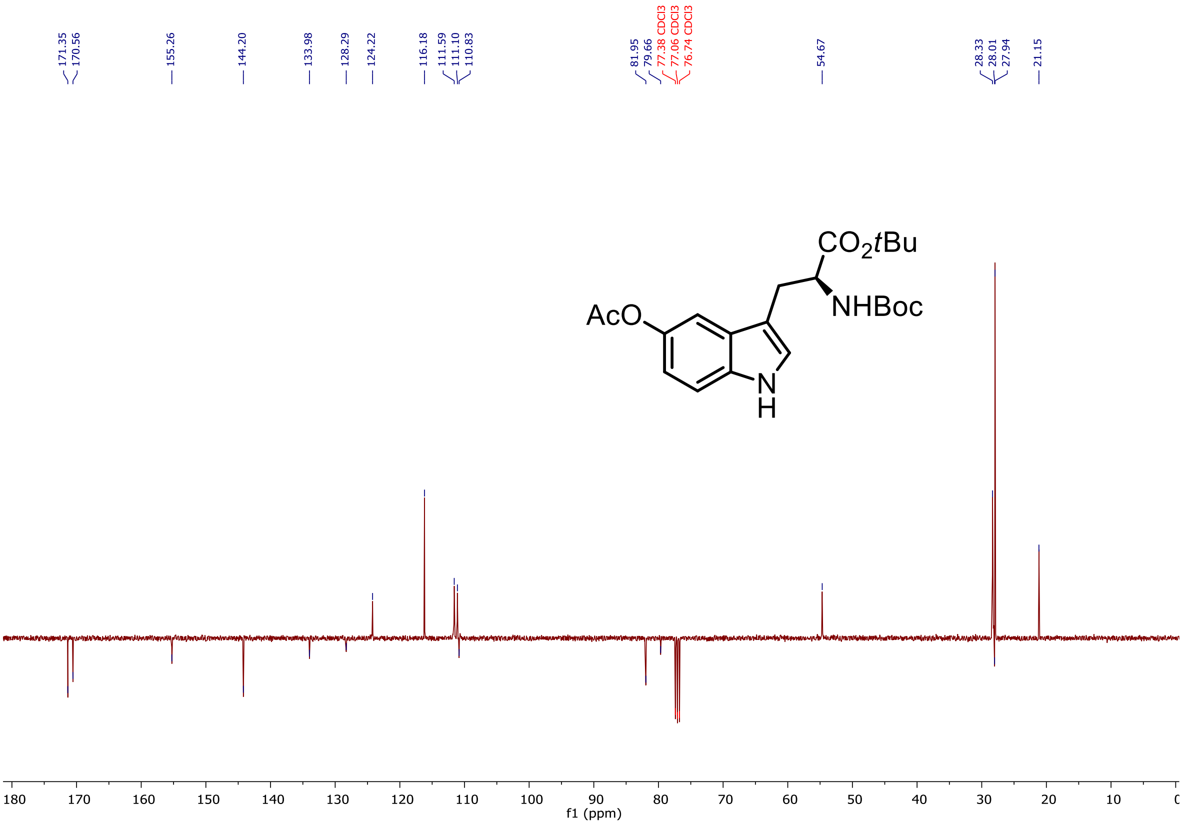

3.1.35 ^{1}H-NMR of *tert*-butyl (*S*)-3-[5-acetoxy-2,7-bis(4,4,5,5-tetramethyl-1,3,2-dioxaborolan-2-yl)-1*H*-indol-3-yl]-2-[(*tert*-butoxycarbonyl)amino]propanoate (18)

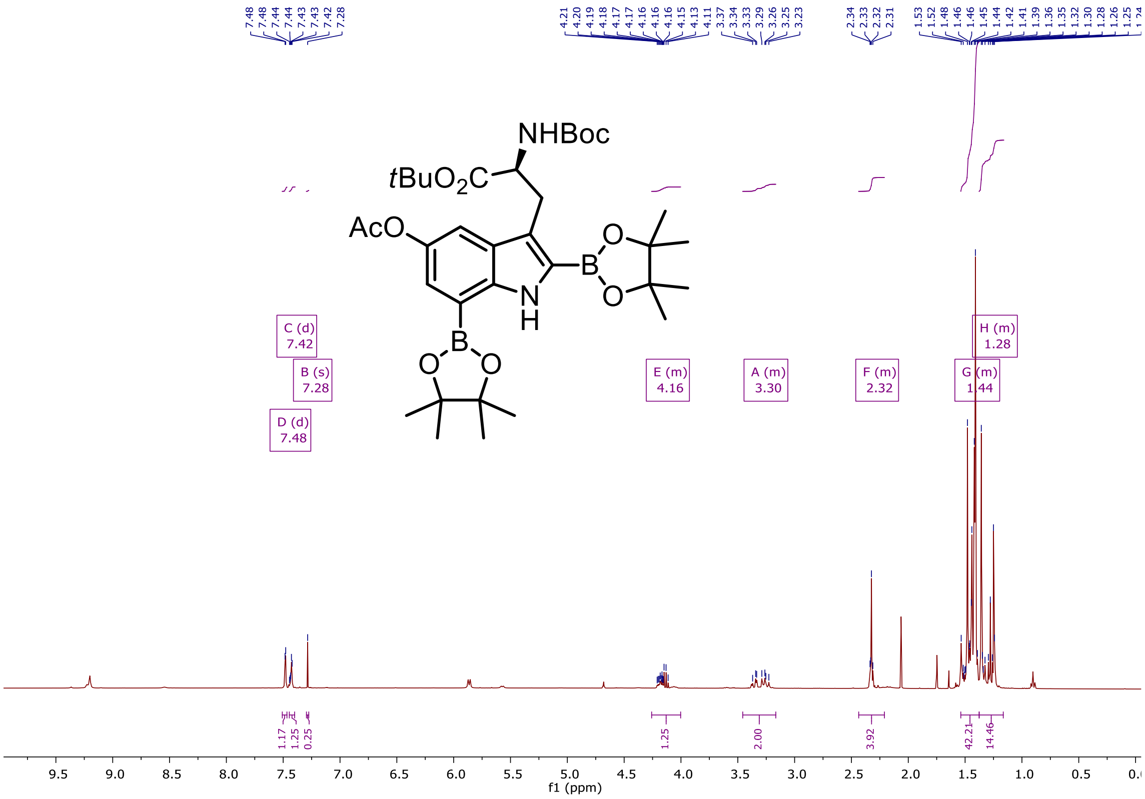

3.1.36 ^{13}C-NMR of *tert*-butyl (*S*)-3-[5-acetoxy-2,7-bis(4,4,5,5-tetramethyl-1,3,2-dioxaborolan-2-yl)-1*H*-indol-3-yl]-2-[(*tert*-butoxycarbonyl)amino]propanoate (18)

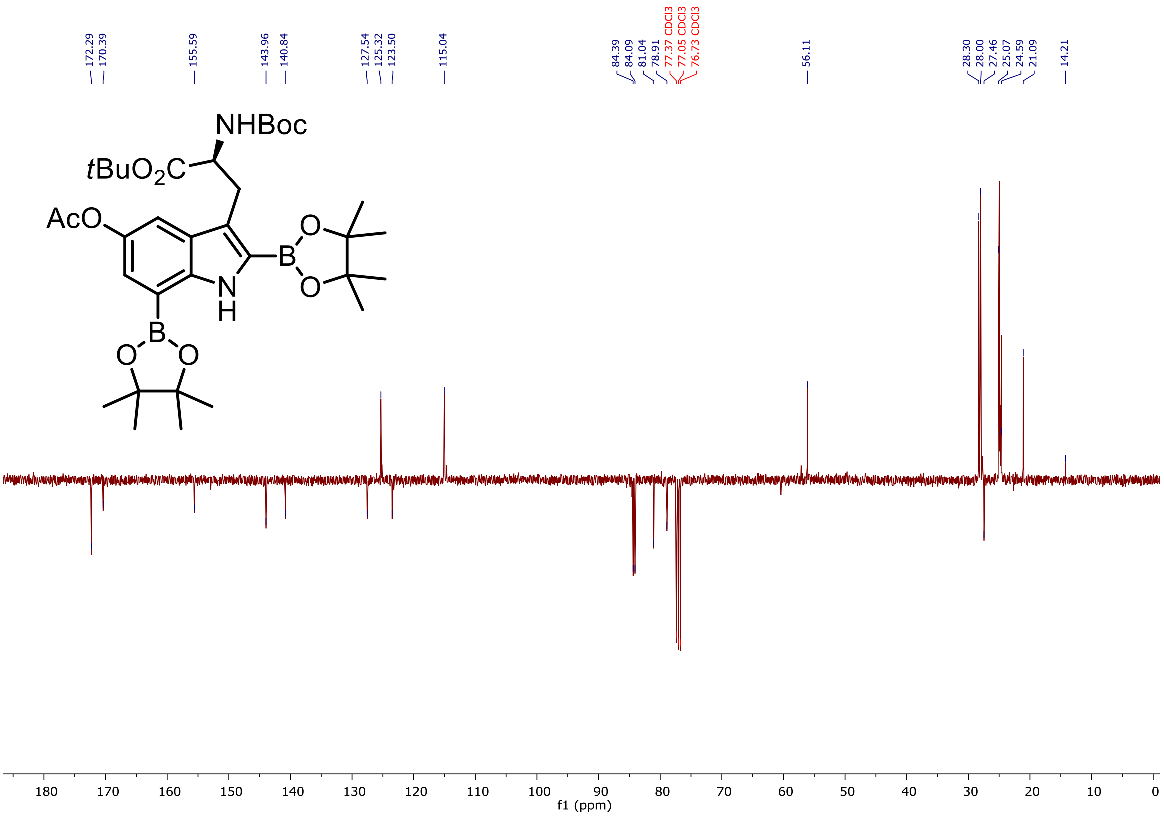

3.1.37 ^{1}H-NMR of *tert*-butyl (*S*)-3-[5-acetoxy-7-(4,4,5,5-tetramethyl-1,3,2-dioxaborolan-2-yl)-1*H*-indol-3-yl]-2-[(*tert*-butoxycarbonyl)amino]propanoate (19)

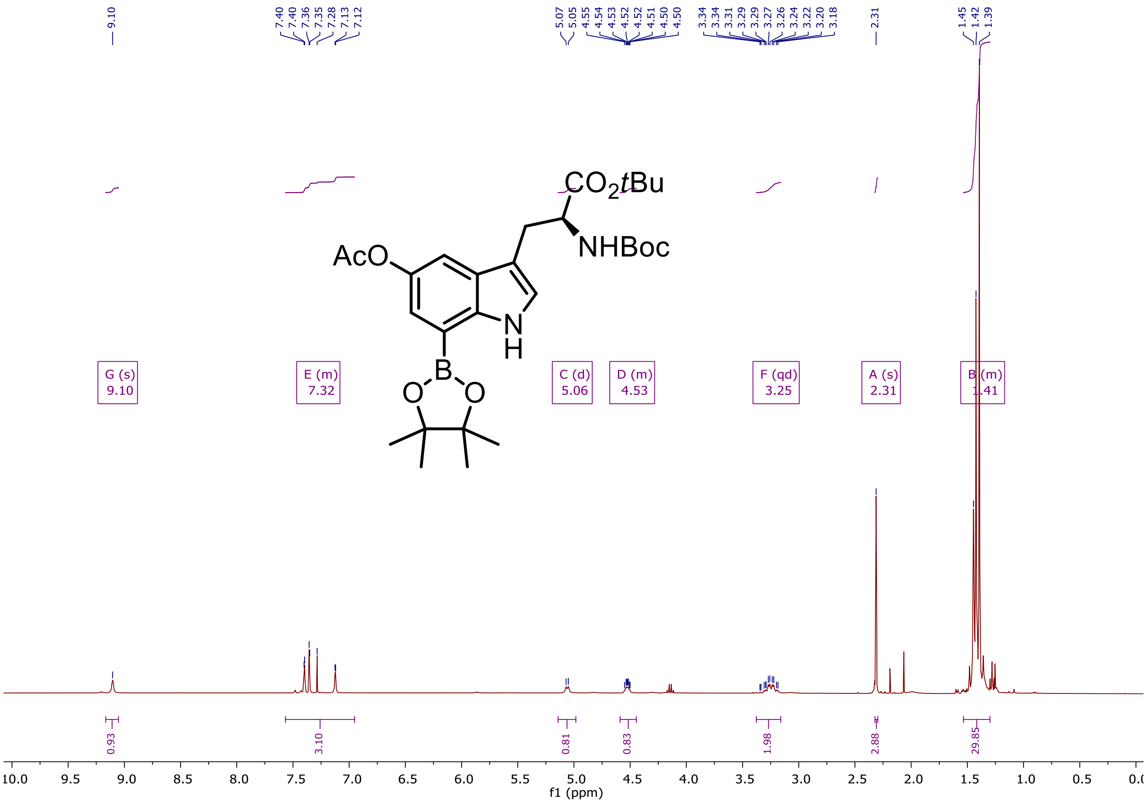

3.1.38 ^{13}C-NMR of *tert*-butyl (*S*)-3-[5-acetoxy-7-(4,4,5,5-tetramethyl-1,3,2-dioxaborolan-2-yl)-1*H*-indol-3-yl]-2-[(*tert*-butoxycarbonyl)amino]propanoate (19)

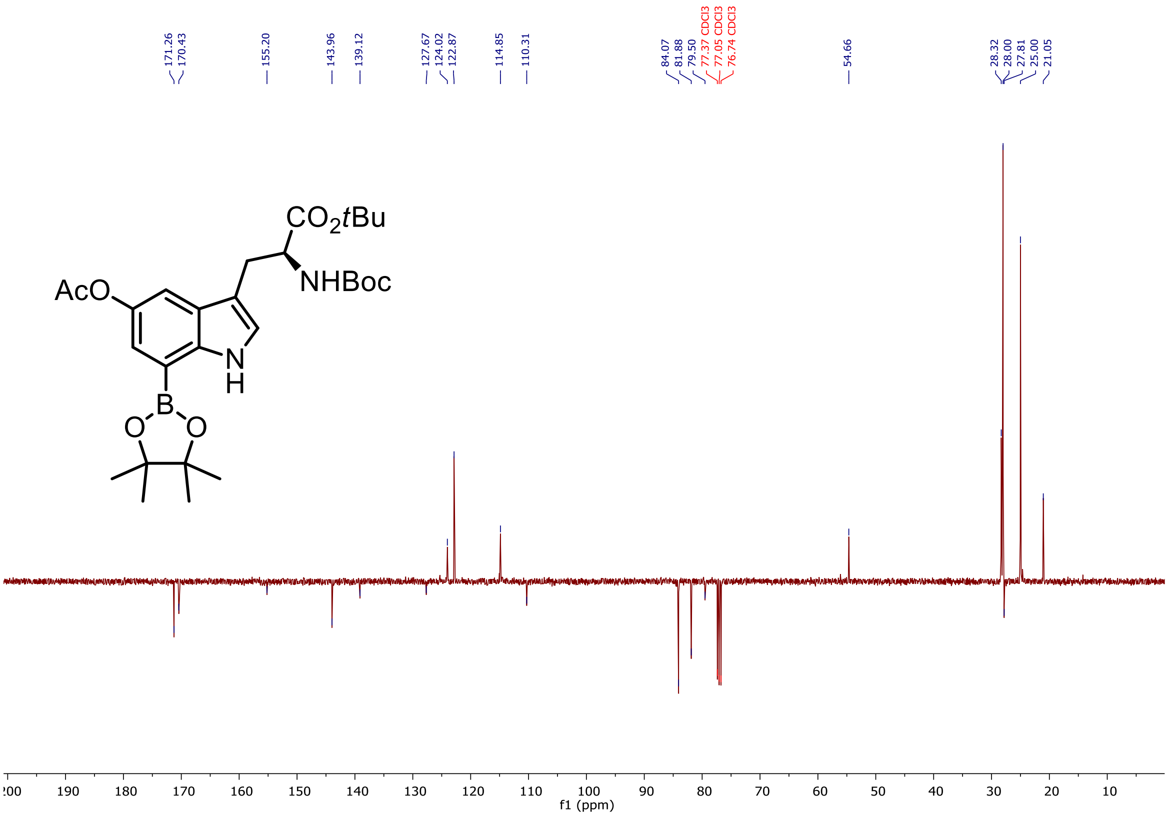

3.1.39 ¹H-NMR of 7-fluoroindoline (21)

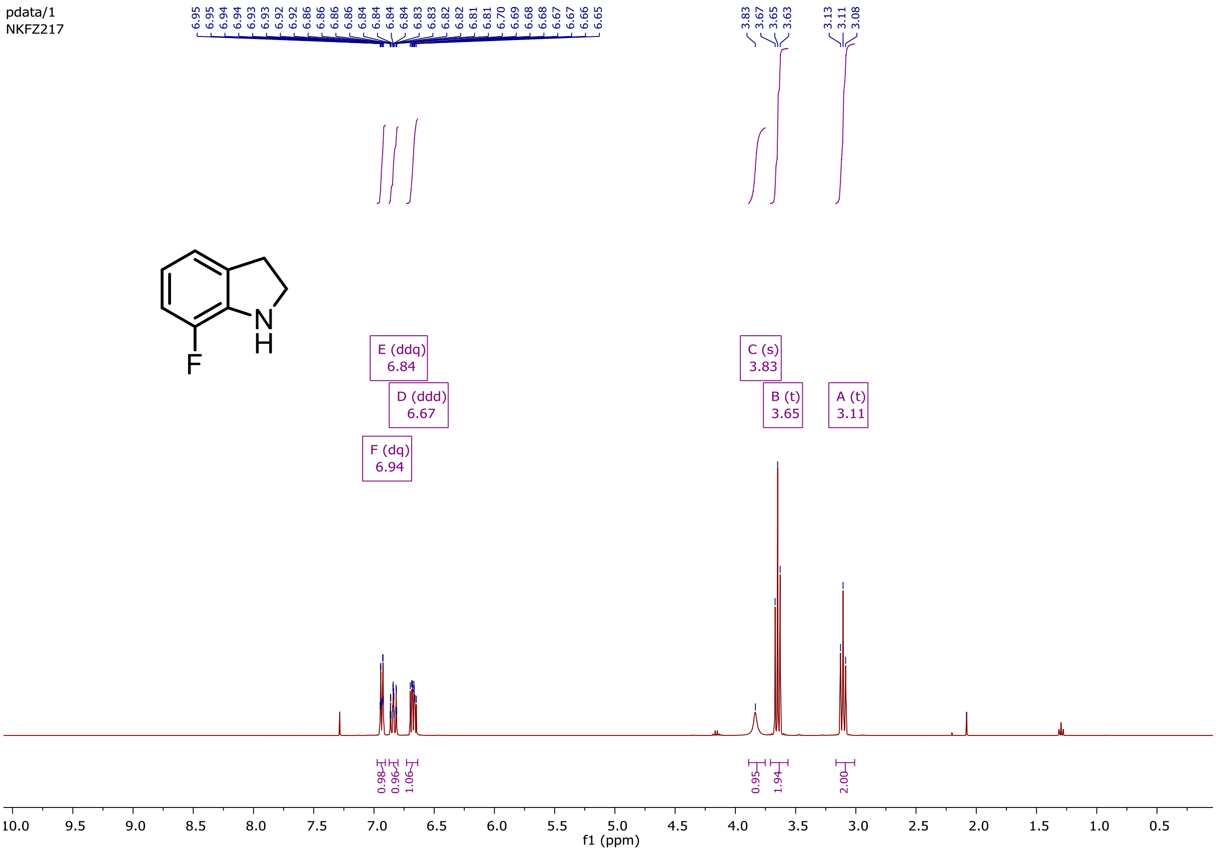

3.1.40 ¹³C-NMR of 7-fluoroindoline (21)

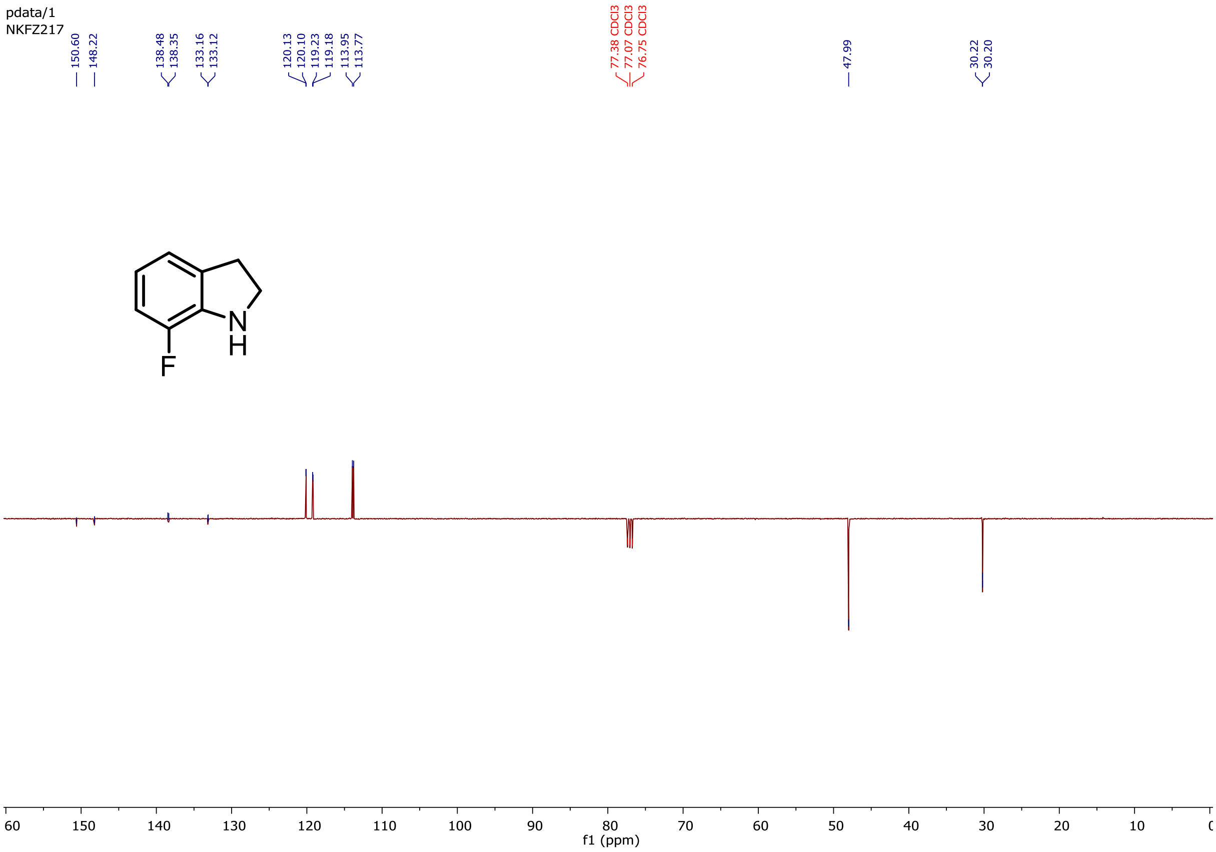

3.1.41 ^{19}F-NMR of 7-fluoroindoline (21)

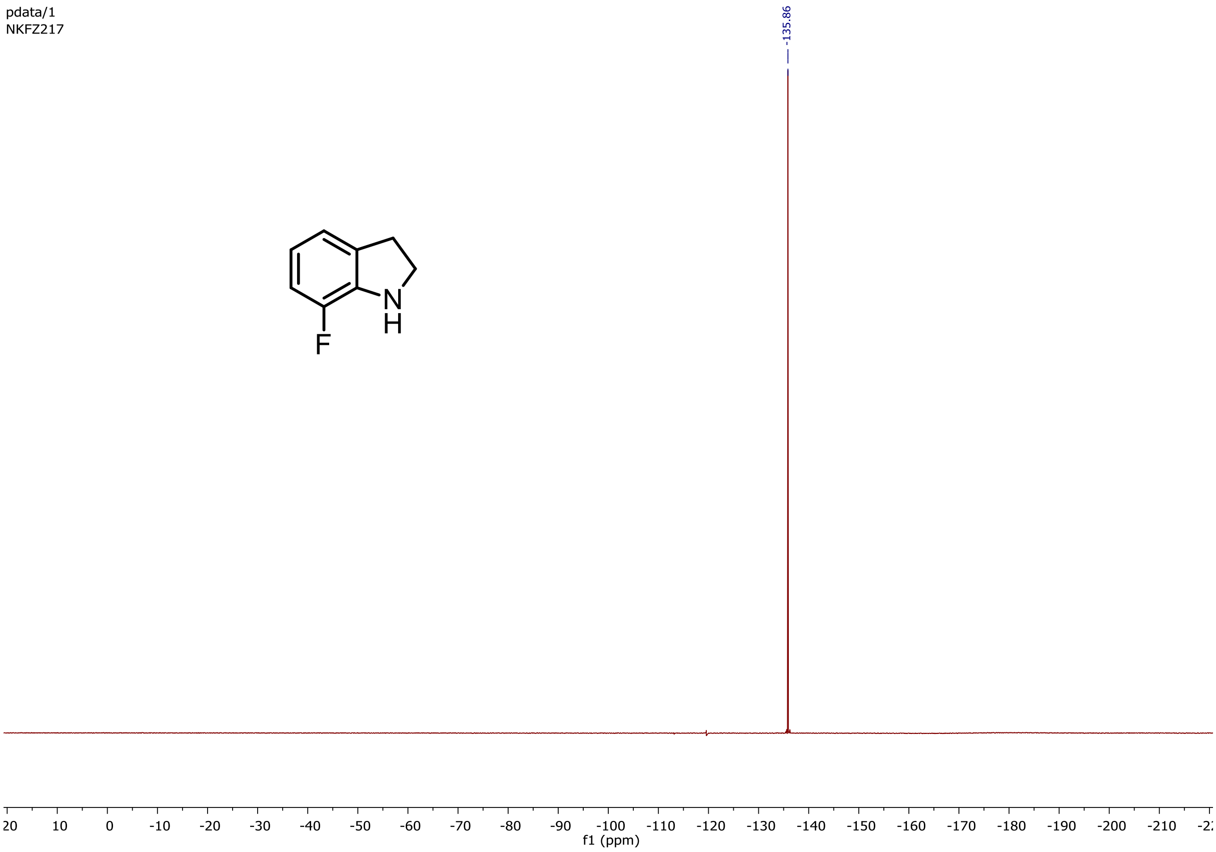

3.1.42 ^{1}H-NMR of 5-hydroxy-7-fluoroindole (22)

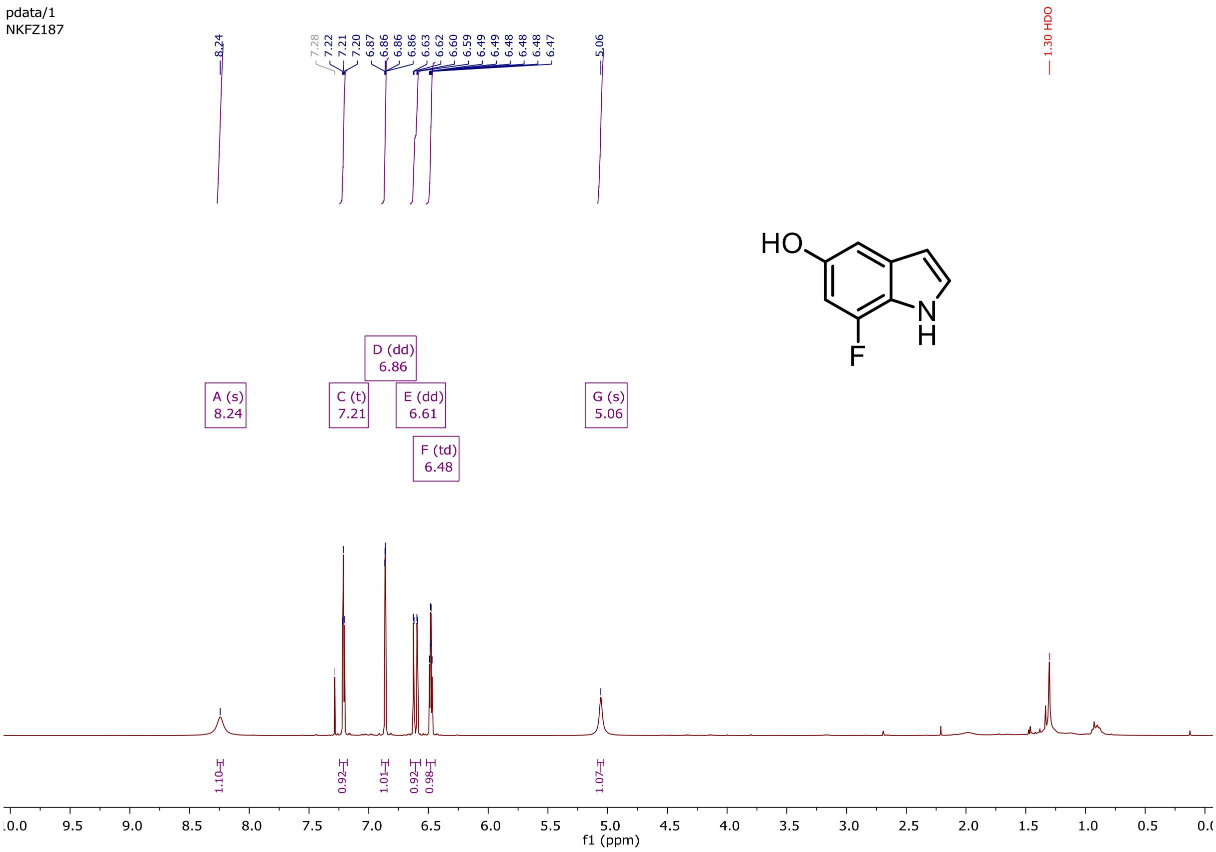

3.1.43 ^{13}C-NMR of 5-hydroxy-7-fluoroindole (22)

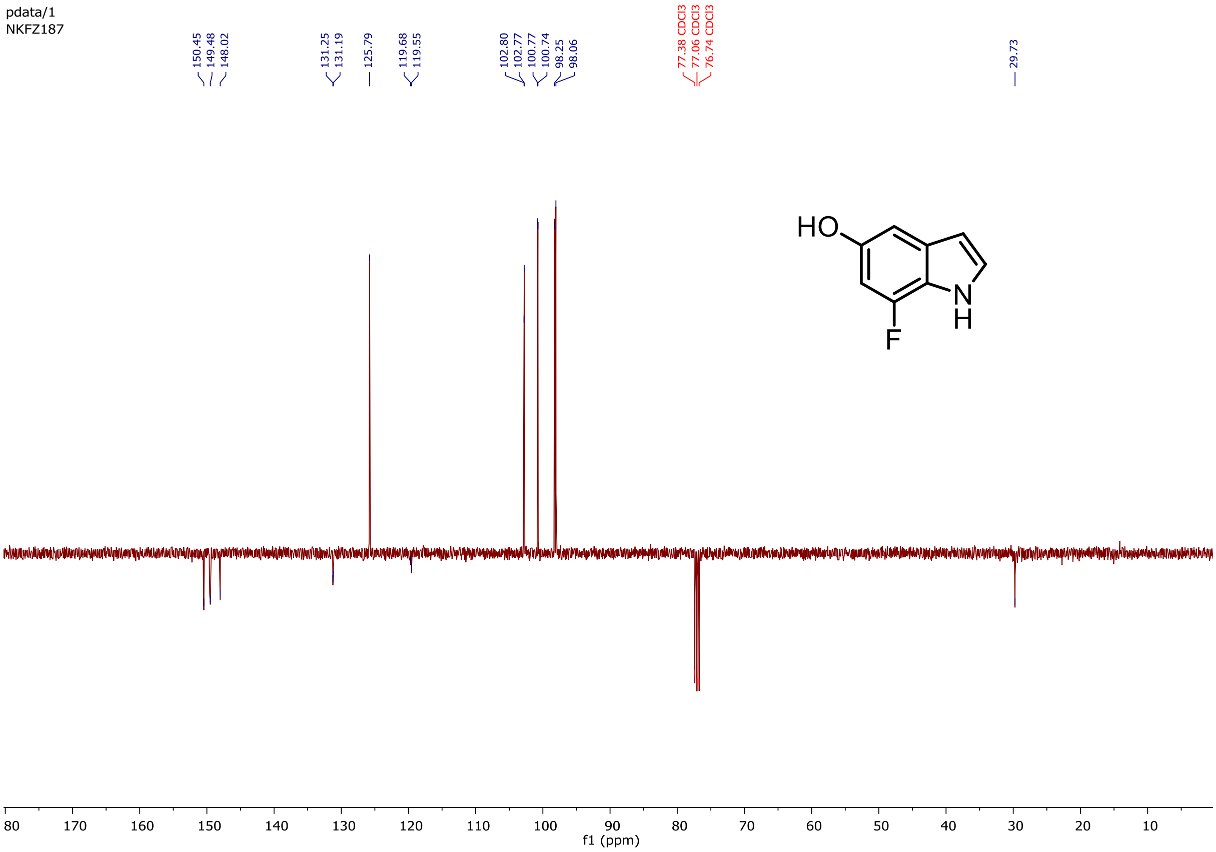

3.1.44 ^{19}F-NMR of 5-hydroxy-7-fluoroindole (22)

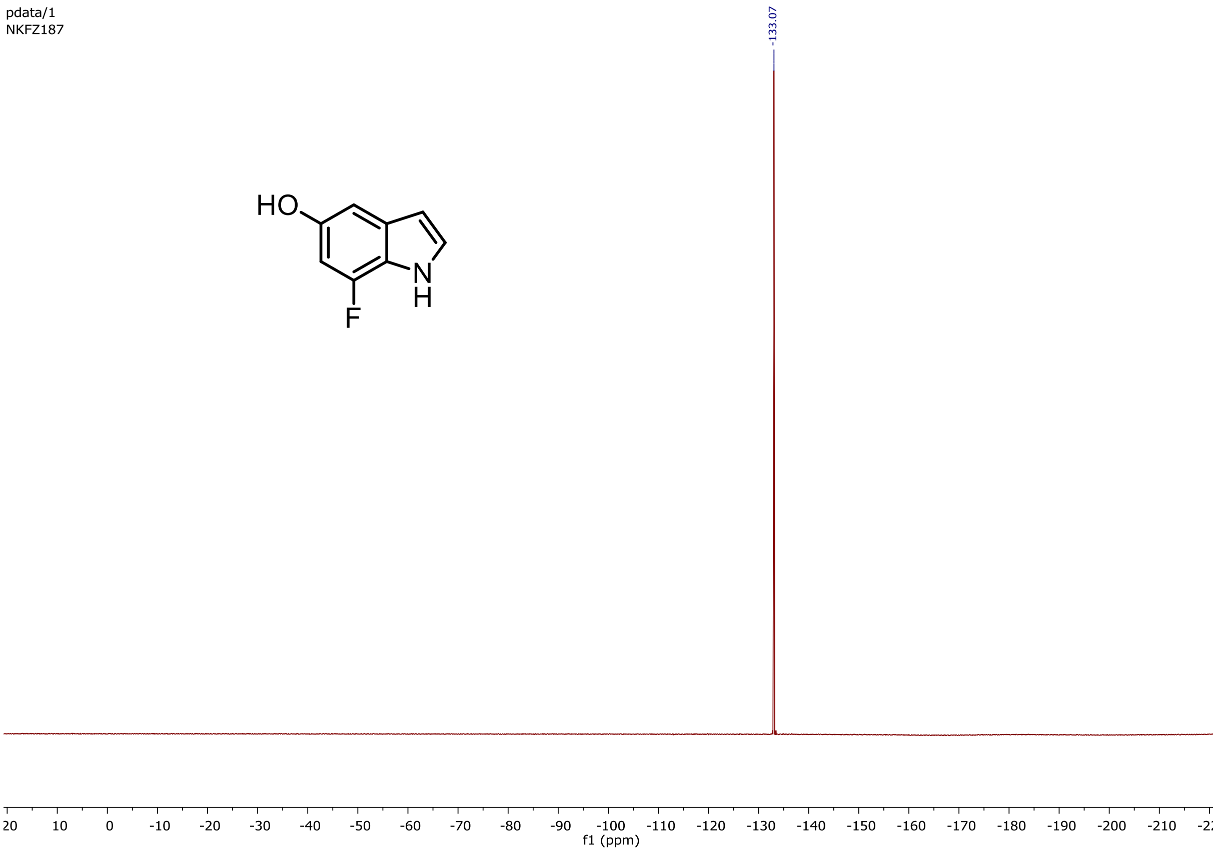

3.1.45 ^{1}H-NMR of 5-acetoxy-7-fluoroindole (23)

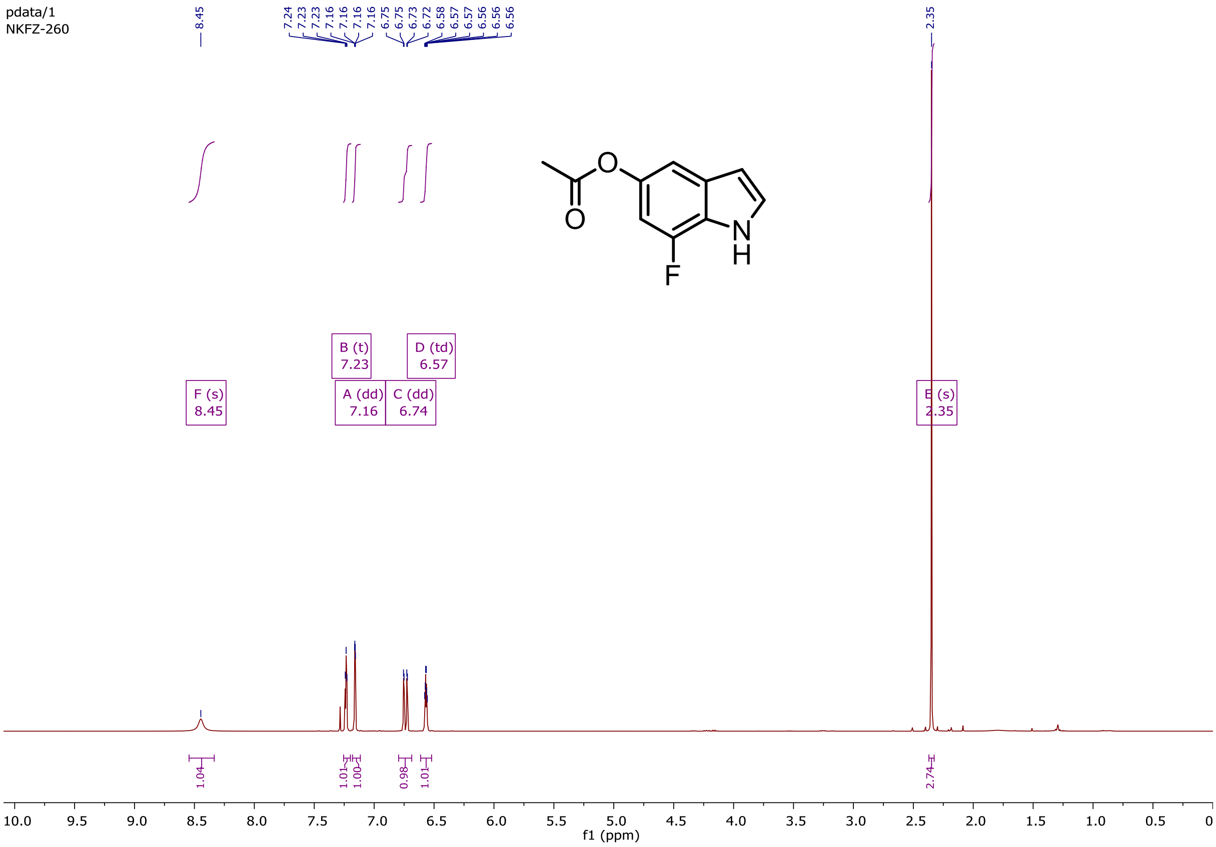

3.1.46 ^{13}C-NMR of 5-acetoxy-7-fluoroindole (23)

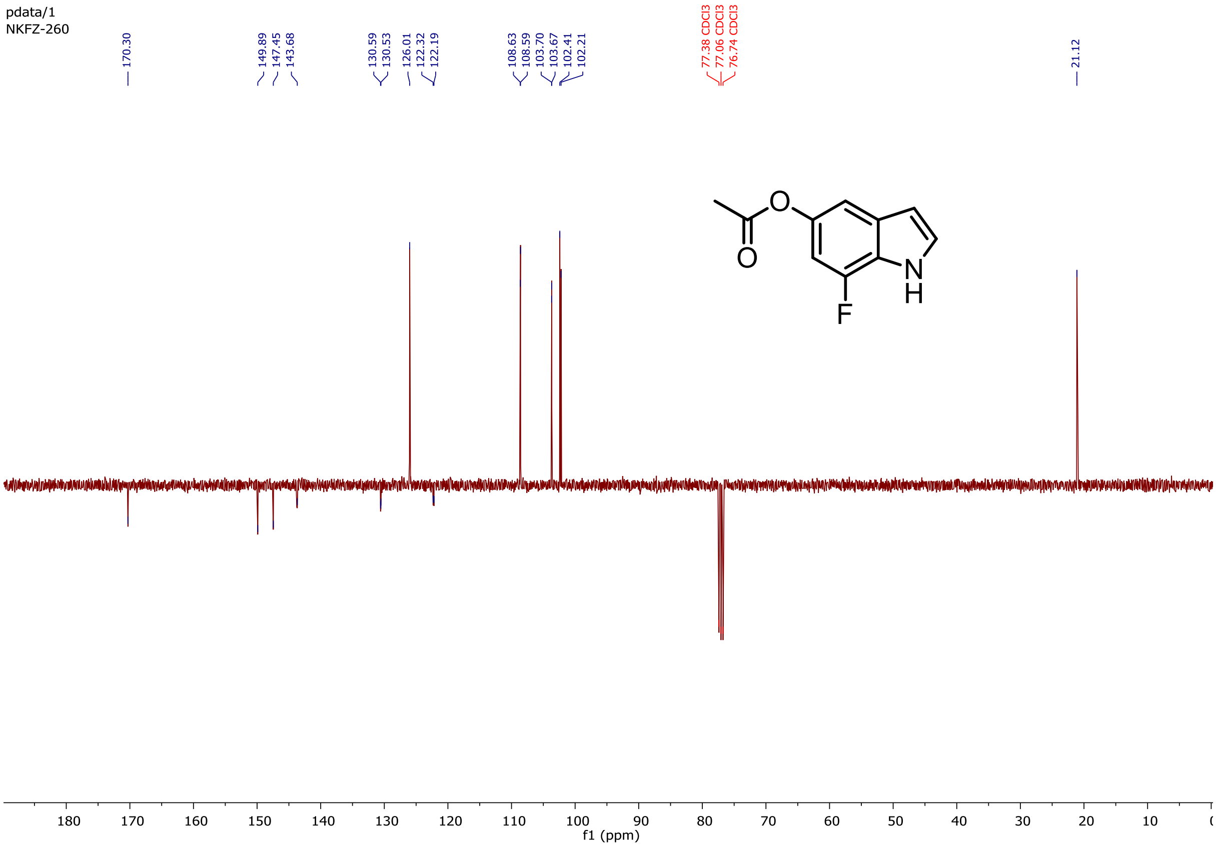

3.1.47 ^{19}F-NMR of 5-acetoxy-7-fluoroindole (23)

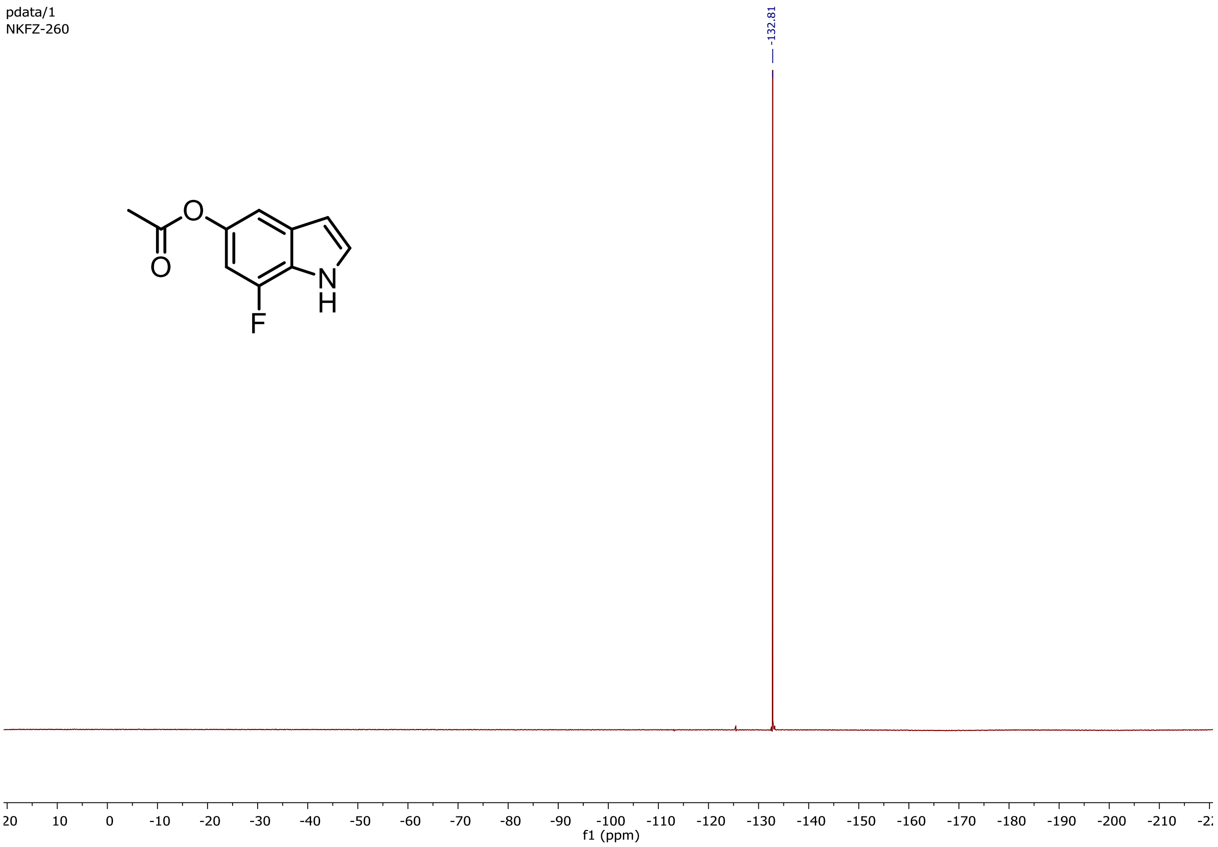

3.1.48 ^{1}H-NMR of methyl 2-acetamido-3-(5-acetoxy-7-fluoro-1*H*-indol-3-yl)propanoate (24)

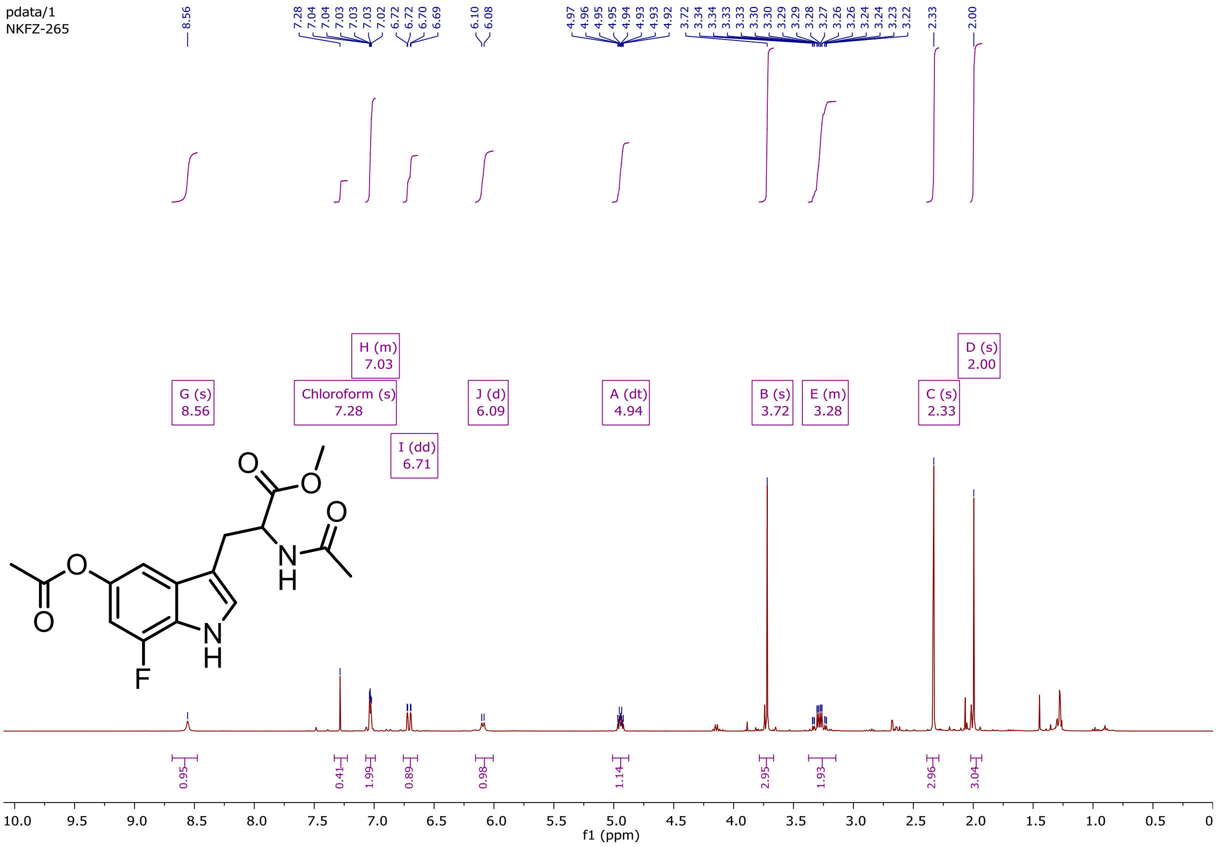

3.1.49 ^{13}C-NMR of methyl 2-acetamido-3-(5-acetoxy-7-fluoro-1*H*-indol-3-yl)propanoate (24)

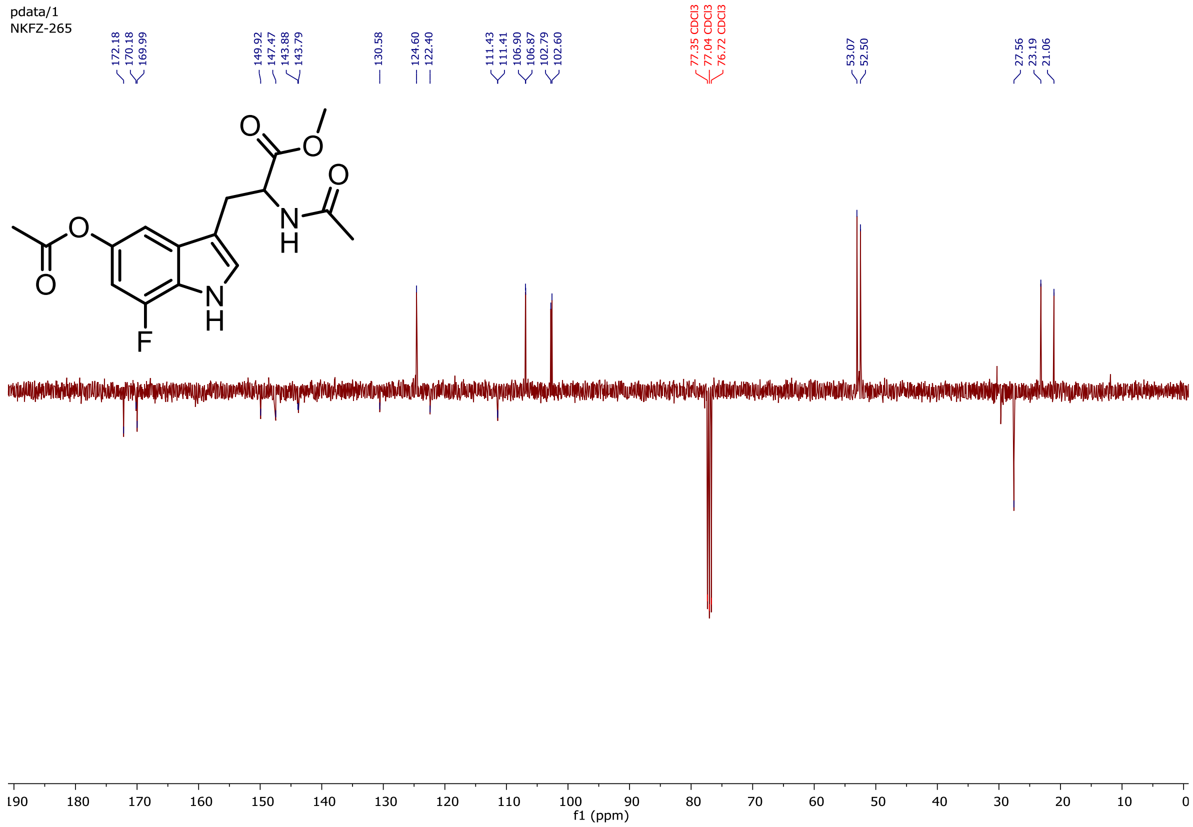

3.1.50 ^{19}F-NMR of methyl 2-acetamido-3-(5-acetoxy-7-fluoro-1*H*-indol-3-yl)propanoate (24)

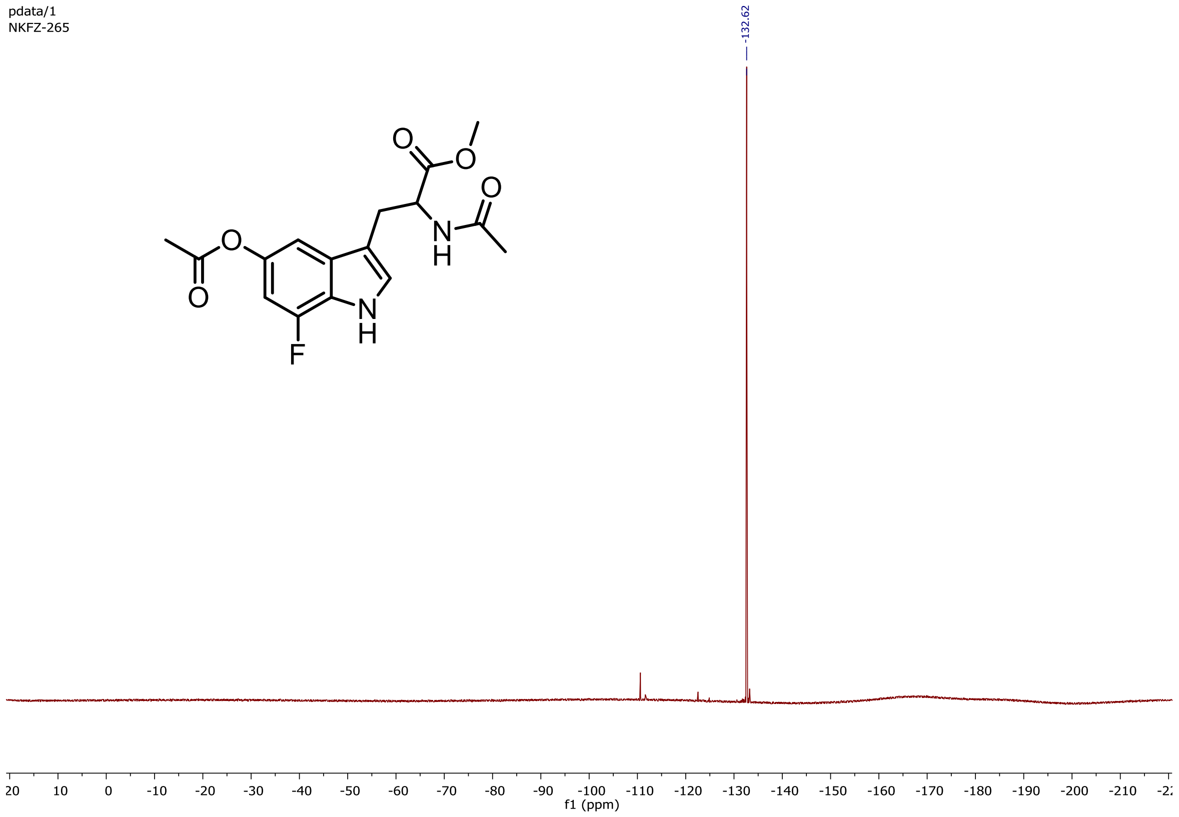

3.1.51 ^{1}H-NMR of *tert*-butyl 5-acetoxy-3-{2-[*N*-(*tert*-butoxycarbonyl)acetamido]-3-methoxy-3-oxopropyl}-7-fluoro-1*H*-indole-1-carboxylate (25)

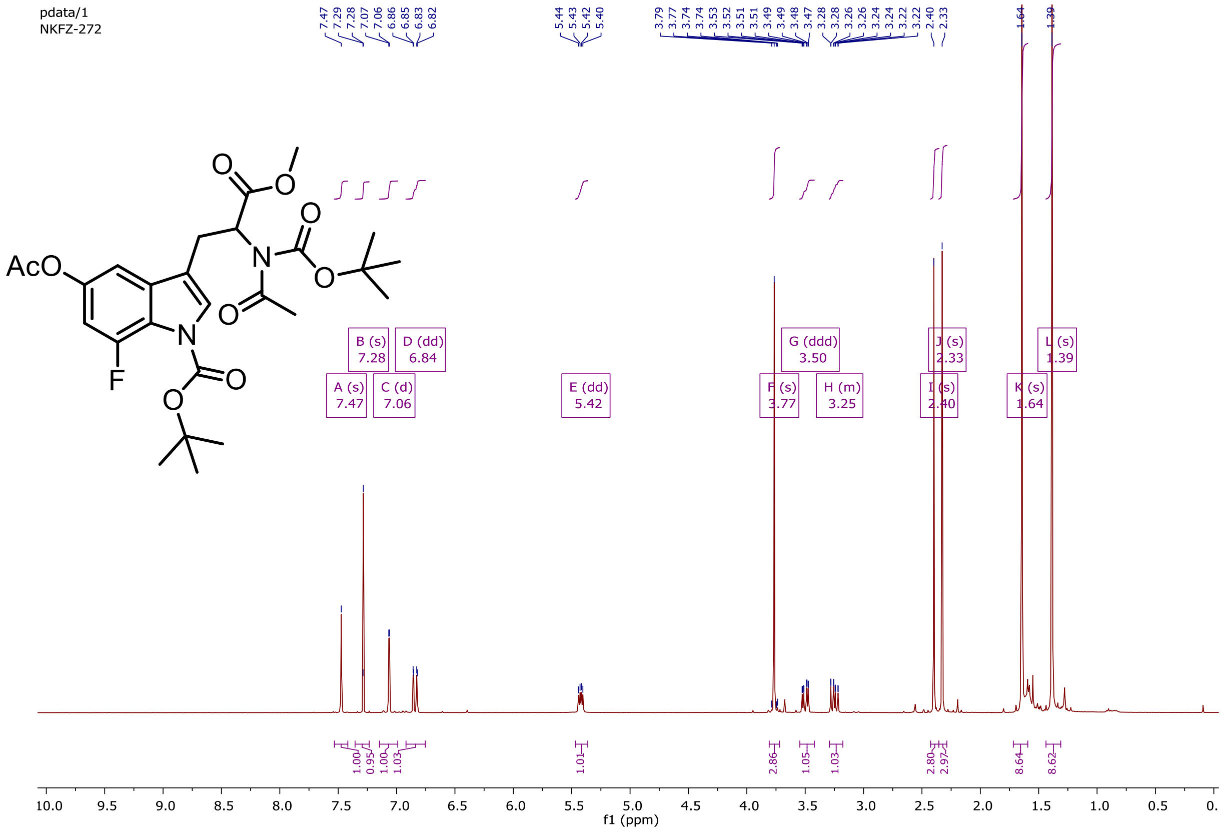

3.1.52 ^{13}C-NMR of *tert*-butyl 5-acetoxy-3-{2-[*N*-(*tert*-butoxycarbonyl)acetamido]-3-methoxy-3-oxopropyl}-7-fluoro-1*H*-indole-1-carboxylate (25)

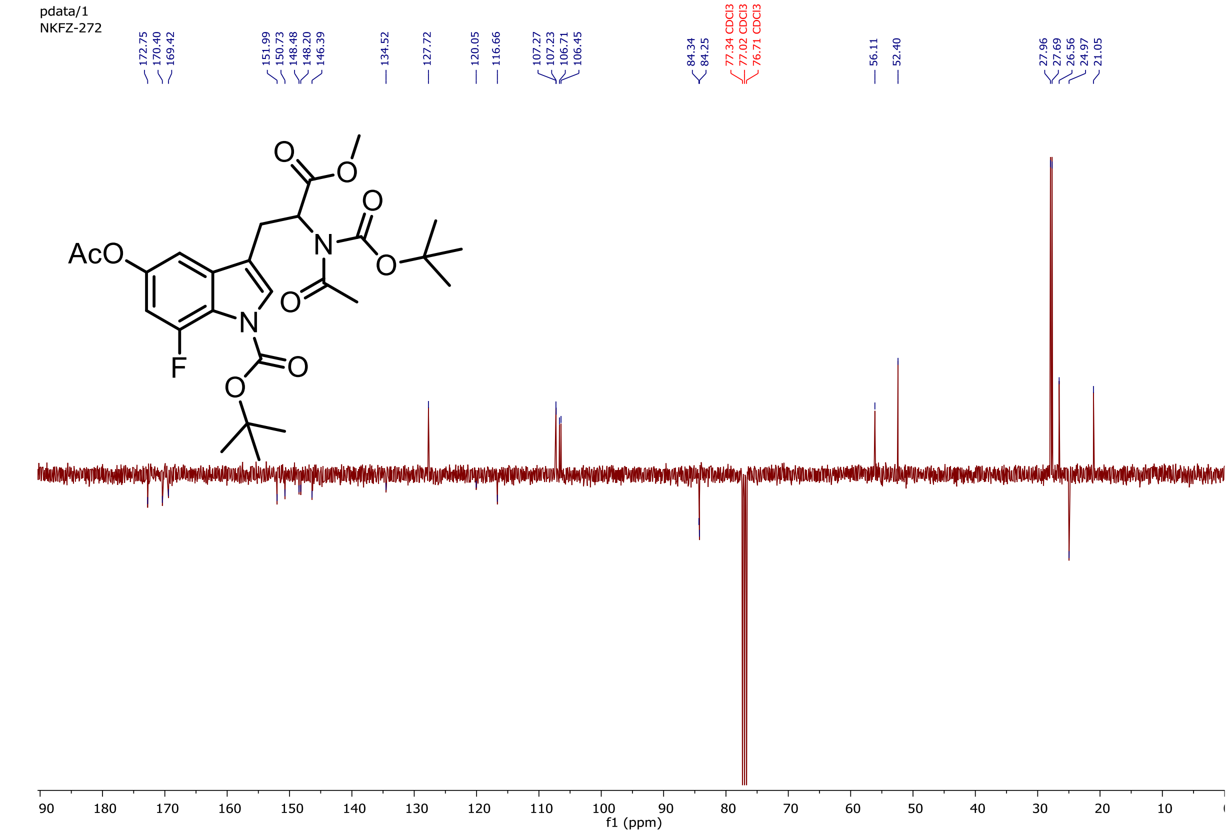

3.1.53 ^{19}F-NMR of *tert*-butyl 5-acetoxy-3-{2-[*N*-(*tert*-butoxycarbonyl)acetamido]-3-methoxy-3-oxopropyl}-7-fluoro-1*H*-indole-1-carboxylate (25)

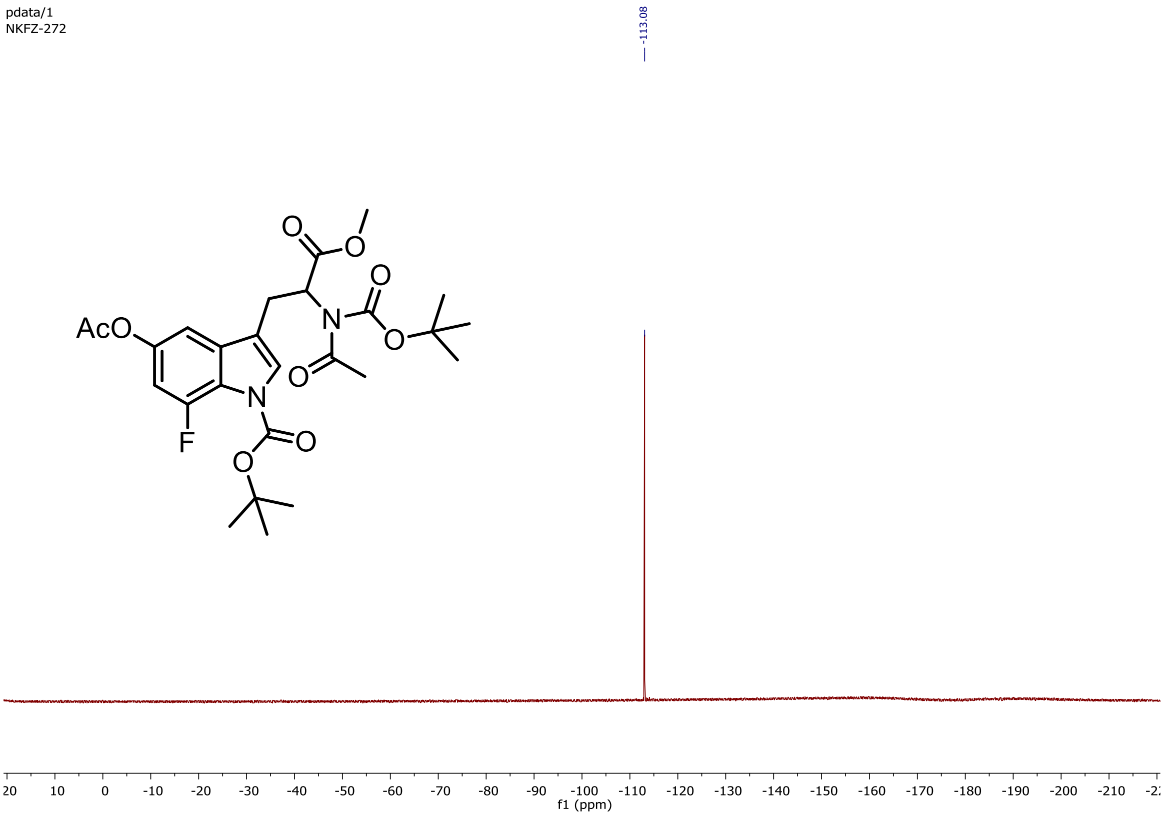

3.1.54 ^{1}H-NMR of 3-[1-(*tert*-butoxycarbonyl)-7-fluoro-5-hydroxy-1*H*-indol-3-yl]-2-[(*tert*-butoxycarbonyl)amino]propanoic acid (26)

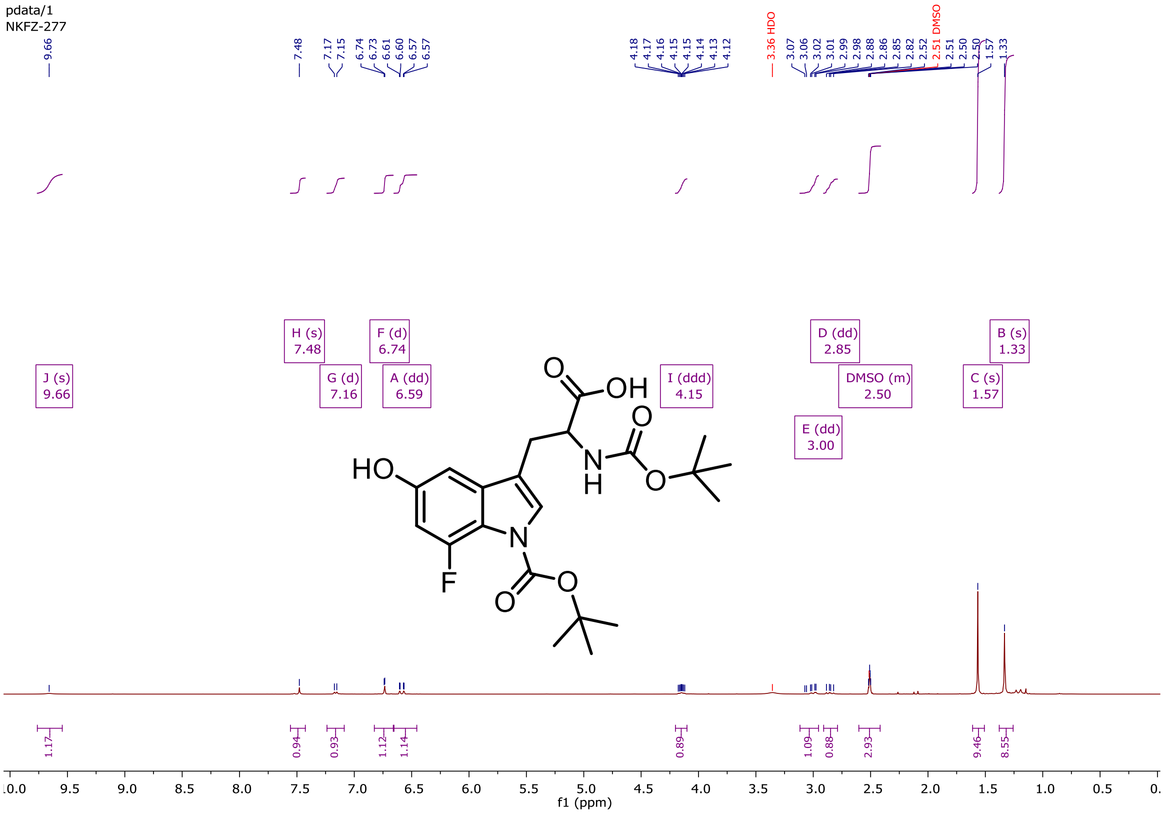

3.1.55 ^{13}C-NMR of 3-[1-(*tert*-butoxycarbonyl)-7-fluoro-5-hydroxy-1*H*-indol-3-yl]-2-[(*tert*-butoxycarbonyl)amino]propanoic acid (26)

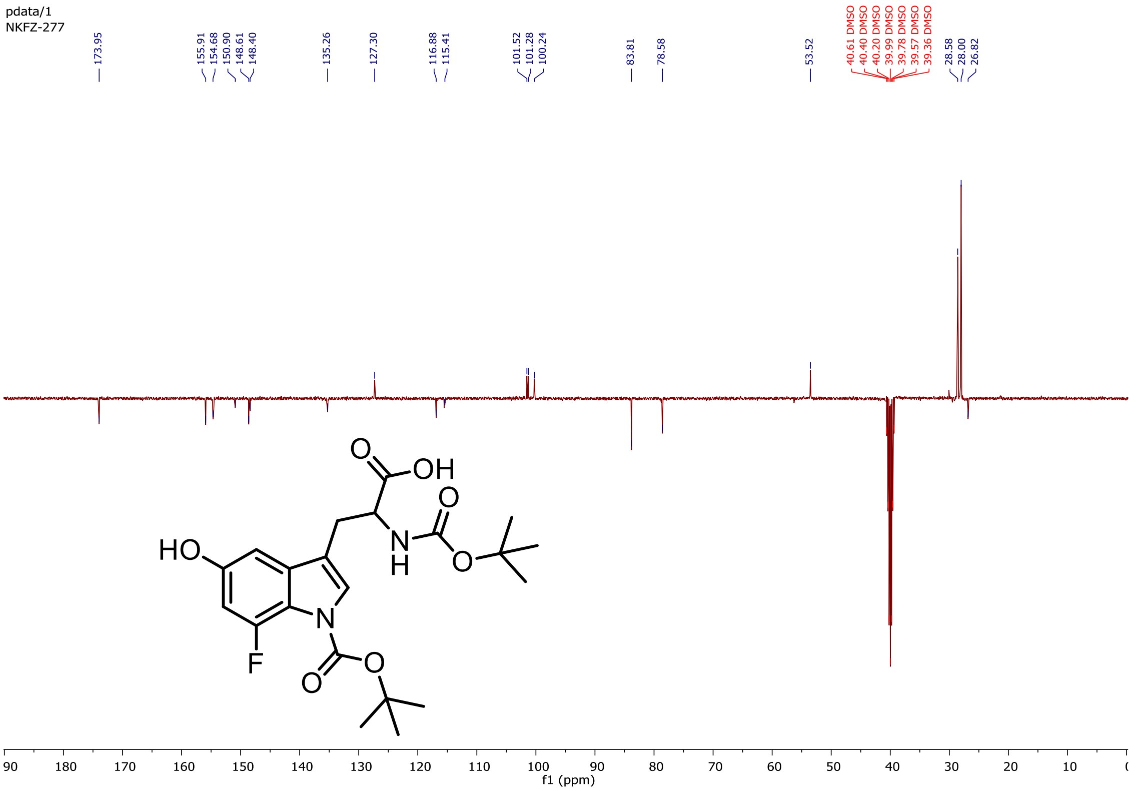

3.1.56 ^{19}F-NMR of 3-[1-(*tert*-butoxycarbonyl)-7-fluoro-5-hydroxy-1*H*-indol-3-yl]-2-[(*tert*-butoxycarbonyl)amino]propanoic acid (26)

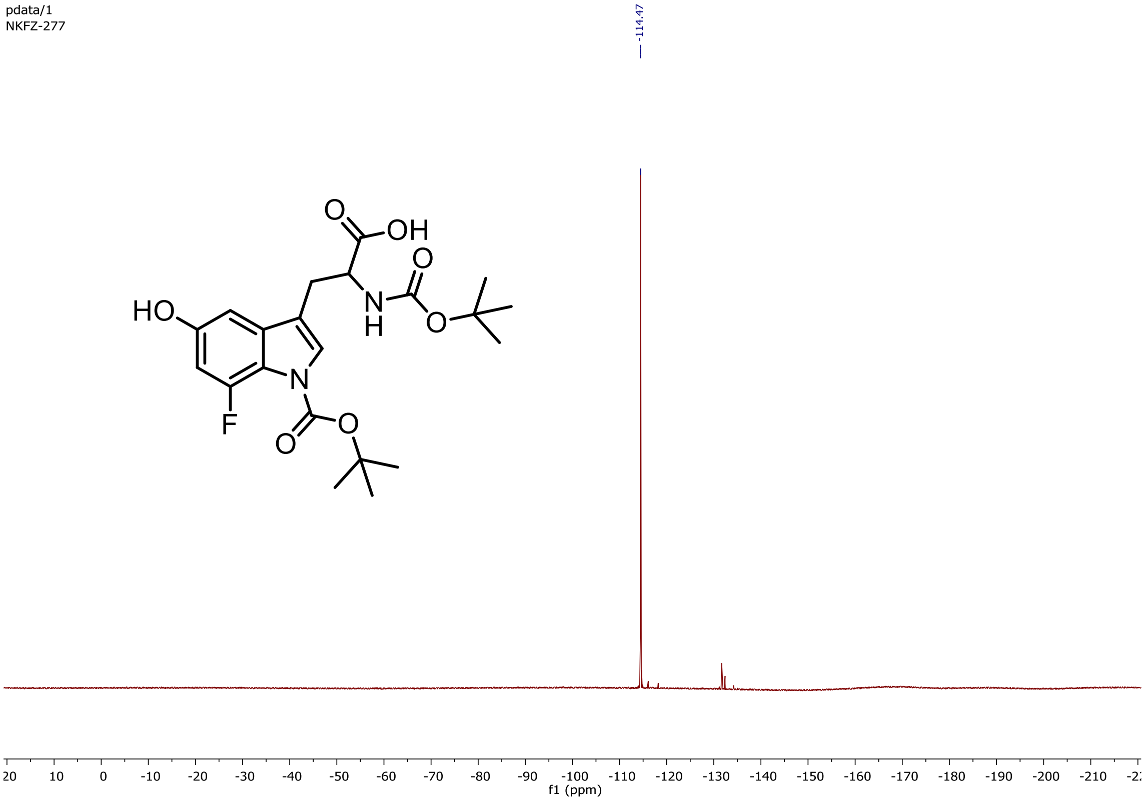

3.1.57 ^{1}H-NMR of 2-amino-3-(7-fluoro-5-hydroxy-1*H*-indol-3-yl)propanoic acid trifluoroacetate (27 × TFA)

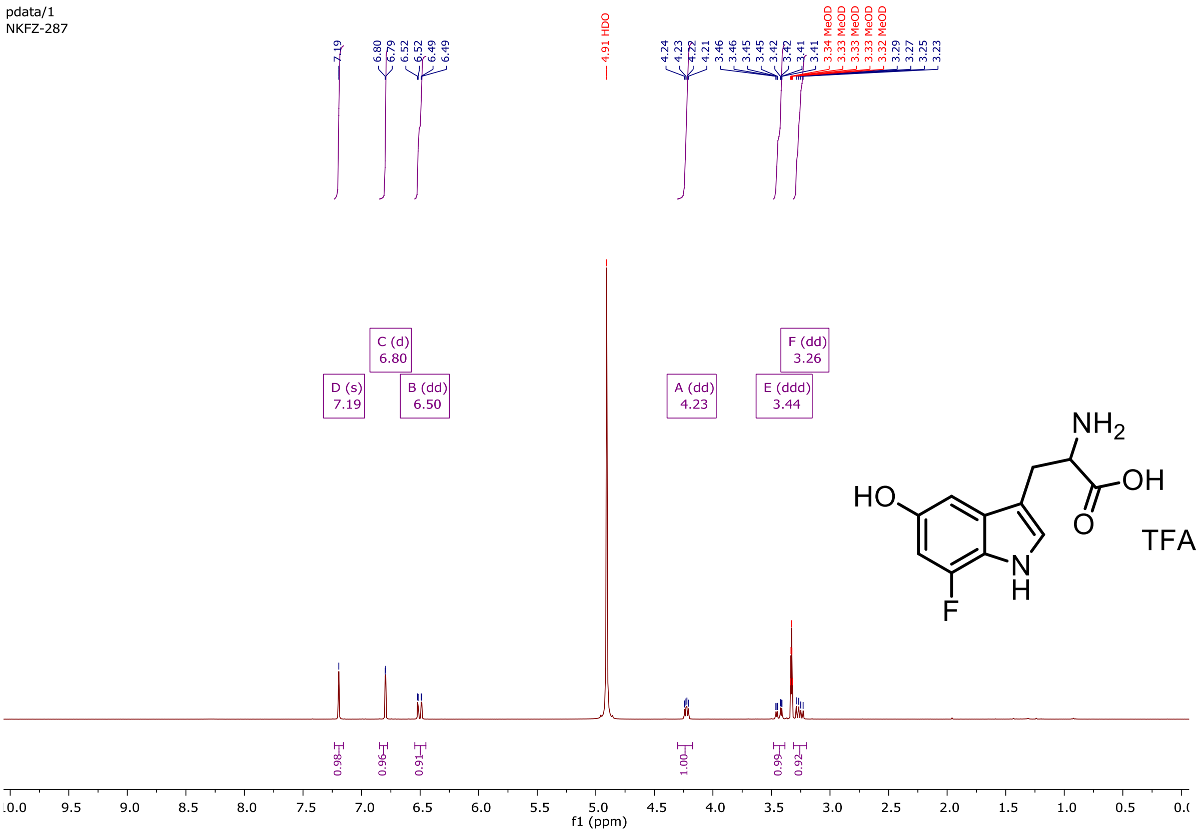

3.1.58 ^{13}C-NMR of 2-amino-3-(7-fluoro-5-hydroxy-1*H*-indol-3-yl)propanoic acid trifluoroacetate (27 × TFA)

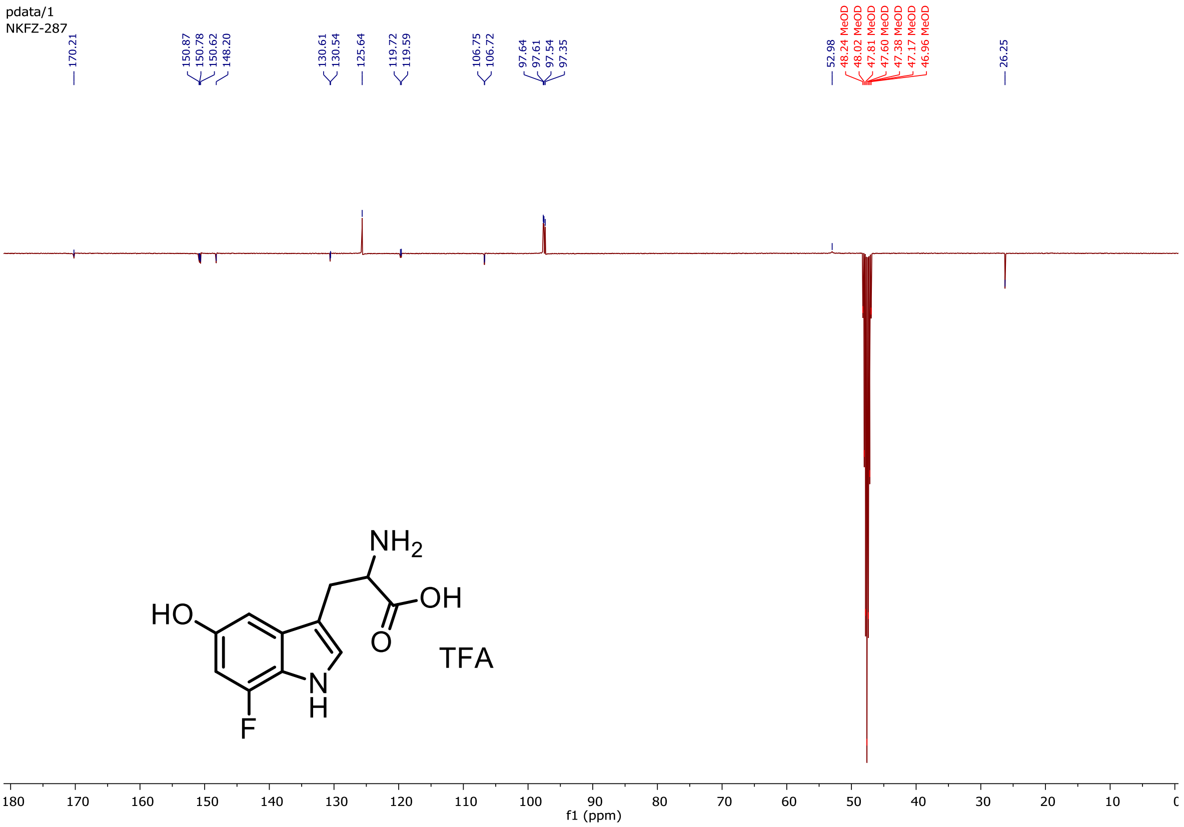

3.1.59 ^{19}F-NMR of 5-hydroxy-7-fluorotryptophan triflate (19)

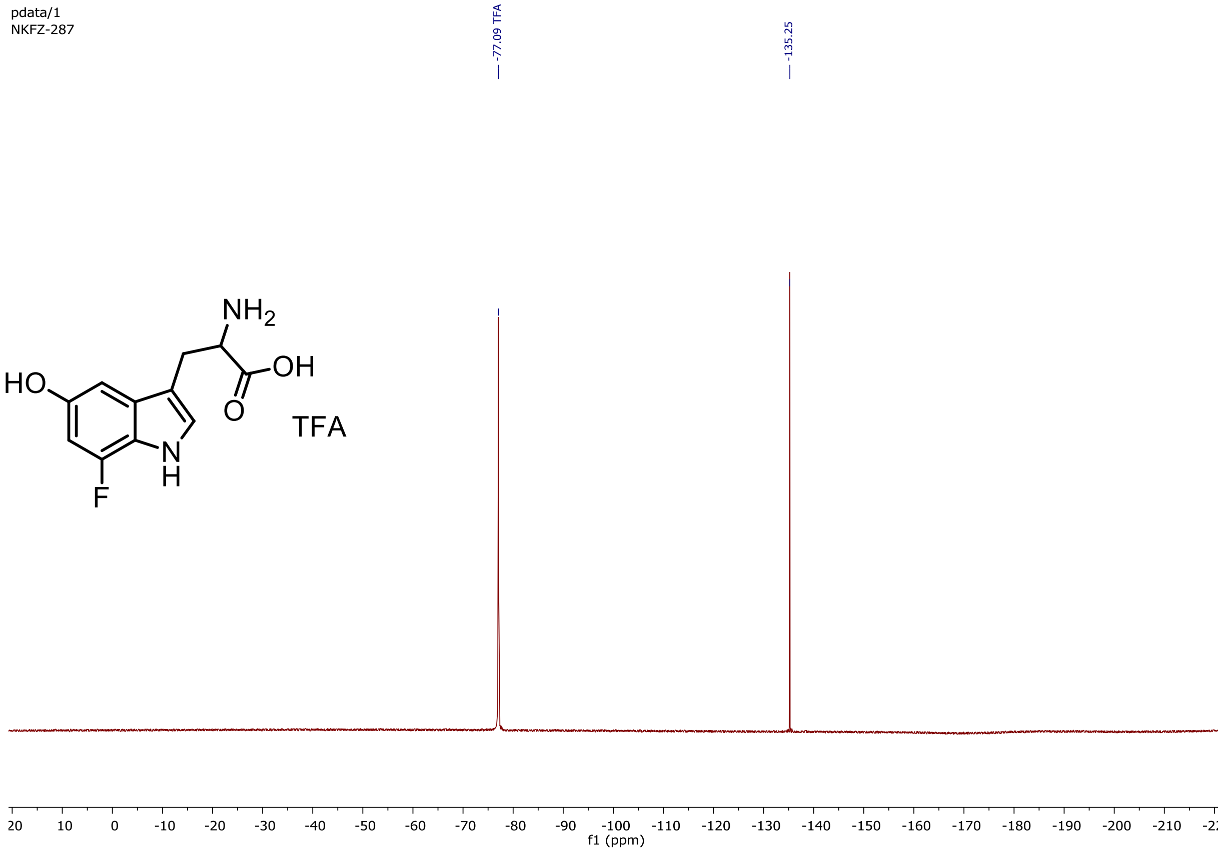

3.1.60 ^{1}H-NMR of 5-bromo-7-fluoroindole (S1)

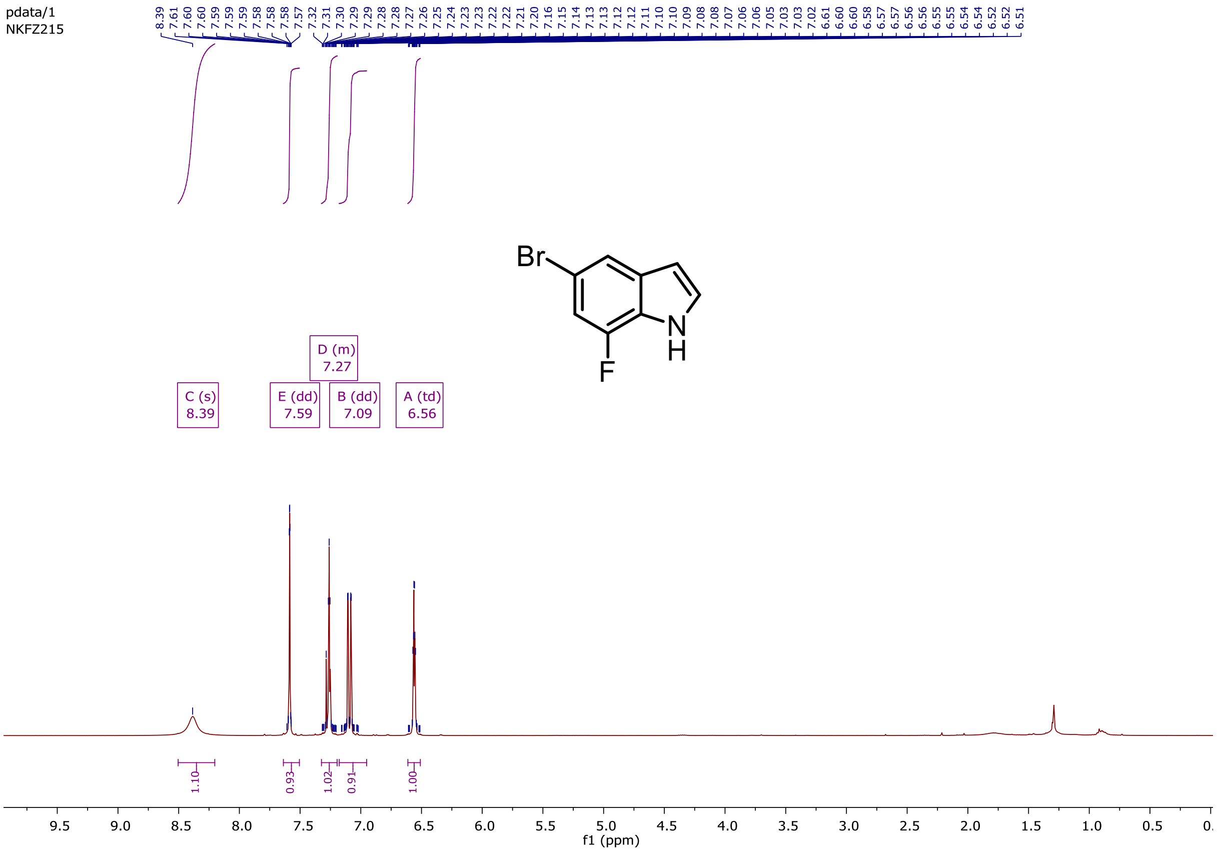

3.1.61 ^{13}C-NMR of 5-bromo-7-fluoroindole (S1)

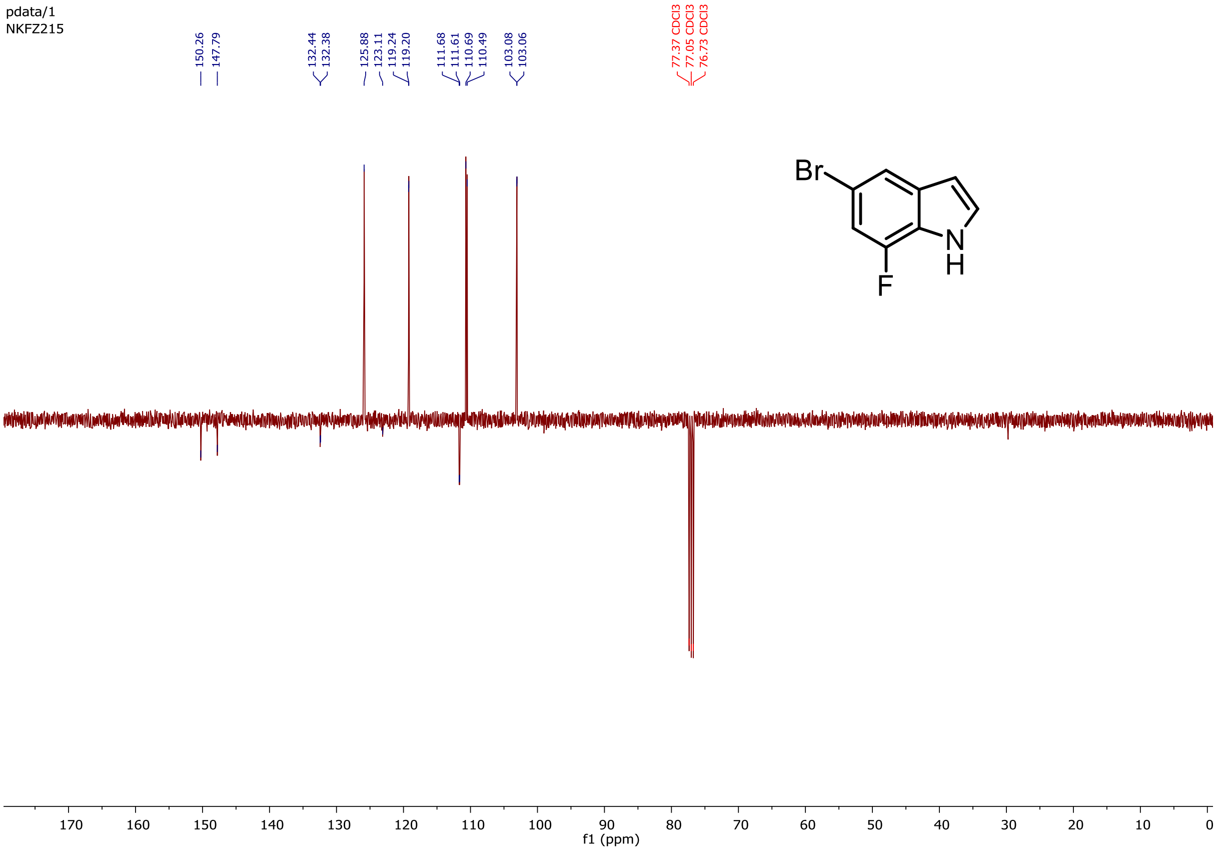

3.1.62 ^{19}F-NMR of 5-bromo-7-fluoroindole (S1)

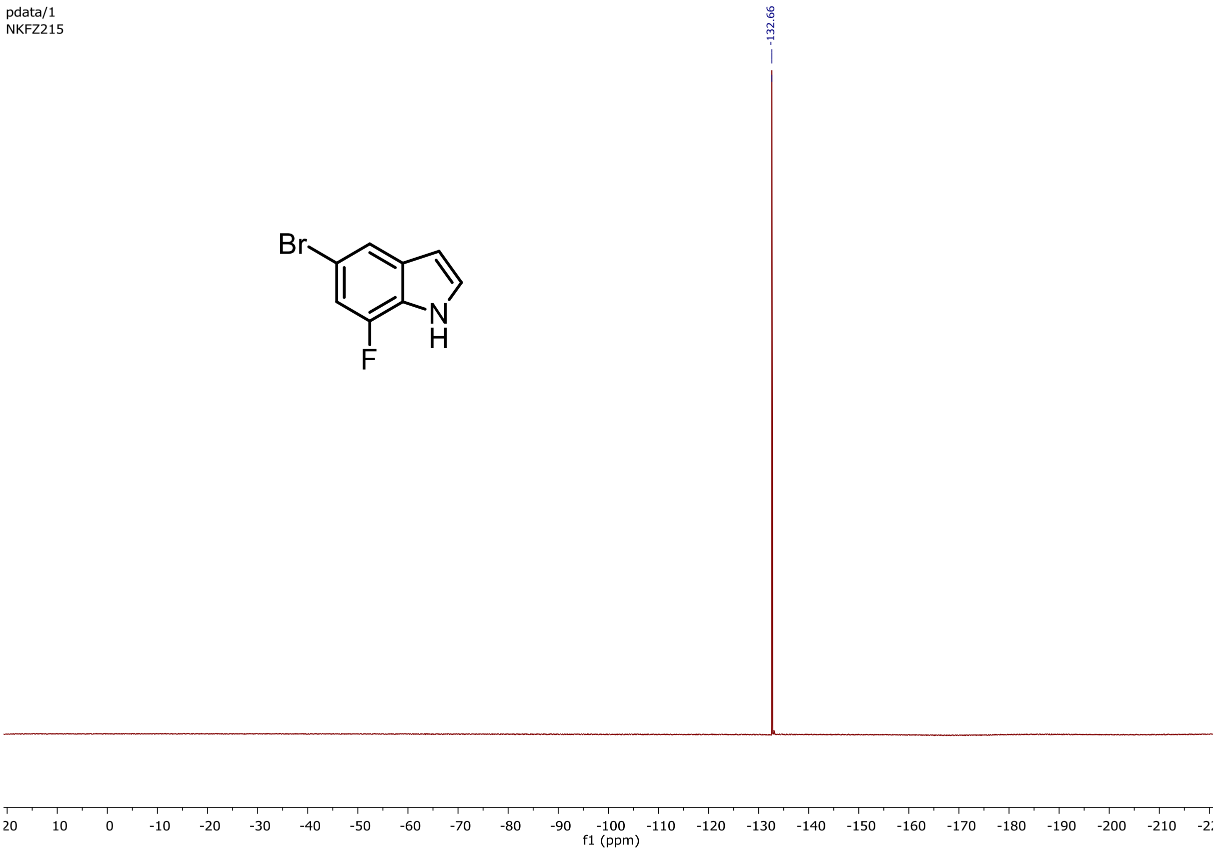

3.1.63 ^{1}H-NMR of *tert*-butyl (2-fluoro-4-hydroxyphenyl)carbamate (S2)

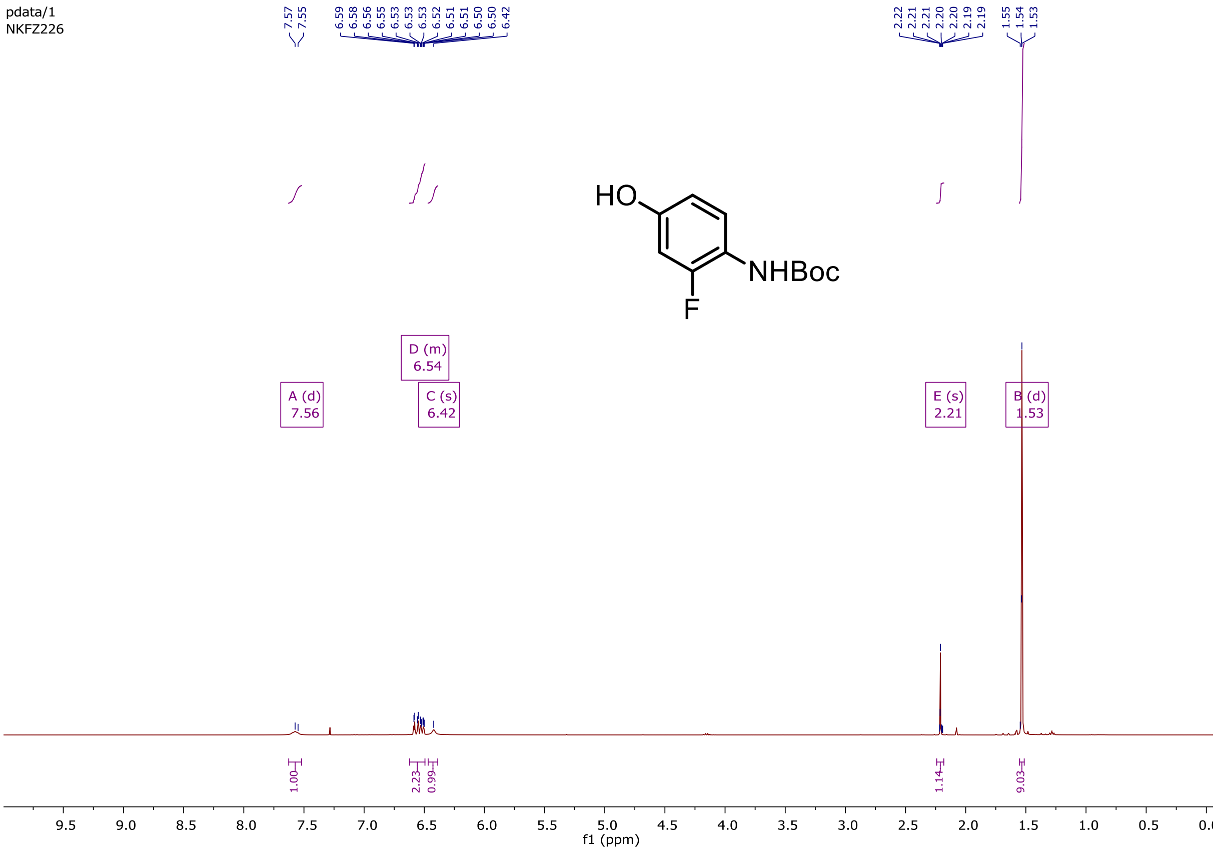

3.1.64 ^{13}C-NMR of *tert*-butyl (2-fluoro-4-hydroxyphenyl)carbamate (S2)

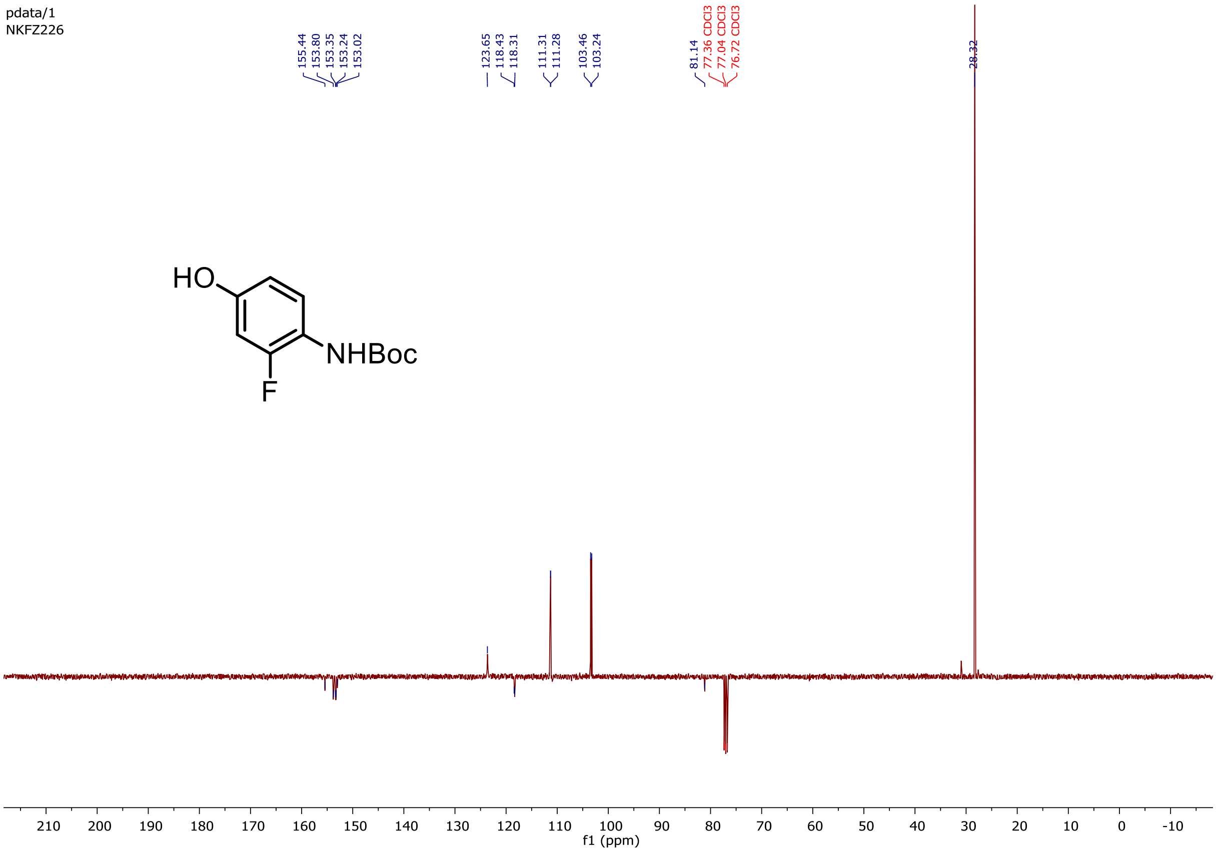

3.1.65 ^{19}F-NMR of *tert*-butyl (2-fluoro-4-hydroxyphenyl)carbamate (S2)

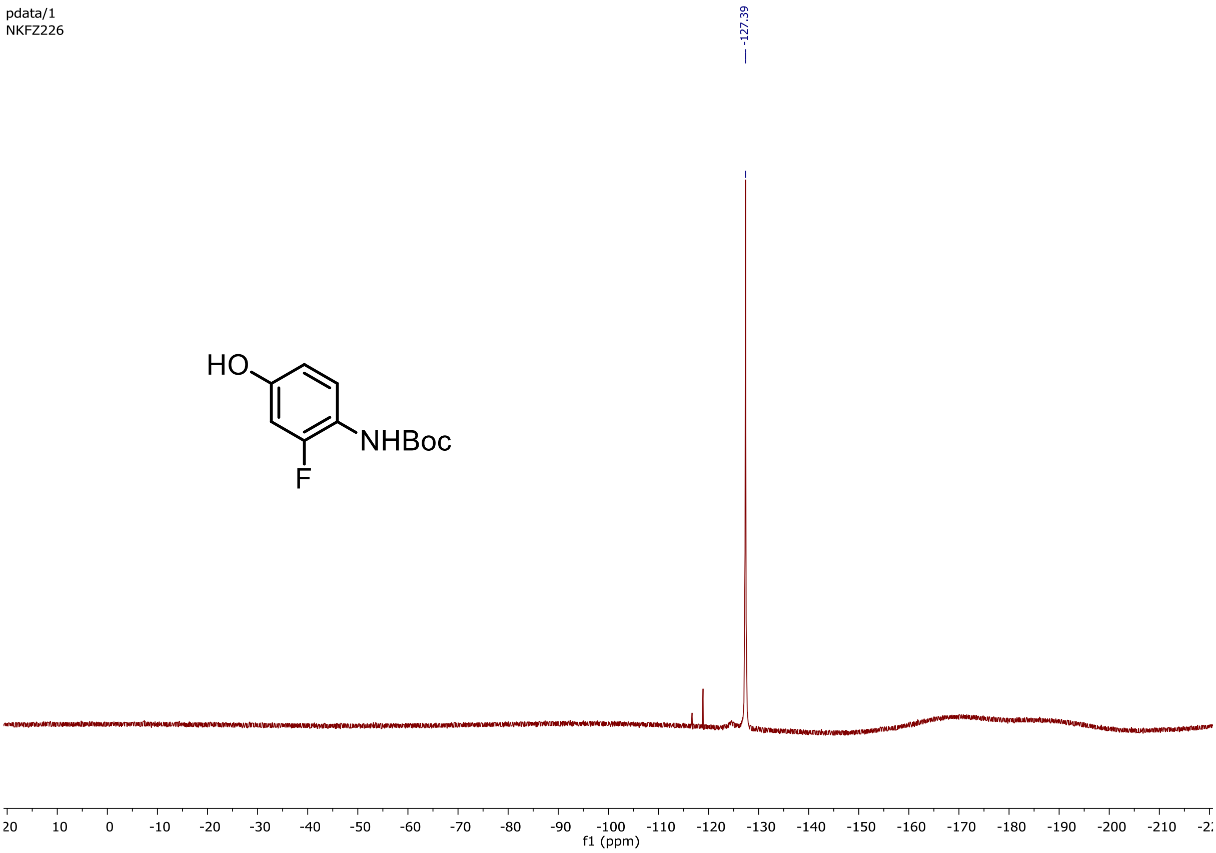

3.1.66 ^{1}H-NMR of *tert*-butyl (4-(*tert*-butoxy)-2-fluorophenyl)carbamate (S3)

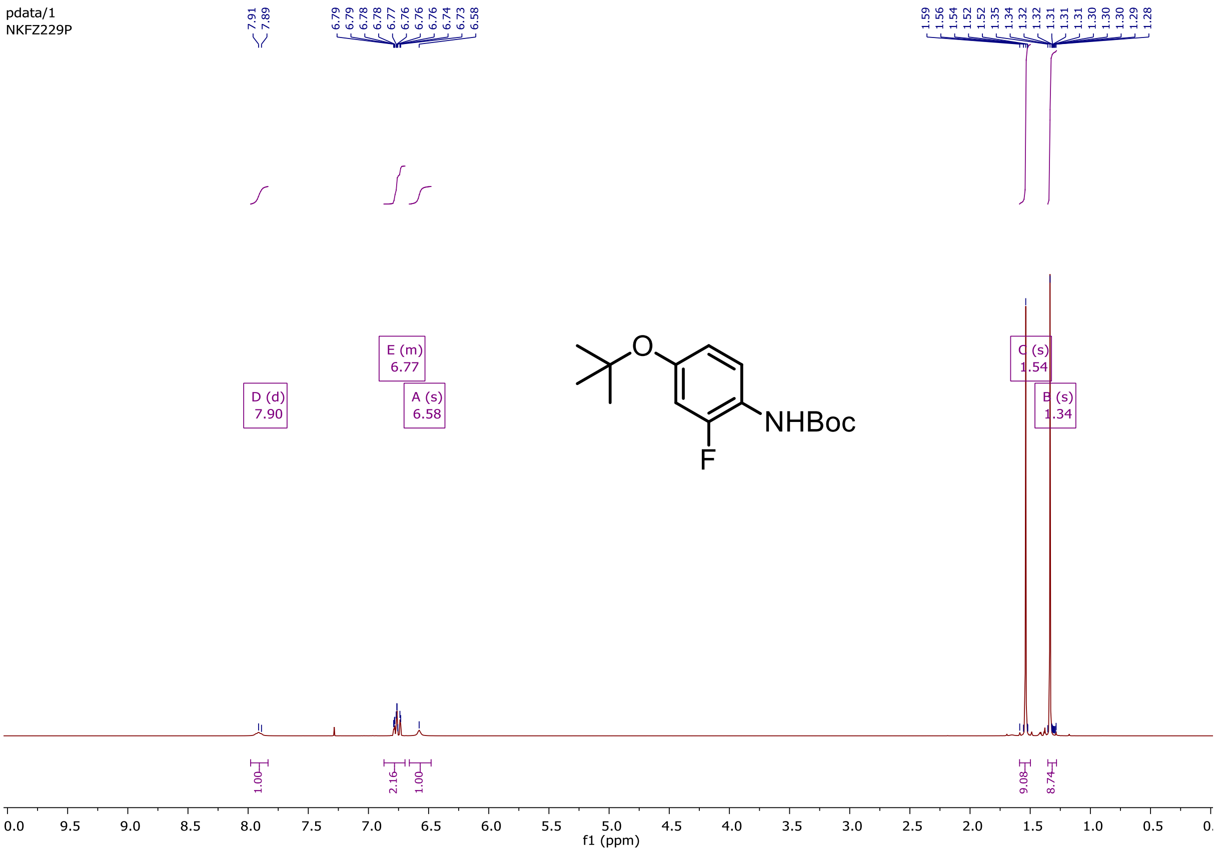

3.1.67 ^{13}C-NMR of *tert*-butyl (4-(*tert*-butoxy)-2-fluorophenyl)carbamate (S3)

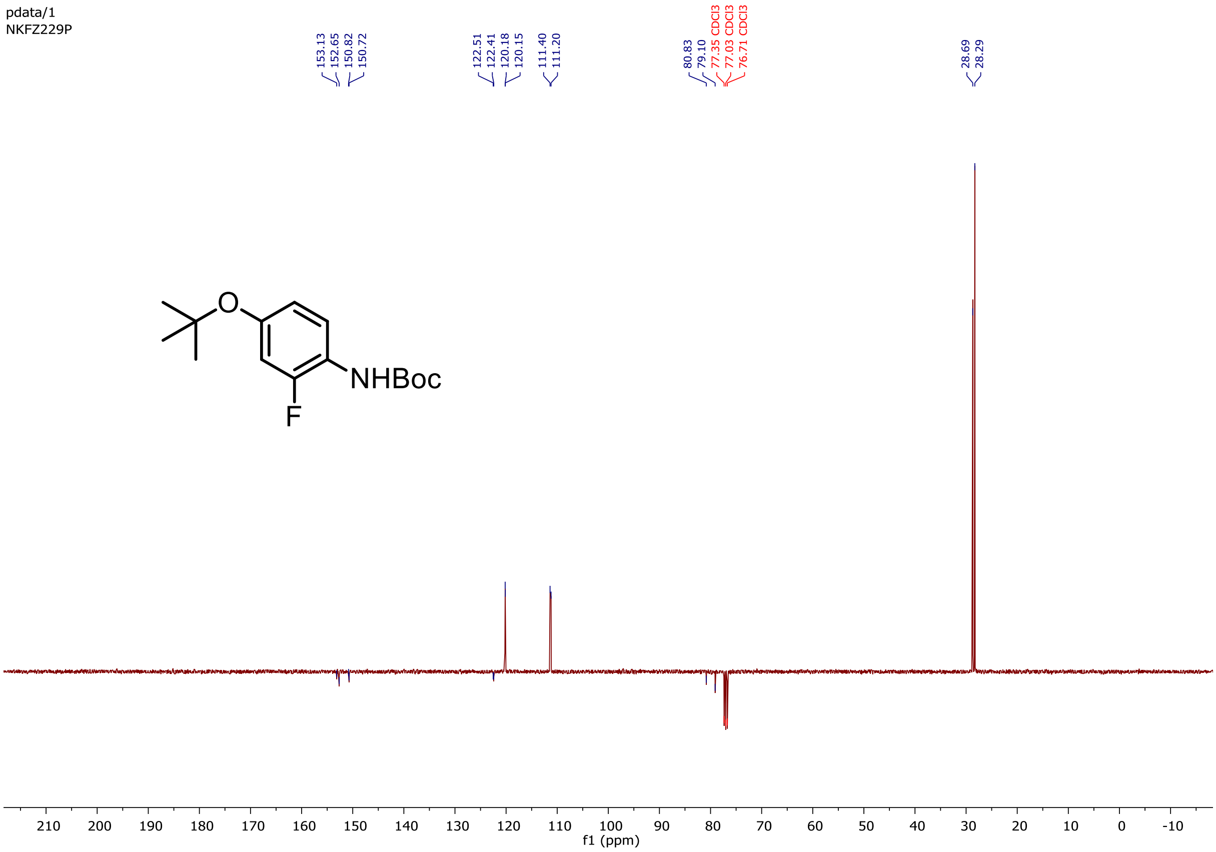

3.1.68 ^{19}F-NMR of *tert*-butyl (4-(*tert*-butoxy)-2-fluorophenyl)carbamate (S3)

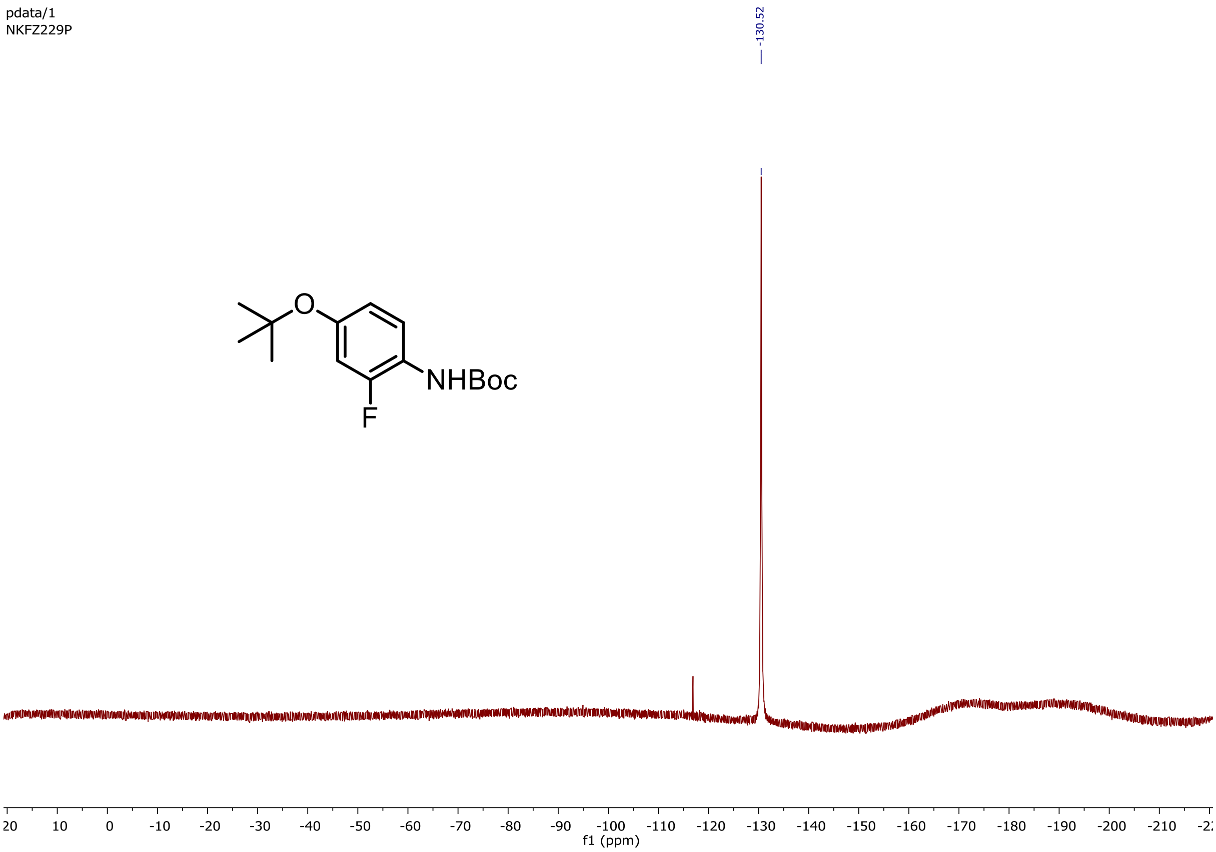

3.1.69 ^{1}H-NMR of (*S*)-1-benzyl 2-*tert*-butyl 5-oxopyrrolidine-1,2-dicarboxylate (S5)

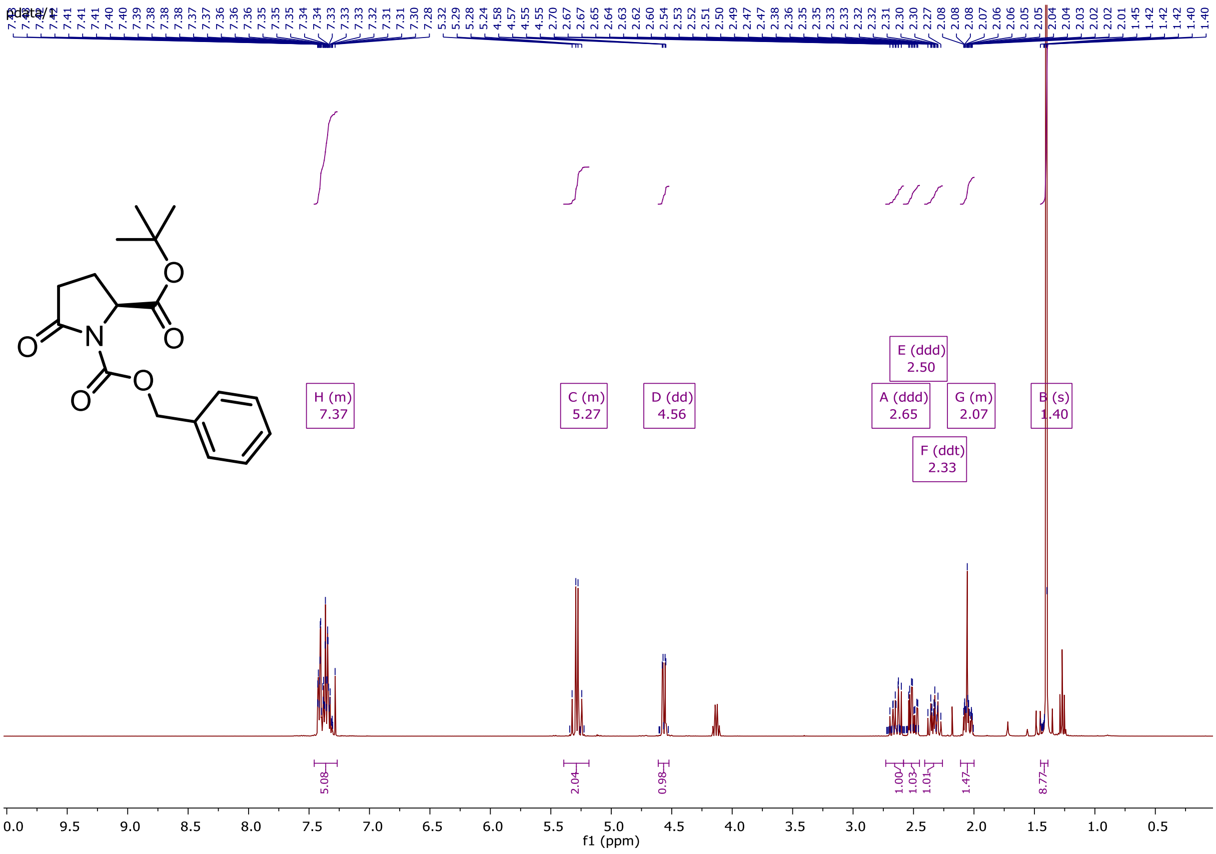

3.1.70 ^{13}C-NMR of (*S*)-1-benzyl 2-*tert*-butyl 5-oxopyrrolidine-1,2-dicarboxylate (S5)

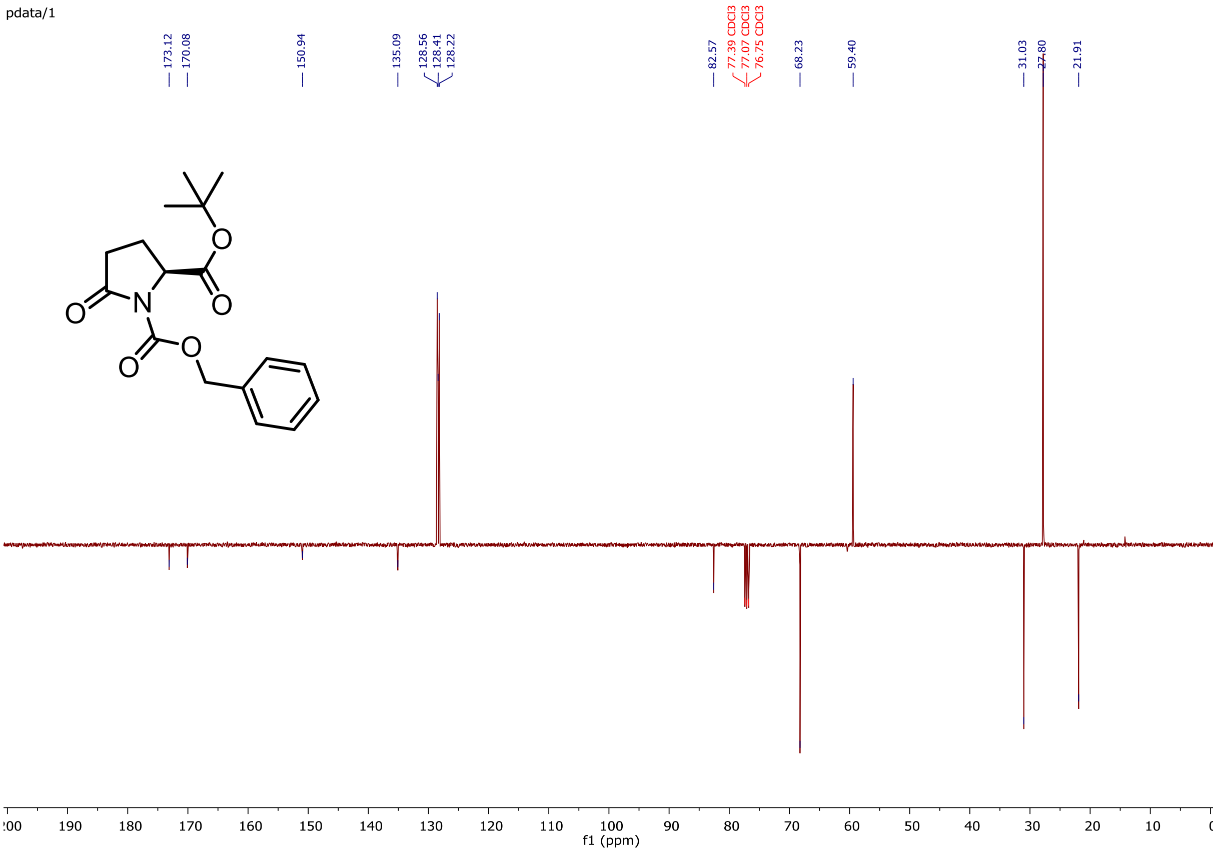

3.1.71 ^{1}H-NMR of (4-fluoro-2-methoxyphenyl)hydrazine hydrochloride (S6)

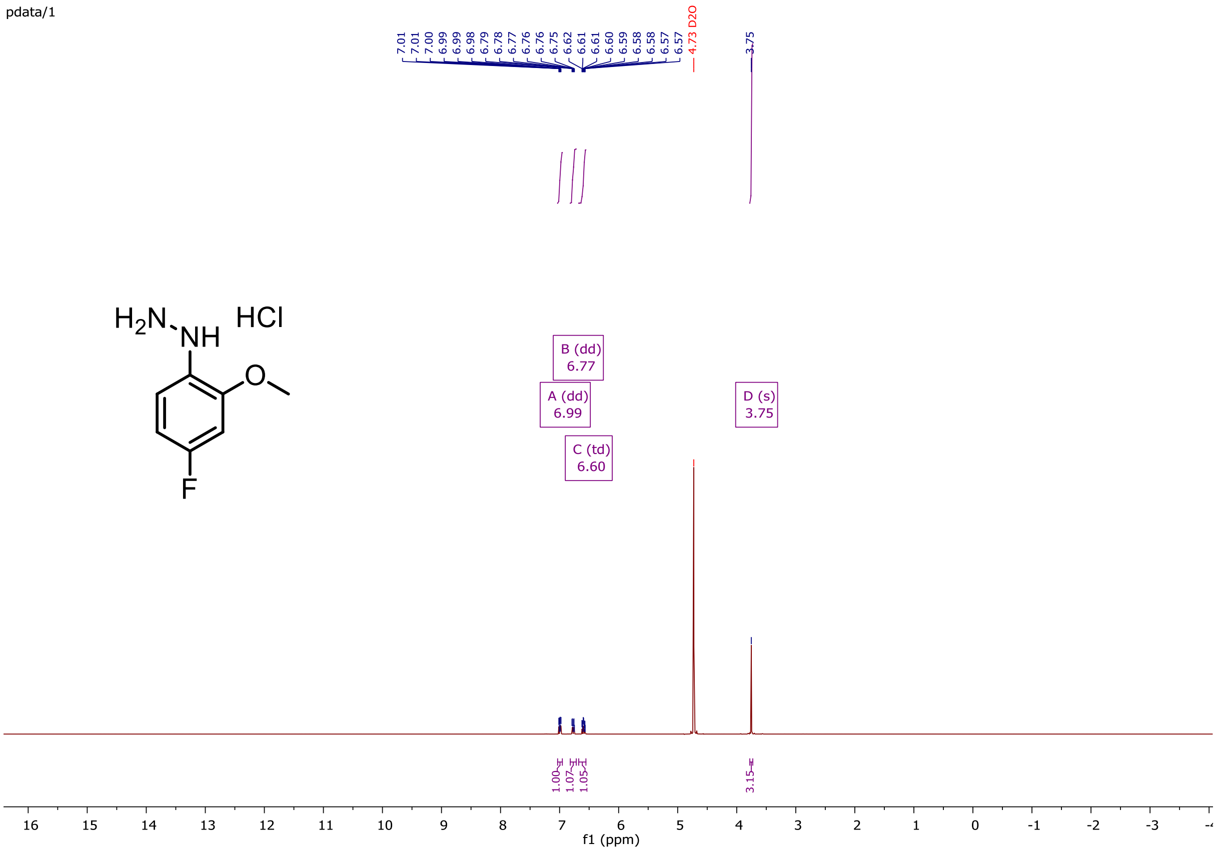

3.1.72 ^{13}C-NMR of (4-fluoro-2-methoxyphenyl)hydrazine hydrochloride (S6)

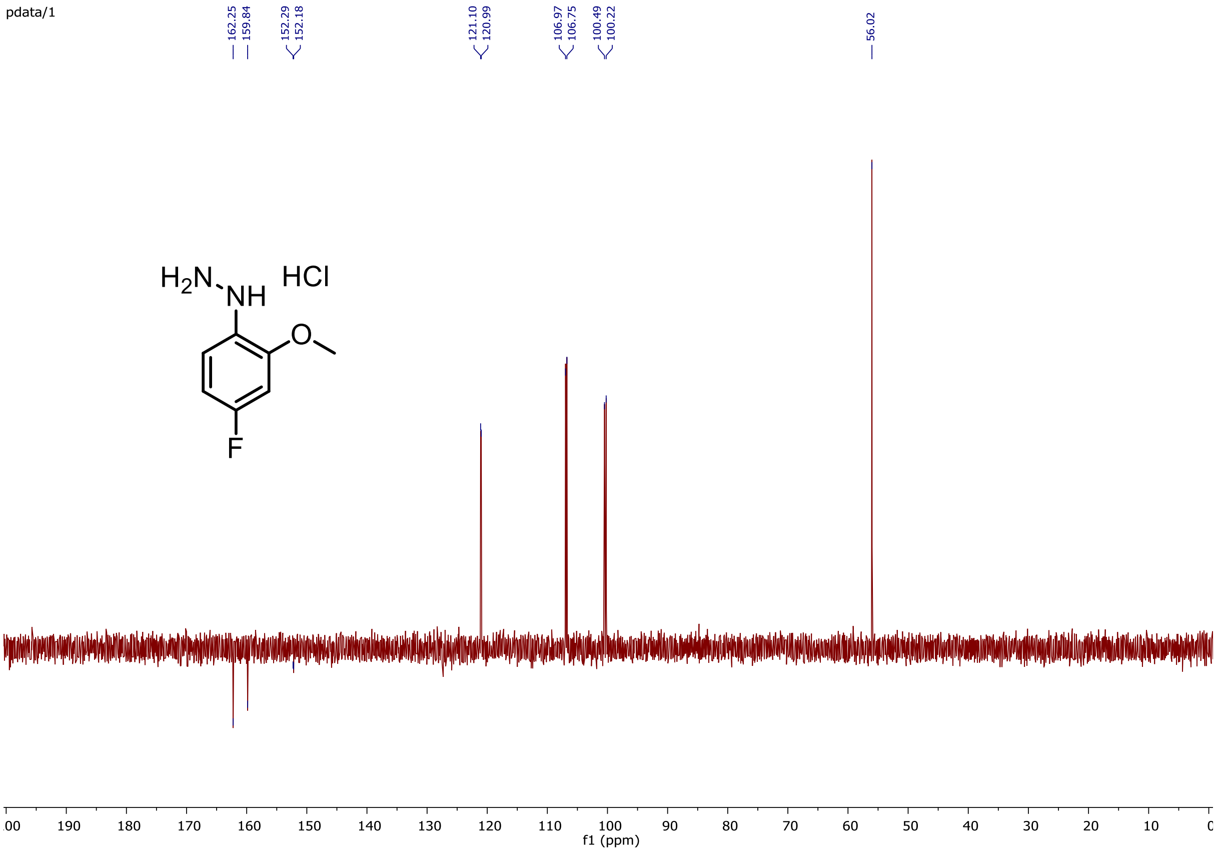

3.1.73 ^{19}F-NMR of (4-fluoro-2-methoxyphenyl)hydrazine hydrochloride (S6)

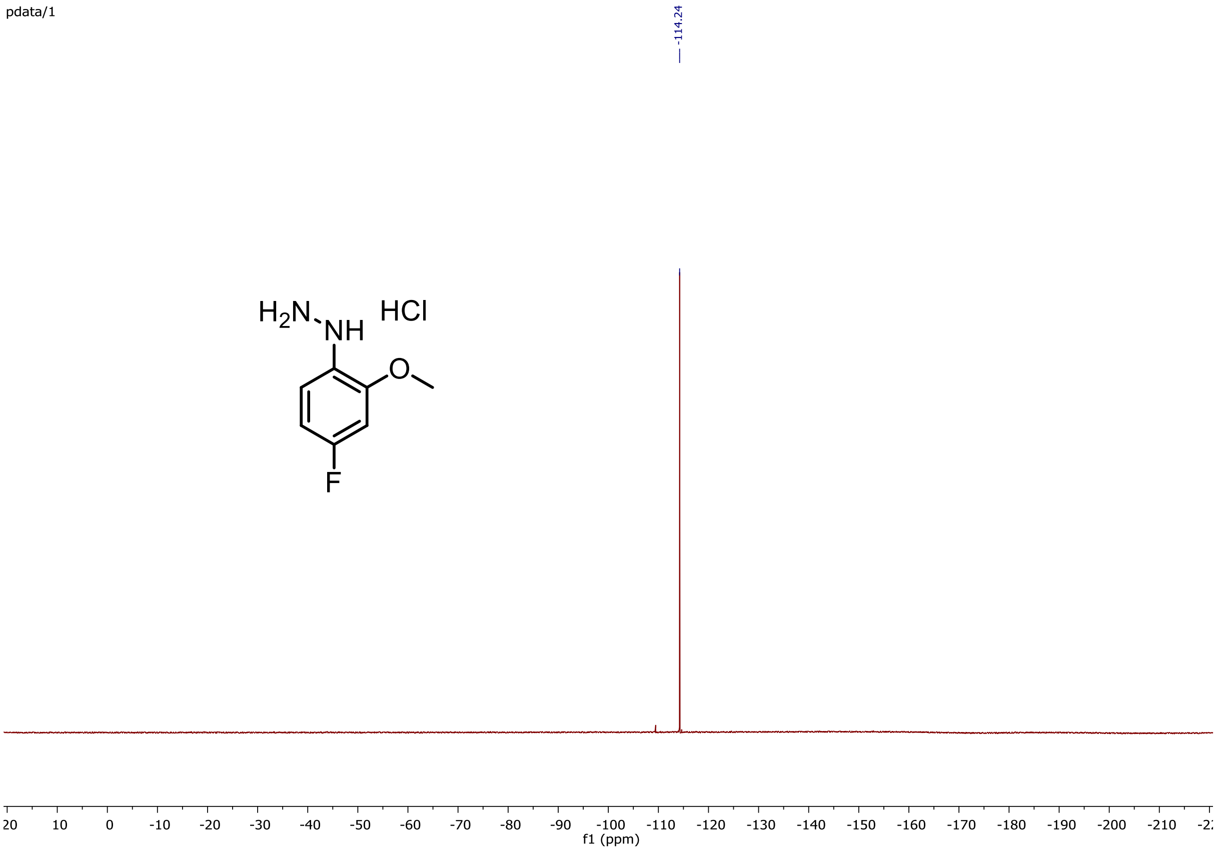

3.1.74 ^{1}H-NMR of 1-(7-fluoro-5-iodo-1*H*-indol-3-yl)-*N,N*-dimethylmethanamine (S9)

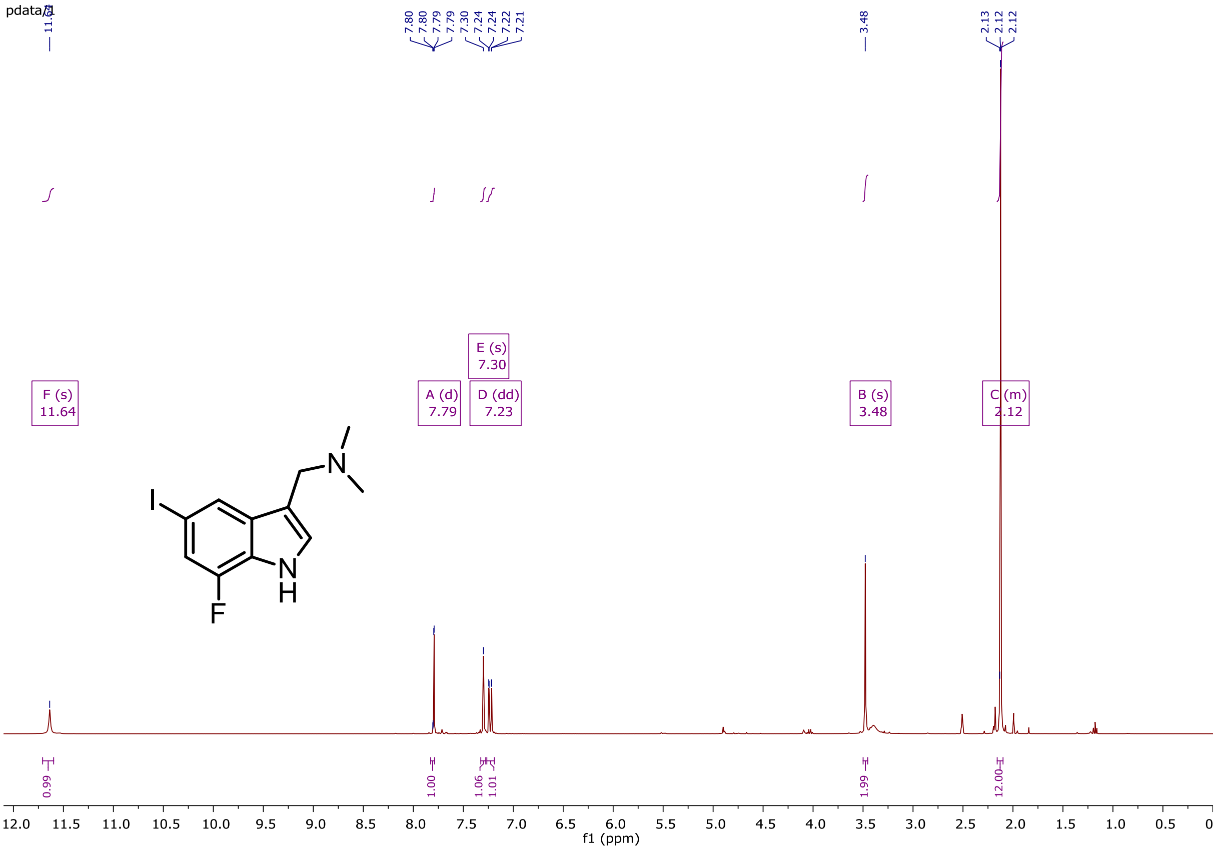

3.1.75 ^{13}C-NMR of 1-(7-fluoro-5-iodo-1*H*-indol-3-yl)-*N,N*-dimethylmethanamine (S9)

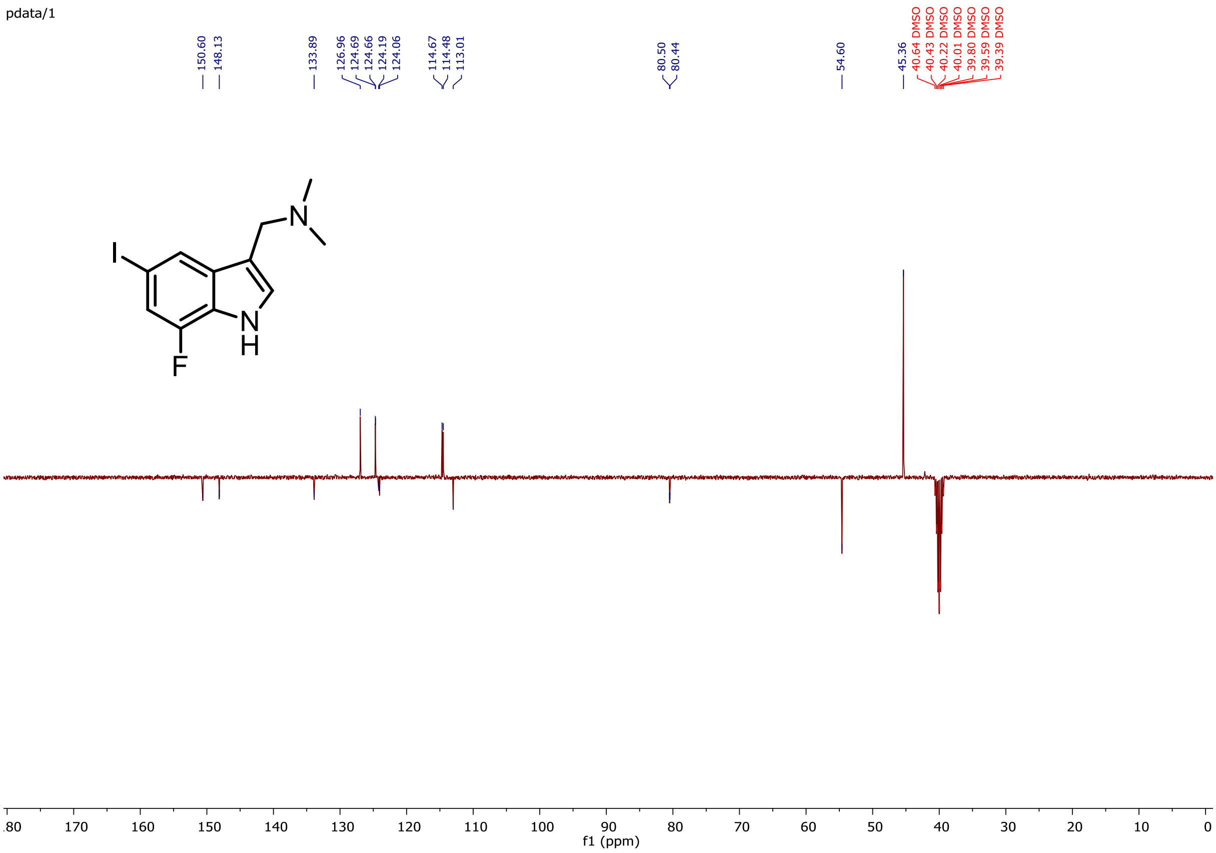

3.1.76 ^{19}F-NMR of 1-(7-fluoro-5-iodo-1*H*-indol-3-yl)-*N,N*-dimethylmethanamine (S9)

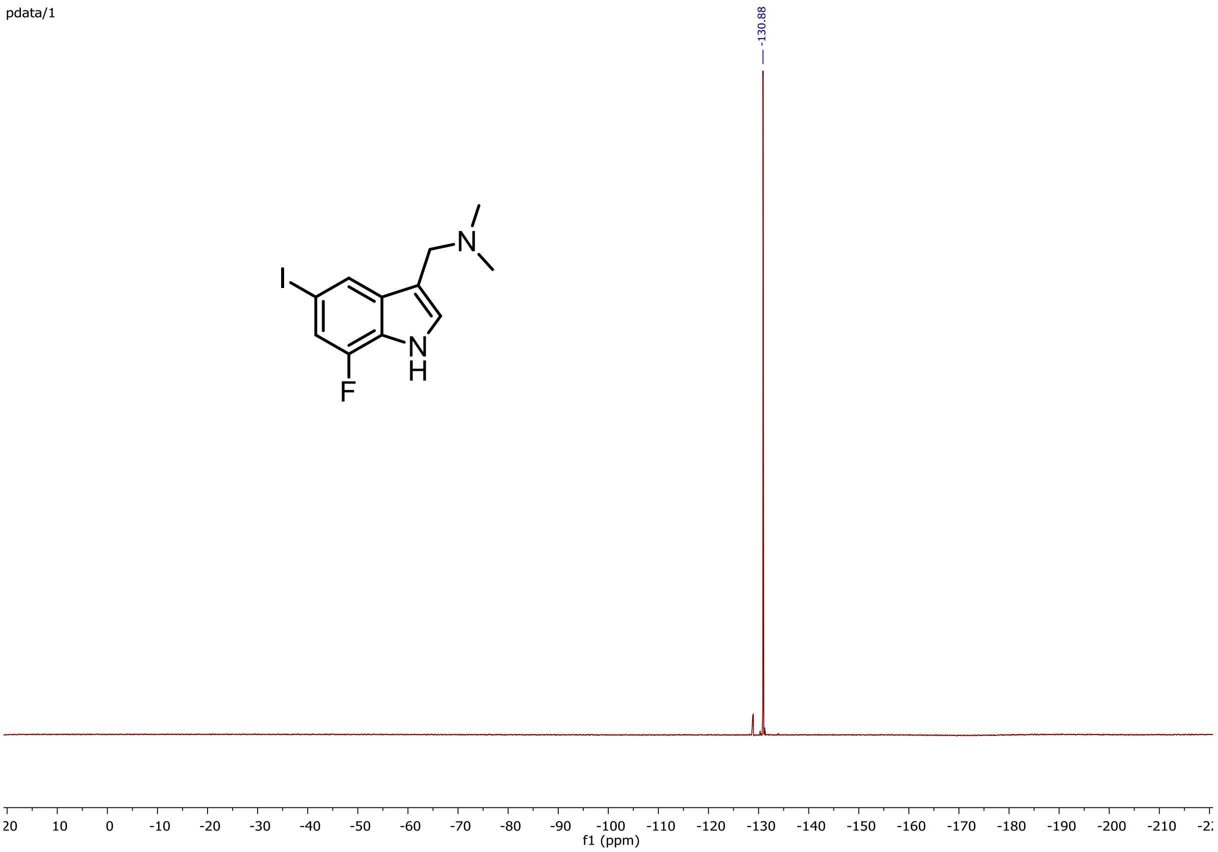

3.1.77 ^{1}H-NMR of diethyl 2-((7-fluoro-5-iodo-1*H*-indol-3-yl)methyl)-2-formamidomalonate (S10)

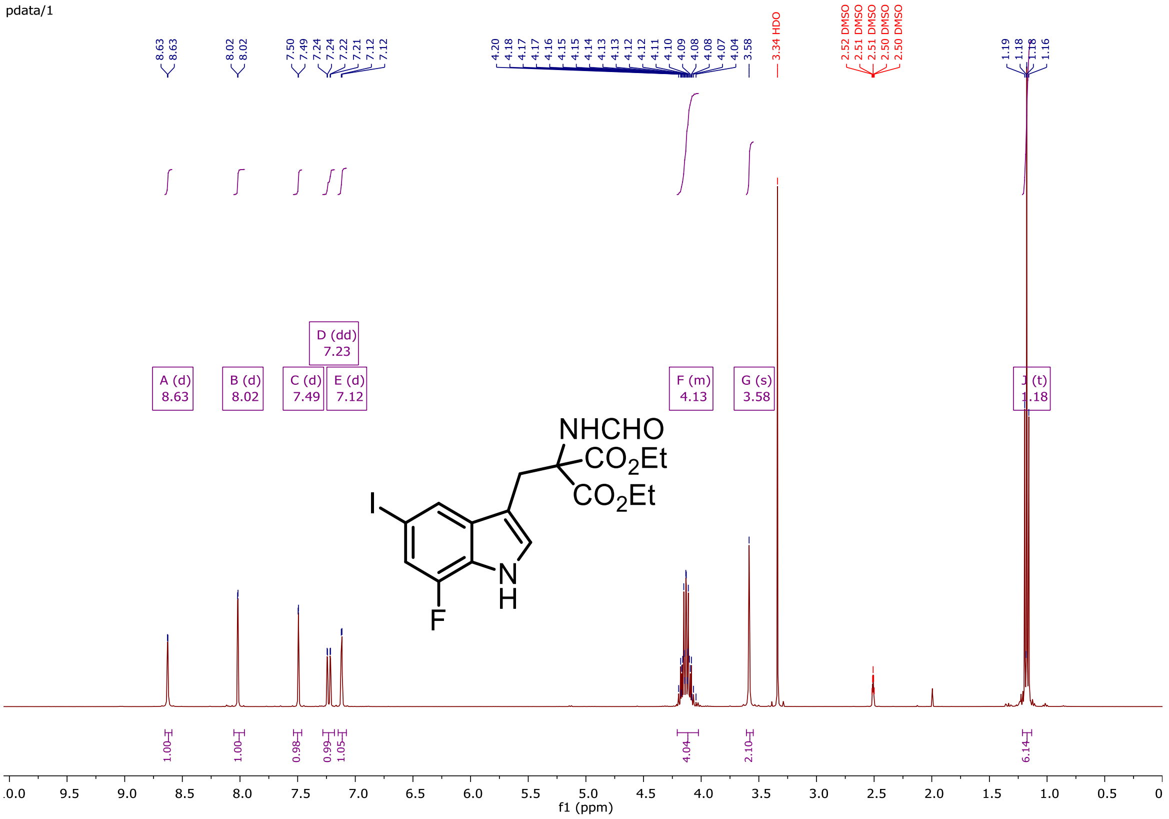

3.1.78 ^{13}C-NMR of diethyl 2-((7-fluoro-5-iodo-1*H*-indol-3-yl)methyl)-2-formamidomalonate (S10)

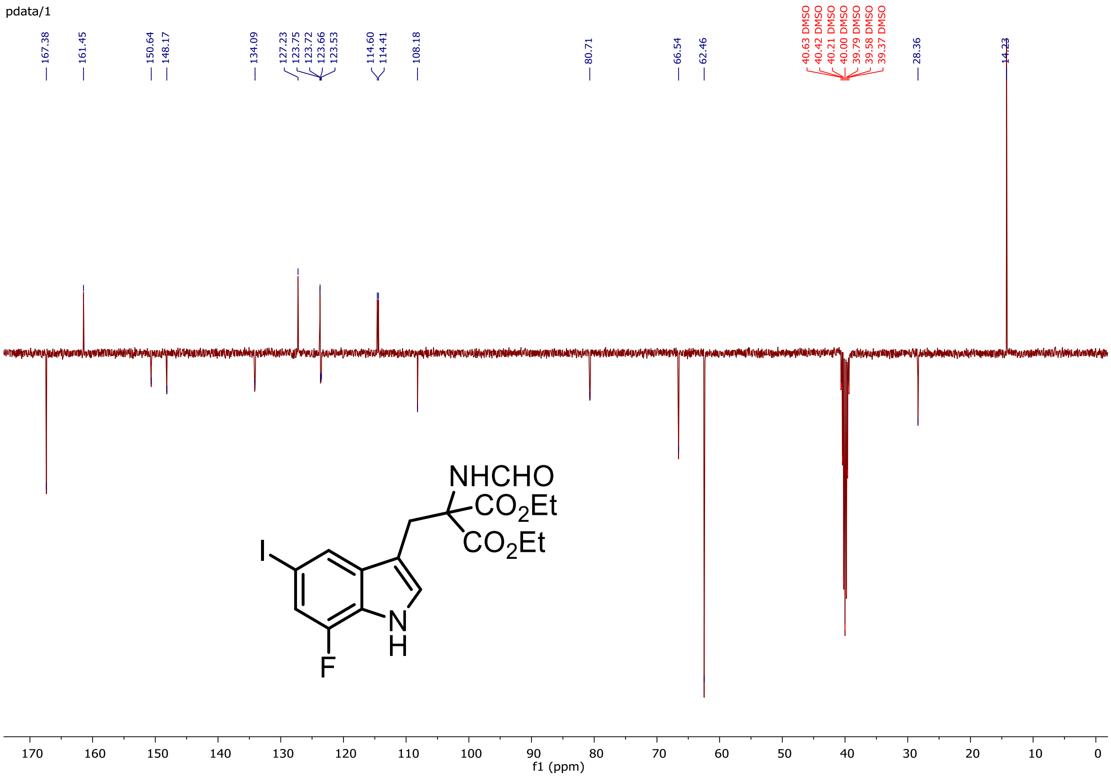

3.1.79 ^{19}F-NMR of diethyl 2-((7-fluoro-5-iodo-1*H*-indol-3-yl)methyl)-2-formamidomalonate (S10)

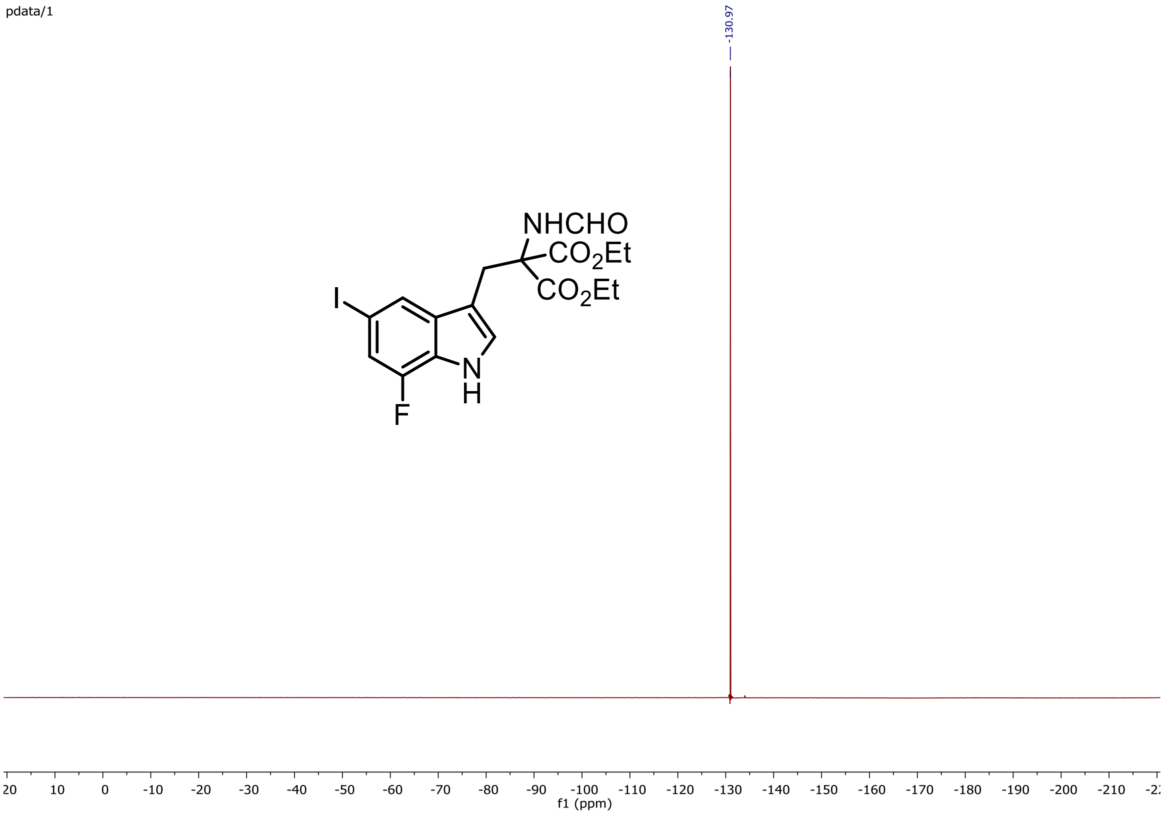

3.1.80 ^{1}H-NMR of 2-amino-3-(7-fluoro-5-iodo-1*H*-indol-3-yl)propanoic acid (S11)

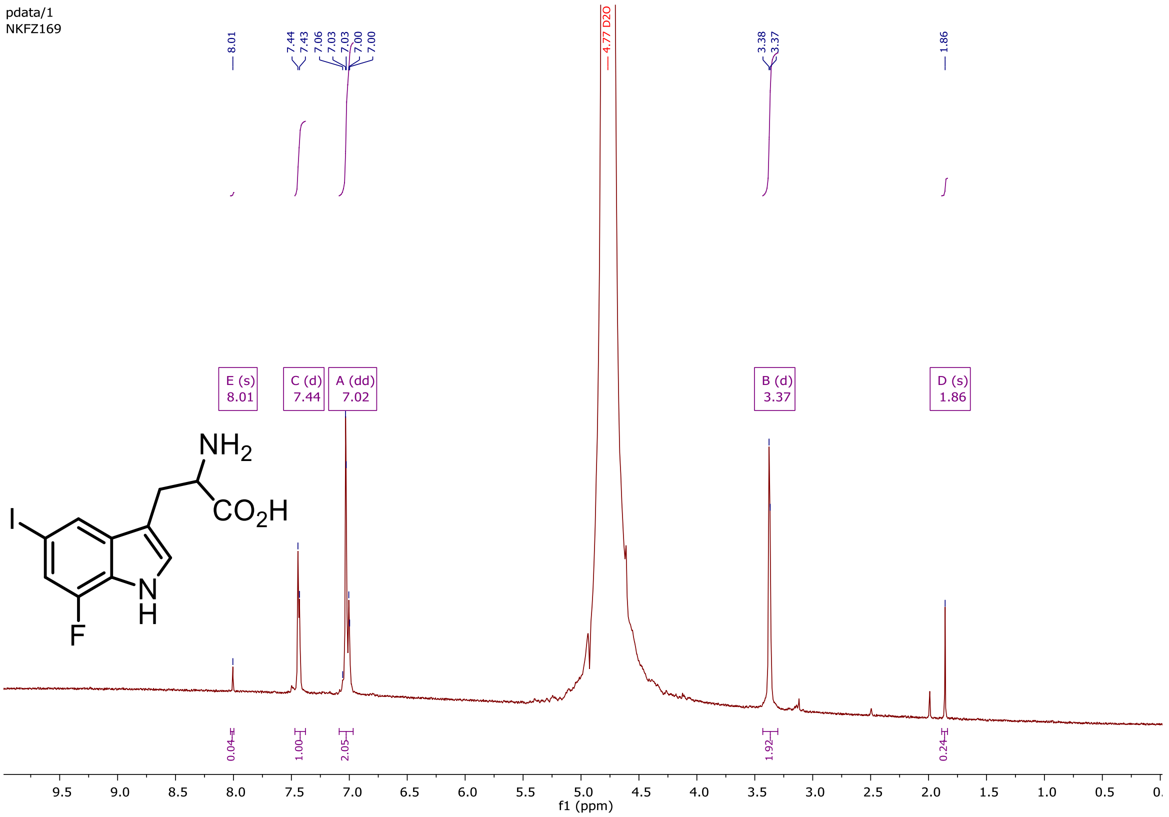

3.1.81 ^{13}C-NMR of 2-amino-3-(7-fluoro-5-iodo-1*H*-indol-3-yl)propanoic acid (S11)

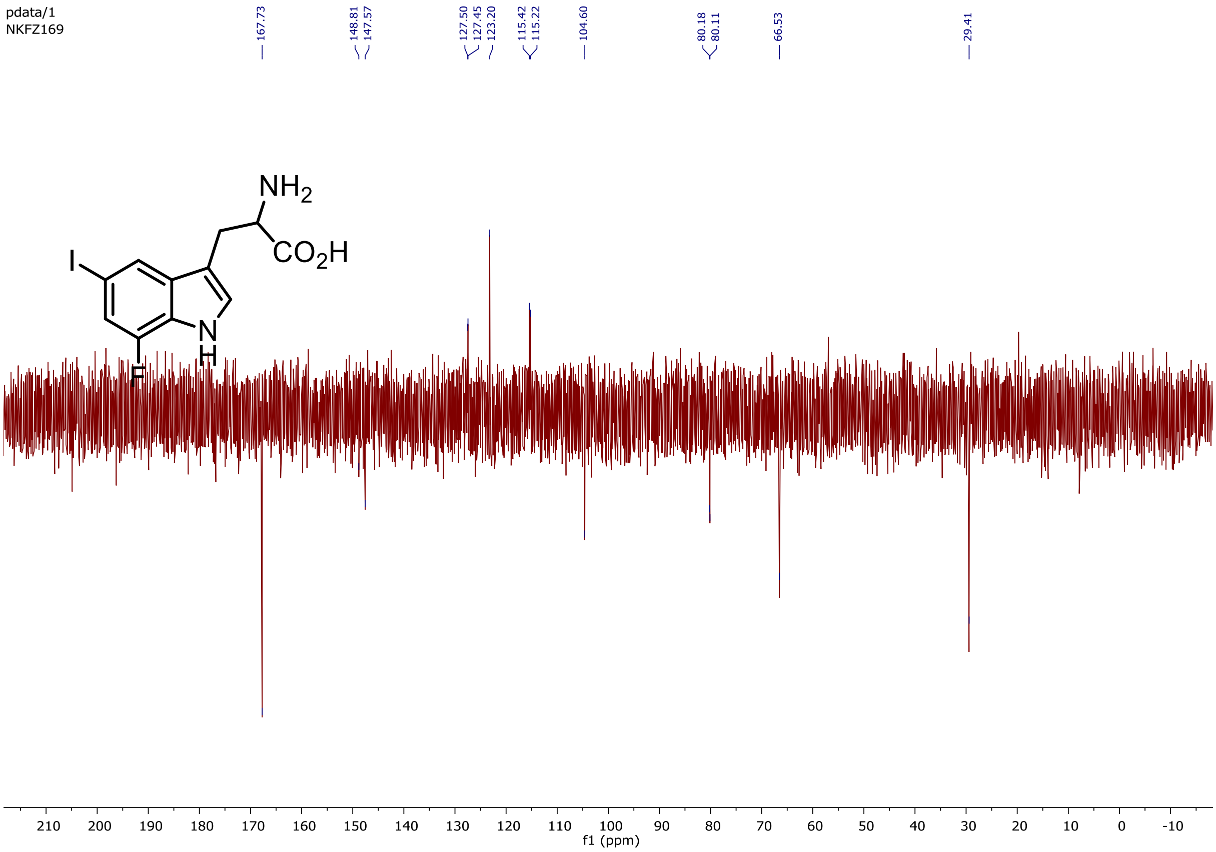

3.1.82 ^{19}F-NMR of 2-amino-3-(7-fluoro-5-iodo-1*H*-indol-3-yl)propanoic acid (S11)

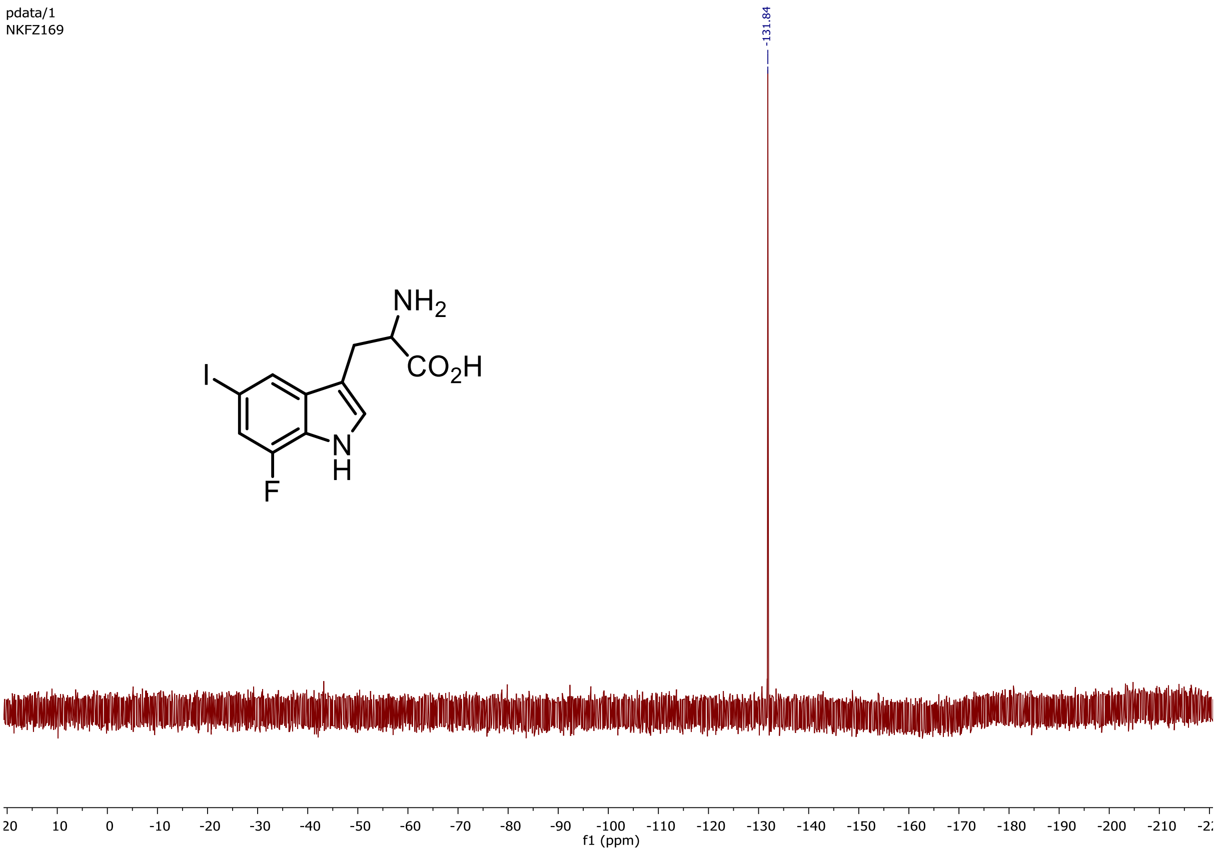

3.1.83 ^{1}H-NMR of 1-(7-fluoro-1*H*-indol-3-yl)-*N,N*-dimethylmethanamine (S13)

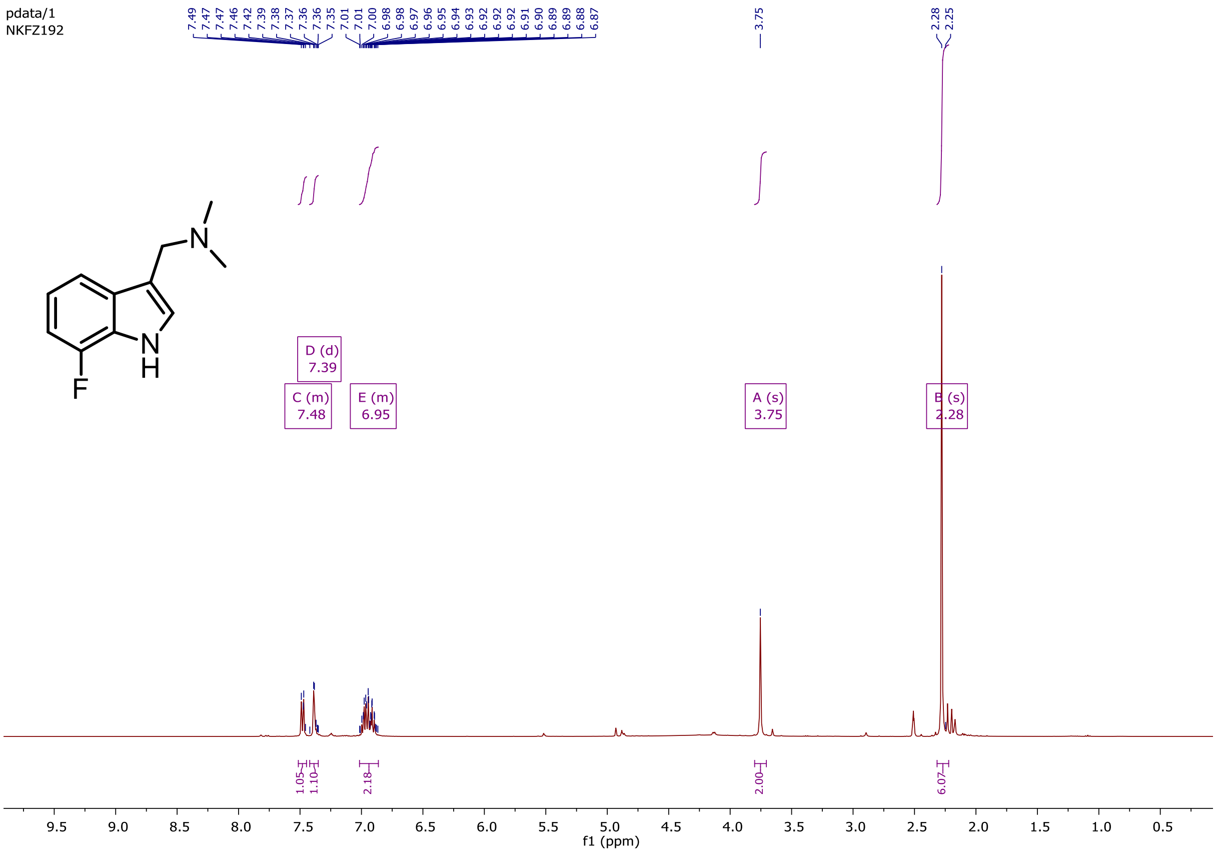

3.1.84 ^{13}C-NMR of 1-(7-fluoro-1*H*-indol-3-yl)-*N,N*-dimethylmethanamine (S13)

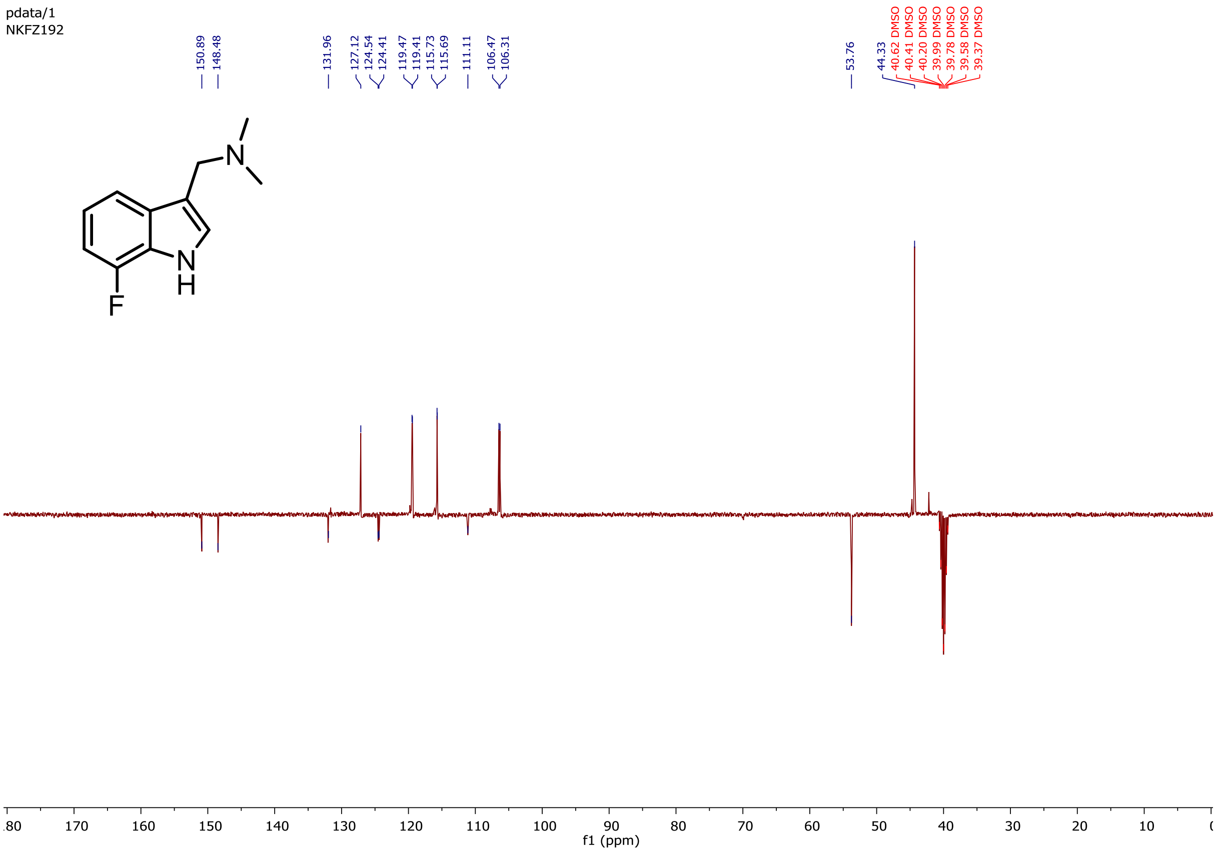

3.1.85 ^{19}F-NMR of 1-(7-fluoro-1*H*-indol-3-yl)-*N,N*-dimethylmethanamine (S13)

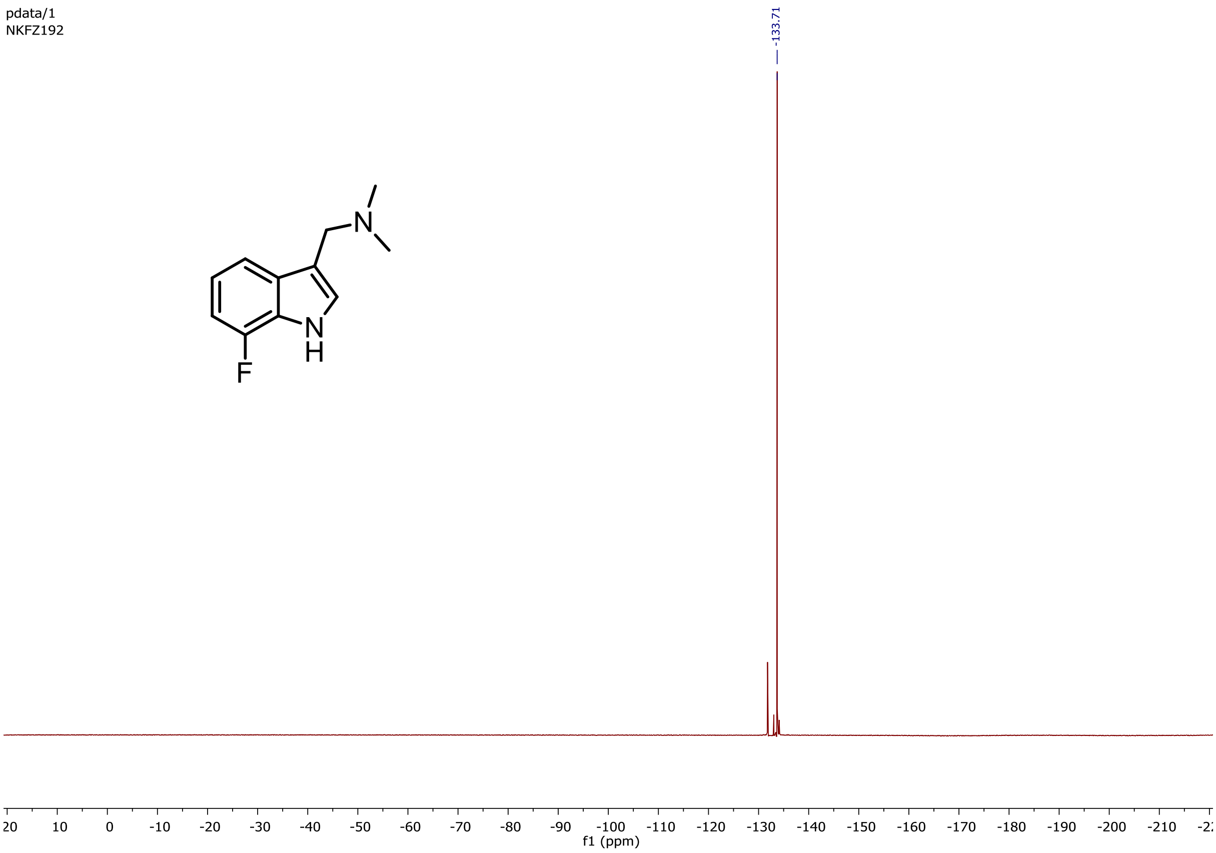

3.1.86 ^{1}H-NMR of diethyl 2-((7-fluoro-1*H*-indol-3-yl)methyl)-2-formamidomalonate (S14)

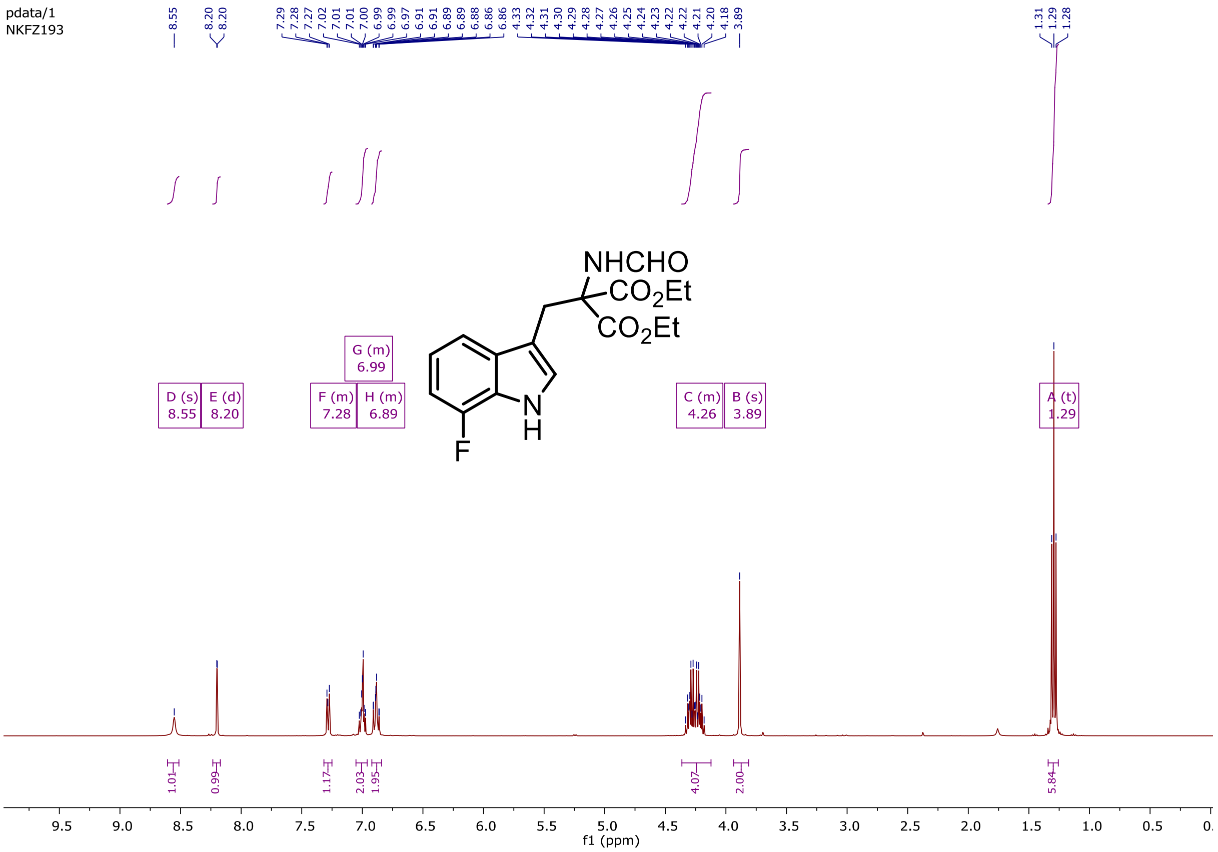

3.1.87 ^{13}C-NMR of diethyl 2-((7-fluoro-1*H*-indol-3-yl)methyl)-2-formamidomalonate (S14)

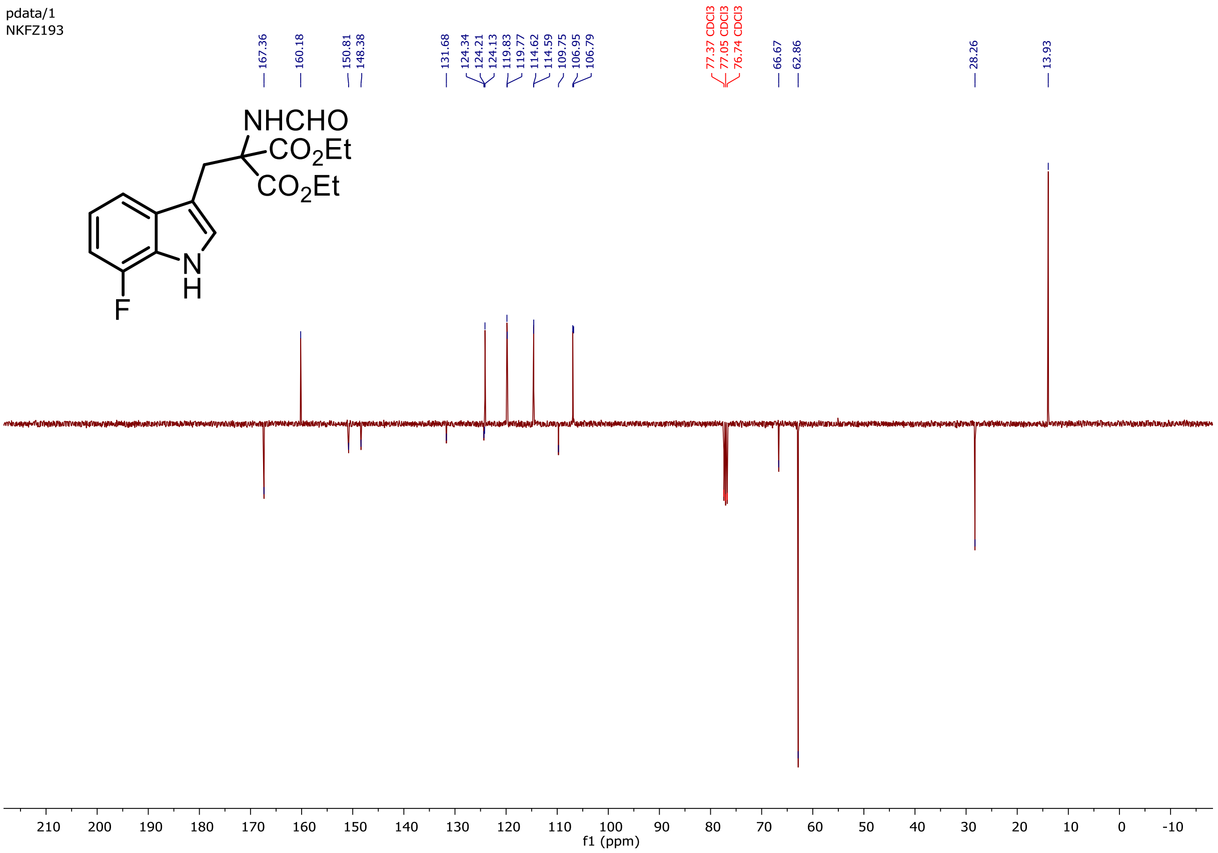

3.1.88 ^{19}F-NMR of diethyl 2-[(7-fluoro-1*H*-indol-3-yl)methyl]-2-formamidomalonate (S14)

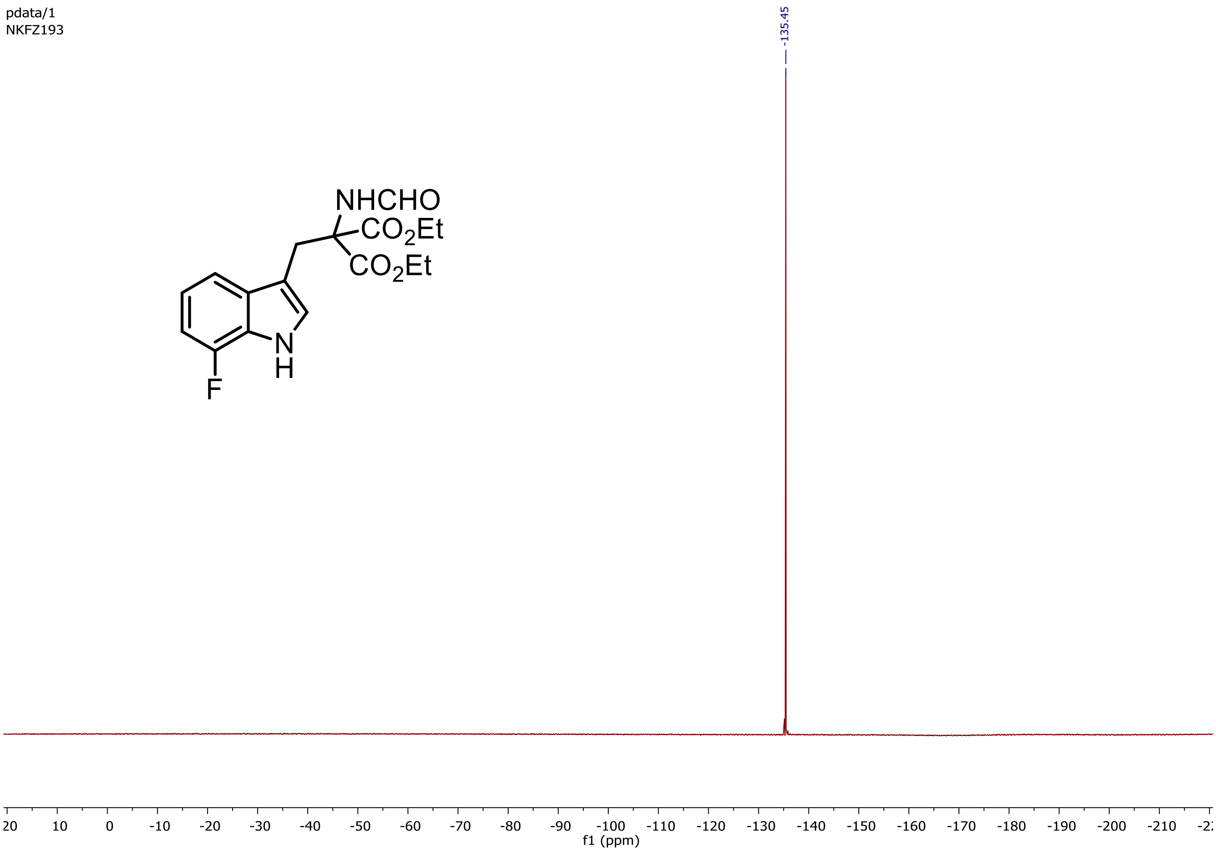

3.1.89 ^{1}H-NMR of 7-fluorotryptophan (S15)

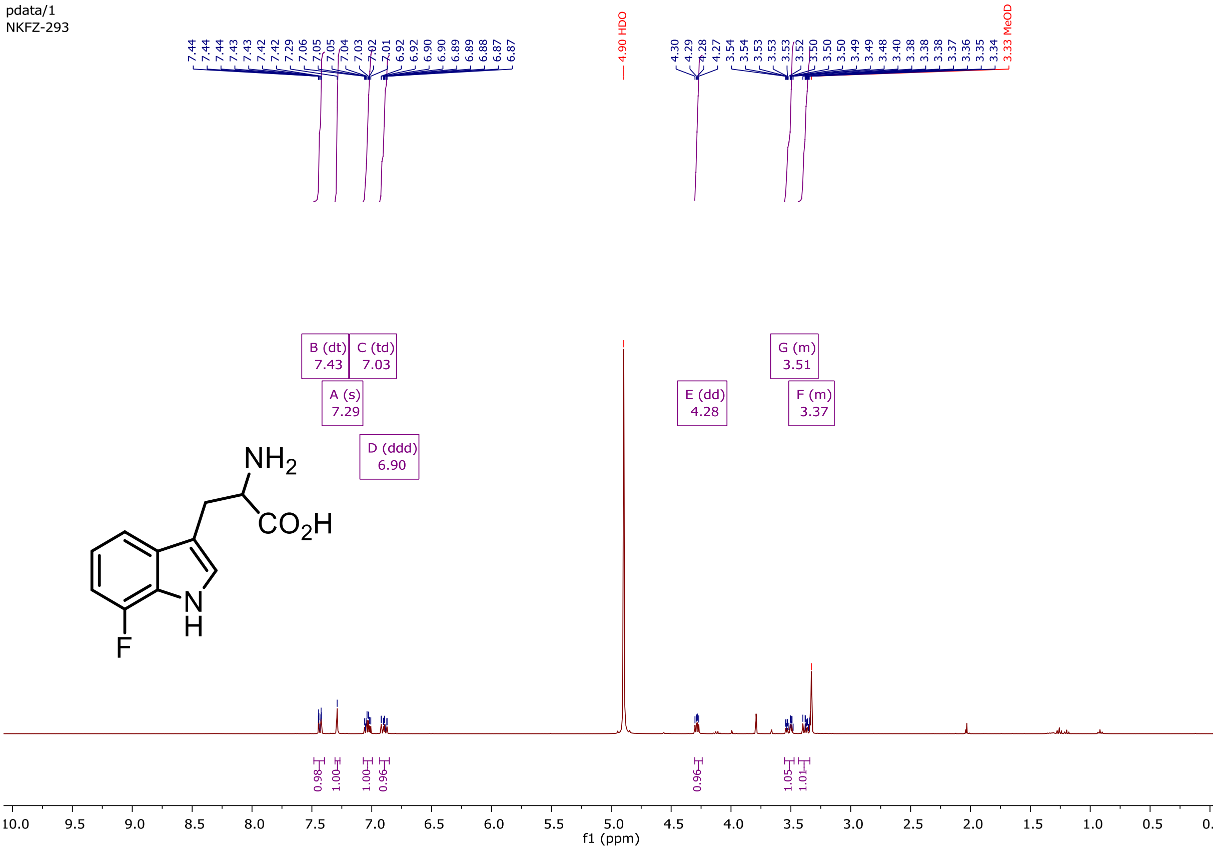

3.1.90 ^{13}C-NMR of 7-fluorotryptophan (S15)

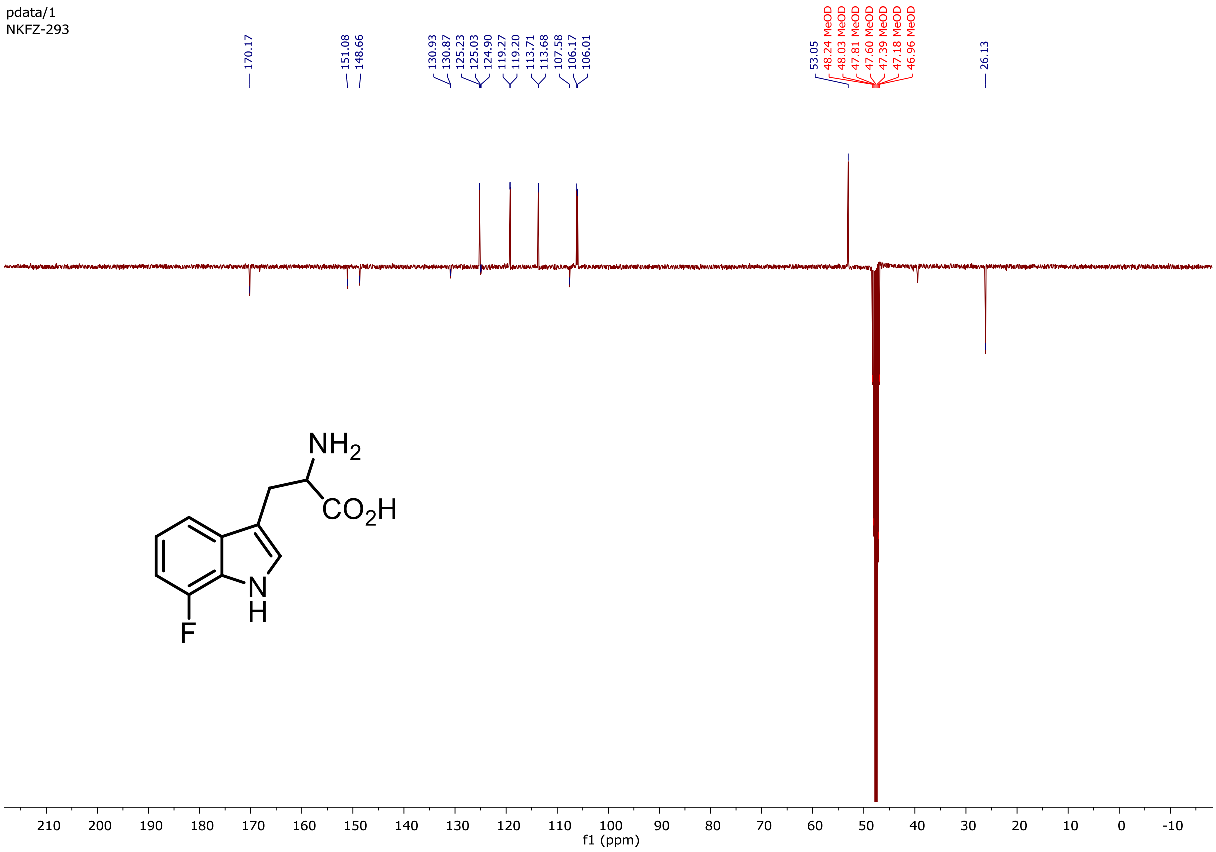

3.1.91 ^{19}F-NMR of 7-fluorotryptophan (S15)

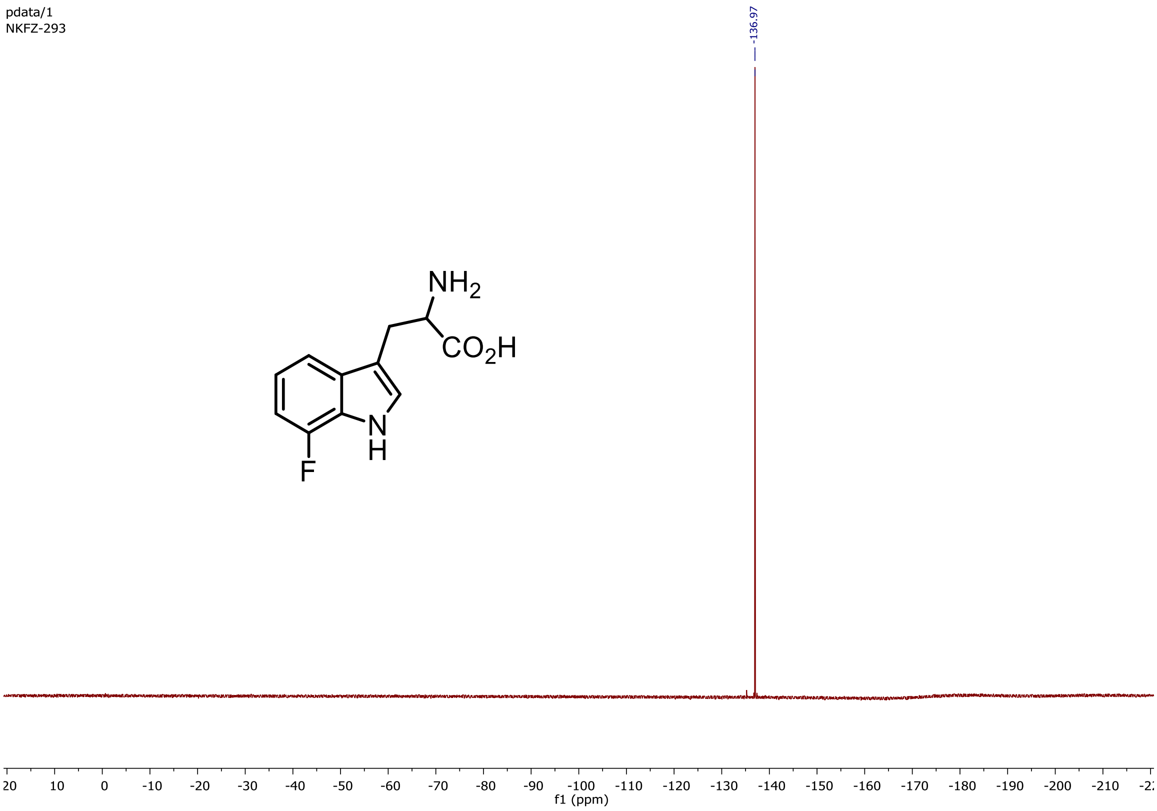

3.1.92 ^{1}H-NMR of 2-[(*tert*-butoxycarbonyl)amino]-3-(7-fluoro-1*H*-indol-3-yl)propanoic acid (S16)

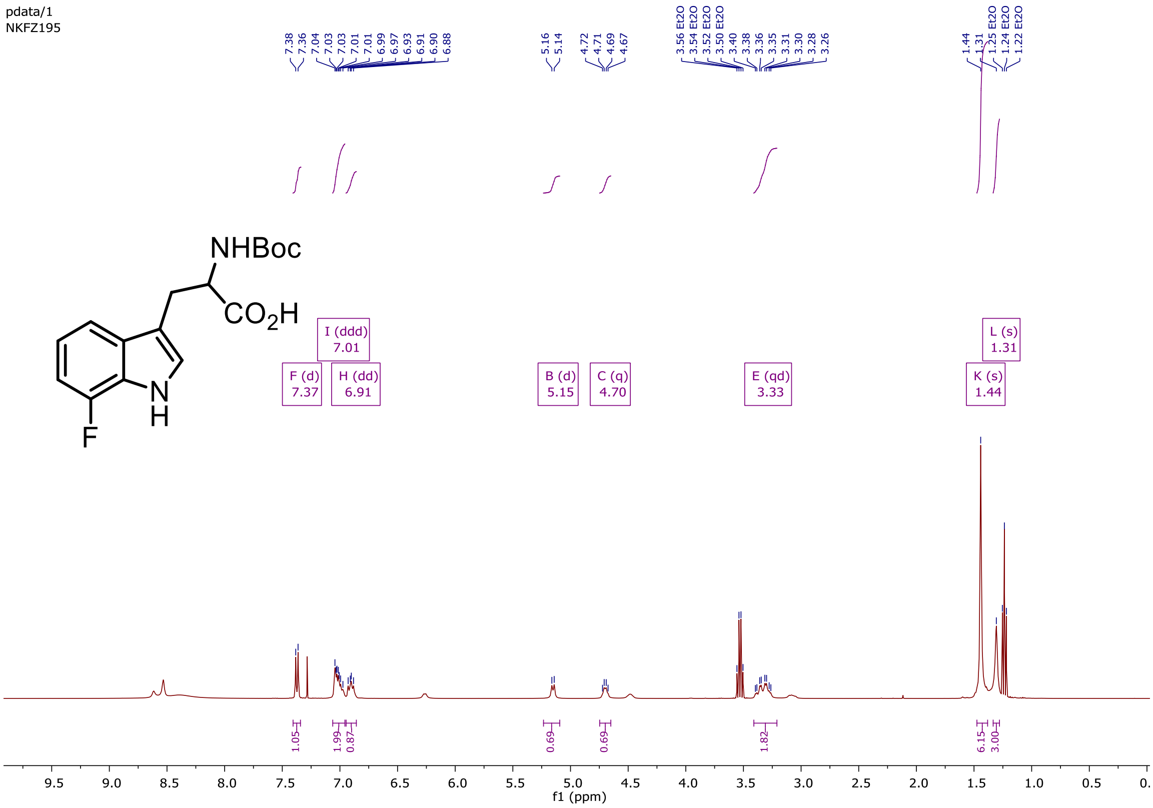

3.1.93 ^{13}C-NMR of 2-[(*tert*-butoxycarbonyl)amino]-3-(7-fluoro-1*H*-indol-3-yl)propanoic acid (S16)

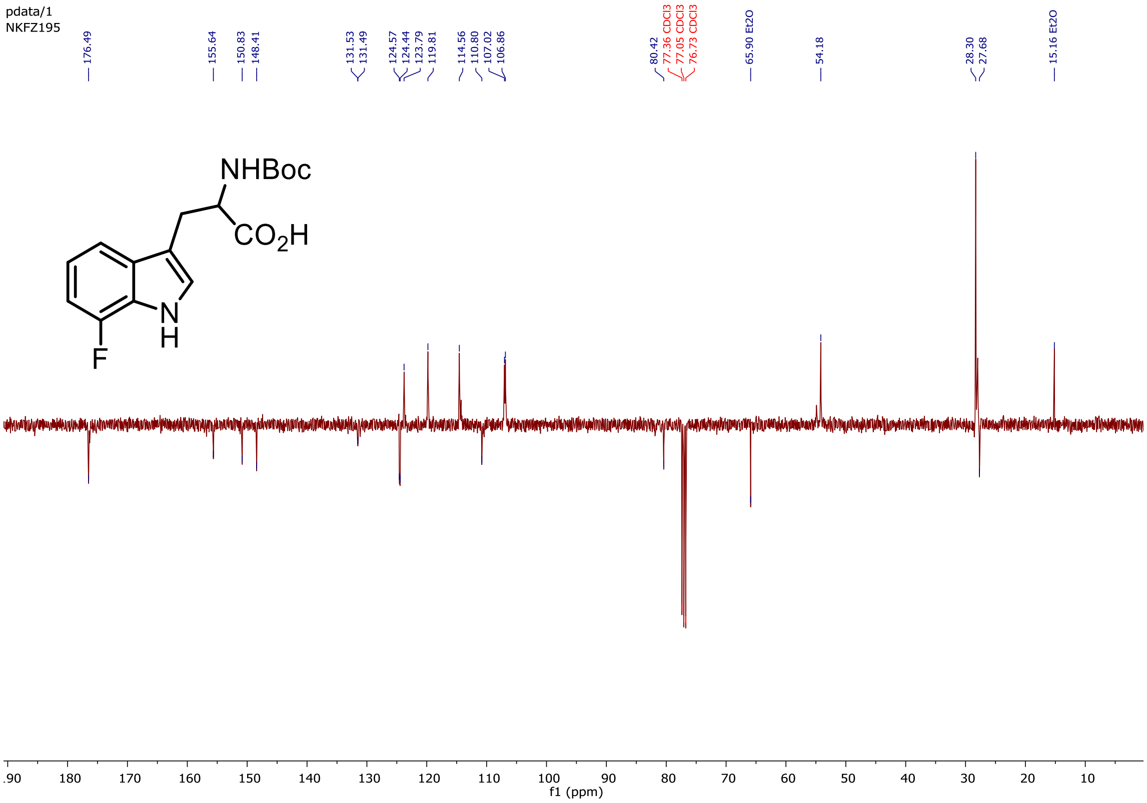

3.1.94 ^{19}F-NMR of 2-[(*tert*-butoxycarbonyl)amino]-3-(7-fluoro-1*H*-indol-3-yl)propanoic acid (S16)

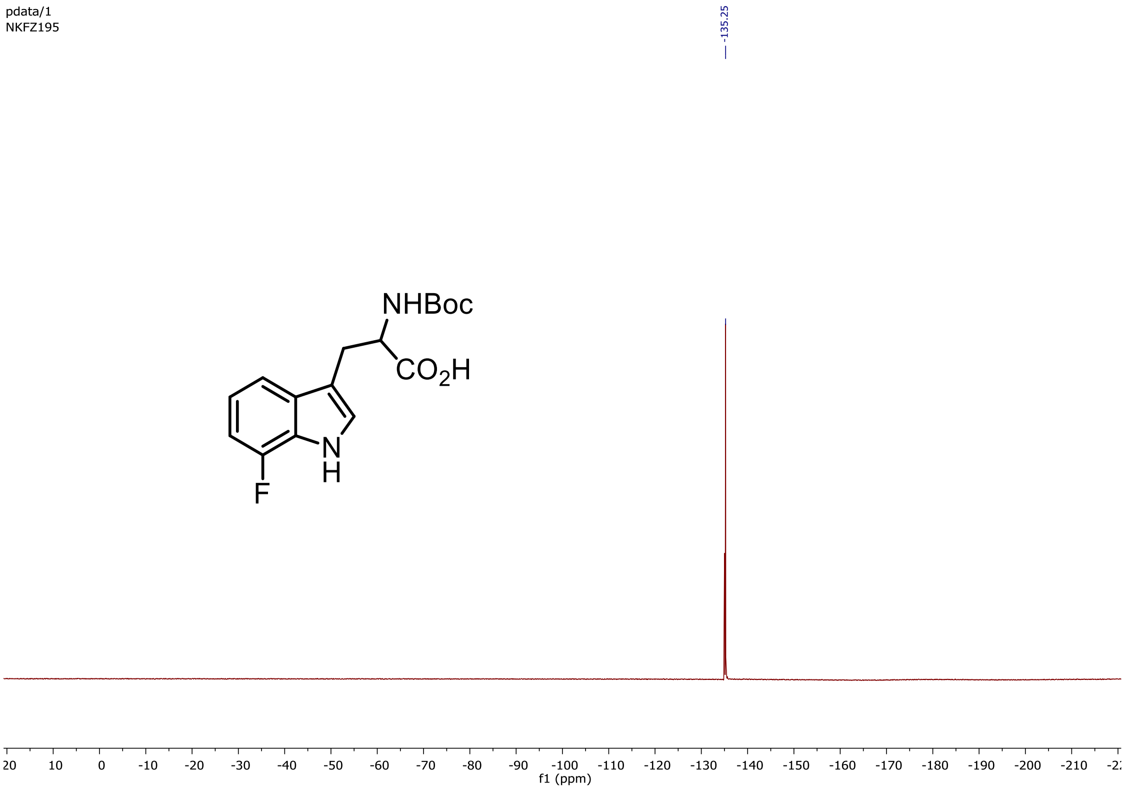

3.1.95 ^{1}H-NMR of *tert*-butyl 2-[(*tert*-butoxycarbonyl)amino]-3-(7-fluoro-1*H*-indol-3-yl)propanoate (S17)

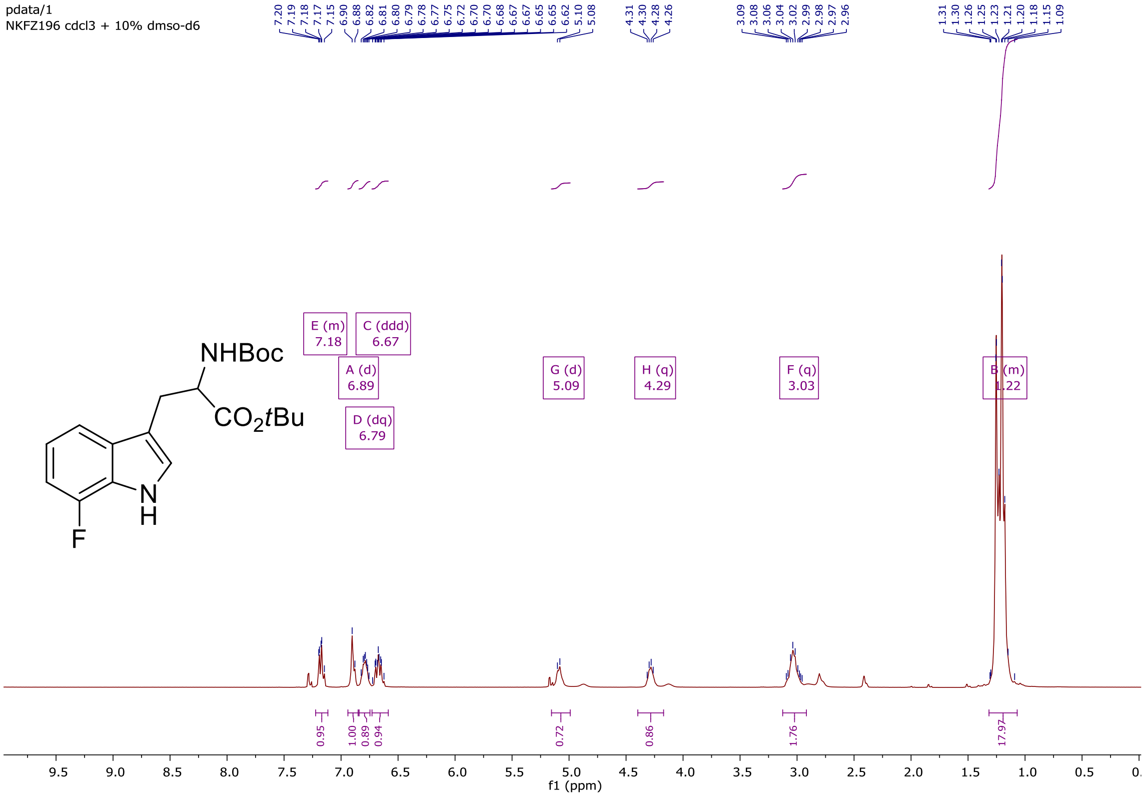

3.1.96 ^{13}C-NMR of *tert*-butyl 2-[(*tert*-butoxycarbonyl)amino]-3-(7-fluoro-1*H*-indol-3-yl)propanoate (S17)

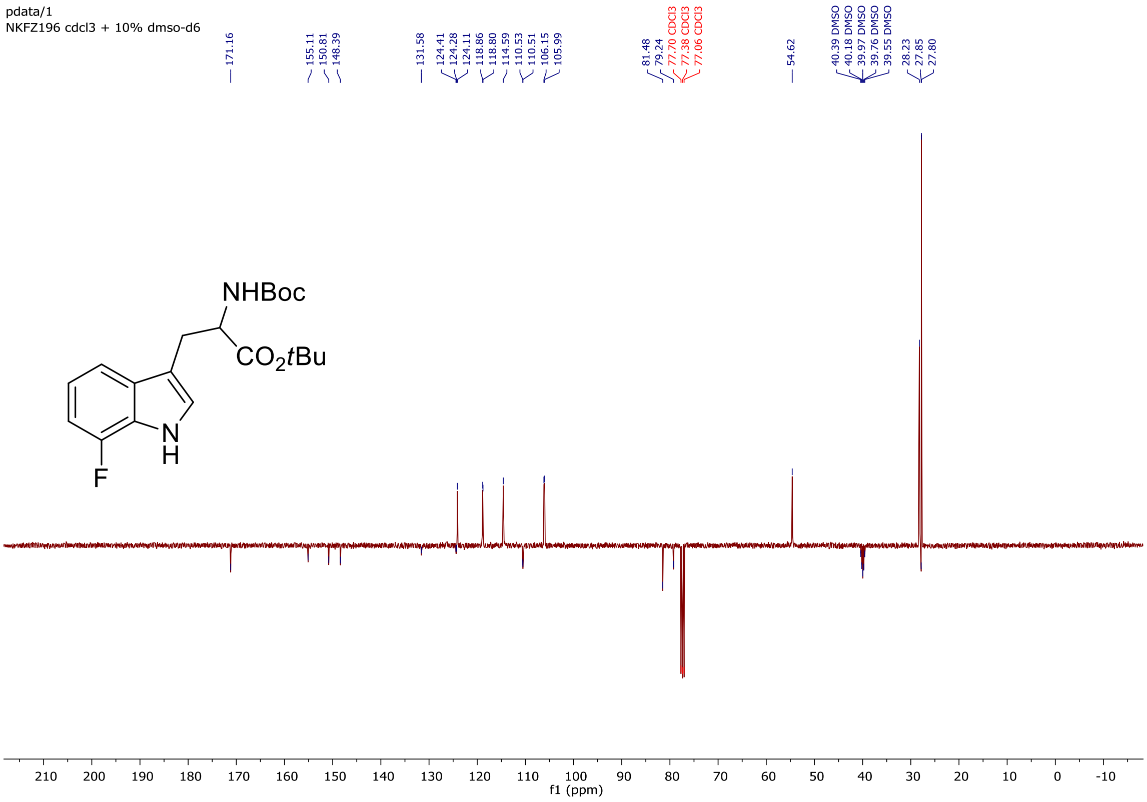

3.1.97 ^{19}F-NMR of *tert*-butyl 2-[(*tert*-butoxycarbonyl)amino]-3-(7-fluoro-1*H*-indol-3-yl)propanoate (S17)

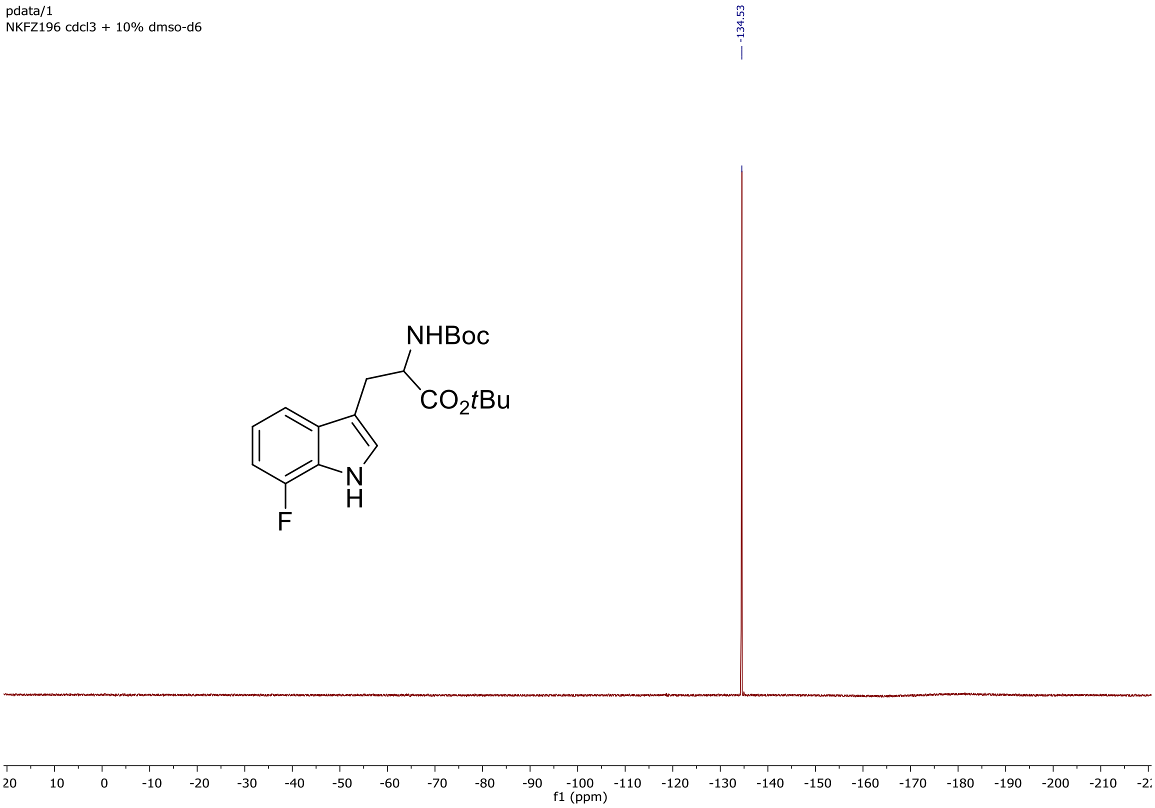

3.1.98 ^{1}H-NMR of 7-fluoro-5-(4,4,5,5-tetramethyl-1,3,2-dioxaborolan-2-yl)-1*H*-indole (S19)

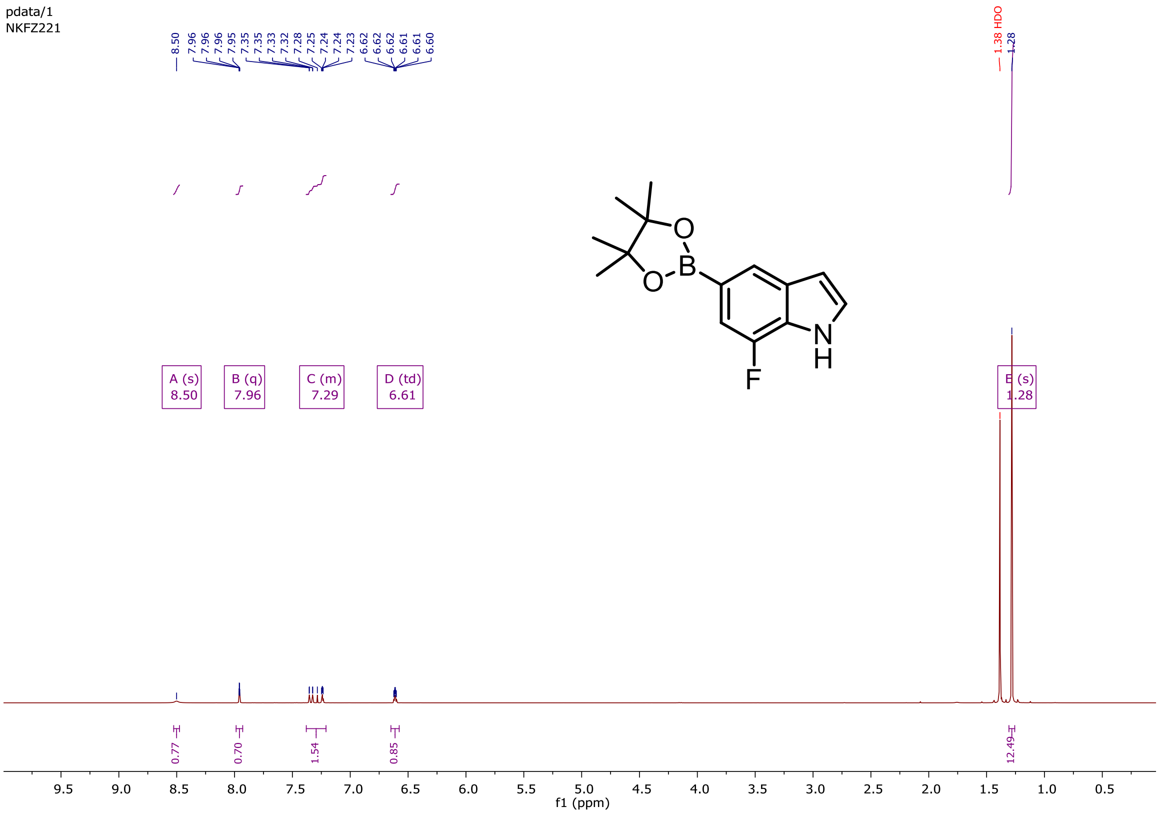

3.1.99 ^{13}C-NMR of 7-fluoro-5-(4,4,5,5-tetramethyl-1,3,2-dioxaborolan-2-yl)-1*H*-indole (S19)

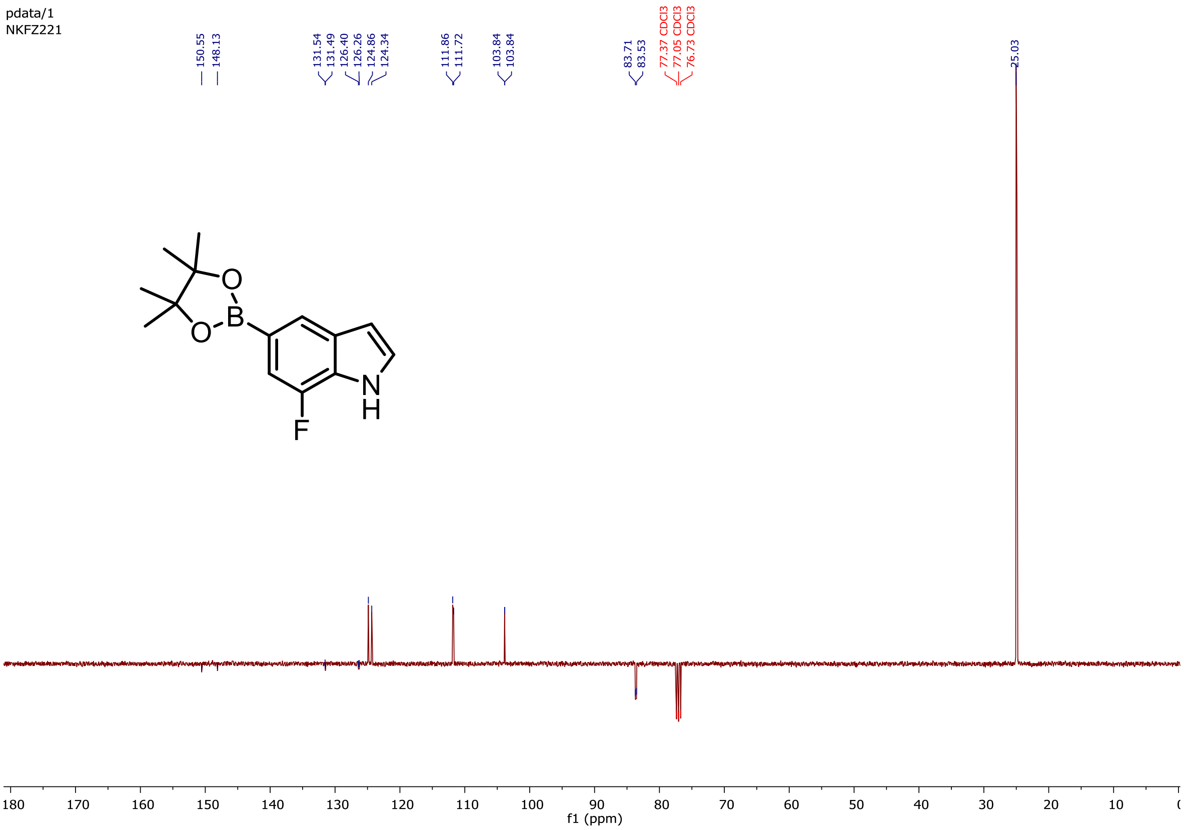

3.1.100 ^{19}F-NMR of 7-fluoro-5-(4,4,5,5-tetramethyl-1,3,2-dioxaborolan-2-yl)-1*H*-indole (S19)

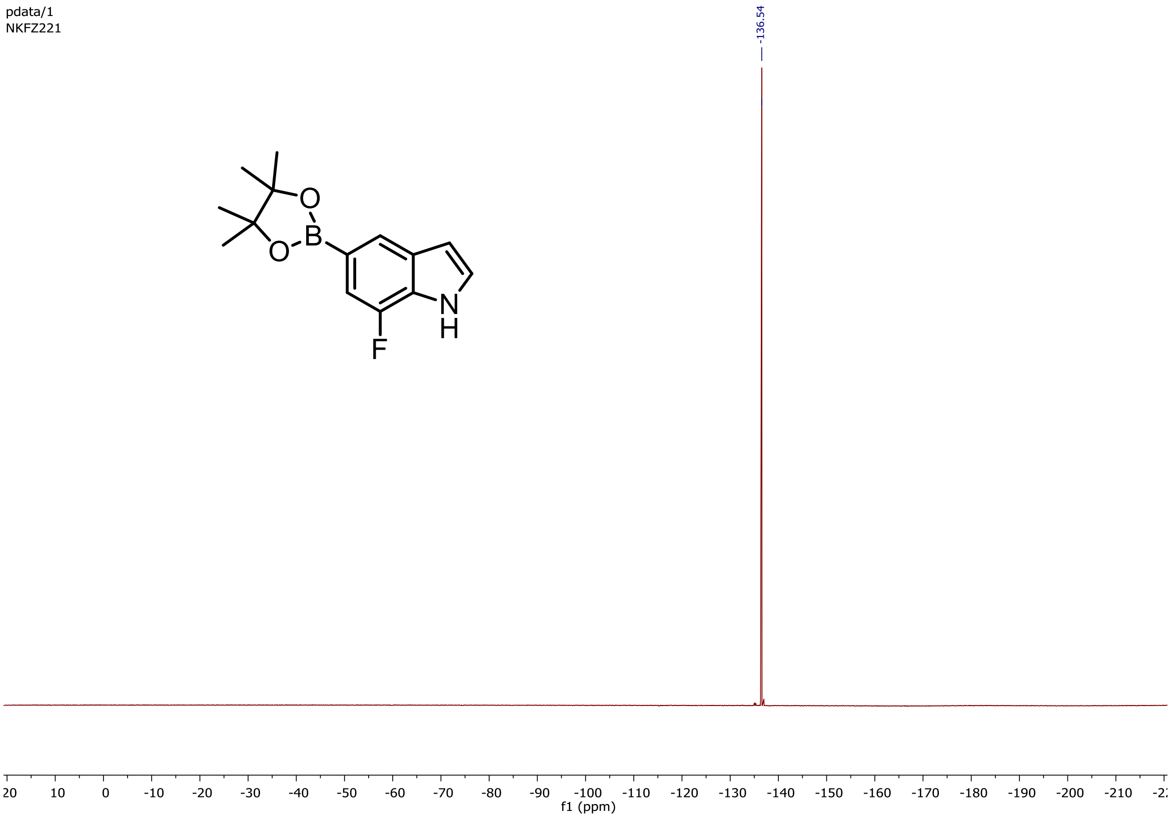

3.1.101 ^{1}H-NMR of 1-[7-fluoro-5-(4,4,5,5-tetramethyl-1,2,3-dioxaborolan-2-yl)-1*H*-indol-3-yl]-*N,N*-dimethylmethanamine (S20)

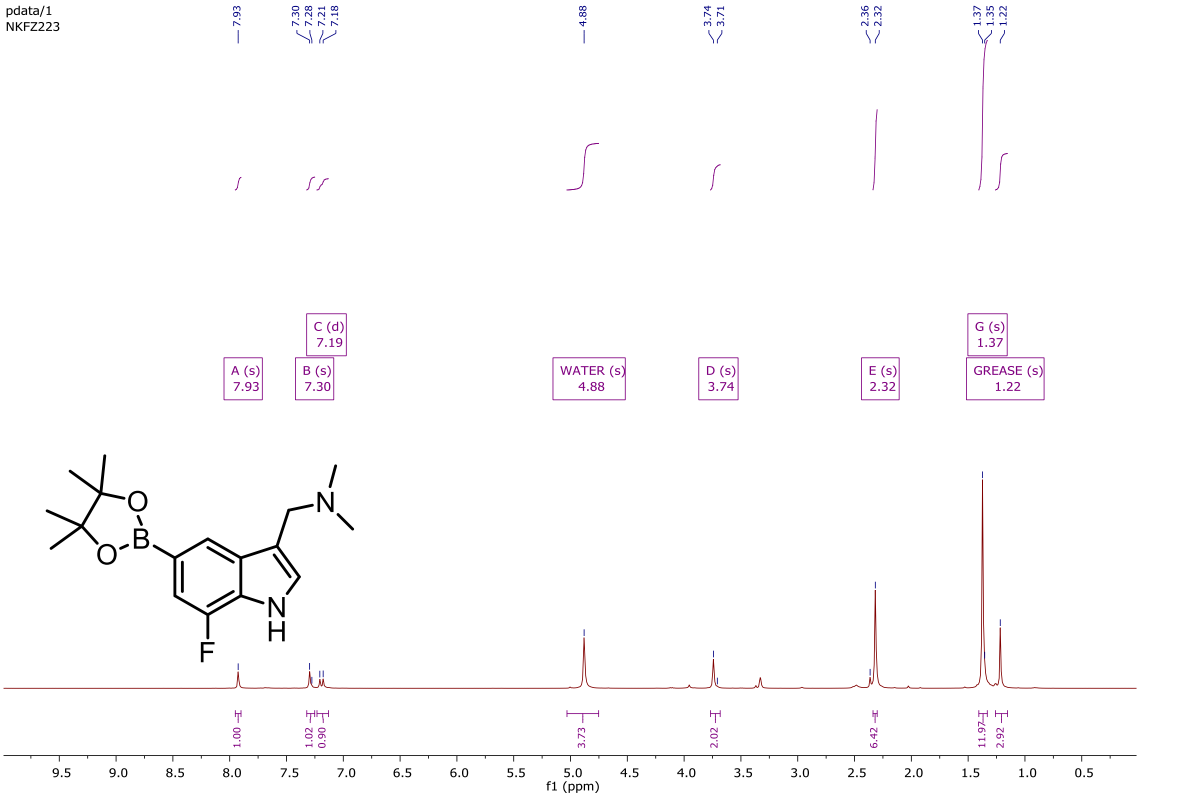

3.1.102 ^{13}C-NMR of 1-[7-fluoro-5-(4,4,5,5-tetramethyl-1,2,3-dioxaborolan-2-yl)-1*H*-indol-3-yl]-*N,N*-dimethylmethanamine (S20)

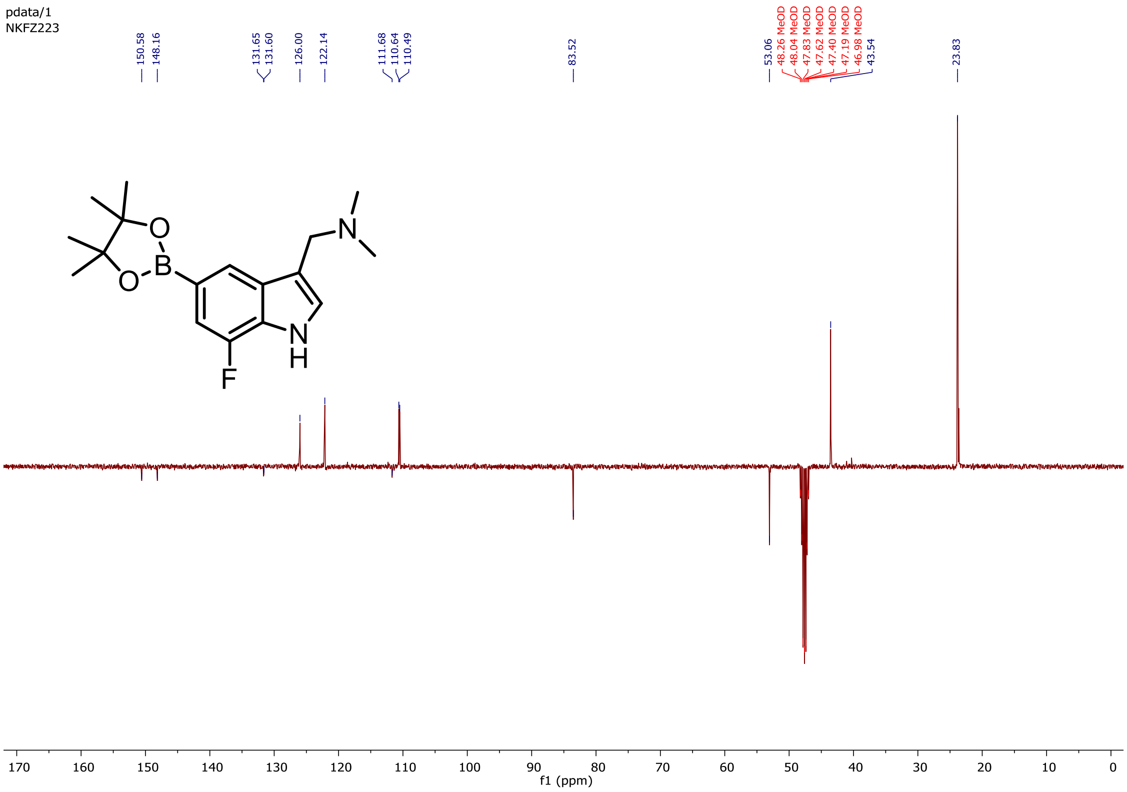

3.1.103 ^{19}F-NMR of 1-[7-fluoro-5-(4,4,5,5-tetramethyl-1,2,3-dioxaborolan-2-yl)-1*H*-indol-3-yl]-*N,N*-dimethylmethanamine (S20)

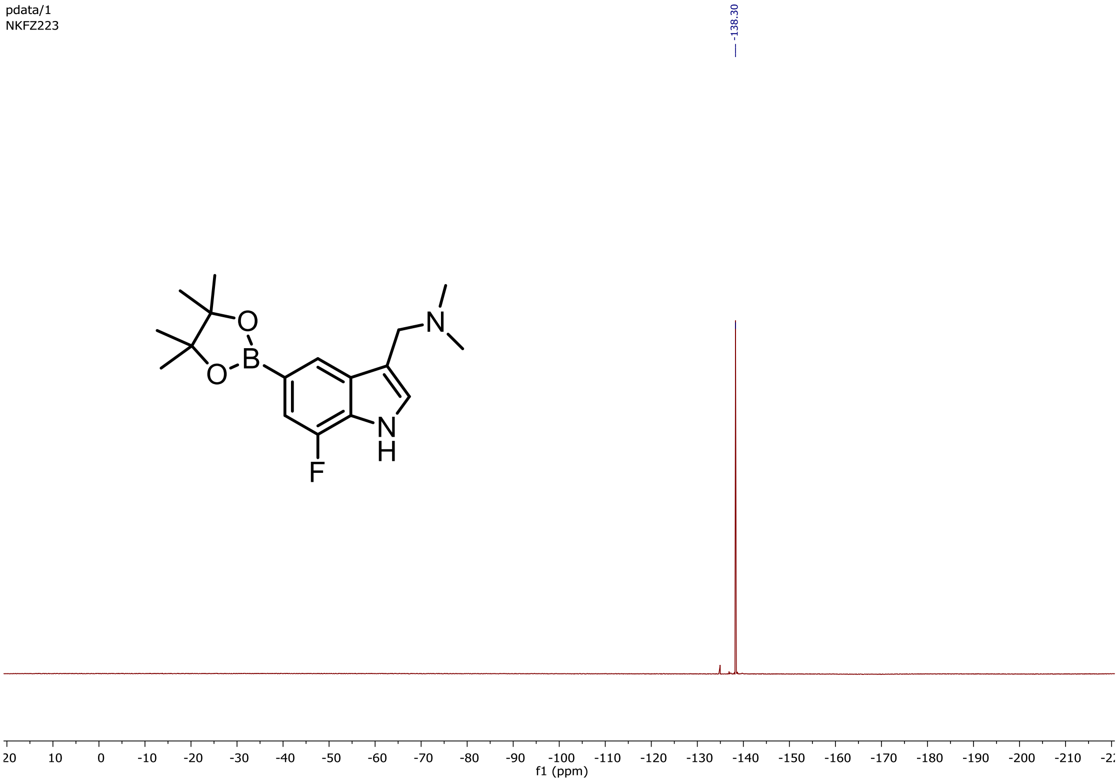

3.1.104 ¹H-NMR of 1-[7-fluoro-5-(4,4,5,5-tetramethyl-1,3,2-dioxaborolan-2-yl)-1*H*-indol-3-yl]-*N,N,N*-trimethylmethanaminium iodide (S21)

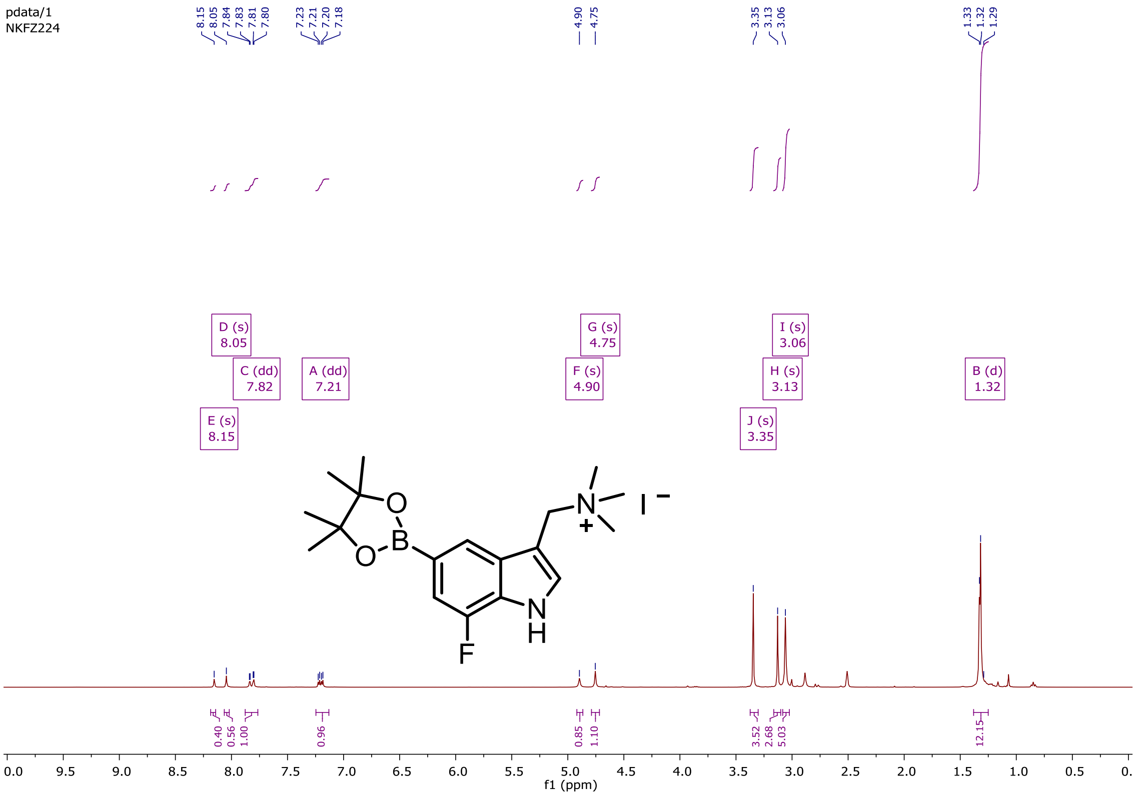

3.1.105 ¹³C-NMR of 1-[7-fluoro-5-(4,4,5,5-tetramethyl-1,3,2-dioxaborolan-2-yl)-1*H*-indol-3-yl]-*N,N,N*-trimethylmethanaminium iodide (S21)

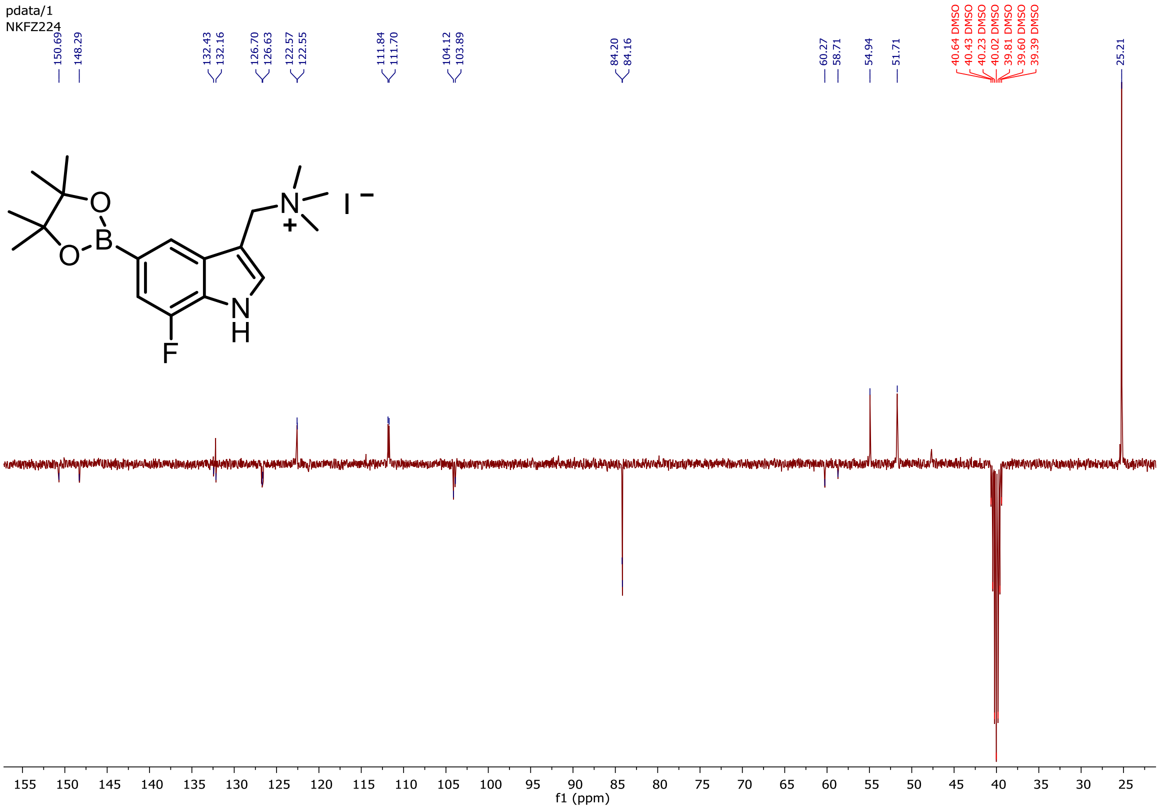

3.1.106 ^{19}F-NMR of 1-[7-fluoro-5-(4,4,5,5-tetramethyl-1,3,2-dioxaborolan-2-yl)-1*H*-indol-3-yl]-*N,N,N*-trimethylmethanaminium iodide (S21)

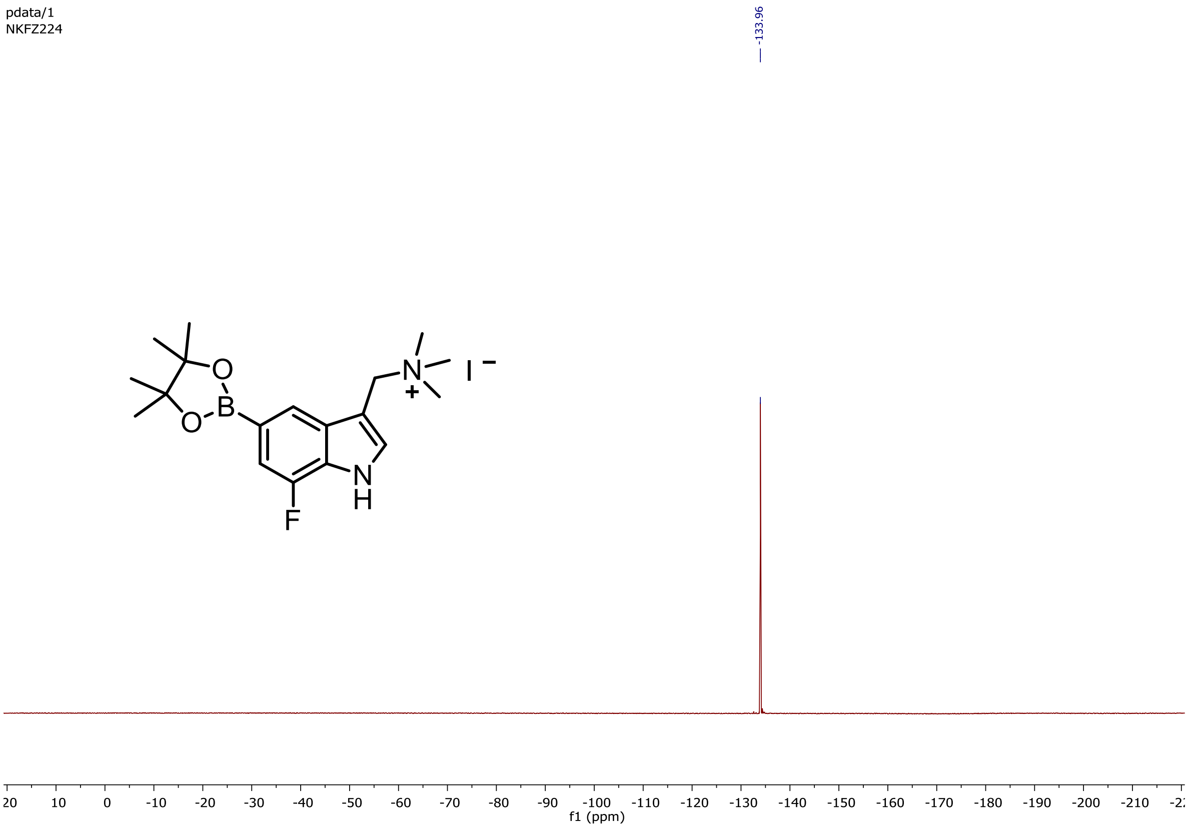

3.1.107 ^{1}H-NMR of (*S,S*)-Ni(II)-BPB-5-BPin-7-FTrp (S22)

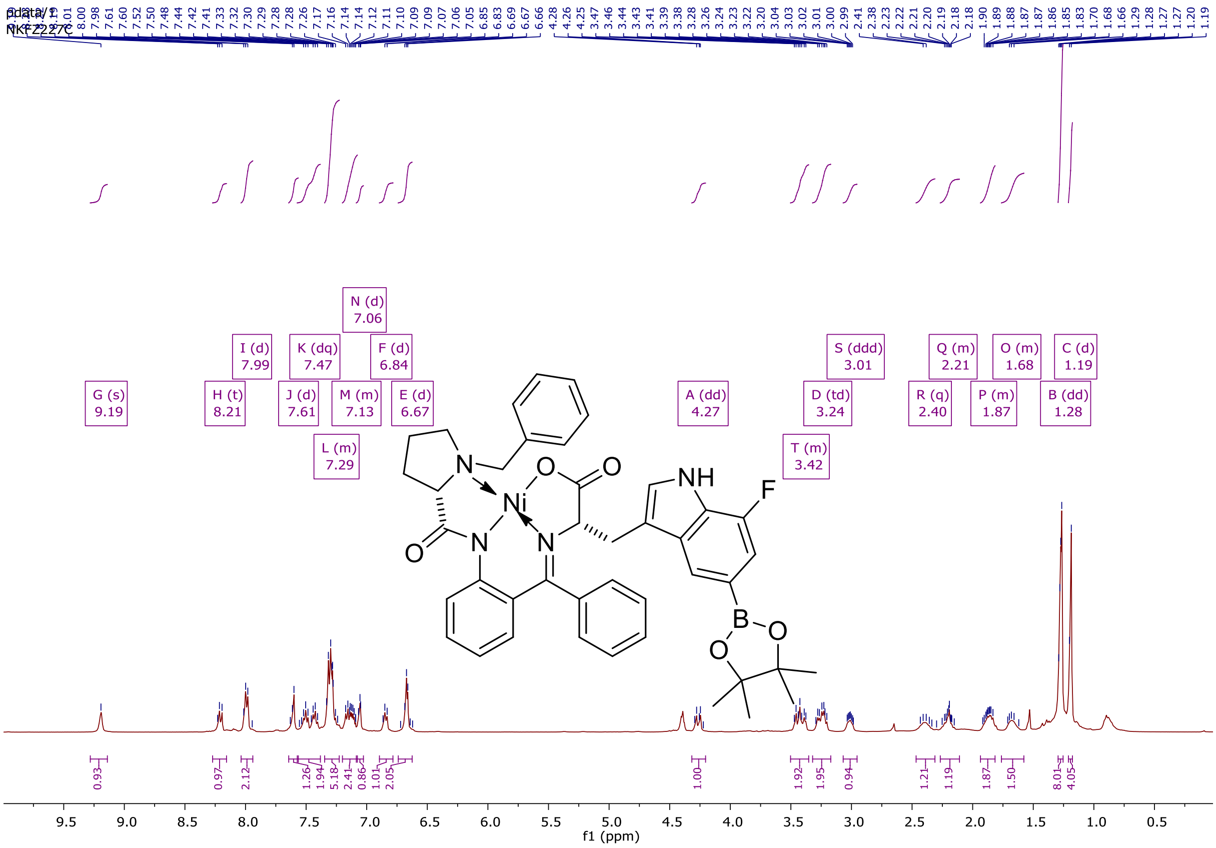

3.1.108 ^{13}C-NMR of (*S*,*S*)-Ni(II)-BPB-5-BPin-7-FTrp (S22)

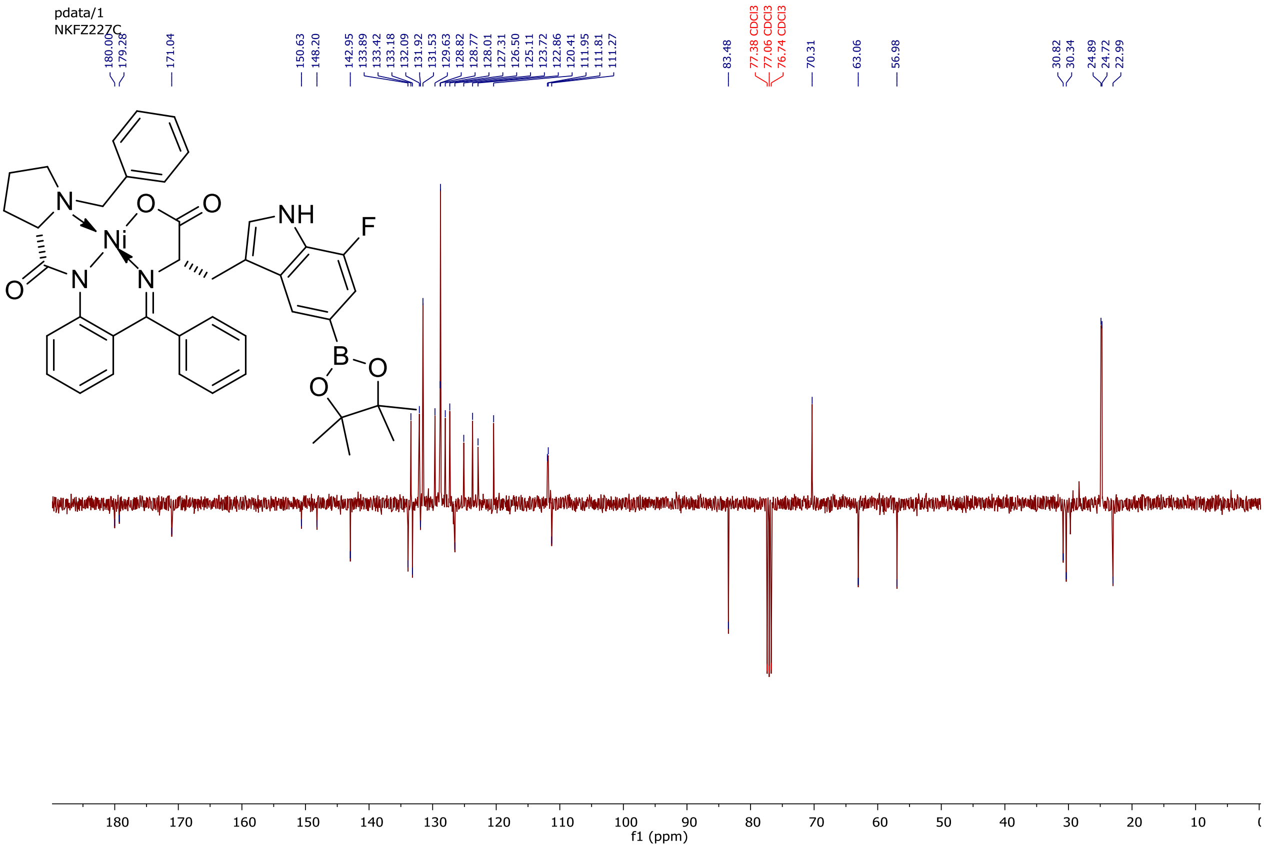

3.1.109 ^{19}F-NMR of (*S*,*S*)-Ni(II)-BPB-5-BPin-7-FTrp (S22)

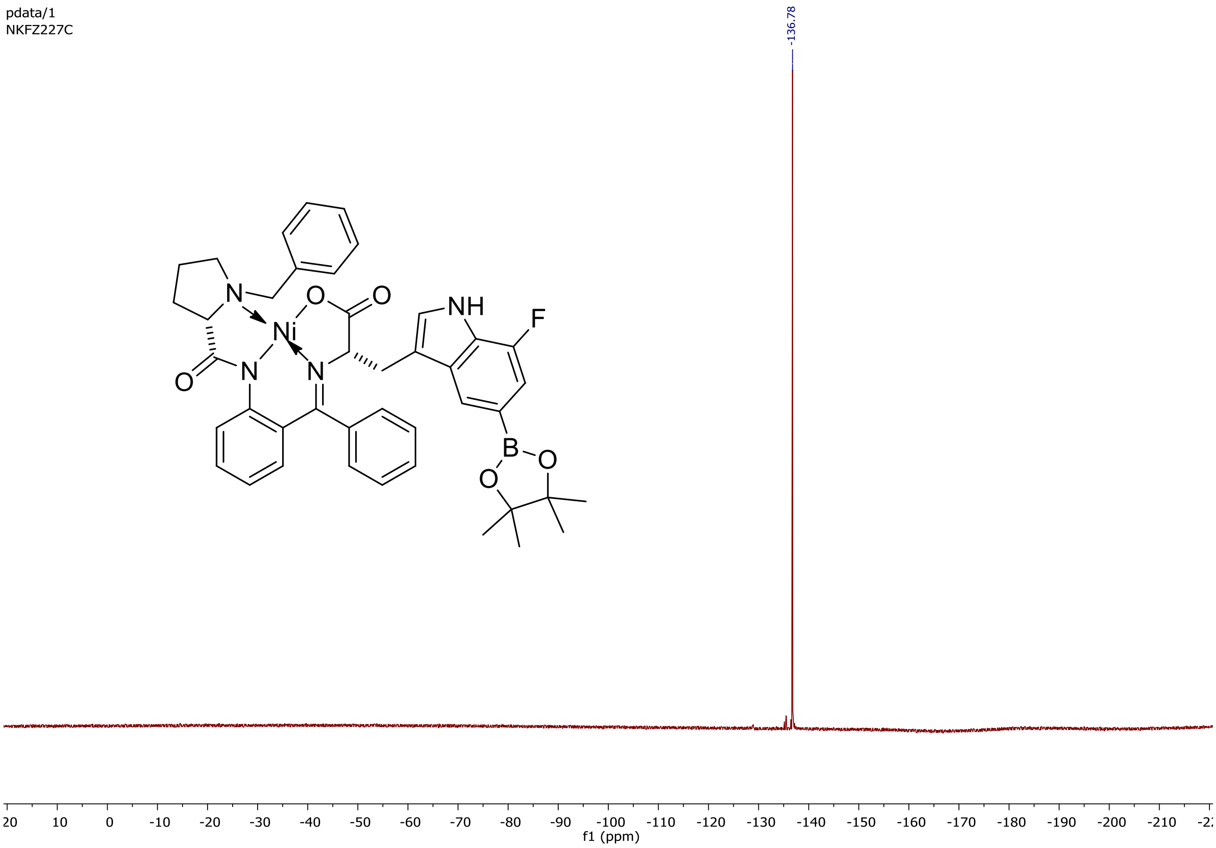

3.1.110 ^{1}H-NMR for (*S*)-2-[(*tert*-butoxycarbonyl)amino]-3-[7-fluoro-5-(4,4,5,5-tetramethyl-1,3,2-dioxaborolan-2-yl)-1*H*-indol-3-yl]propanoic acid (S23)

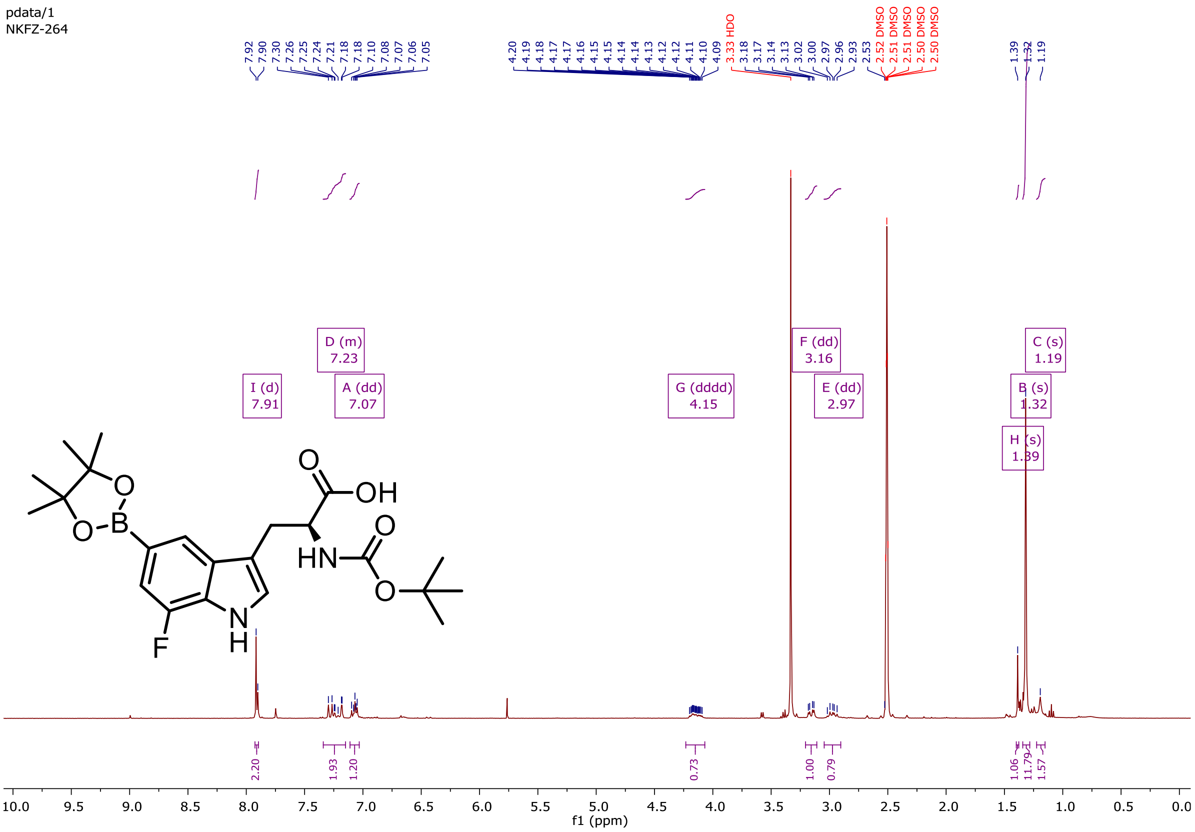

3.1.111 ^{13}C-NMR for (*S*)-2-[(*tert*-butoxycarbonyl)amino]-3-[7-fluoro-5-(4,4,5,5-tetramethyl-1,3,2-dioxaborolan-2-yl)-1*H*-indol-3-yl]propanoic acid (S23)

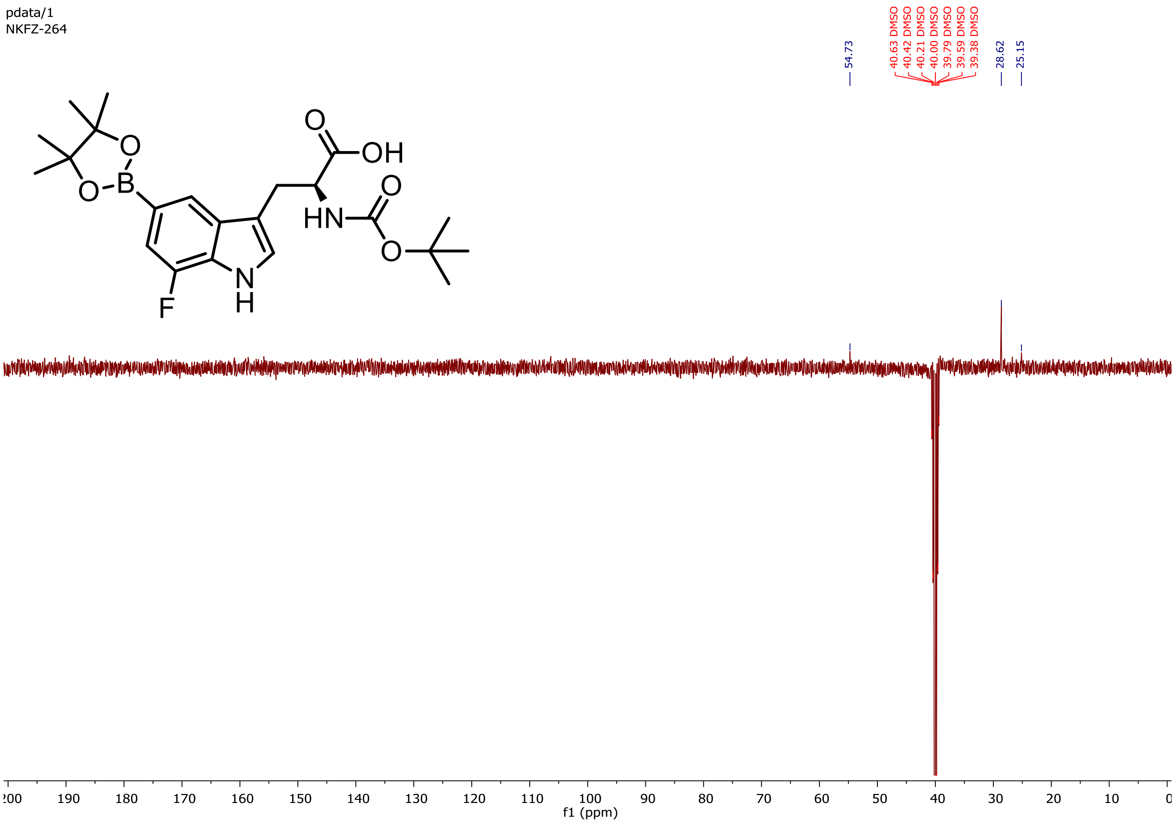

3.1.112 ^{19}F-NMR for (*S*)-2-[(*tert*-butoxycarbonyl)amino]-3-[7-fluoro-5-(4,4,5,5-tetramethyl-1,3,2-dioxaborolan-2-yl)-1*H*-indol-3-yl]propanoic acid (S23)

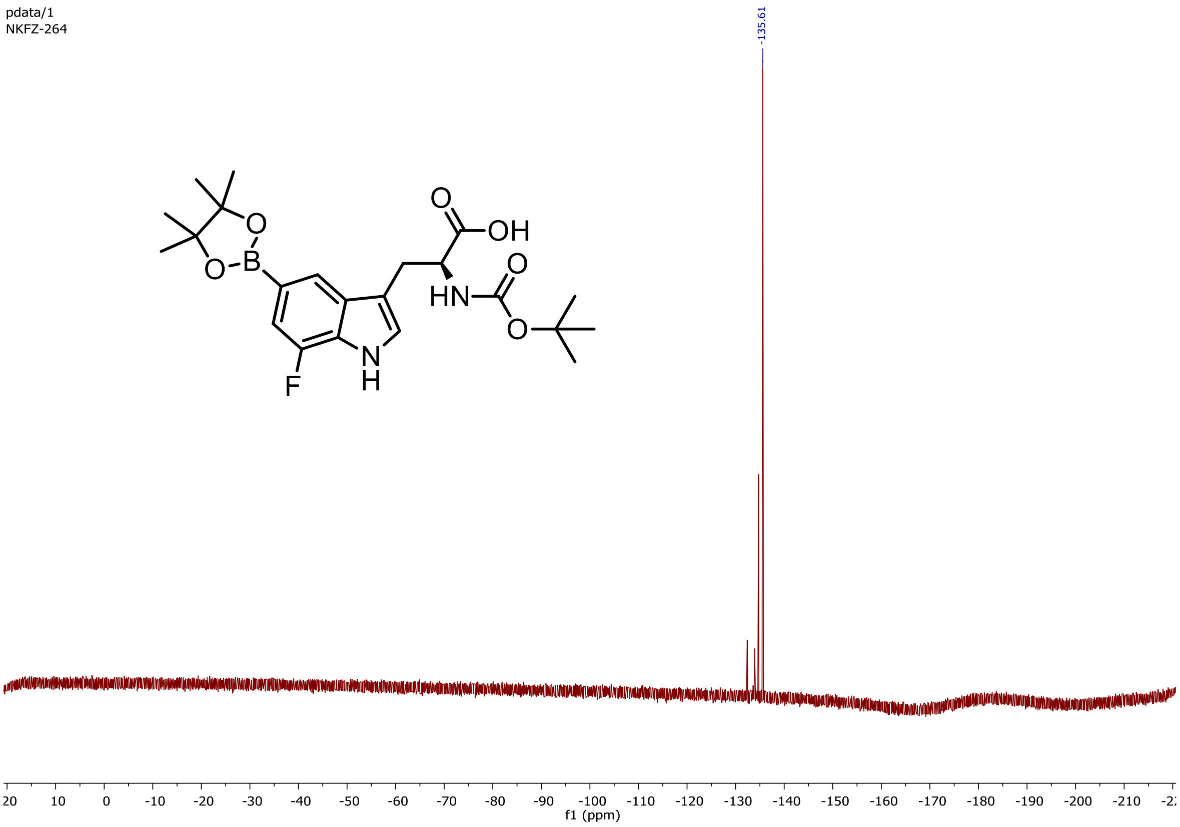

3.1.113 ^{1}H-NMR of *tert*-butyl (7-fluoro-1*H*-indol-5-yl) carbonate (S25)

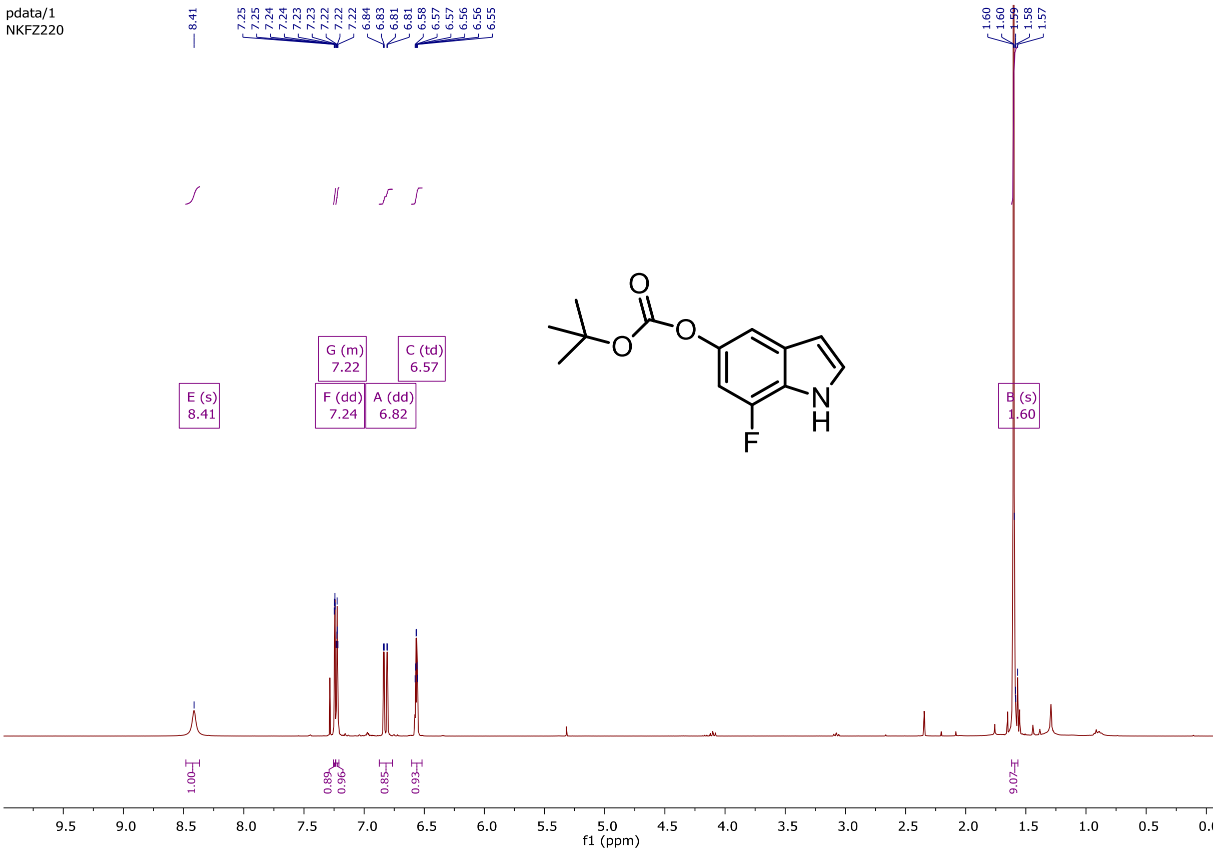

3.1.114 ^{13}C-NMR of *tert*-butyl (7-fluoro-1*H*-indol-5-yl) carbonate (S25)

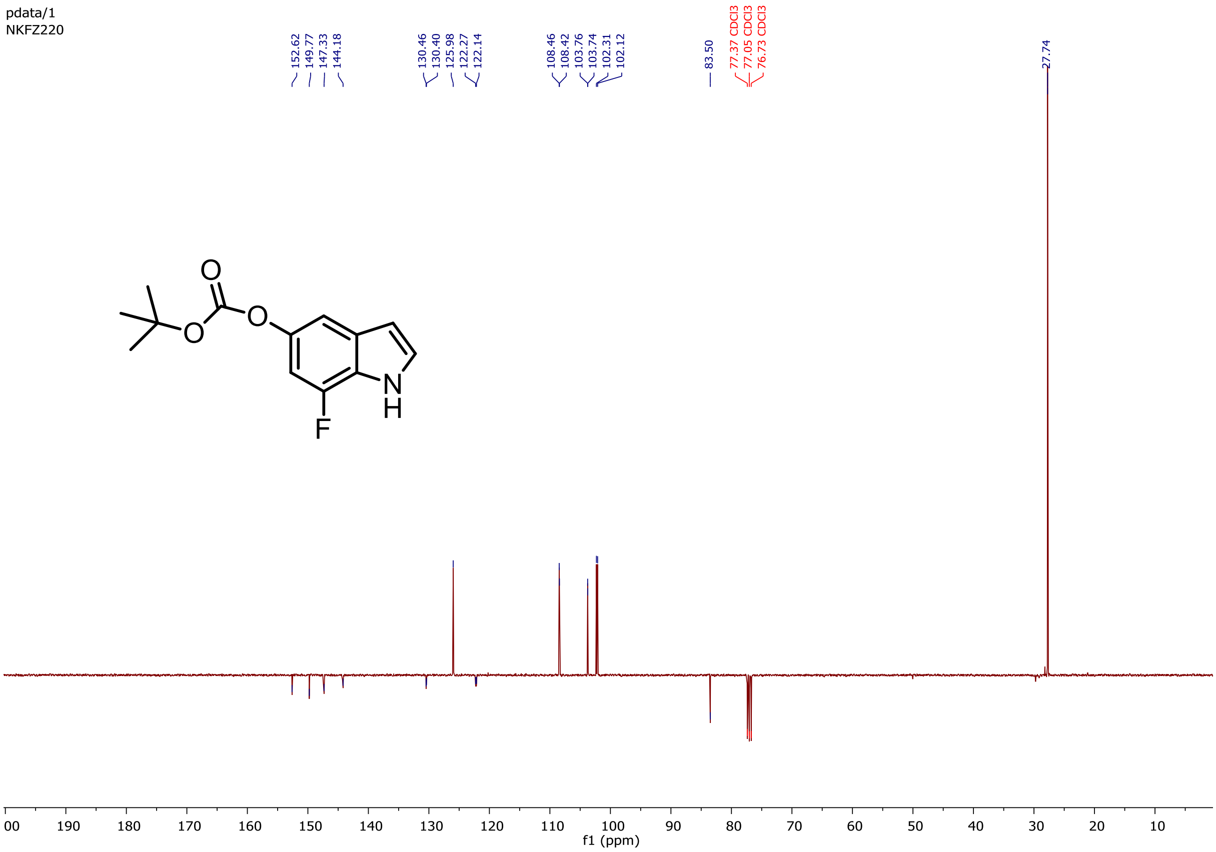

3.1.115 ^{19}F-NMR of *tert*-butyl (7-fluoro-1*H*-indol-5-yl) carbonate (S25)

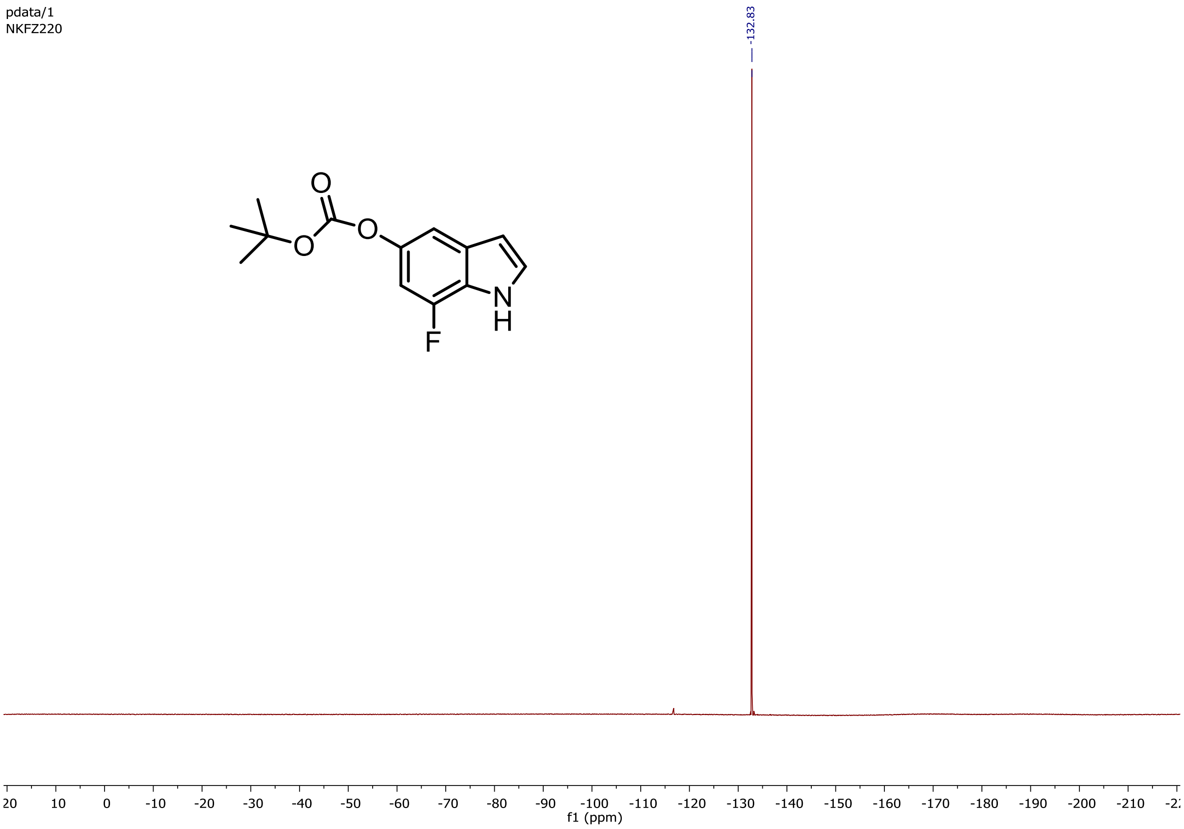

3.1.116 ^{1}H-NMR of *tert*-butyl {3-[(dimethylamino)methyl]-7-fluoro-1*H*-indol-5-yl}carbonate (S26)

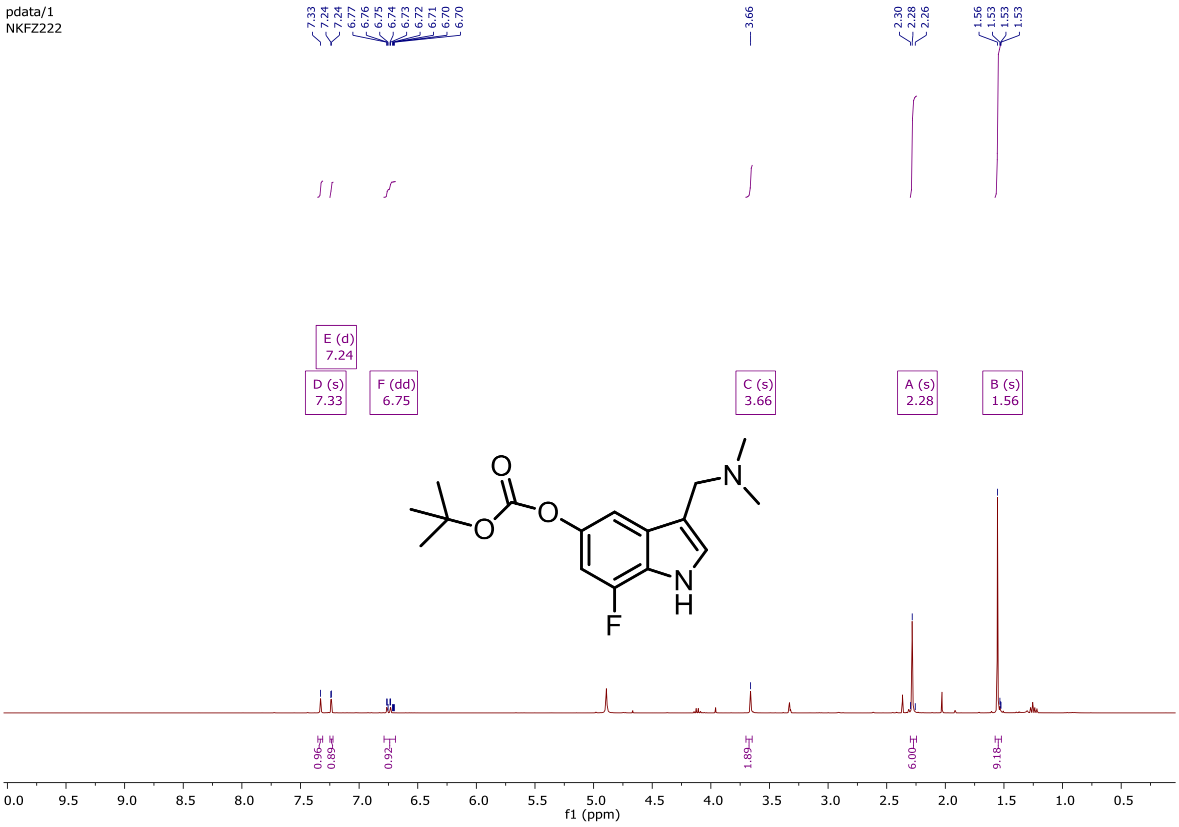

3.1.117 ^{13}C-NMR of *tert*-butyl {3-[(dimethylamino)methyl]-7-fluoro-1*H*-indol-5-yl}carbonate (S26)

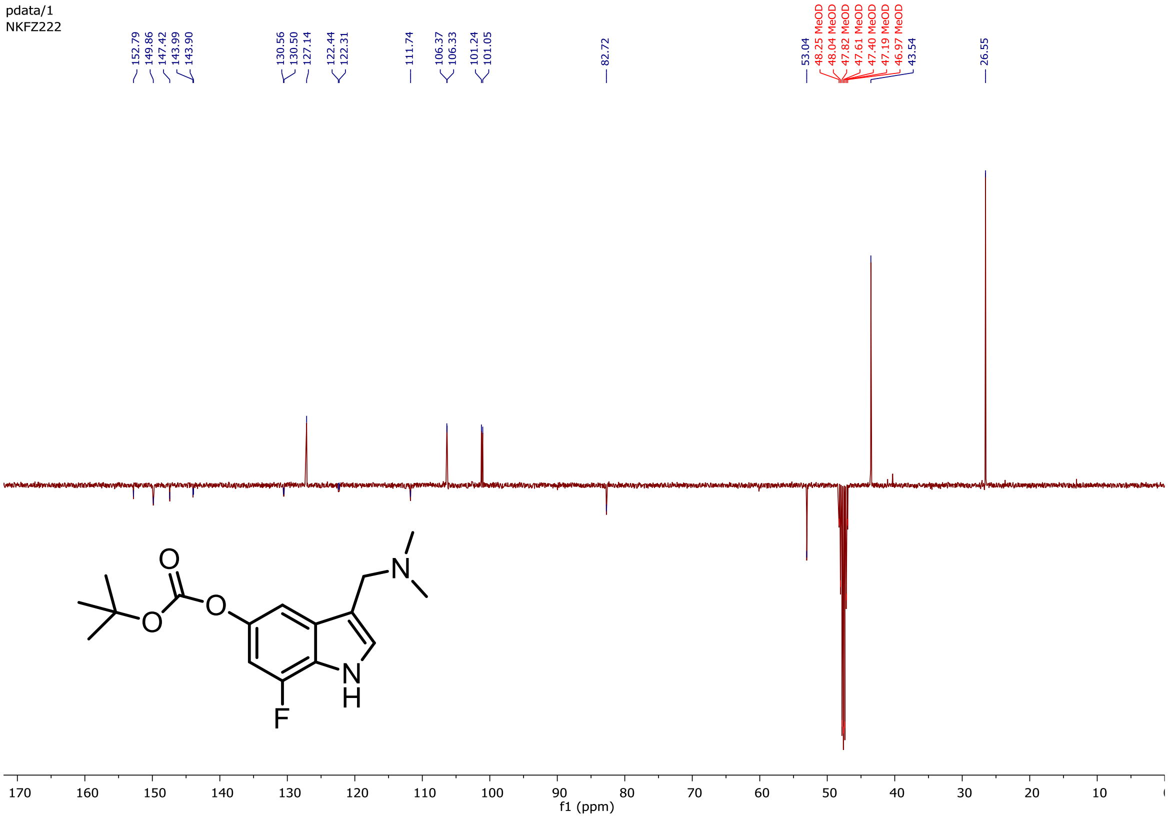

3.1.118 ^{19}F-NMR of *tert*-butyl {3-[(dimethylamino)methyl]-7-fluoro-1*H*-indol-5-yl}carbonate (S26)

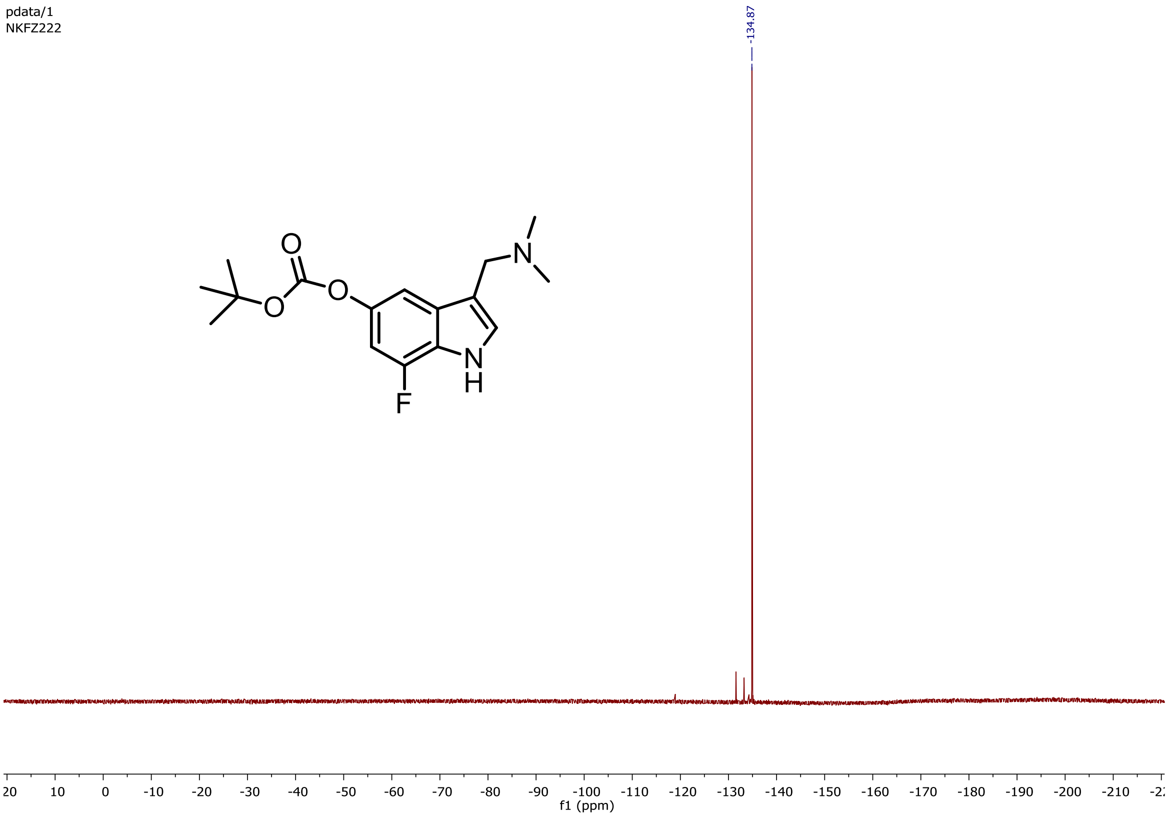

3.1.119 ^{1}H-NMR of 1-{5-[(*tert*-butoxycarbonyl)oxy]-7-fluoro-1*H*-indol-3-yl}-*N,N,N*-trimethylmethanaminium iodide (S27)

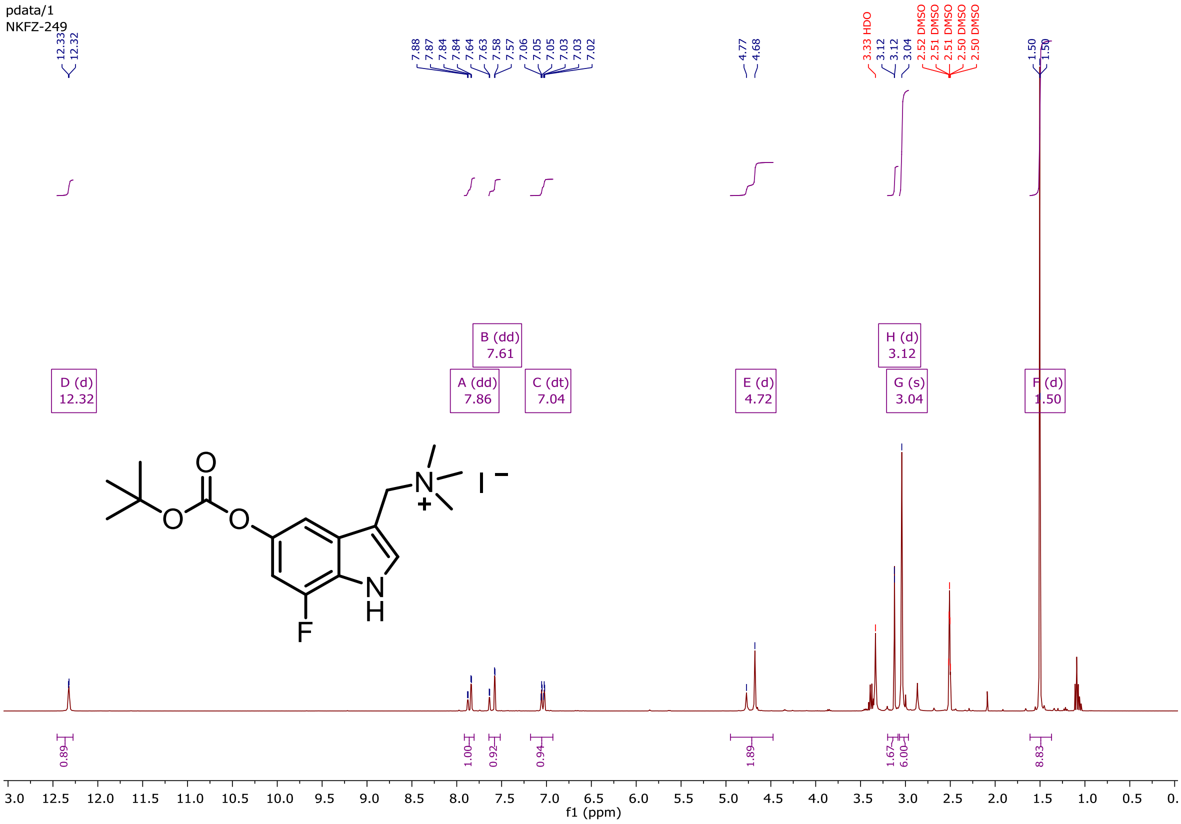

3.1.120 ^{13}C-NMR of 1-{5-[(*tert*-butoxycarbonyl)oxy]-7-fluoro-1*H*-indol-3-yl}-*N,N,N*-trimethylmethanaminium iodide (S27)

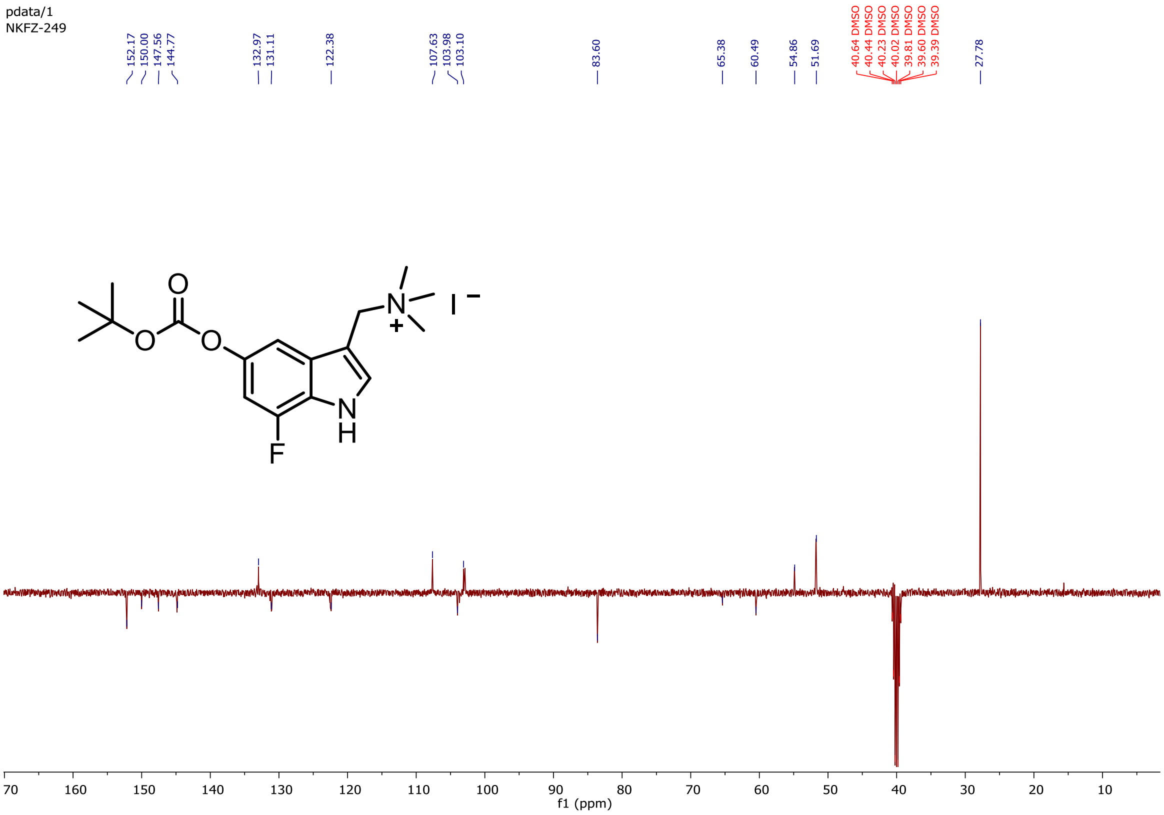

3.1.121 ^{19}F-NMR of 1-{5-[(*tert*-butoxycarbonyl)oxy]-7-fluoro-1*H*-indol-3-yl}-*N,N,N*-trimethylmethanaminium iodide (S27)

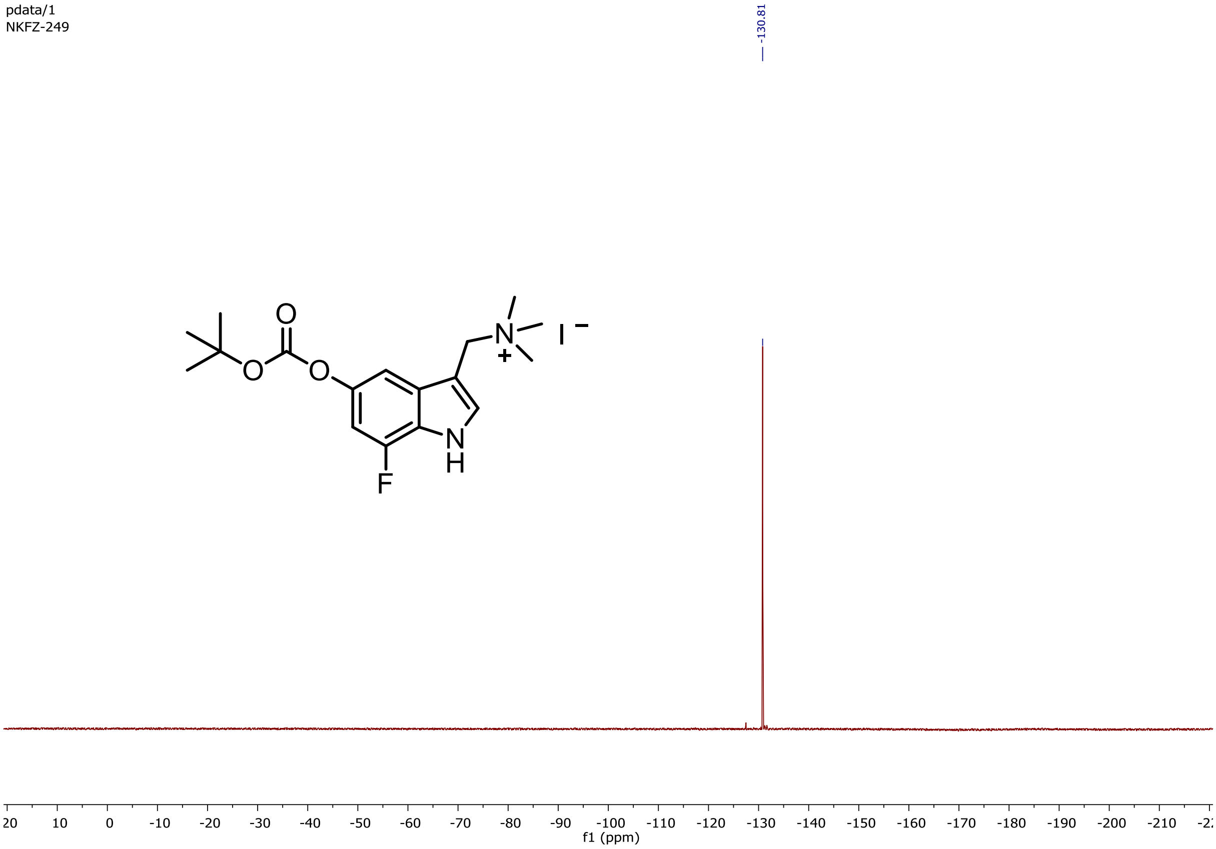

3.1.122 ¹H-NMR of (*S,S*)-Ni(II)-BPB-5-BocO-7-FTrp (S28)

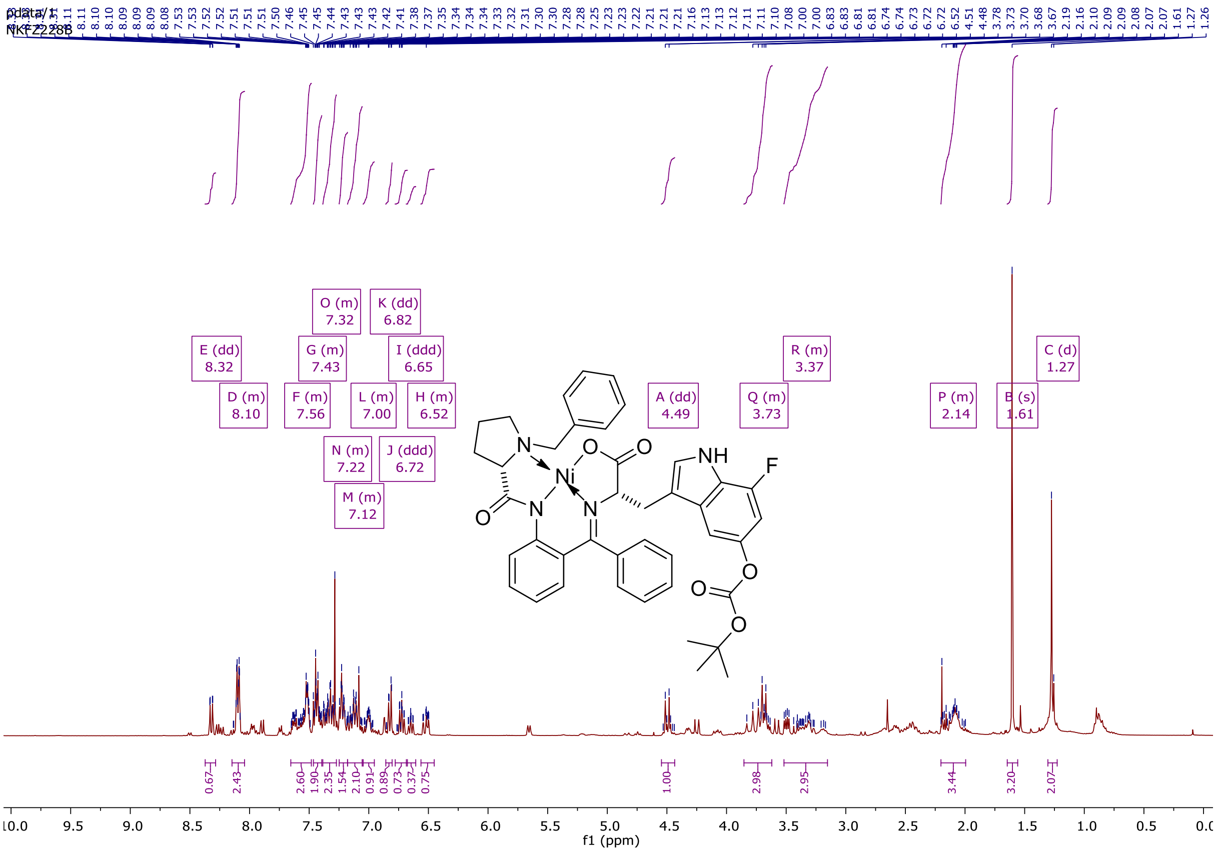

3.1.123 ¹³C-NMR of (*S,S*)-Ni(II)-BPB-5-BocO-7-FTrp (S28)

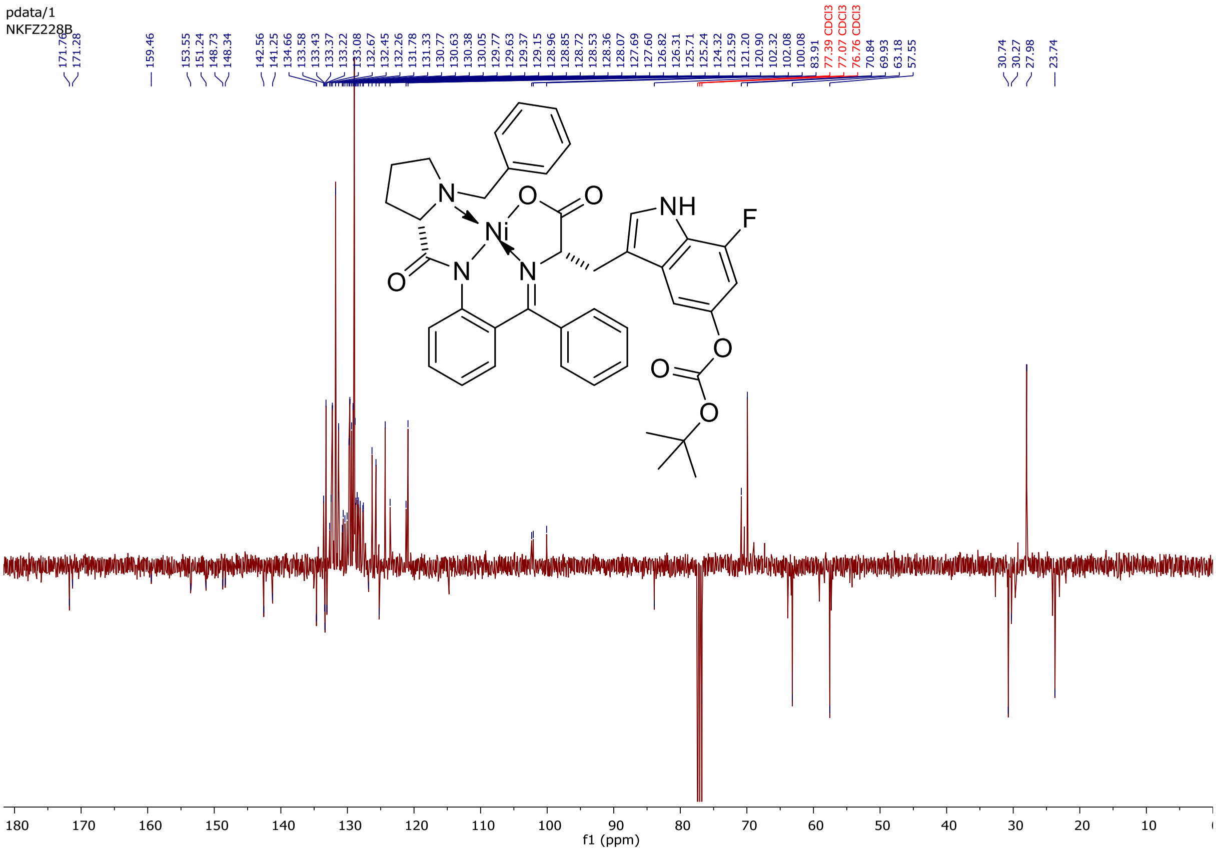

3.1.124 ^{19}F-NMR of (*S*,*S*)-Ni(II)-BPB-5-BocO-7-FTrp (S28)

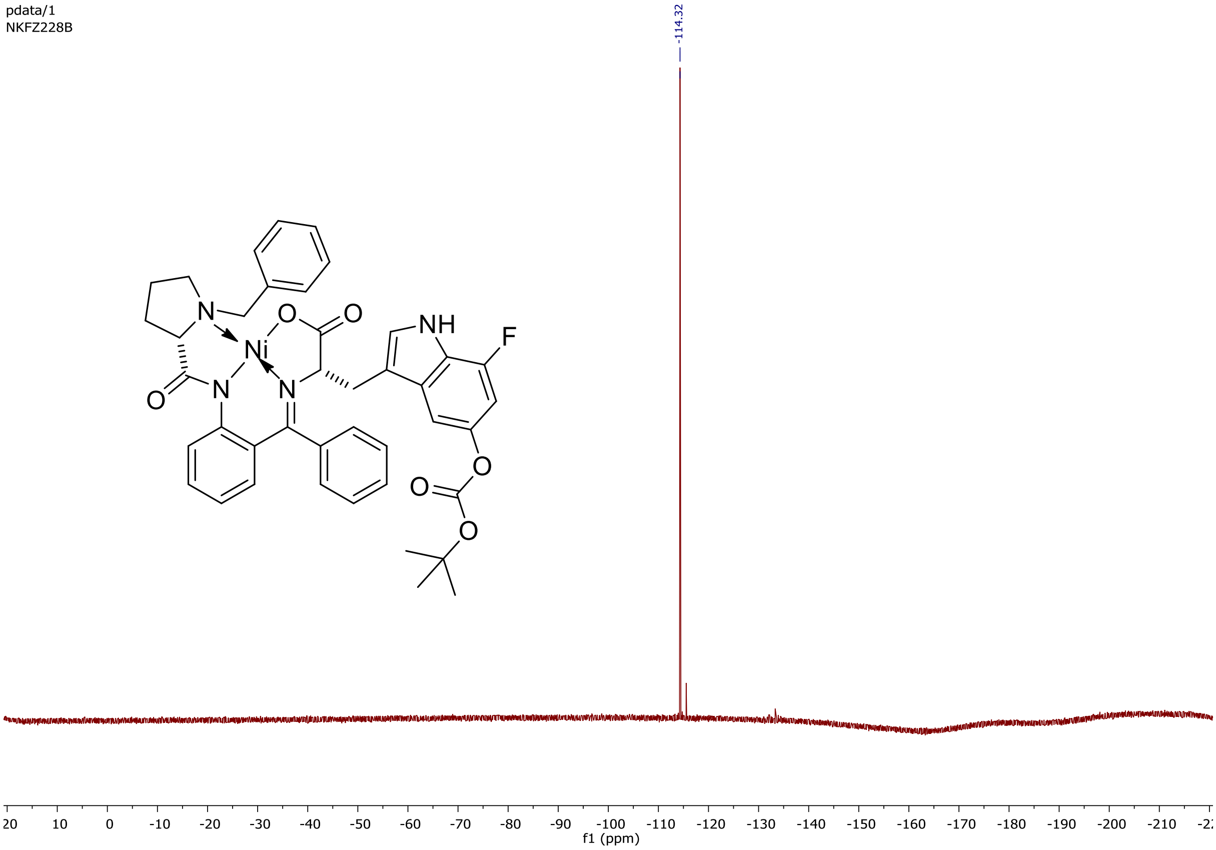

3.2 HPLC-Data

3.2.1 (*S*)-N_{In}-Methyl-6-[^{18}F]fluorotryptophan: HPLC chromatograms and calibration curve for the determination of molar activity

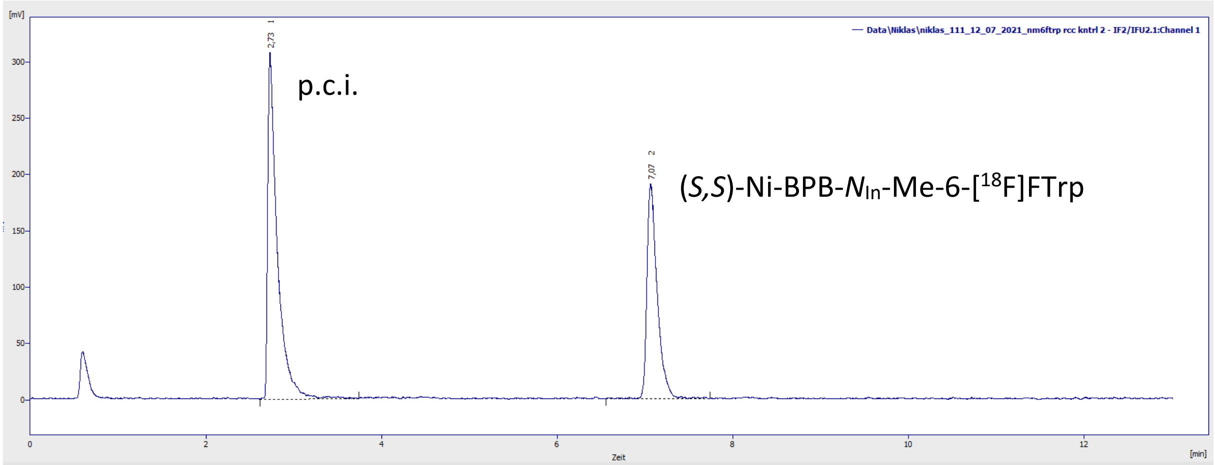

HPLC chromatogram of the crude radiolabeled intermediate (*S*,*S*)-Ni-BPB-N_{In}-Me-6-[^{18}F]FTrp. Abbreviation: p.c.i. – post-column injection.

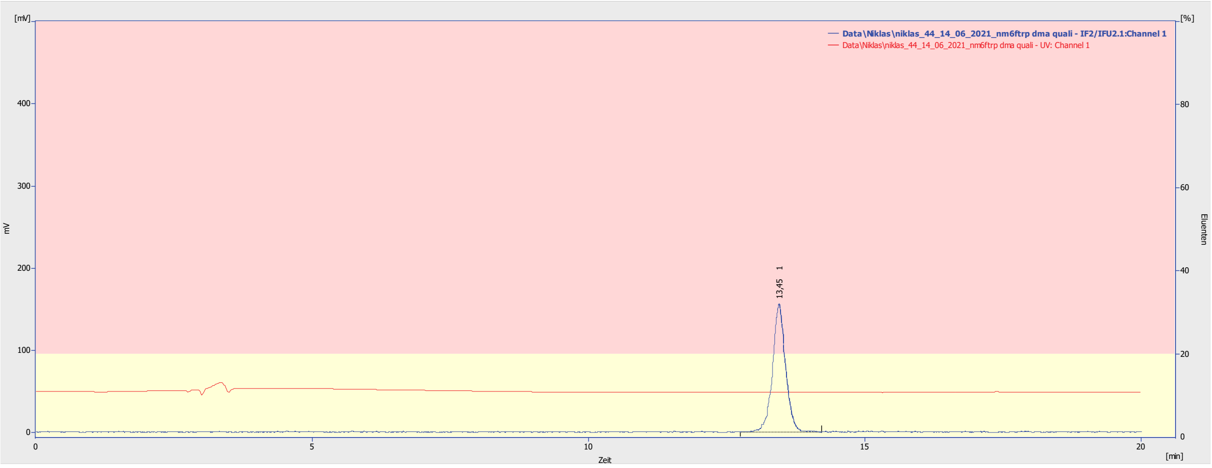

HPLC chromatogram of purified (*S*)-N_{In}-Me-6-[^{18}F]FTrp [column: Synergi Hydro-RP, 4 µm, 80 Å, 250 × 4.6 mm equipped with the appropriate SecurityGuard™ cartridge (2 × 3 mm) (Phenomenex, Aschaffenburg, Germany); eluent: 20% MeCN (0.1% TFA); flow rate: 1 mL/min; detection: radioactivity (blue trace), UV λ = 210 nm (red trace)].

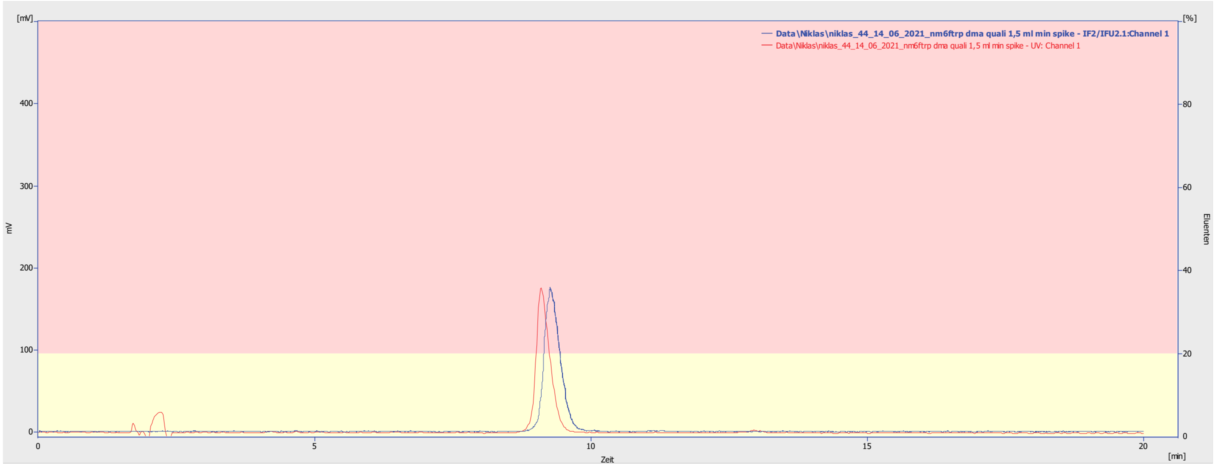

HPLC chromatogram of purified (S)-N_{In}-Me-6-[^{18}F]FTrp spiked with the non-radioactive reference compound. The radioactive trace (tracer) is shown in blue, while the UV trace (reference compound, λ = 210 nm) is shown in red [column: Synergi Hydro-RP, 4 µm, 80 Å, 250 × 4.6 mm equipped with the appropriate SecurityGuard™ cartridge (2 × 3 mm) (Phenomenex, Aschaffenburg, Germany); eluent: 20% MeCN (0.1% TFA); flow rate: 1.5 mL/min].

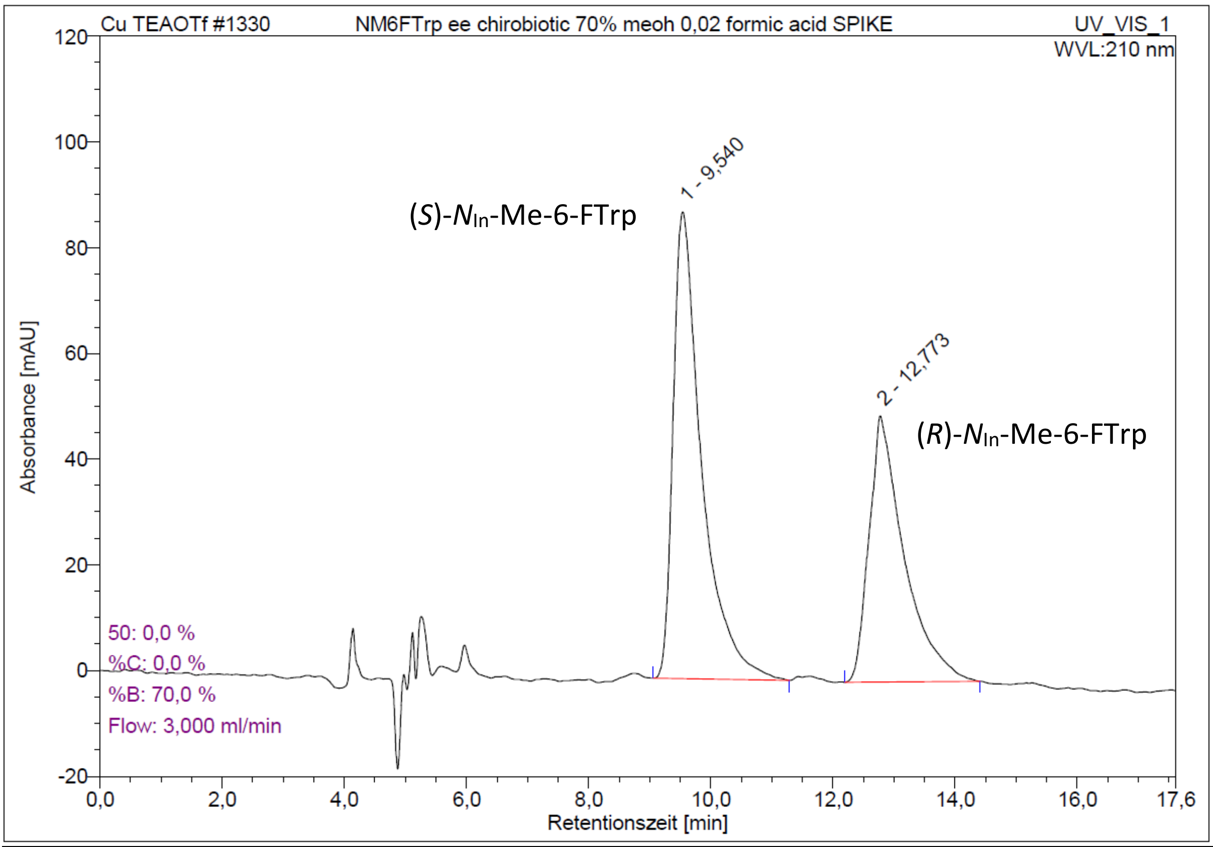

HPLC chromatogram of racemic N_{In}-Me-6-FTrp [column: Astec Chirobiotic T, 5 µm, 250 × 10 mm (Supelco Analytical) equipped with the appropriate SecurityGuard™ cartridge (2 × 3 mm); eluent: 70% MeOH (0.02% formic acid); flow rate: 3 mL/min].

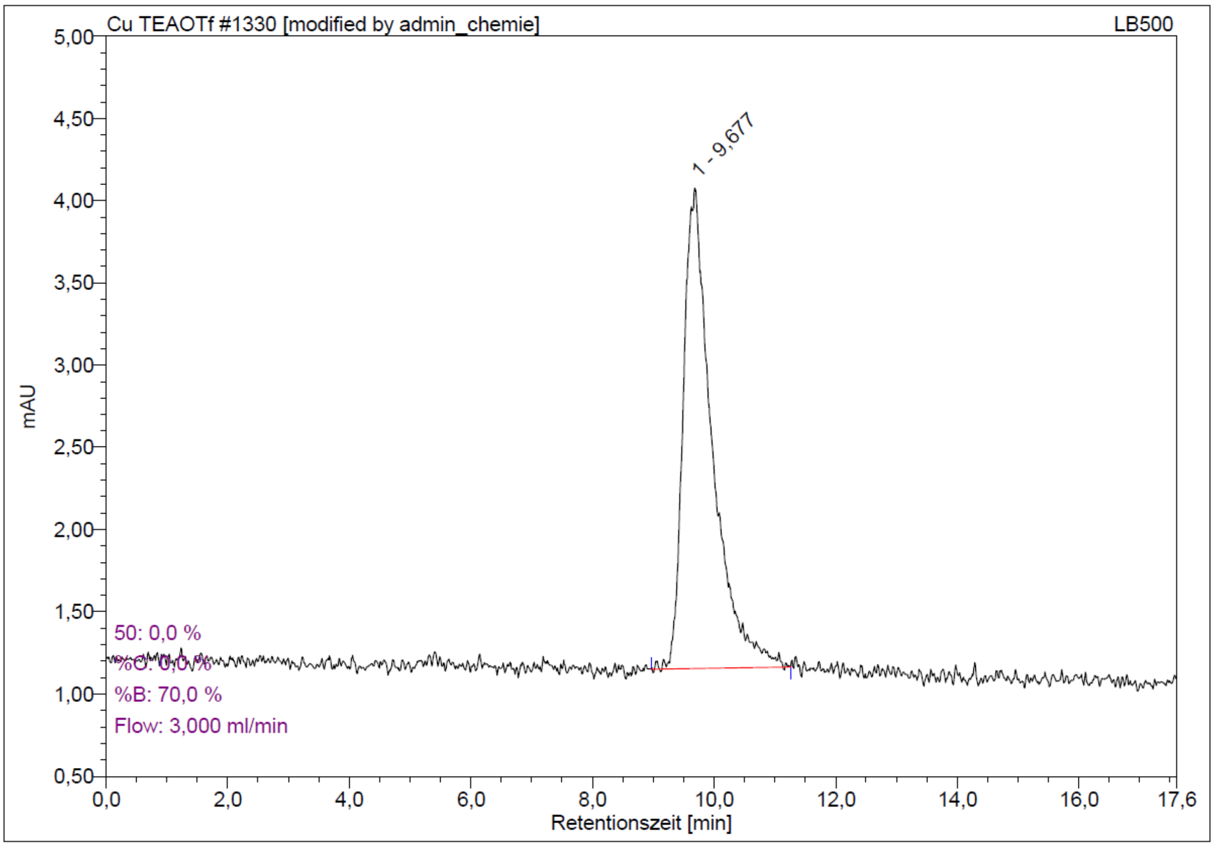

Control of the enantiomeric purity of (*S*)-N_{In}-Me-6-[^{18}F]FTrp [column: Astec Chirobiotic T, 5 µm, 250 × 10 mm (Supelco Analytical) equipped with the appropriate SecurityGuard™ cartridge (2 × 3 mm); eluent: 70% MeOH (0.02% formic acid); flow rate: 3 mL/min].

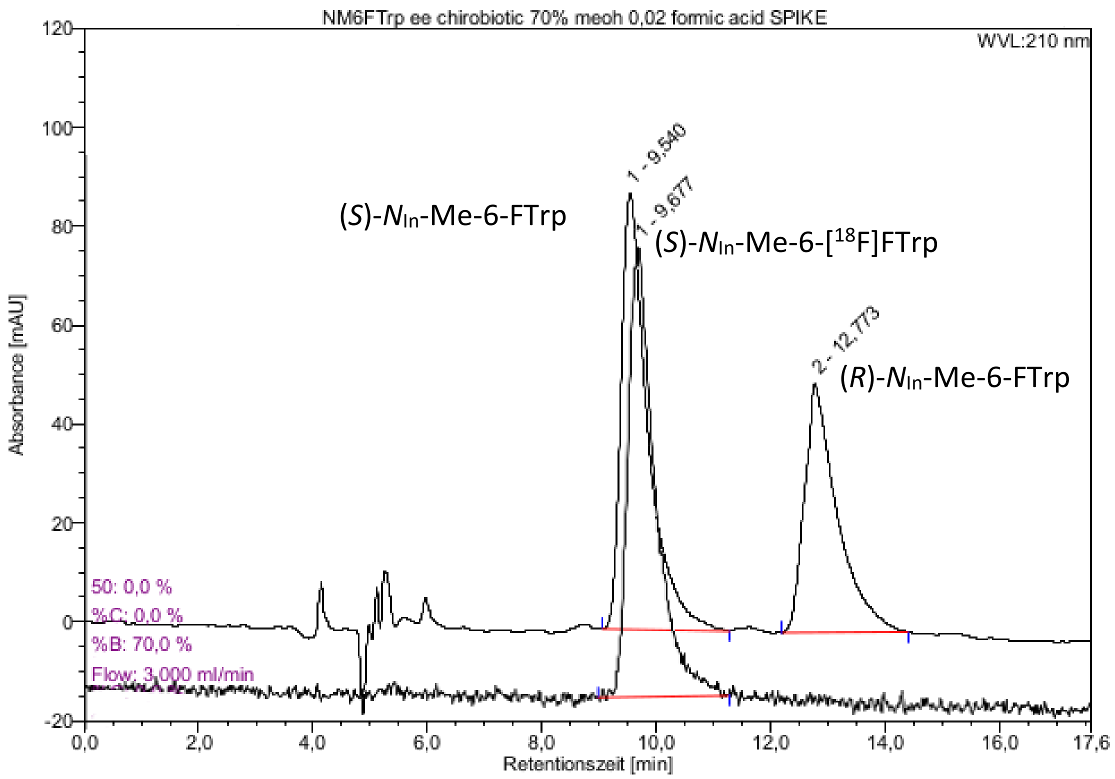

UV and radioactivity traces of the HPLC-chromatogram of (*S*)-N_{In}-Me-6-[^{18}F]FTrp (radioactivity trace) spiked with the racemic non-radioactive reference compound (UV trace, $\lambda = 210$ nm) [column: Astec Chirobiotic T, 5 µm, 250 × 10 mm (Supelco Analytical) equipped with the appropriate SecurityGuard™ cartridge (2 × 3 mm); eluent: 70% MeOH (0.02% formic acid); flow rate: 3 mL/min].

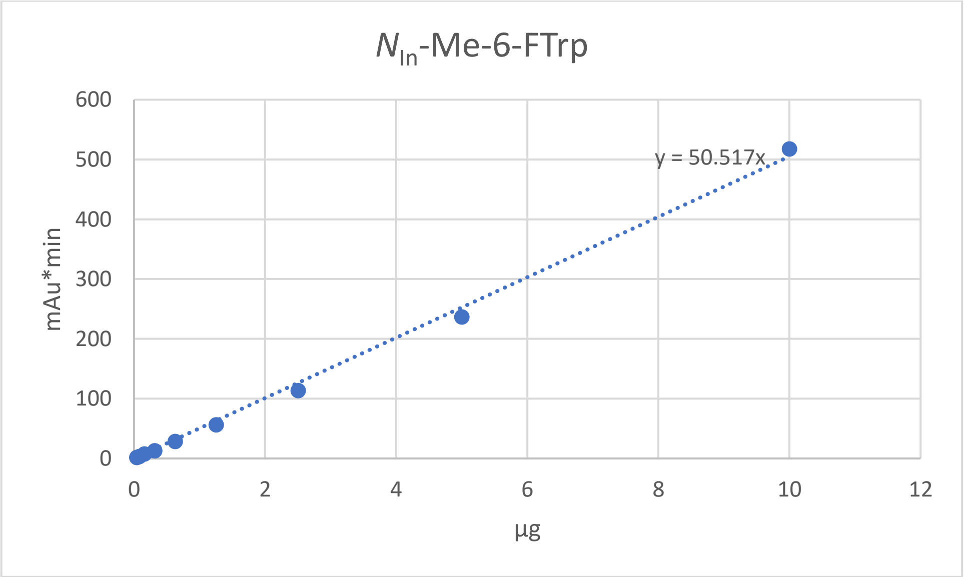

Calibration curve for the determination of molar activity of N_{In}-Me-6-[^{18}F]FTrp [column: Synergi Hydro-RP, 4 µm, 80 Å, 250 × 4.6 mm equipped with the appropriate SecurityGuard™ cartridge (2 × 3 mm) (Phenomenex, Aschaffenburg, Germany); eluent: 20% MeCN (0.1% TFA); flow rate: 1 mL/min; detection: UV: $\lambda = 210$ nm].

3.2.2 (*S*)-5-Hydroxy-7-[^{18}F]fluorotryptophan: HPLC-chromatograms and calibration curve for the determination of molar activity

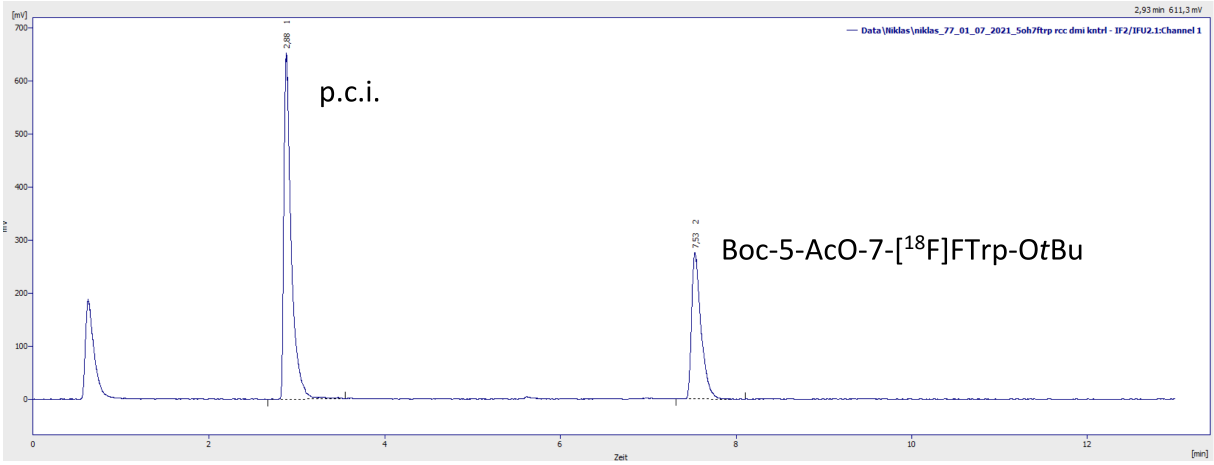

HPLC chromatogram of crude Boc-5-AcO-7-[^{18}F]FTrp-O*t*Bu. Abbreviation: p.c.i – post-column injection.

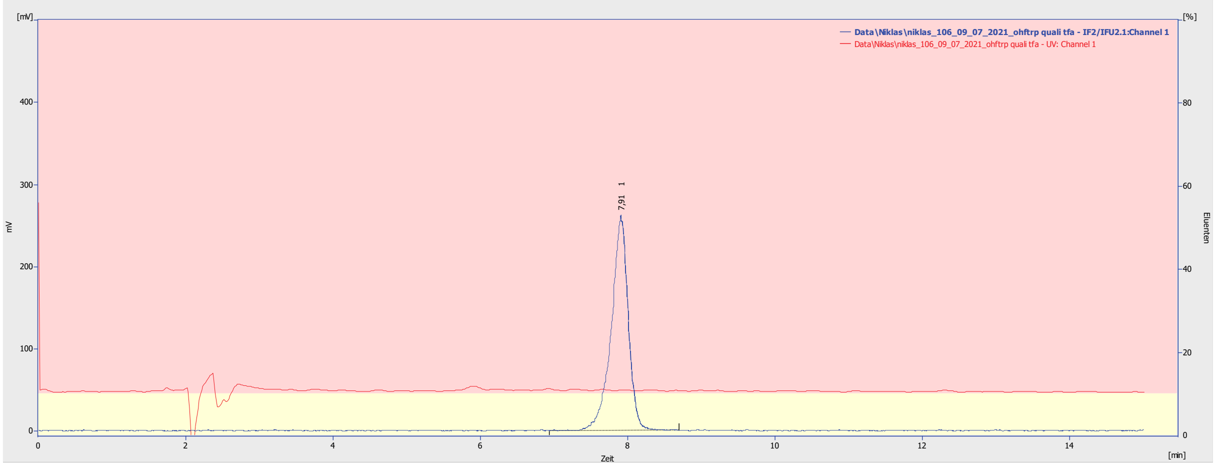

HPLC chromatogram of purified 5-HO-7-[^{18}F]FTrp [column: Synergi Hydro-RP, 4 µm, 80 Å, 250 × 4.6 mm equipped with the appropriate SecurityGuard™ cartridge (2 × 3 mm) (Phenomenex, Aschaffenburg, Germany); eluent: 10% MeCN (0.1% TFA); flow rate: 1.5 mL/min; detection: radioactivity (blue trace), UV: λ = 210 nm (red trace)].

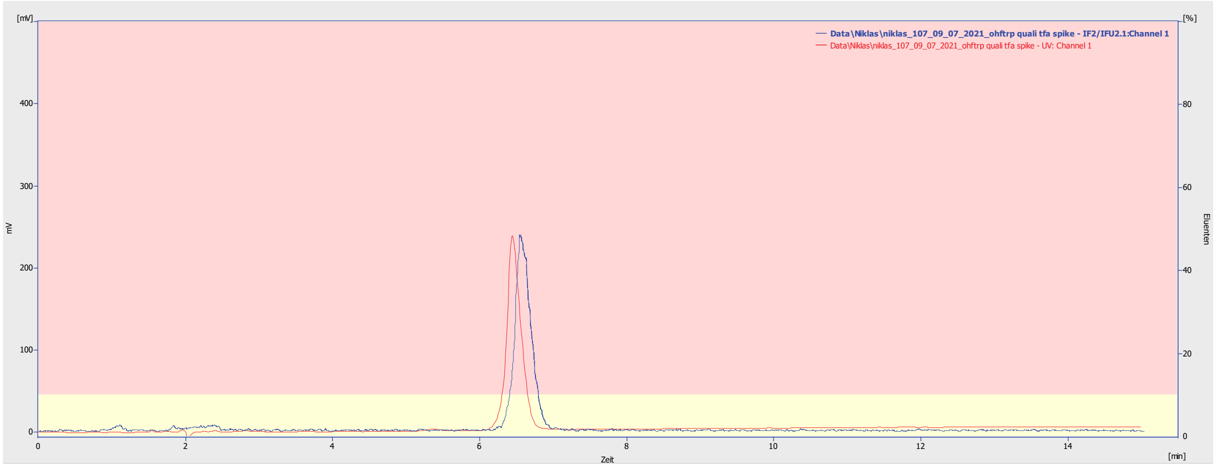

HPLC chromatogram of the 5-HO-7-[^{18}F]FTrp spiked with the non-radioactive reference compound. The radioactive trace is shown in blue, while the UV trace (λ = 210 nm) is shown in red (column: Synergi Hydro-RP, 4 μm, 80 Å, 250 × 4.6 mm equipped with the appropriate SecurityGuard™ cartridge (2 × 3 mm) (Phenomenex, Aschaffenburg, Germany); eluent: 10% MeCN (0.1% TFA); flow rate: 1.5 mL/min).

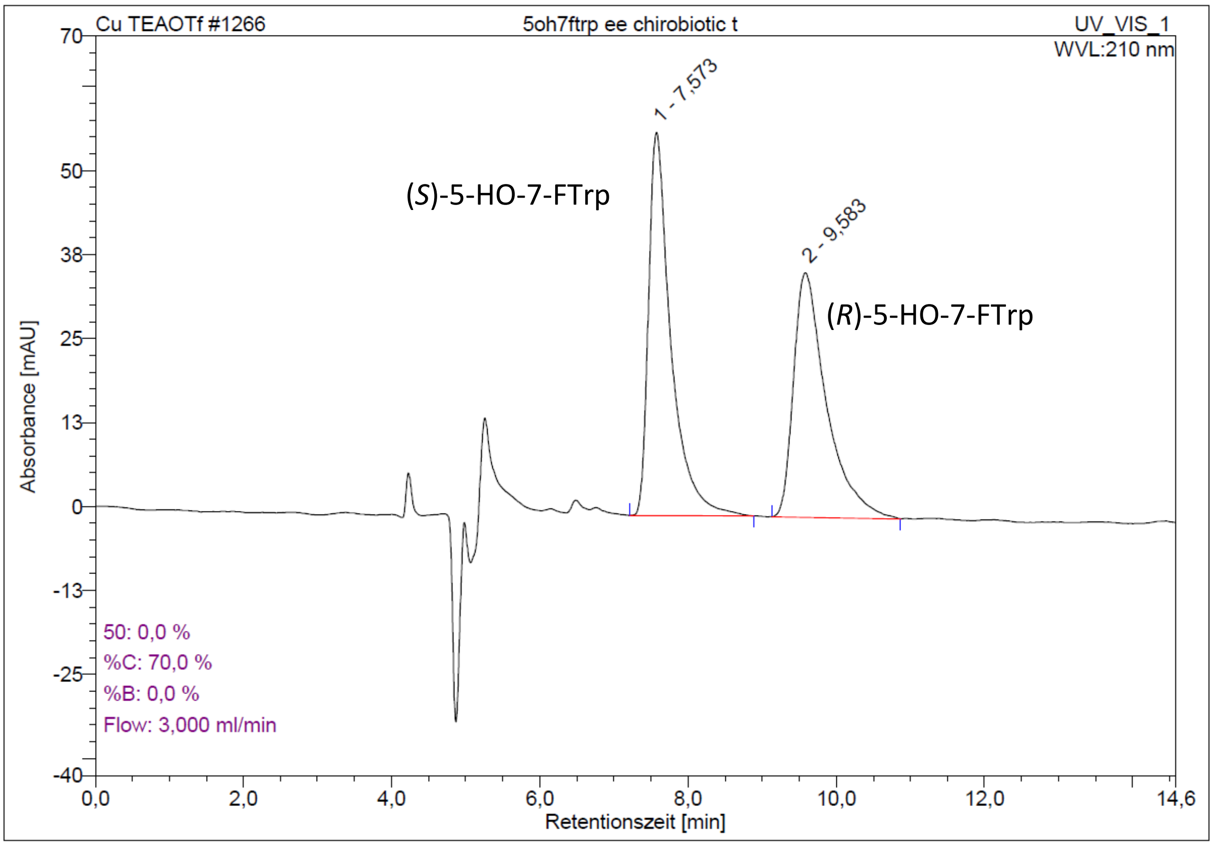

HPLC chromatogram of the racemic 5-HO-7-FTrp [column: Astec Chirobiotic T, 5 μm, 250 × 10 mm (Supelco Analytical) equipped with the appropriate SecurityGuard™ cartridge (2 × 3 mm); eluent: 70% MeOH (0.02% formic acid); flow rate: 3 mL/min, detection: UV: λ = 210 nm].

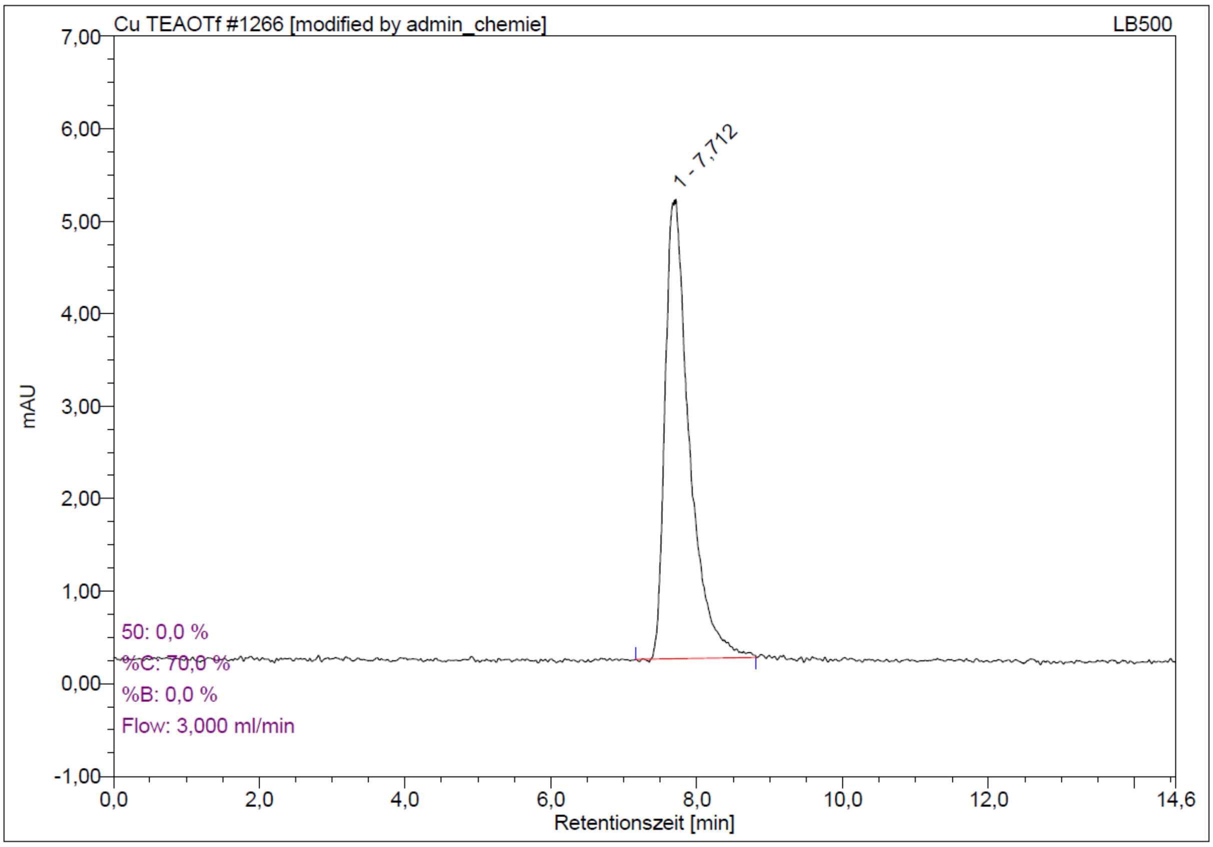

Determination of enantiomeric purity of 5-HO-7-[^{18}F]FTrp [column: Astec Chirobiotic T, 5 µm, 250 × 10 mm (Supelco Analytical) equipped with the appropriate SecurityGuard™ cartridge (2 × 3 mm); eluent: 70% MeOH (0.02% formic acid); flow rate: 3 mL/min].

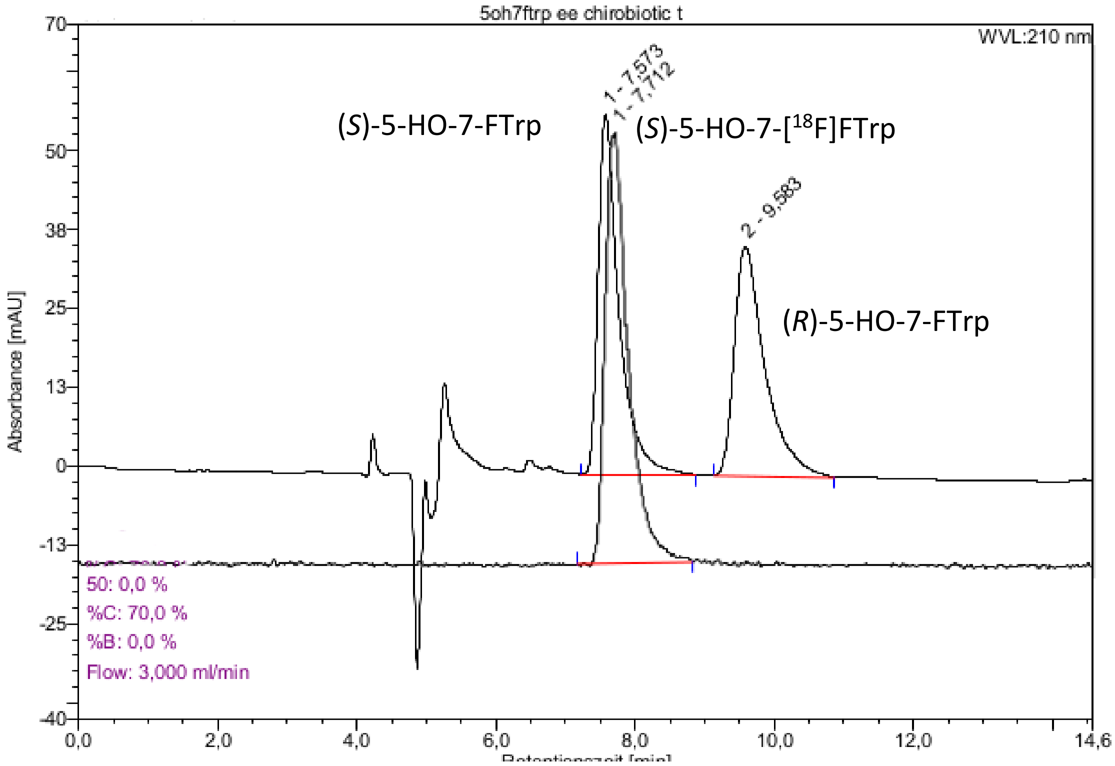

UV and radioactivity traces of the HPLC-chromatogram of 5-HO-7-[^{18}F]FTrp spiked with the racemic reference compounds. [Column: Astec Chirobiotic T, 5 µm, 250 × 10 mm (Supelco Analytical) equipped with the appropriate SecurityGuard™ cartridge (2 × 3 mm); eluent: 70% MeOH (0.02% formic acid); flow rate: 3 mL/min, detection: radioactivity, UV: λ = 210 nm].

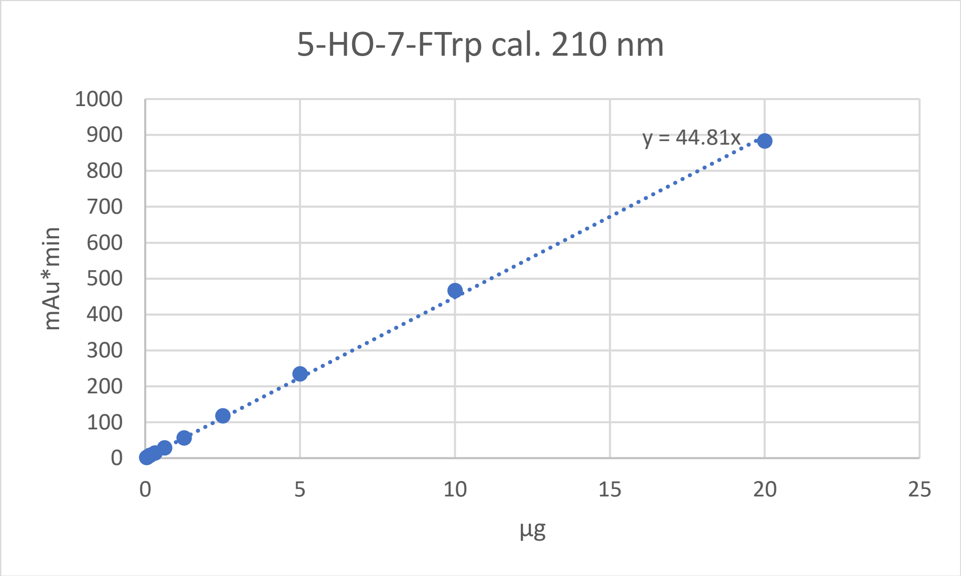

Calibration curve for the determination of molar activity of 5-HO-7-[^{18}F]FTrp. [Column: Synergi Hydro-RP, 4 µm, 80 Å, 250 × 4.6 mm equipped with the appropriate SecurityGuard™ cartridge (2 × 3 mm) (Phenomenex, Aschaffenburg, Germany); eluent: 10% MeCN (0.1% TFA); flow rate: 1.5 mL/min; detection: UV, λ = 210 nm].

4 References

1. Schlosser, M.; Ginanneschi, A.; Leroux, F. In Search of Simplicity and Flexibility: A Rational Access to Twelve Fluoroindolecarboxylic Acids. *European J. Org. Chem.* **2006**, *2006*, 2956–2969, doi:10.1002/ejoc.200600118.

2. Somei, M.; Kizu, K.; Kunimoto, M.; Yamada, F. The Chemistry of Indoles. XXIV. Syntheses of 3-Indoleacetic Acid and 3-Indoleacetonitrile Having a Halogeno Group and a Carbon Functional Group at the 4-Position. *Chem. Pharm. Bull.* **1985**, *33*, 3696–3708, doi:10.1248/cpb.33.3696.

3. Lutz, C.; Simon, W.; Werner-Simon, S.; Müller, C.; Hechler, T.; Kulke, M. Method for Synthesizing Amanitins. US/10961277 B2, 2021.

4. Orlovskaya, V. V.; Modemann, D.J.; Kuznetsova, O.F.; Fedorova, O.S.; Urusova, E.A.; Kolks, N.; Neumaier, B.; Krasikova, R.N.; Zlatopolskiy, B.D. Alcohol-Supported Cu-Mediated 18F-Fluorination of Iodonium Salts under "Minimalist" Conditions. *Molecules* **2019**, *24*, 3197, doi:10.3390/molecules24173197.

5. Lohray, B.B.; Bhushan, V.; Rao, B.P.; Madhavan, G.R.; Murali, N.; Rao, K.N.; Reddy, A.K.; Rajesh, B.M.; Reddy, P.G.; Chakrabarti, R.; et al. Novel Euglycemic and Hypolipidemic Agents. 1. *J. Med. Chem.* **1998**, *41*, 1619–1630, doi:10.1021/jm970444e.

6. Ishiyama, T.; Ishida, K.; Miyaura, N. Synthesis of Pinacol Arylboronates via Cross-Coupling Reaction of Bis(Pinacolato)Diboron with Chloroarenes Catalyzed by Palladium(0)–Tricyclohexylphosphine Complexes. *Tetrahedron* **2001**, *57*, 9813–9816, doi:10.1016/S0040-4020(01)00998-X.

7. Belokon, Y.N.; Bulychev, A.G.; Vitt, S. V.; Struchkov, Y.T.; Batsanov, A.S.; Timofeeva, T. V.; Tsyryapkin, V.A.; Ryzhov, M.G.; Lysova, L.A. General Method of Diastereo- and Enantioselective Synthesis of β-Hydroxy-α-Amino Acids by Condensation of Aldehydes and Ketones with Glycine. *J. Am. Chem. Soc.* **1985**, *107*, 4252–4259, doi:10.1021/ja00300a030.

8. Modemann, D.; Zlatopolskiy, B.D.; Urusova, E.; Zischler, J.; Craig, A.; Ermert, J.; Guliyev, M.; Endepols, H.; Neumaier, B. 2-[18F]Fluorophenylalanine: Synthesis by Nucleophilic 18F-Fluorination and Preliminary Biological Evaluation. *Synthesis (Stuttg).* **2019**, *51*, 664–676, doi:10.1055/s-0037-1611370.
